# Unanticipated intermediates during isopenicillin N synthase catalysis identified by time-resolved X-ray free-electron laser studies

**DOI:** 10.1038/s41929-026-01618-4

**Published:** 2026-10-09

**Authors:** Patrick Rabe, Hiroki Makita, Rebecca Southwart, Midhun George Thomas, Mihails Kazaks, Shyam Basak, Tiankun Zhou, William K. Myers, Margaret D. Doyle, Philipp S. Simon, Shusaku Hayama, J. Frederick W. Mosselmans, Alexander T. Stead, Abraham O. Oluwole, Malkit Sami, Ian Clifton, Pierre Aller, Asmit Bhowmick, Anthony Tumber, Jos J. A. G. Kamps, Karina Calvopiña, Agata Butryn, Robert Bosman, Arjun Cheema, Jordan Welsh, James D. S. Linyard, Frederick J. H. Foster, Pauline A. Lang, Mariska de Munnik, Stephen M. Keable, Medhanjali Dasgupta, In-Sik Kim, Isabel Bogacz, Kuntal Chatterjee, Kyle D. Sutherlin, Corey J. Kaminsky, Jürgen Brem, Michael A. McDonough, Christina Redfield, James R. D. Montgomery, Coral Mycroft, Anastasya Shilova, Nicholas Devenish, Frederic P. Poitevin, Sebastian Dehe, Vandana Tiwari, Humberto Sanchez, Daniel J. Rosenberg, Mark S. Hunter, Leland B. Gee, Uwe Bergmann, Roberto Alonso-Mori, Jaehyun Park, Shigeki Owada, Kensuke Tono, Danny Axford, Robin L. Owen, Daniel W. Paley, Nicholas K. Sauter, David Mittan-Moreau, Aaron S. Brewster, Carol V. Robinson, Vittal K. Yachandra, Junko Yano, Edgars Suna, Christo Z. Christov, Allen M. Orville, Jan F. Kern, Christopher J. Schofield

**Affiliations:** 1 Chemistry Research Laboratory, Department of Chemistry, University of Oxford, Oxford, UK; 2 Diamond Light Source, Diamond House, Harwell Science and Innovation Campus, Didcot, UK; 3 Molecular Biophysics and Integrated Bioimaging Division, Lawrence Berkeley National Laboratory, Berkeley, CA USA; 4 Department of Chemistry, Michigan Technological University, Houghton, MI USA; 5 Latvian Institute of Organic Synthesis, Riga, Latvia; 6 Ineos Oxford Institute for Antimicrobial Research, University of Oxford, Oxford, UK; 7 Research Complex at Harwell, Harwell Science and Innovation Campus, Didcot, UK; 8 Centre for Advanced Electron Spin Resonance, University of Oxford, Oxford, UK; 9 The Kavli Institute for Nanoscience Discovery, University of Oxford, Oxford, UK; 10 Department of Biochemistry, University of Oxford, Oxford, UK; 11 Linac Coherent Light Source, SLAC National Accelerator Laboratory, Menlo Park, CA USA; 12 Department of Physics, University of Wisconsin-Madison, Madison, WI USA; 13 Pohang Accelerator Laboratory, Pohang University of Science and Technology, Pohang, Republic of Korea; 14 RIKEN SPring-8 Center, Sayo-gun, Japan; 15 Japan Synchrotron Radiation Research Institute, Sayo-gun, Japan; 16 Present Address: European Synchrotron Radiation Facility, Grenoble, France; 17 Present Address: Francis Crick Institute, London, UK; 18 Present Address: University Medical Center Hamburg-Eppendorf (UKE), Hamburg, Germany; 19 Present Address: Oak Ridge National Laboratory, Oak Ridge, TN USA; 20 Present Address: University of Nebraska Medical Center, Omaha, NE USA; 21 Present Address: APS BioCars, University of Chicago, Chicago, IL USA; 22 Present Address: Lam Research, Tualatin, OR USA; 23 Present Address: ExxonMobil, Annandale, NJ USA; 24 Present Address: Enzymology and Applied Biocatalysis Research Center, Babes-Bolyai University, Cluj-Napoca, Romania

**Keywords:** Structural biology, Biocatalysis, Enzyme mechanisms, Oxidoreductases

## Abstract

Biosynthesis of penicillin and cephalosporin antibiotics involves the oxidation of the tripeptide δ-(l-α-aminoadipoyl)-l-cysteinyl-d-valine (ACV) to give the conformationally strained bicyclic penicillin ring system, as catalysed by the Fe(II)-dependent oxidase isopenicillin N synthase (IPNS). The structures of the precursors for the sequential β-lactam and thiazolidine ring forming steps in IPNS have remained elusive. Here we studied the reaction of anaerobic IPNS:Fe(II):ACV microcrystals with O_2_ using time-resolved serial femtosecond crystallography and X-ray emission spectroscopy. High-resolution structures reveal that ACV cysteinyl oxidation produces a thioaldehyde that unexpectedly does not coordinate the iron centre. The use of deuterium-labelled ACV enabled the observation of a monocyclic β-lactam with restoration of the Fe–S bond. Together with mutagenesis, spectroscopic and computational studies, the results support Fe(IV)-mediated penicillin formation involving active-site water molecules. These findings provide structural insight into IPNS catalysis, inform on the wider Fe(II) oxygenase superfamily and highlight the power of X-ray free-electron laser methods for resolving transient enzymatic intermediates.

**Figure:**
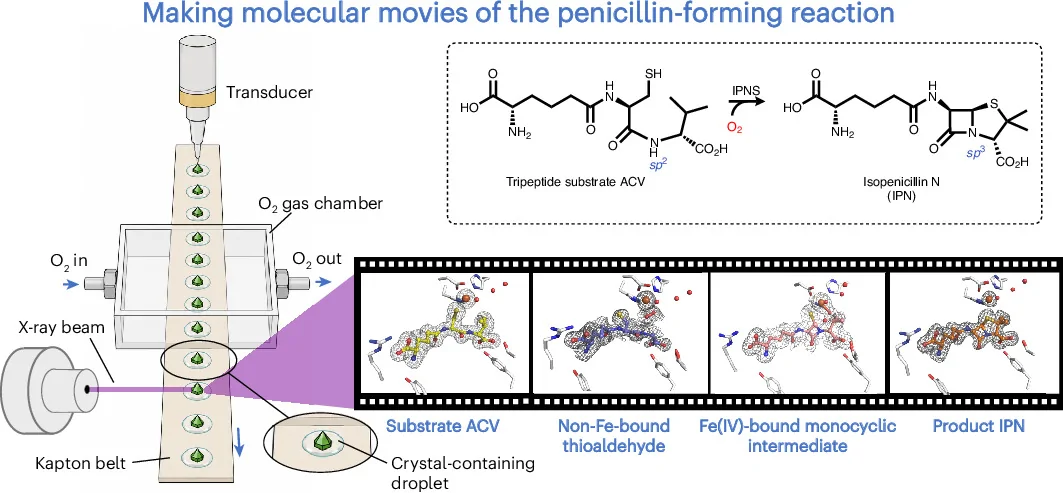

## Abstract (web summary)

The structural basis of key intermediates in isopenicillin N synthase catalysis, underlying β-lactam biosynthesis, has remained incomplete. Now time-resolved X-ray free-electron laser studies capture transient states and reveal how protein dynamics and solvent networks govern oxygen activation and ring formation.

## Main

Understanding how the precise positioning and dynamics of atoms within enzymes enable catalysis involving high-energy intermediates remains a central challenge in structural biology. Isopenicillin N synthase (IPNS) catalyses the biosynthesis of isopenicillin N (IPN), forming the fused four-membered β-lactam and five-membered thiazolidine ring system that is characteristic of the penicillins^1^. The unique iron- and dioxygen-dependent IPNS-catalysed reaction^2^ involves two energetically demanding C–H bond cleavage steps, both exhibiting substantial primary kinetic isotope effects (KIE)^3,4^. IPNS belongs to the widespread Fe(II)/2-oxoglutarate (2OG) oxygenase structural superfamily, members of which have diverse roles, including in collagen biosynthesis, lipid metabolism, nucleic acid repair and signalling^5–7^. While most 2OG oxygenases catalyse two-electron hydroxylation reactions that are coupled to oxidative decarboxylation of 2OG, IPNS performs a four-electron oxidation, transforming the tripeptide δ-(l-α-aminoadipoyl)-l-cysteinyl-d-valine (ACV) into the ring-strained penicillin scaffold^4,8^.

**Fig. 1:**
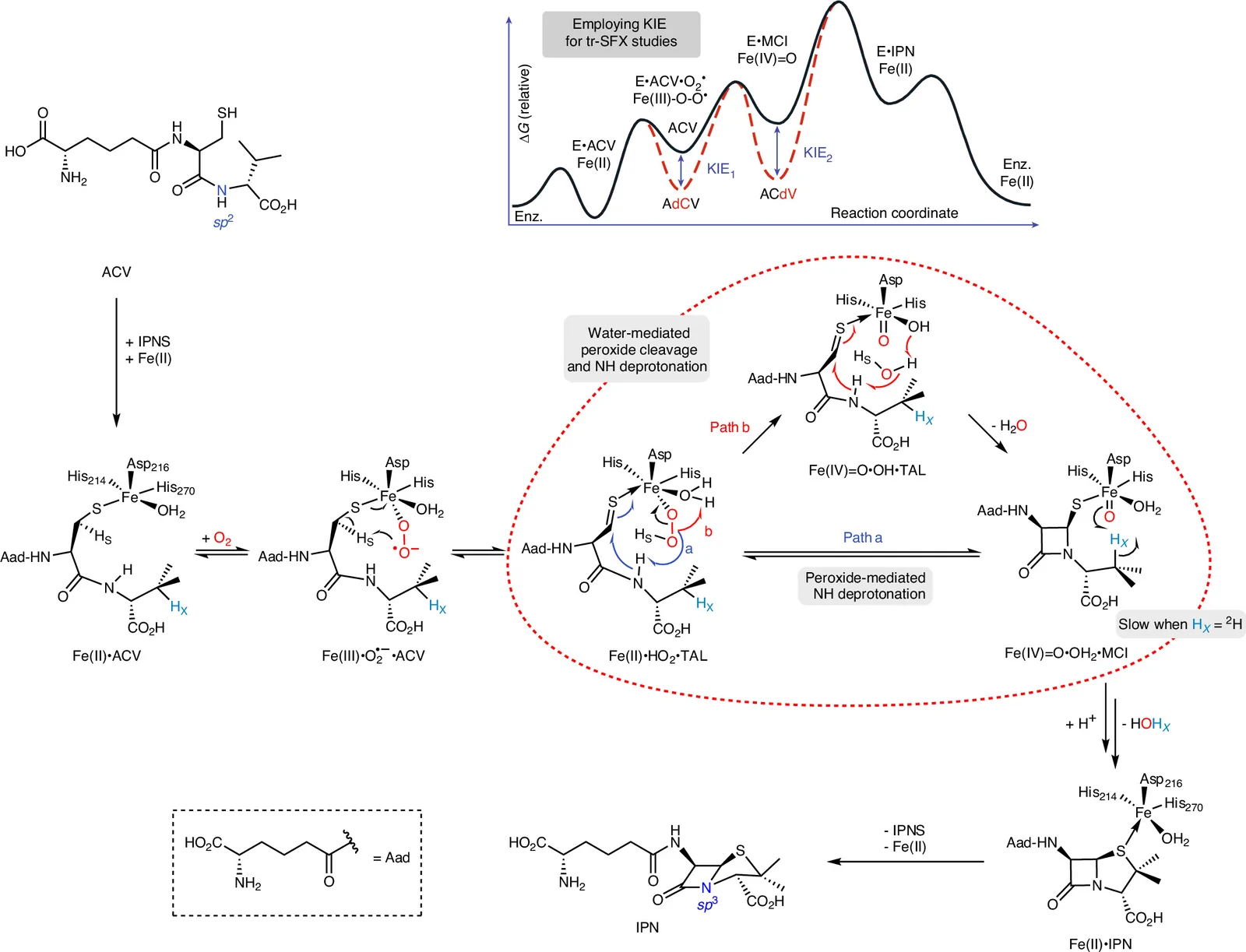
Overview of IPNS catalysis. IPNS catalyses the four-electron oxidation of ACV, giving IPN via a proposed Fe–S linked MCI. Reaction with O_2_ forms an Fe(III)-superoxo species^2,11^ that enables Cys-CH_2_ *pro*-(*S*)*-*hydrogen abstraction giving a putative Fe(II)-hydroperoxo TAL. Two mechanisms are proposed for the O–O bond cleavage and ACV valine NH abstraction steps: (path a) direct peroxide-mediated NH deprotonation or (path b) peroxide-derived water valine NH abstraction^2,12–14^; both lead to an Fe(IV)-MCI capable of Val-β CH deprotonation, enabling thiazolidine ring formation. The inset shows a qualitative reaction energy profile illustrating the proposed sequence of intermediates and transition states during IPNS catalysis. β-Lactam and thiazolidine ring formation are subject to primary KIEs as observed with l-δ-(α-aminoadipoyl)-L-(3,3-^2^H_2_)-cysteinyl-d-valine (AdCV, KIE_1_) and l-δ-(α-aminoadipoyl)-l-cysteinyl-d-(3-^2^H)-valine (ACdV, KIE_2_), respectively^3,4,21^. We used ACdV (H_*x*_ = ^2^H) in tr-SFX experiments to trap the MCI. Source data

X-ray diffraction analyses employing single crystals of the anaerobic IPNS:Fe:ACV complex have revealed that ACV ligates the iron via its sulfur atom^2,4,8–10^. Kinetics, spectroscopy and computational studies using ACV and peptide substrate analogues show that O_2_ binds to the IPNS:Fe(II):ACV complex forming a ferric superoxo intermediate, proposed to abstract a hydrogen from the cysteinyl β-carbon, generating a thioaldehyde (TAL) and an Fe(II)-hydroperoxide intermediate^2,11^. Computational studies have proposed two possible pathways for β-lactam formation^2,12–14^. In one possible pathway, the ACV valine amide NH directly transfers a proton to the superoxo species, triggering O–O bond cleavage and formation of an Fe(IV)=O species (Fig. 1, path a). In the other pathway, the distal superoxo oxygen abstracts a proton from an Fe-ligating water, producing a water molecule and an Fe(IV)=O(OH) species; the Fe–OH then removes the valine NH proton via a water-mediated relay process (Fig. 1, path b). Both pathways converge on a monocyclic intermediate (MCI) linked via the ACV cysteinyl derived sulfur to an Fe(IV)=O(OH_2_) species; the latter abstracts the valine β-CH, enabling thiazolidine ring closure and IPN formation^2^.

Recent time-resolved serial femtosecond crystallography (tr-SFX) studies using X-ray free-electron lasers (XFEL), combined with solution spectroscopy, have revealed O_2_ binding induces correlated motions involving helix α3 and β-strand β11, which are linked to transient changes in active-site shape^11,15^. The structures of the proposed Fe(II)-hydroperoxide.TAL and Fe(IV)=O.MCI intermediates involved in the key ring forming reactions have, however, remained elusive. In particular, the roles of active-site water molecules and their involvement in proton transfers during catalysis have been largely unresolved.

To address these questions, we employed tr-SFX coupled with X-ray emission spectroscopy (XES) to enable analysis of the electronic structure of iron at different points across the reaction coordinate^16–18^. This approach enables interrogation of radiation-sensitive high-valent Fe intermediates under damage-minimizing XFEL conditions. We used the acoustic droplet ejection (ADE) tape-drive method to react anaerobic IPNS:Fe(II):ACV microcrystals with O_2_ (refs. ^19,20^), exploiting the known KIE with deuterated AC(3-^2^H)V (ACdV)^3,4,21^, to characterize the MCI and high-valent iron species that are critical to catalysis. Together with subsequent solution and mutagenesis studies, the results reveal unexpected insight into the IPNS reaction mechanism including the vital roles of active-site water molecules.

## Results

### Characterization of early intermediates during IPNS catalysis

We first describe how tr-SFX studies enabled us to characterize the structures of intermediates during IPNS catalysis, then integrate these observations with mutagenesis and computational analyses, so providing a revised sequence of events during IPNS catalysis. We hypothesized that the large primary KIE (~30)^4,21^ observed with l-δ-(α-aminoadipoyl)-l-cysteinyl-(3-^2^H)-d-valine (ACdV) (synthesis outlined in Supplementary Figs. 1–3) during IPNS catalysis in solution would increase the concentration of the proposed Fe(IV)-linked MCI during turnover in crystals.

**Fig. 2:**
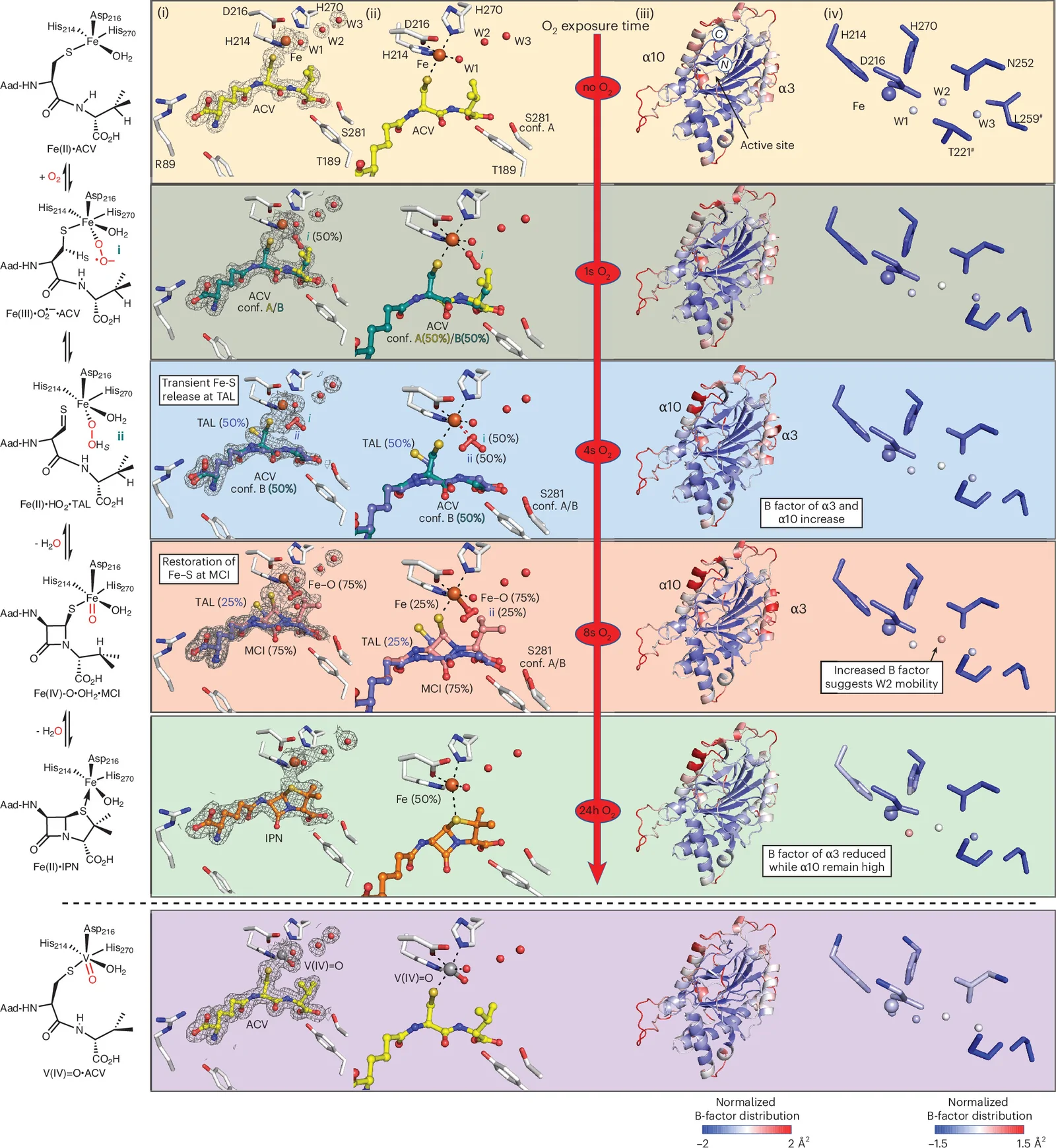
Time-resolved structural snapshots of IPNS catalysis by tr-SFX. (i) Refined 2F_o_–F_c_ electron density maps (1.0 σ contour level, carved around substrate/intermediate/product/O_2_) and (ii) active-site views for IPNS:Fe:ACdV (PDB: 8PY6; 1.52-Å resolution, anaerobic, yellow, LCLS, SFX), IPNS:Fe:ACdV:O_2_ (superoxo intermediate, PDB: 8PY7; 1.49-Å resolution, 1 s timepoint, pale green, LCLS, SFX), IPNS:Fe:ACV/TAL:O_2_ (superoxo/hydroperoxo intermediate, PDB: 8PYA; 1.50-Å resolution, 4 s O_2_, blue, LCLS, SFX), IPNS:Fe/Fe=O:TAL/MCI (PDB: 9I3B; 1.60-Å resolution, 8 s O_2_, red, LCLS, SFX), IPNS:Fe:IPN (product complex, PDB: 9QPH; 1.70-Å resolution, green, LCLS, SFX) and IPNS:VO:ACV (Fe(IV)=O mimic, PDB: 6ZAK; 1.54-Å resolution, purple, SACLA, SFX) revealing changes along the reaction coordinate. (iii) Normalized backbone-carbon B-factor analysis highlighting dynamic regions of the α3 and α10 helices. Note, the α3 B-factors are lower in the IPN complex, but for α10 remain high (compared to intermediate states). (iv) Normalized B-factor analysis including ligands/waters showing the dynamic nature of W2 at the MCI stage, supporting its involvement in catalysis (Extended Data Fig. 1). Note, the superoxo (i) and hydroperoxo (ii) intermediates were observed in two conformations; both were refined as an O_2_-derived species (OXY ligand in the PDB). The major proposed species present at different timepoints are shown on the left. conf., conformation. Source data

**Fig. 3:**
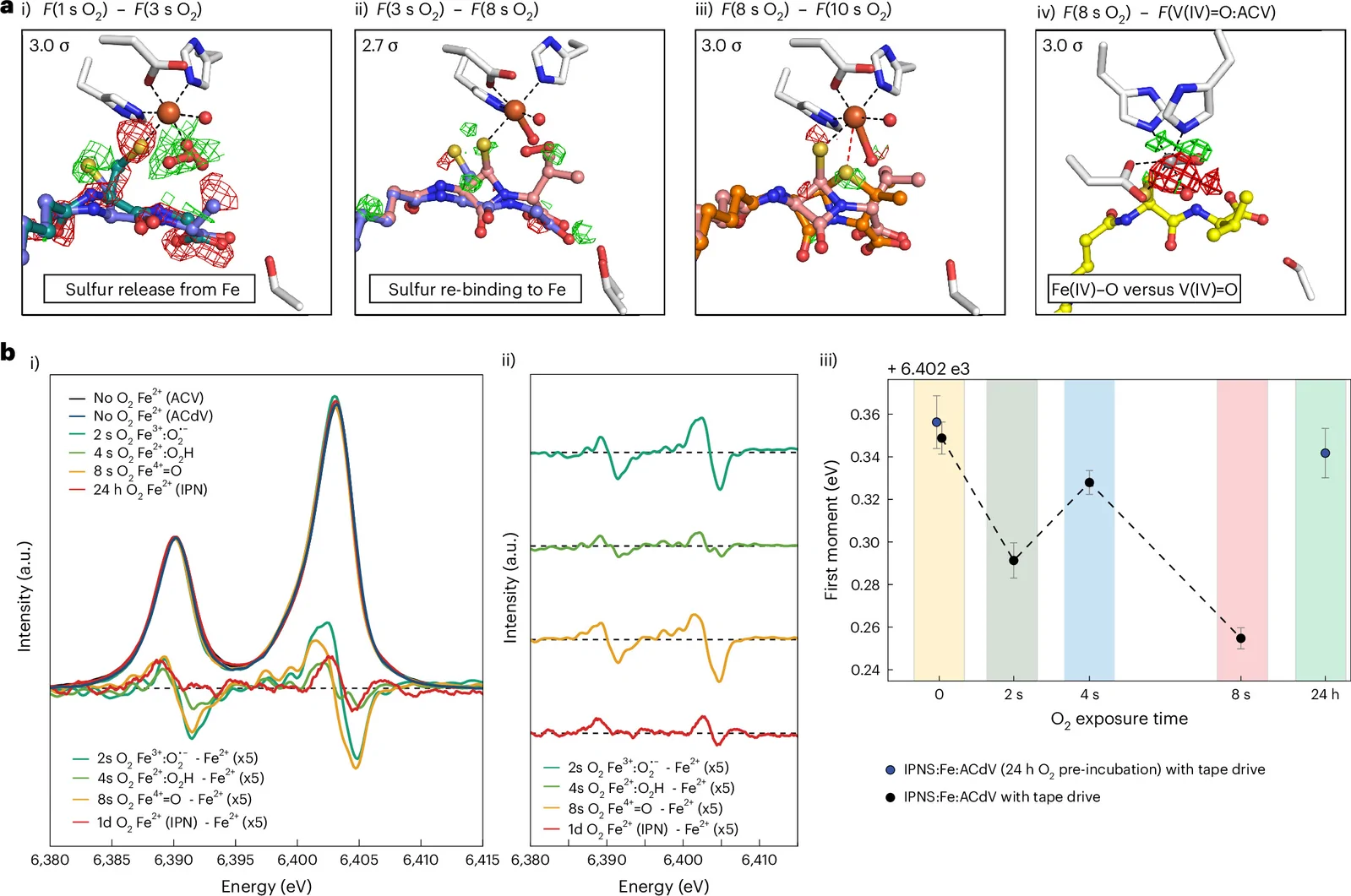
Difference map and XES analyses support transient catalytic intermediates during IPNS catalysis. **a**, *F*_obs_ − *F*_obs_ isomorphous difference maps (2.7–3.0 σ contour level) for (i) IPNS:Fe:ACV:O_2_ (1 s O_2_, PDB: 8PY7) relative to IPNS:Fe:TAL:O_2_H (3 s O_2_, PDB: 8PY9), evidencing a lack of ACV-sulfur coordination in the latter; (ii) IPNS:Fe:TAL:O_2_H (3 s O_2_, PDB: 8PY9) relative to IPNS:Fe=O:MCI (8 s O_2_, PDB: 9I3B), showing restoration of the Fe–S link; (iii) IPNS:Fe=O:MCI (8 s O_2_, PDB: 9I3B) relative to IPNS:Fe=O:MCI/IPNS:Fe:IPN (10 s O_2_, PDB: 9I3D), showing partial formation of IPN; and (iv) IPNS:Fe=O:MCI (8 s O_2_ exposure, PDB: 9I3B) relative to IPNS:V=O:ACV (PDB: 6ZAK), showing different locations of the Fe=O/V=O oxygens. Control data are given in Extended Data Figs. 2, 5 and 6. Note, that in the 3 s timepoint, the partial positional shift of the ACV-derived sulfur occurs with residual occupancy at the original site; accordingly, the positive difference density represents the net shift between overlapping states and is therefore not centred exactly on the refined sulfur position. **b**, (i) Fe Kα_1,2_ XES of anaerobic and O_2_-exposed IPNS:Fe:ACdV microcrystals after 2 s, 4 s, 6 s, 8 s and 24 h O_2_, corresponding to the tr-SFX data in Fig. 2, with difference spectra (×5) between the O_2_-exposed and anaerobic data shown below. (ii) An enlarged stacked view of difference spectra (as shown in (i)) between 2 s, 4 s, 6 s, 8 s and 24 h O_2_-exposed datasets compared with the anaerobic dataset. (iii) The trend of the first moment of the Fe Kα_1_ XES peak following O_2_. The first moment shows a progressive shift of the Kα_1_ peak across the time course, with the largest shift at 8 s. Regeneration of Fe(II) correlates with IPN formation, as observed by tr-SFX. Error bars reflect statistical uncertainties of the spectral analysis and are derived from counting statistics (Supplementary Methods). Data are presented as nominal values calculated on the filtered spectra ± standard deviations calculated using the unfiltered spectra on 98 and 150 data points of the spectra in the Kα_1_ region for the IPNS:Fe:ACdV + O_2_ (black, beam time: LV56) and IPNS:Fe:ACdV with O_2_ pre-incubation (blue, beam time: P-10035) datasets, respectively. See Extended Data Figs. 1, 3 and 4 for additional timepoints, as well as XES IAD and FWHM control data and reference compound analysis^16^. Source data

**Extended Data Fig. 1:**
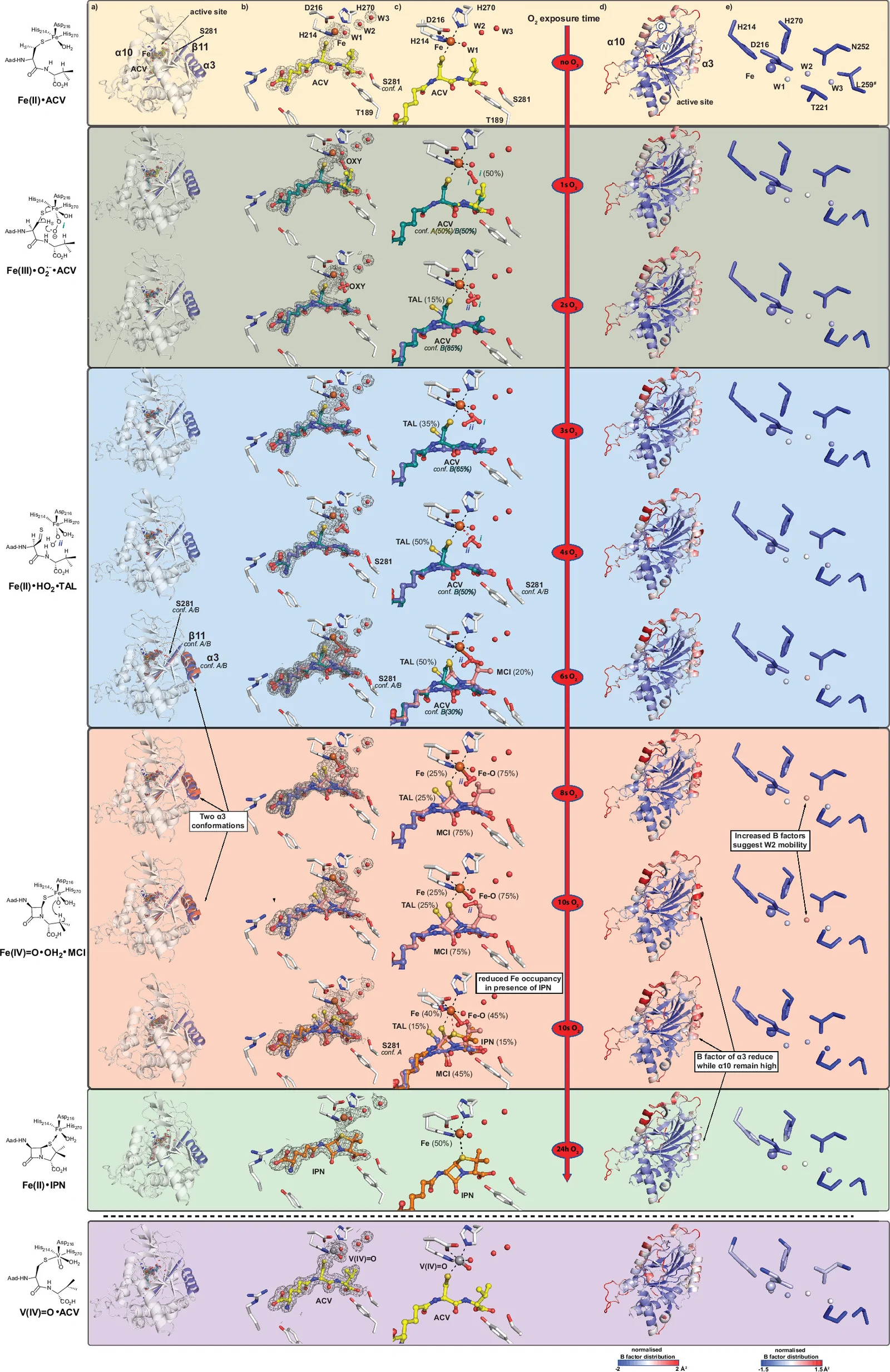
Structural analysis of the reaction cycle and dynamics of IPNS at varying O_2_ exposure times. Tr-SFX structures of IPNS:Fe:ACdV (PDB: 8PY6, 1.52 Å resolution, anaerobic), IPNS:Fe:ACdV:O_2_ (PDB: 8PY7, 1.49 Å resolution, 1 s O_2_), IPNS:Fe:ACdV:O_2_ (PDB: 8PY8, 1.45 Å resolution, 2 s O_2_), IPNS:Fe:ACdV/TAL:O_2_ (PDB: 8PY9, 1.50 Å resolution, 3 s O_2_), IPNS:Fe:ACdV/TAL:O_2_ (PDB: 8PYA, 1.50 Å resolution, 4 s O_2_), IPNS:Fe/Fe=O:TAL/MCI (PDB: 9I3A, 1.52 Å resolution, 6 s O_2_), IPNS:Fe/Fe=O:TAL/MCI (PDB: 9I3B, 1.60 Å resolution, 8 s O_2_), IPNS:Fe/Fe=O:TAL/MCI (PDB: 9I3C, 1.70 Å resolution, 10 s O_2_), IPNS:Fe/Fe=O: MCI/IPN (PDB: 9I3D, 1.68 Å resolution, 10 s O_2_), IPNS:Fe:IPN (PDB: 9QPH, 1.70 Å resolution) and IPNS:VO:ACV (Fe(IV) = O mimic, PDB: 6ZAK, 1.54 Å resolution) at different O_2_ exposure times. Complementary tr-XES is shown in Extended Data Fig. 4. The major proposed species present in the different time points are shown on the left. (**a**) Ribbon views after varying O_2_ times revealing an alternative conformation of α3 after 6 s, 8 s and 10 s O_2_ exposure, with return to the starting conformation in the product (IPN) complex. Active site residues with Fe and ACV and α3 and α10 are highlighted. Crystal lattice interactions which prevent IPN release after O_2_ exposure are shown in Supplementary Fig. 4. (**b**) 2F_o_-F_c_ maps (1.0 σ contour level, carved around substrate/intermediate/product/O_2_) revealing changes in density along the reaction coordinate, indicating initial O_2_ binding, ACV-S release to give a TAL, MCI formation, then IPN formation. Note, as observed in IPNS structures forming penicillins, the β-lactam carbonyl of the MCI and IPN show relatively weak / sometimes poorly defined electron density. (**c**) Active site view of alternative IPNS models after differing O_2_ times highlighting occupancies of different intermediates. Note, the Fe occupancy drops to 50% in the IPN complex^11^. Note: the superoxo (i) and hydroperoxo (ii) intermediates were observed in two conformations; but were refined as an O_2_ molecule (OXY ligand in the PDB). (**d**) Normalized B factor analysis of IPNS backbone carbons highlighting dynamic regions of α3 / α10. Note, while α3 B-factors reduce in the product complex, those of α10 remain high. (**e**) Normalized B factor analysis of IPNS including ligands/waters highlighting a dynamic nature of W2 in Fe=O intermediate after 8 s and 10 s O_2_ suggesting potential W2 involvement during catalysis.

A slurry of IPNS:Fe(II):ACdV microcrystals (~3 μm × 3 μm × 80 μm, needle morphology, *P*2_1_2_1_2_1_ space group) was prepared under anaerobic conditions. To confirm the integrity of the ADE tape-drive XFEL sample delivery system^19^, we collected anaerobic SFX and XES control datasets at both the LCLS and PAL-XFEL, the results demonstrated no reaction and ACdV at full occupancy. Similar to unlabelled ACV^11,22^, the IPNS:Fe(II):ACdV microcrystals diffracted to at least 1.5-Å resolution using the XFEL sources (Supplementary Tables 2 and 3). To initiate reactions, we passed the ~3.9-nl volume droplets through a 100% O_2_-containing reaction chamber^11,19^ (Fig. 2 and Extended Data Fig. 1). Importantly, lattice interactions prevent product release in crystallo, but do not stop O_2_-dependent catalysis. Consequently, we observed a stable IPNS:Fe(II):IPN complex on prolonged O_2_ exposure, thereby enabling single turnover studies on the events involved in conversion of ACV to IPN (Supplementary Fig. 4). As reported, the Fe(II) coordination sphere exhibits octahedral geometry involving His214, Asp216, His270, a water (W1) and the ACV thiolate, with an open coordination site *trans* to Asp216^10^. The XES spectrum was consistent with an initial Fe(II) state (Fig. 3b).

The reaction time was regulated by changing the tape speed and consequently the time delay between O_2_ exposure and X-ray interaction^19^. We collected multiple datasets, at 1 s, 2 s, 3 s, 4 s, 6 s, 8 s and 10 s O_2_ exposure times. To enable robust comparisons, datasets were collected on the same day with the same IPNS:Fe(II):ACdV batch. To minimize bias in data interpretation and eliminate XES signals from tape contamination, the order of data collection was randomized (that is, 3 s, 4 s, 6 s, 1 s, 2 s, 8 s, then 10 s exposure times) and water-only control droplet runs were randomly interspersed. All the XFEL results reported herein were repeated at least twice.

**Extended Data Fig. 2:**
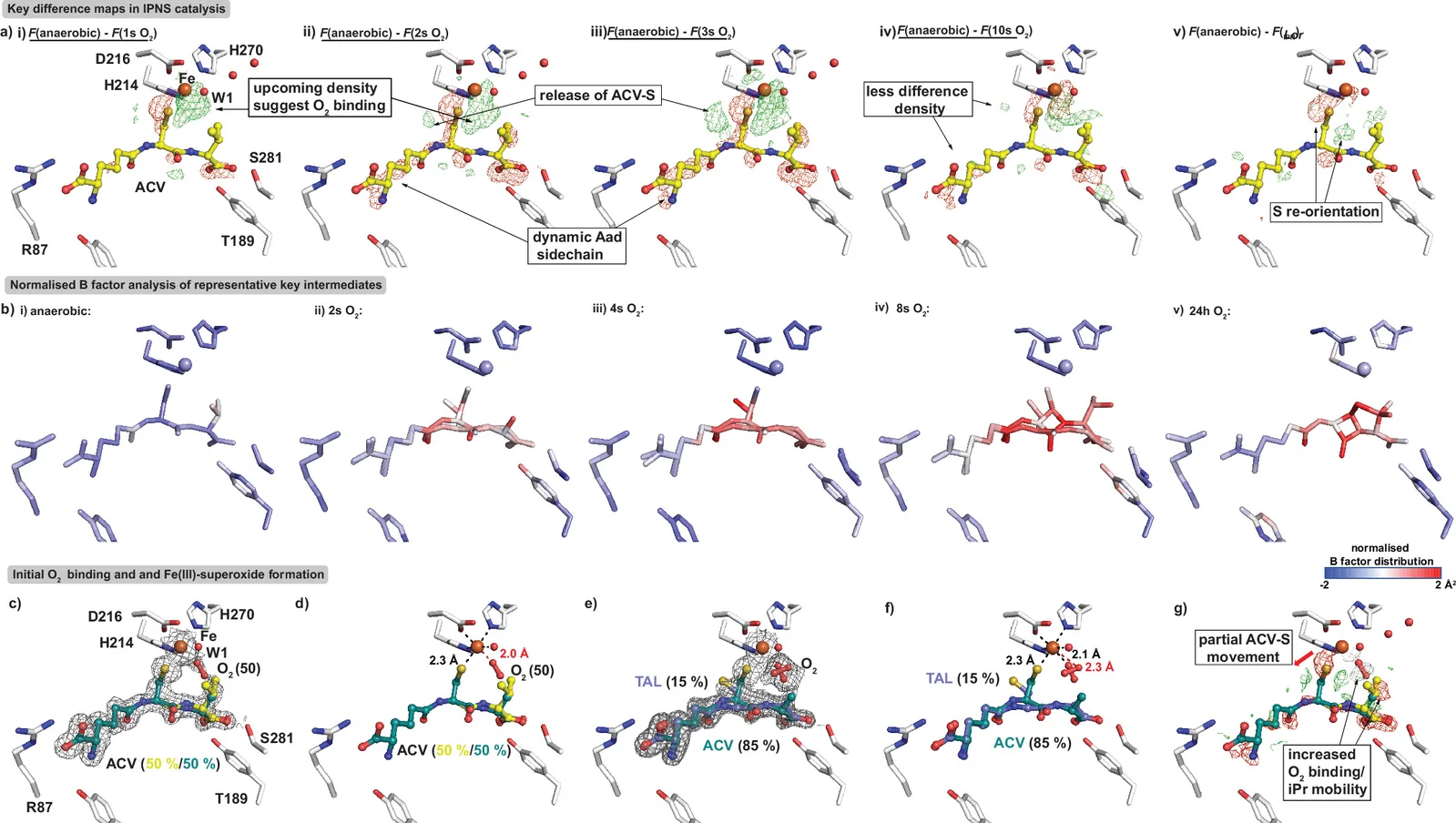
Structural analysis of O_2_ binding and Fe(III) superoxo formation. (**a**) *F*_obs_ − *F*_obs_ isomorphous difference maps of IPNS:Fe:ACdV (PDB: 8PY6, 1.52 Å resolution, anaerobic) with (i) IPNS:Fe:ACV:O_2_ (1 s O_2_ exposure, PDB: 8PY7), (ii) IPNS:Fe:ACdV:O_2_ (PDB: 8PY8, 1.45 Å resolution, 2 s O_2_), (iii) IPNS:Fe:ACdV/TAL:O_2_ (PDB: 8PY9, 1.50 Å resolution, 3 s O_2_), (iv) IPNS:Fe/Fe=O:TAL/MCI (PDB: 9I3B, 1.60 Å resolution, 8 s O_2_) and (v) IPNS:Fe:IPN (PDB: 9QPH, 1.70 Å resolution) suggesting initial O_2_ binding *trans* to D216, accumulation of a transient non Fe-coordinating TAL (from 2 s to 3 s O_2_), followed by its reduction concurrent with Fe-coordinating MCI formation (8 s O_2_), then IPN formation with relocation of the IPN-S. After 2 s and 3 s O_2_ exposure, the increased difference density suggests a mobile Aad sidechain, while after 8 s O_2_, these features decrease consistent with a more ordered Aad sidechain conformation. (**b**) Normalised B factor analysis of the active site of the key intermediates: (i) IPNS:Fe:ACdV (PDB: 8PY6), (ii) IPNS:Fe:ACdV:O_2_ (PDB: 8PY8), (iii) IPNS:Fe:ACdV/TAL:O_2_ (PDB: 8PYA), (iv) IPNS:Fe/Fe=O:TAL/MCI (PDB: 9I3B) and (v) IPNS:Fe:IPN (PDB: 9QPH). c-f) 2F_o_-F_c_ maps (1.0 σ contour level, carved around ACV/TAL/Fe/O_2_) of (**c**) IPNS:Fe:ACV:O_2_ (1 s O_2_ exposure, PDB: 8PY7) and (**e**) IPNS:Fe:ACdV:O_2_ (PDB: 8PY8, 2 s O_2_), with key distances and occupancies shown in panels (**d**) (1 s O_2_) and (**f**) (2 s O_2_). (**g**) *F*_obs_ − *F*_obs_ isomorphous difference maps of IPNS:Fe:ACV:O_2_ (1 s O_2_ exposure, PDB: 8PY7) and IPNS:Fe:ACdV:O_2_ (PDB: 8PY8, 2 s O_2_) reveal increased O_2_ binding, enhanced delocalization of the ACV-Val isopropyl group^11^, and partial release of ACV-S after 2 s O_2_.

**Extended Data Fig. 3:**
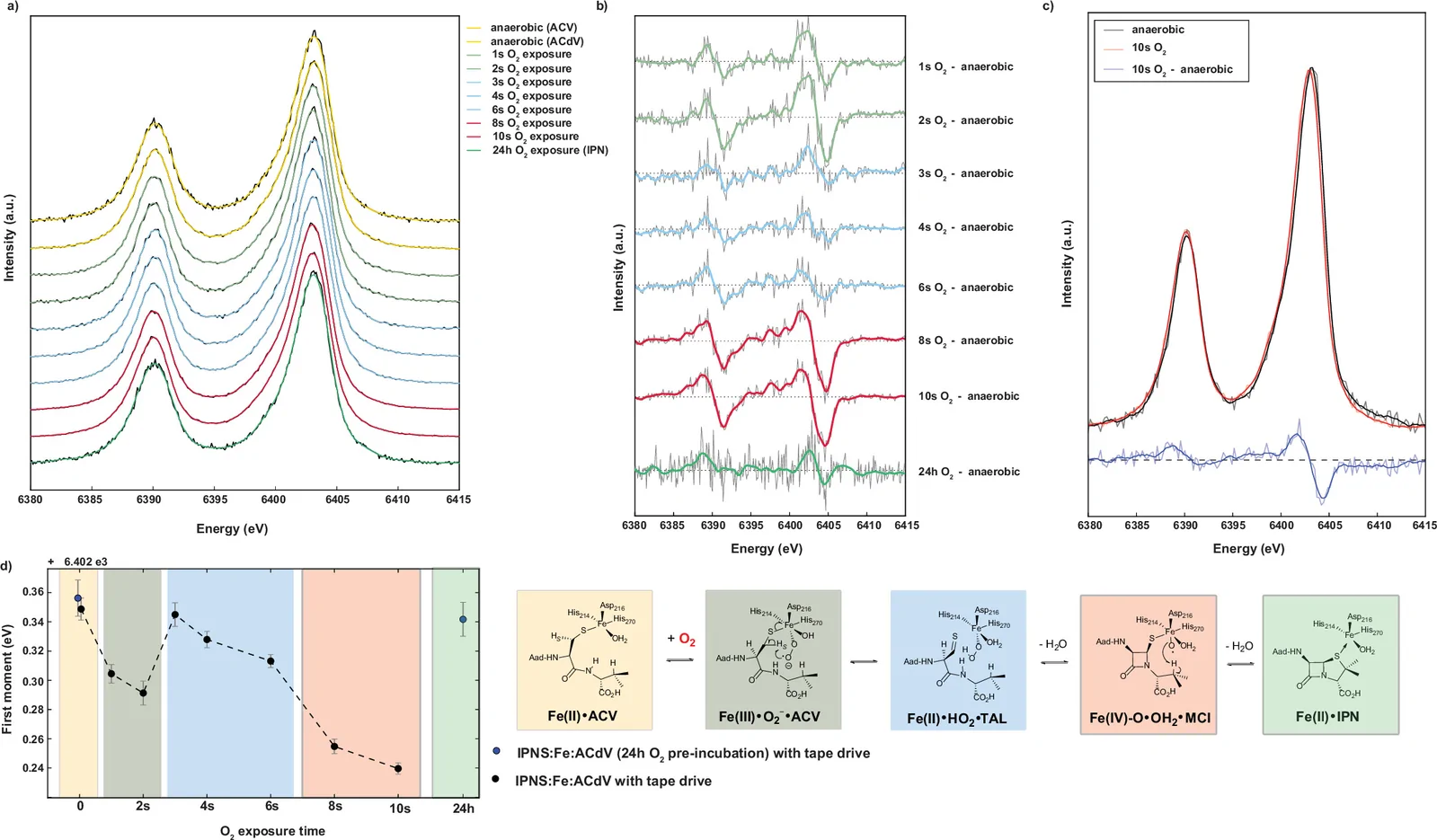
Time resolved Fe Kα XES during IPNS catalysis. (**a**) Fe Kα_1,2_ XES of anaerobic and O_2_ exposed IPNS:Fe:ACdV microcrystals collected at LCLS beam times LV56 and P-10035 using an ePix 100detector. Fe Kα_1,2_ XES data (LCLS beam time: LV56) of IPNS:Fe:ACdV anaerobic and after 1 s, 2 s, 3 s, 4 s, 6 s, 8 s and 10 s O_2_, and data (LCLS beam time: P-10035) of IPNS:Fe:ACV anaerobic and after 24 h O_2_ exposure (IPN from IPNS:Fe:ACdV).This data is complementary to tr-SFX data in Extended Data Fig. 1. All spectra were collected within a single experimental campaign using an unchanged spectrometer configuration; together with the randomized measurement order, this minimizes potential calibration drift. Area-normalized but otherwise unprocessed data are overlaid in gray to the smoothed data in color. (**b**) Difference spectra for 1 s, 2 s, 3 s, 4 s, 6 s, 8 s, 10 s O_2_ exposed data compared to anaerobic data from LCLS LV56 and difference spectra for 24 h O_2_ exposed data compared to anaerobic data from LCLS P-10035. Difference spectra calculated from the non-smoothed spectra are overlaid in gray. (**c**) Complementary Fe Kα_1,2_ XES of anaerobic and O_2_ exposed IPNS:Fe:ACdV microcrystals after 10 s O_2_ exposure obtained at PAL-XFEL beam time 2019-2nd-NCI-029 using the Jungfrau 0.5 M detector, with difference spectrum between O_2_ exposed and anaerobic data shown below. This data is complementary to the 10 s O_2_ tr-SFX data (PDB: 9I3D) in Extended Data Fig. 1. (**d**) Trend of first moment of the Fe Kα_1_ XES peak following O_2_ exposure (see coloured scheme). A moderate shift at 1 s and 2 s (±0.05 eV) indicates Fe oxidation supporting Fe(III) formation consistent with the tr-SFX Fe^3+^:O_2_^−^ data; smaller shifts at 3 s, 4 s and 6 s (±0.02 eV) suggest partial reduction consistent with formation of a Fe^2+^:O_2_H complex as observed by tr-SFX; a pronounced increase at 8 s and 10 s (>±0.10 eV) reflects substantial change in the electronic structure consistent with interpretation of the tr-SFX data as a Fe(IV) = O species followed by regeneration of Fe(II) in the IPN complex after 24 h O_2_, again consistent with the tr-SFX data. Error bars reflect statistical uncertainties of the spectral analysis and are derived from counting statistics (see Methods). Data are presented as nominal values calculated on the filtered spectra +/- standard deviation calculated using the unfiltered spectra on 98 and 150 data points of the spectra in the Kα_1_ region for the IPNS:Fe:ACdV + O_2_ (black, beam time: LV56) and IPNS:Fe:ACdV with O_2_ pre-incubation (blue, beam time: P-10035) datasets, respectively. Representative views of the Kα_1_ emission profile and peak maximum are shown in Supplementary Fig. 5.

**Extended Data Fig. 4:**
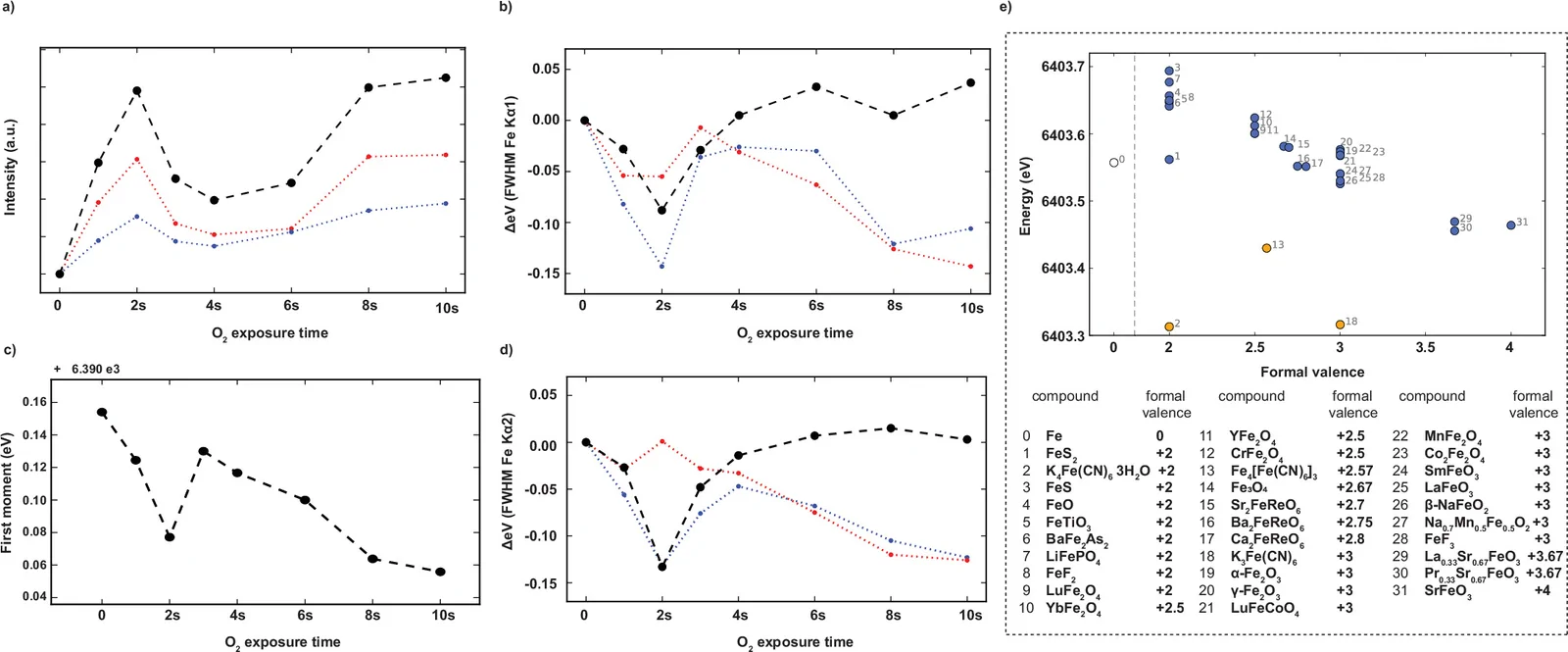
Quantitative analysis of Fe Kα XES spectral parameters. (**a**) Integrated absolute difference (IAD) analysis as a function of O_2_ exposure time. Three traces are shown corresponding to different integration ranges: full spectrum (Kα_1_ + Kα_2_, black), Kα_1_ region (red), and Kα_2_ region (blue). All three plots show similar trends in which the intermediate time points exhibit higher values. Notably, the IAD does not clearly distinguish intermediate time points (for example 2 s vs 8–10 s), highlighting its limited sensitivity to the type of spectral shift observed here. (**b**) Changes in full width at half maximum (FWHM) of the Fe Kα_1_ peak as a function of O_2_ exposure time (black). Changes in half-width at half maxiumum (HWHM) for higher energy half and lower energy half are plotted in blue and red, respectively. The FWHM shows only minor variation and does not capture the dominant spectral evolution. This reflects that the principal spectral change is not symmetric broadening but a shift of the emission profile, consistent with observations from the difference spectra. These phenomena are captured by HWHM: at early time point (2 s) changes in both HWHM are well-sparated, indicating asymmetric change in the band width. At later time points(4–10 s), a consorted shift in both HWHM indicate the spectral envelope is shifting in energy without substantial broadening or narrowing. (**c**) First moment analysis of the Fe Kα_2_ XES emission. The first moment shows a clear temporal evolution, with later time points (8-10 s) displaying larger shifts relative to early intermediates (1-2 s). This behaviour mirrors that observed for Kα_1_ (Extended Data Fig. 3d, Fig. 3b) and supports the presence of distinct electronic states at later stages of the reaction. (**d**) FWHM and HWHM analysis of the Fe Kα_2_ peak (black FWHM, blue: higher energy half, red: lower energy half). Similar to Kα_1_, the FWHM changes are small and do not reproduce the trend observed in the difference spectra or first moment analysis, reinforcing that peak width alone is not an adequate descriptor of the spectral changes in this system. (**e**) Correlation of Fe Kα_1_ first moment with formal valence using a set of reference Fe compounds^16^. The plot includes compounds with known formal oxidation states spanning Fe(0), Fe(II), Fe(III) to Fe(IV), demonstrating an overall trend of increasing first moment energy with increasing formal valence, with some minor exceptions for cyanide compounds (orange, compounds 2, 13, 18). The first moment values for the reference compounds were analysed independently using the same approach as applied to the IPNS datasets. The IPNS data points fall within this broader trend, supporting the use of relative first moment shifts to track changes in Fe electronic structure during catalysis, without requiring assignment of discrete oxidation states. Compound numbering corresponds to those listed in the list, restricted to species with available Kα data^16^.

Atomic models to fit positive difference electron density features apparent at the Fe-coordination site *trans* to Asp216 in the 1s and 2 s O_2_ timepoints, were refined as an ‘end-on’ bound O_2_ molecule; the ACV-valine isopropyl group was slightly rearranged compared with the anaerobic structure (Fig. 2 and Extended Data Figs. 1 and 2a,c,e), as observed for the initial IPNS:Fe(II):ACV reaction with O_2_ (ref. ^11^). The refined O_2_ occupancy increased from 50% after 1 s of O_2_ exposure, to near complete (>90%) after 2 s. Time-resolved XES (tr-XES) first moment, integrated absolute difference (IAD) and full-width at half-maximum (FWHM) analysis of the 1 s and 2 s timepoints revealed changes in the Fe K_α1,2_ XES compared with the anaerobic dataset, consistent with a higher level of the Fe(III)-superoxide intermediate after 2 s, compared to the 1 s timepoint (Fig. 3b and Extended Data Figs. 2c–g, 3 and 4). The isomorphous difference maps of the 2 s timepoint compared with unexposed crystals (Extended Data Fig. 2a(ii)) reveal small differences, suggesting partial movement of the ACV sulfur—such changes were more prominent at later timepoints.

### Transient release of sulfur during TAL formation

**Extended Data Fig. 5:**
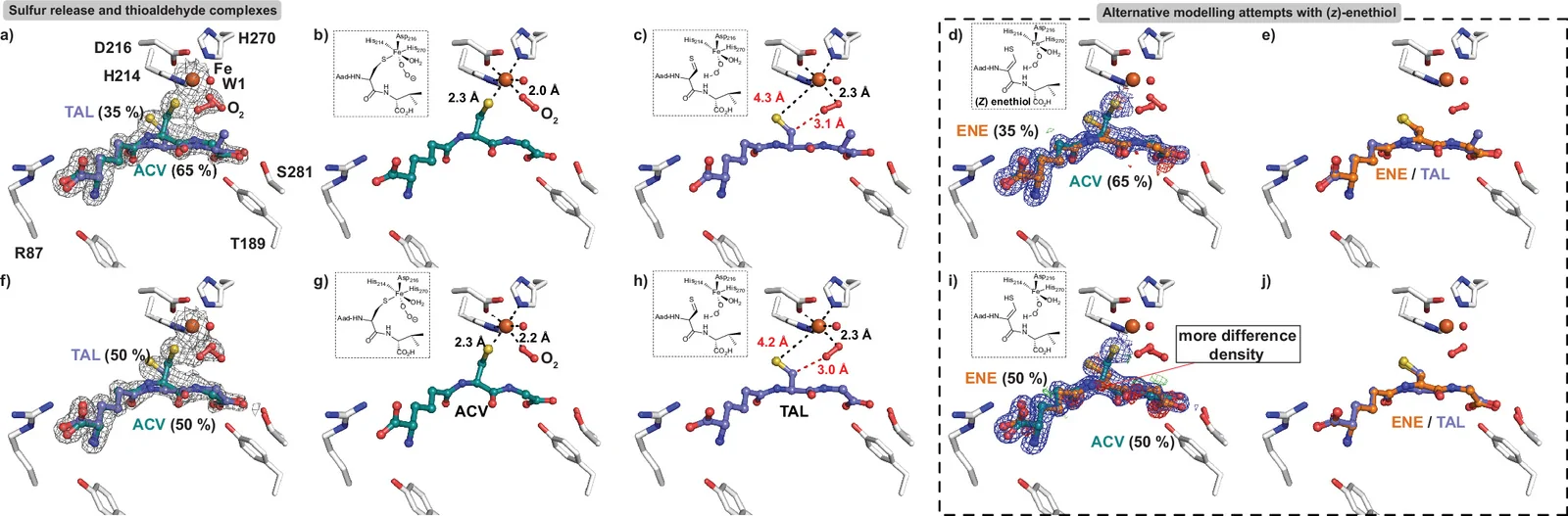
Structural analysis of TAL formation and evaluation of the enethiol model. (**a-c**) 2F_o_-F_c_ maps (1.0 σ) of IPNS:Fe:ACdV/TAL:O_2_ (PDB: 8PY9, 3 s O_2_) reveal two coexisting states: (1) the ACV–superoxo complex (~65%) and (2) the TAL /peroxo species (~35%) following C–H abstraction and Fe-S release. Note the reorientation of O_2_H for productive C–H abstraction at the cysteinyl CH_2_ group. Key distances are shown for the superoxo-ACV species in (b) and the peroxo–TAL species in (c). (**d**) 2mF_o_-DF_c_ maps (1.0 σ, blue) and mF_o_-DF_c_ maps (3.0 σ, green and −3.0 σ, red) from 3 s O_2_ data modeled with the (*Z*)-enethiol instead of the TAL suggest the presence of an enethiol cannot be fully excluded. (**e**) Superimposition of enethiol and TAL models in IPNS:Fe:ACdV/TAL:O_2_ (3 s O_2_, PDB: 8PY9) show only minor differences in orientation. (**f-h**) 2F_o_-F_c_ maps (1.0 σ) of IPNS:Fe:ACdV/TAL:O_2_ (PDB: 8PYA, 4 s O_2_) with two coexisting states of ACV–superoxo complex (~50%) and the TAL/peroxo species (~50%). Key distances are shown for the superoxo-ACV species in (g) and the peroxo–TAL species in (h). (**i**) 2mF_o_-DF_c_ maps (1.0 σ, blue) and mF_o_-DF_c_ maps (3.0 σ, green and −3.0 σ, red) from 4 s O_2_ data modeled with the (*Z*)-enethiol instead of the TAL suggest small difference features supporting a TAL. (**j**) Superimposition of the enethiol and TAL models in IPNS:Fe:ACdV/TAL:O_2_ (4 s O_2_, PDB: 8PYA) shows only minor differences.

The 3 s, 4 s and 6 s timepoint electron density maps exhibit features indicative of clear displacement of the ACV sulfur atom (ACV-S) from the Fe (Fig. 3 and Extended Data Figs. 1 and 5a,f), as supported by isomorphous difference maps (Fig. 3a and Extended Data Fig. 2a(iii)). The ACV-S:Fe distance in the refined atomic models increases from ~2.3 Å in the IPNS:Fe(II):ACdV structure to ~4.2 Å at the 3–6 s timepoints (Extended Data Fig. 5b,c,g,h). The maps are consistent with an *sp*^3^ ACV cysteinyl α-carbon, implying the presence of a non-Fe-coordinating TAL rather than an enethiol(ate) intermediate (Extended Data Fig. 5d,e,i,j). tr-XES data for the 3 s, 4 s and 6 s timepoints show evidence of reduction to Fe(II) (first moment analysis in Fig. 3b and Extended Data Fig. 3; IAD and FWHM analysis in Extended Data Fig. 4). These structures show mixed populations of Fe-linked unmodified ACV and an Fe-released TAL with occupancy increasing with time (ACV:TAL: 65:35 at 3 s, 50:50 at 4 s and 30:50 at 6 s). Notably, because the ACV valine isopropyl-side chain exhibits substantial conformational mobility, as manifested by weak/absent electron density, it was omitted from the final refined atomic models at these timepoints. Comparison of the electron density across the 3 s, 4 s and 6 s timepoints provides strong evidence for movement of the O_2_-derived atoms. We observe that O_2_ initially binds to the IPNS:Fe(II):ACdV complex in an end-on manner, but in the 3 s, 4 s and 6 s timepoints the two oxygen derived atoms are reoriented into a quasi side-on binding mode with the distal oxygen approaching the ACV cysteinyl *pro* 3–H bond, suggestive of a productive geometry for stereospecific hydrogen abstraction, as occurs in solution^2^ and as supported by computational studies (see below).

### MCI formation during IPNS catalysis

**Extended Data Fig. 6:**
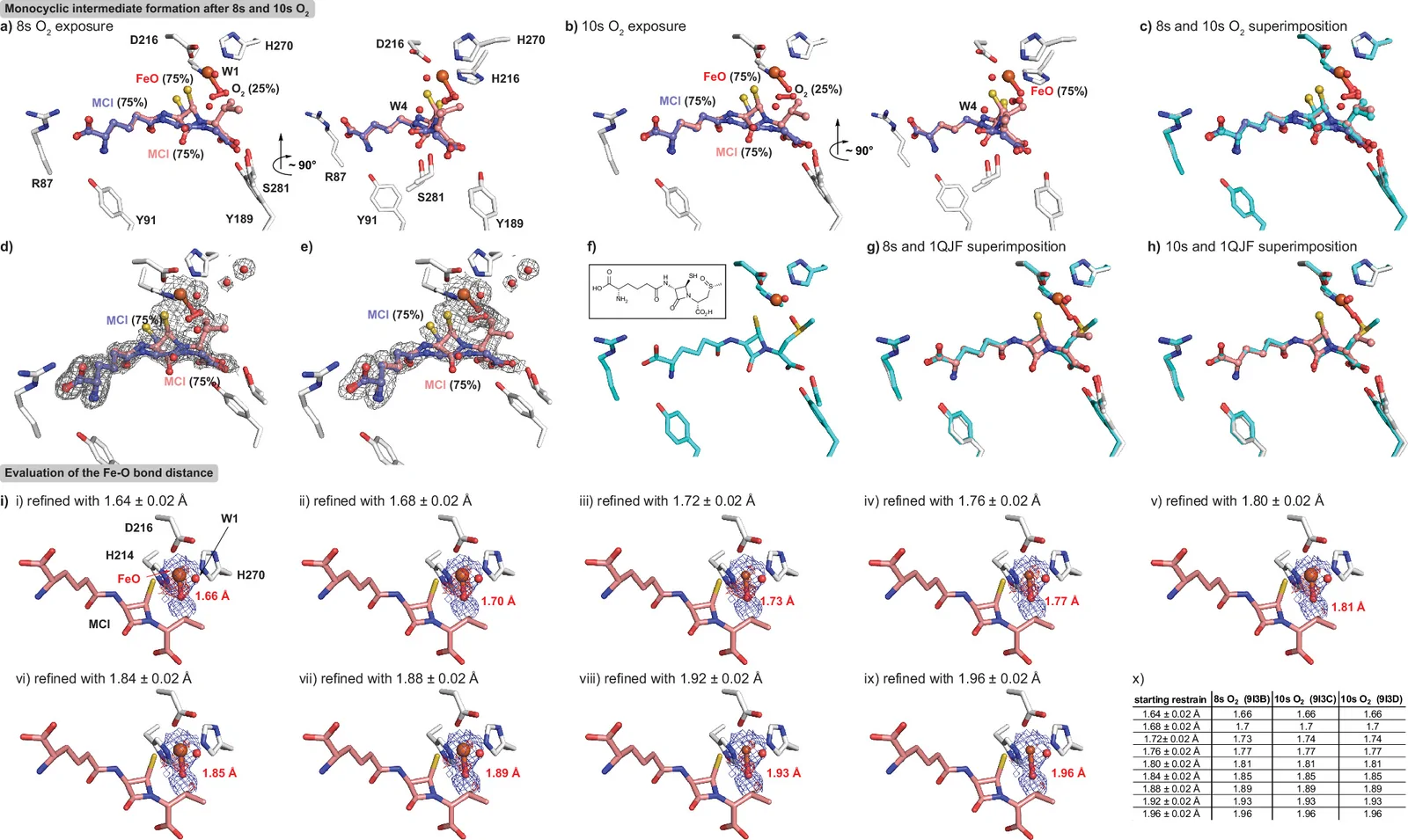
Structural analysis of the monocyclic intermediate and Fe=O bond refinement. Active site views of (**a**) IPNS:Fe/Fe=O:TAL/MCI (PDB: 9I3B, 1.60 Å resolution, 8 s O_2_) and (**b**) IPNS:Fe/Fe=O:TAL/MCI (PDB: 9I3C, 1.70 Å resolution, 10 s O_2_) highlighting the presence of the two main states after 8 s and 10 s O_2_ with the IPNS:Fe=O:MCI (75% occupancy) and the IPNS:Fe:O_2_H:TAL (25% occupancy). (**c**) Superimposition of IPNS:Fe/Fe=O:TAL/MCI (PDB: 9I3B, 8 s O_2_, white) and IPNS:Fe/Fe=O:TAL/MCI (PDB: 9I3C, 10 s O_2_, cyan) structures showing near identical active site orientation. 2F_o_-F_c_ maps (1.0 σ contour level, carved around TAL/MCI/Fe=O/O_2_) of (**d**) IPNS:Fe/Fe=O:TAL/MCI (PDB: 9I3B, 1.60 Å resolution, 8 s O_2_) and (**e**) IPNS:Fe/Fe=O:TAL/MCI (PDB: 9I3C, 1.70 Å resolution, 10 s O_2_). Note: alternative modelling attempts were made by modelling an Fe-bound uncyclized TAL species instead of MCI by loosening the bond angle and distance restraints in the corresponding CIF file. Although the electron density in these models was of sufficient quality, the resulting geometry of the uncyclized TAL ligand exhibited unrealistic bond angles and was inconsistent with the stereochemical constraints expected for MCI formation. Therefore we modelled our 8 s and 10 s O_2_ data sets with the MCI. (**f**) Active site view of an analogous MCI complex (PDB: 1QJF)^8^ obtained after O_2_ exposure of crystals with *l*-δ-(α-aminoadipoyl)-*l*-cysteinyl-*d*-(S-methyl)-cysteine. Superimposition with our tr-SFX MCI structures after (**g**) 8 s and (**h**) 10 s O_2_ exposure revealing nearly identical orientation. (**i**) Representative 2mFo–DFc (1.0σ, blue) and mFo–DFc (+3.0σ green, –3.0σ red) maps of IPNS:Fe/Fe=O:TAL/MCI (PDB: 9I3B, 1.60 Å resolution, 8 s O_2_) using varying Fe=O bond restraints from (i) 1.64 ± 0.02 Å to (iv) 1.96 ± 0.02 Å. Reduced difference features are observed at the longer Fe-O bond distance of 1.96 ± 0.02 Å. Note that the observed Fe=O bond length (in red) exceeds the applied restraint in the shorter distances, supporting a ‘correct’ bond length of 1.96 ± 0.02 Å. (x) Summary of observed Fe=O bond lengths from refinements using restrained distances in IPNS:Fe/Fe=O:TAL/MCI (PDB: 9I3B, 1.60 Å resolution, 8 s O_2_), IPNS:Fe/Fe=O:TAL/MCI (PDB: 9I3C, 1.70 Å resolution, 10 s O_2_) and IPNS:Fe/Fe=O: MCI/IPN (PDB: 9I3D, 1.68 Å resolution, 10 s O_2_).

The isomorphous difference maps for the 8 s and 10 s timepoints (for example, 8 s versus 3 s) (Fig. 3a(ii)) clearly reveal restoration of Fe–S coordination (~2.4 Å). We modelled an MCI at 75% occupancy for both the 8 s and 10 s O_2_ datasets, with residual electron density indicative of a partial occupancy TAL (Fig. 2 and Extended Data Figs. 1 and 6a–e). Comparison of our MCI structure with a structure of IPNS complexed with a substrate analogue, l-δ-(α-aminoadipoyl)-l-cysteinyl-d-(S-methyl)-cysteine (PDB: 1QJF), which following O_2_ exposure gives a stable MCI complex^8^, reveals that the two structures closely correlate (Extended Data Fig. 6f–h), supporting the assignment of the ACV-derived MCI as observed by tr-SFX.

We observed a substantial decrease in difference density features at the 8 s and 10 s timepoints compared with the 3 s, 4 s and 6 s timepoints, possibly reflecting increased flexibility of the non-Fe–S coordinating TAL compared to the Fe-coordinating MCI (Extended Data Fig. 2a(iv)). Consistent with this, well-defined electron density was observed for the previously disordered ACV valine isopropyl group, suggesting increased conformational rigidity owing to the formation of an Fe-linked MCI (Extended Data Fig. 6d,e). Importantly, at the MCI stage the valine 3–H projects towards the Fe, in a manner appropriate for productive abstraction to enable thiazolidine ring formation, contrasting with the anaerobic structure, where the valine 3–H projects away from the Fe (Fig. 2).

**Extended Data Fig. 7:**
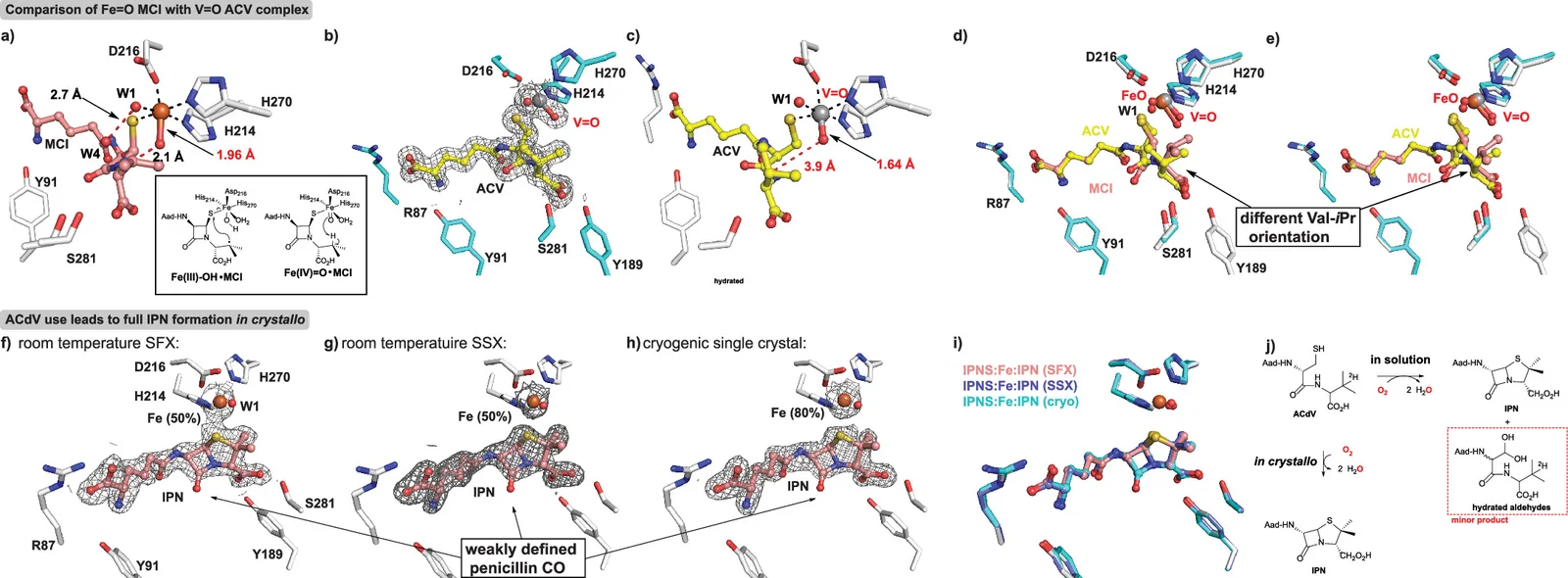
Comparison of the Fe=O intermediate with the V=O mimic and structural analysis of IPN formation. (**a**) Active site view and key distances of IPNS:Fe/Fe=O:TAL/MCI (PDB: 9I3B, 1.60 Å resolution, 8 s O_2_) revealing the refined Fe-O bond length of 1.96 Å, with the oxygen apparently positioned for productive Val-CH cleavage, the presence of W4 (likely due to homolytic peroxide cleavage), which is positioned to hydrogen bond with W1 (2.7–2.8 Å). The inset summarises potential intermediates reflected by this state: (1) the IPNS:Fe(IV) = O: MCI or the IPNS:Fe(III)-OH: MCI-radical post CH abstraction. (**b**) 2F_o_-F_c_ maps (1.0σ, grey) of IPNS:VO:ACV (Fe(IV) = O mimic, PDB: 6ZAK, 1.54 Å resolution) revealing V(IV) = O binding with an anticipated bond distance of 1.64 ± 0.02 Å^29^, excluding bond distance elongation due to photoelectric effects caused by tr-SFX measurement. (**c**) Active site view with key distances of IPNS:VO:ACV (PDB: 6ZAK, 1.54 Å resolution). (**d** + **e**) Superimposition of IPNS:VO:ACV (PDB: 6ZAK, cyan with (**d**) IPNS:Fe/Fe=O:TAL/MCI (PDB: 9I3B, 8 s O_2_) and (**e**) IPNS:Fe/Fe=O:TAL/MCI (PDB: 9I3C, 10 s O_2_) revealing a slightly altered orientation of Fe(IV) = O vs V(IV) = O and an altered Val-iPr orientation positioning the ACV Val-CH in a productive conformation for CH abstraction. (**f-i**) Active site view and 2F_o_-F_c_ maps (1.0σ, grey) of (**f**) IPNS:Fe:IPN (PDB: 9QPH, 1.70 Å resolution, >24 h O_2_, SFX LCLS), (**g**) IPNS:Fe:IPN (PDB: 9QPJ, 1.90 Å resolution, 24 h O_2_, SSX DLS), (**h**) IPNS:Fe:IPN (PDB: 9QPG, 1.79 Å resolution, 18 h O_2_, cryo DLS) and (**i**) superimposition confirming complete IPN formation. Note that the DLS datasets (g, h) and the LCLS SFX dataset (f) originate from the same crystal preparation following full turnover and were collected under complementary experimental conditions; structural comparison confirms no substantial differences in the overall fold or product binding mode. B-factor analyses discussed in the main text were performed exclusively on SFX datasets refined under identical protocols, and XES measurements were obtained only from SFX experiments.(**j**) Note with ACdV and IPNS in solution, the formation of hydrated aldehyde diasteromers (**1**, ~5%) is observed (in addition to IPN) due to operation of an isotope effect^33^. Note that the Fe occupancy drops to 50%-80% (**f-h**) in the IPN complex, possibly due to potential oxidation to Fe(III) and release into solution. Note that as observed in previous IPNS structures^8,11^, the carbonyl group of the β-lactam ring in the MCI and in IPN shows weak and sometimes poorly defined electron density.

For the 8 s and 10 s timepoints, we focused on modelling the site *trans* to Asp216, where the Fe=O group is proposed to form then mediate cleavage of the valine Cβ–^2^H bond in ACdV. Initial refinements using a restrained Fe–O bond length of 1.64 Å (Extended Data Fig. 6i), consistent with the predicted Fe(IV)=O species^23^, produced difference features in the electron density maps, potentially indicating a longer Fe–O bond. Consequently, we refined models with varying Fe–O bond distances and obtained a bond length of 1.96 ± 0.04 Å for both the 8 s and 10 s O_2_-exposed structures, somewhat longer than anticipated for an Fe=O species^24^. The interpretation of this distance is complicated by the presence of a residual TAL associated peroxide, hence, cautious analysis is required. It is possible that at least some ^2^H atom abstraction at the Cβ valine carbon has occurred with concomitant formation of an Fe(III)–OH species in crystallo. In any event, mixtures of these reaction intermediates are difficult to resolve structurally even with high-resolution (1.5 Å) SFX data (Extended Data Fig. 7a).

To investigate our modelling of the Fe(IV)=O species and rule out potential, albeit unlikely, X-ray induced artefacts using XFEL sources, we collected a dataset of IPNS complexed with ACV and a catalytically inactive vanadyl ion (V(IV)=O) (PDB: 6ZAK) (Fig. 2 and Extended Data Fig. 7b,c). This approach has been used previously to mimic high-valent metal-oxo geometry within Fe-dependant oxygenase catalysis^25–27^. The IPNS:V(IV)=O:ACV complex structure closely resembled that of the wild-type Fe:ACV complex, both in terms of overall substrate orientation and active-site geometry. Importantly, the Fe-coordinating water W1 was conserved and the V(IV)=O oxygen occupies the site *trans* to Asp216, consistent with the expected position of the Fe(IV)=O oxygen. Compared with the putative Fe(IV)=O intermediate in the 8 s/10 s datasets, the V(IV)=O bond vector displayed a slightly different orientation (Fig. 3a(iv) and Extended Data Fig. 7d,e), an observation which may reflect subtle differences in geometry or ligand-field effects^28^. The observed V–O bond distance of 1.64 ± 0.02 Å is consistent with literature reports^29^, implying that the observed differences in V(IV)=O and Fe(IV)=O bond length are probably not due to radiation damage artefacts, as supported by reported studies^30–32^.

tr-XES first moment analysis of the 8 s/10 s O_2_-exposed samples is consistent with a species more oxidized than Fe(III), indicating at least the partial presence of the Fe(IV) intermediate (Fig. 3b and Extended Data Fig. 3). In addition to the first moment analysis, we evaluated the IAD and FWHM parameters (Extended Data Fig. 4a–d). The IAD shows broadly similar trends over all timepoints, though the FWHM exhibits only minor variation at the 8 s and 10 s timepoints. The limited sensitivity of the FWHM probably reflects the non-uniform and partially asymmetric nature of the spectral changes, which are dominated by a shift of the emission envelope rather than symmetric peak broadening (Extended Data Fig. 4 and Supplementary Fig. 5). The randomized data collection order and unchanged spectrometer configuration indicate no detectable calibration drift over the course of the experiment.

Overall, the combined evidence supports reaction towards an MCI with the high-valent Fe(IV)-state critical for ACV valine C3-H abstraction. Notably, both the 8 s and 10 s O_2_ structures exhibited a new positive electron density feature near the MCI isopropyl group, assigned to a water molecule (W4) (Extended Data Fig. 6a,b), which may originate from homolytic cleavage of the Fe-peroxide resulting in the Fe(IV)=O species.

### Penicillin ring formation with ACdV

We also obtained a 10 s timepoint at the PAL-XFEL using tr-SFX with the tape drive. The resultant data revealed a clear difference density at the position of the sulfur atom (Fig. 3a(iii)) indicating its re-localization adjacent to, but not coordinating, the Fe. The 10 s PAL-XFEL structure (PDB: 9I3D, SFX) showed evidence for partial IPN formation together with the MCI. Prolonged (~24 h) O_2_ exposure of the IPNS:Fe(II):ACdV complex led to a structure with apparent full occupancy of the IPNS:Fe:IPN complex (PDB: 9QPH, LCLS, SFX) (Fig. 2 and Extended Data Fig. 7f–j). The geometry of the IPN product is clearly tetrahedral, as observed for crystalline penicillins^1^. There was no evidence for any other products, contrasting with solution studies in which ~5% of a shunt product (hydrated aldehyde **1**) was observed when using ACdV, although the formation of low levels of **1** in crystallo may be below observable limits^33^. As reported with unlabelled ACV, extended O_2_ exposure of the IPNS:Fe(II):ACdV complex to O_2_ resulted in decreased Fe occupancy (to ~50% after 24 h) with the IPN product remaining bound^11^ (Extended Data Fig. 7f–h). Importantly, the diffraction quality and overall lattice order were maintained after prolonged O_2_ exposure, consistent with previous observations^11^, indicating that crystal integrity is preserved over this timescale. tr-XES results implied most of the iron in the sample had returned to the Fe(II) state in the IPN complex (Fig. 3b and Extended Data Fig. 4), albeit slightly more oxidized than in the anaerobic Fe(II) starting state, potentially reflecting partial Fe(II) dissociation and oxidation to Fe(III) in solution.

### B-factor analysis reveals protein and water dynamics

With time-resolved structural snapshots capturing the catalytic cycle of IPNS in hand, we investigated potentially dynamic regions during catalysis via B-factor analysis of all our SFX structures (Fig. 2(iii),(iv) and Extended Data Fig. 1). Each dataset was initially refined against the same starting model (IPNS:Fe:ACV) using molecular replacement, followed by two refinement rounds with ligand and Fe occupancies fixed at 1.0 and automatic water placement enabled. The B-factors for the backbone atoms (N, Cα, C and O, representing the amide-nitrogen, alpha-carbon, carbonyl-carbon and carbonyl-oxygen, respectively) were extracted using PyMOL and normalized via *Z*-score transformation to allow cross-dataset comparison. In a second analysis, we included all ligands/water molecules to investigate solvent and side chain atoms.

B-factor analysis of the backbone atoms revealed higher thermal factors indicative of increased mobility of the α3 and α10 helices, consistent with previous observations^11,15^. B-factors for helix α3 increased progressively to 10 s, then returned to starting levels in the IPN complex (Fig. 2(iii) and Extended Data Fig. 1). Two distinct α3 conformations were modelled in the 6 s, 8 s and 10 s O_2_ datasets, suggesting conformational heterogeneity; all other atomic models refined with a single α3 conformation. By contrast, α10 exhibited elevated B factors in all the O_2_-exposed structures, consistent with the observation that dynamic movement is required to release the IPN product, but lattice packing interactions prevent this (Fig. 2(iii), Extended Data Fig. 1 and Supplementary Fig. 4). These findings support the involvement of remote helical rearrangements in the global protein during catalysis.

B-factor analysis of the ligands and water molecules showed that the Fe-coordinated water W1, as well as two additional waters (W2 and W3), which with W1 form a hydrogen-bonded chain, displayed increased mobility of W2 in the 8 s and 10 s O_2_-exposed datasets (Fig. 2(iv) and Extended Data Fig. 1). This behaviour suggests a possible role for solvent rearrangement in catalysis, evidence for which was provided by subsequent mutagenesis studies.

### Second shell mutations support water-mediated catalysis

**Fig. 4:**
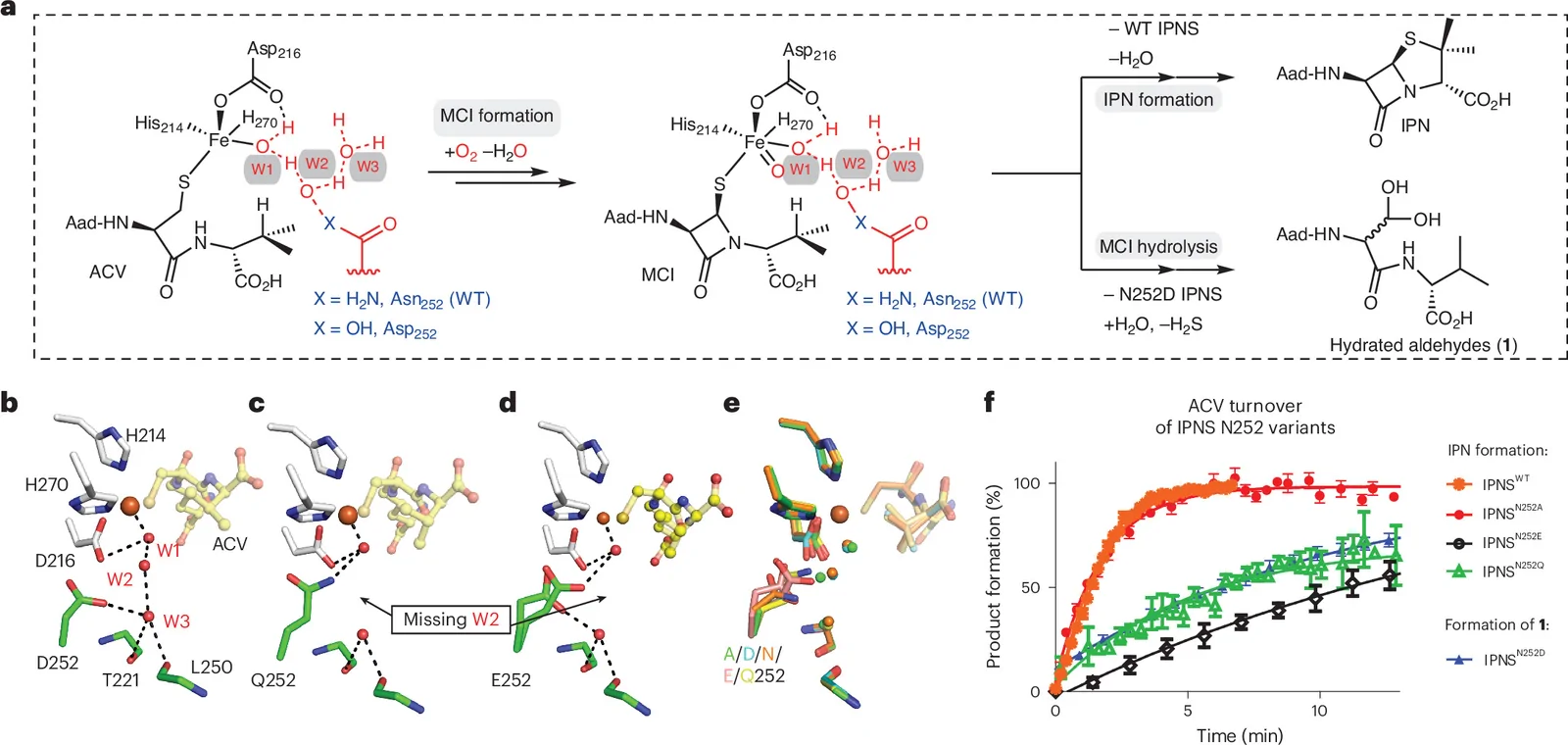
Importance of the Fe-bound water chain in IPNS catalysis. **a**, Different outcomes of IPNS^WT^ and IPNS^N252D^ catalysis showing the impact of the negatively charged D252 side chain on the role of W2 at the MCI stage, resulting in the release of the shunt product **1**. **b**–**d**, Active-site views of the Fe-linked H_2_O chain in IPNS^N252D^:Fe:ACV (PDB: 8P48, 1.63-Å resolution, anaerobic, DLS, cryo single crystal) (**b**), IPNS^N252Q^:Fe:ACV (PDB: 8PTV, 1.60-Å resolution, anaerobic, DLS, cryo single crystal) (**c**) and IPNS^N252E^:Fe:ACV (PDB: 8RDB, 1.37-Å resolution, anaerobic, DLS, cryo single crystal) (**d**). Note the intact H_2_O chain in IPNS^N252D^ and interrupted H_2_O chain in IPNS^N252Q^ and IPNS^N252E^. **e**, Superimposition of the N252 variants (Extended Data Fig. 8). **f**, Turnover by IPNS^N252X^ variants (2 μM), ACV (400 μM), (NH_4_)_2_Fe(SO_4_)_2_ (100 μM), TCEP (800 μM) and l-ascorbate (800 μM) in 25 mM Tris, 100 mM NaCl pH 8.0 under ambient O_2_ levels using solid-phase extraction mass spectrometry (s.e.m., *n* = 3 independent replicates)^15^. Note, IPNS^WT^, IPNS^N252A^, IPNS^N252Q^ and IPNS^N252E^ produced IPN; IPNS^N252D^ does not produce IPN, instead giving hydrated aldehyde **1**. For additional data see Supplementary Fig. 6.

**Extended Data Fig. 8:**
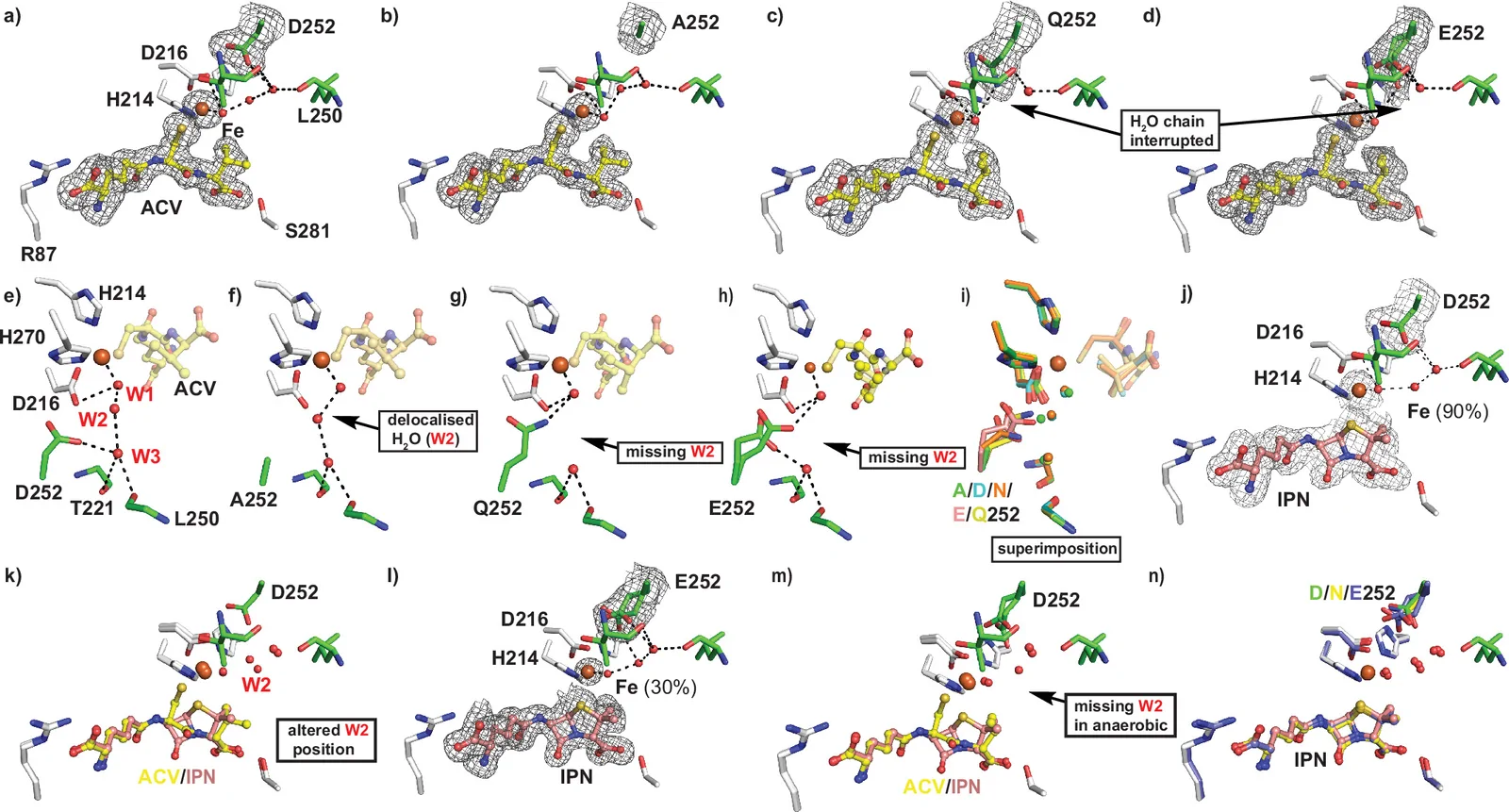
Crystallographic and spectroscopic analysis of IPNS^N252X^ studying the impact of X252 variants on the Fe-bound H_2_O chain. Polder omit map (3.0 σ contour level, carved around ACV, Fe and X252 residue) of the active site of: (**a**) IPNS^N252D^:Fe:ACV (PDB: 8P48, 1.63 Å resolution), (**b**) IPNS^N252A^:Fe:ACV (PDB: 8P47, 1.56 Å resolution), (**c**) IPNS^N252Q^:Fe:ACV (PDB: 8PTV, 1.60 Å resolution) and (**d**) IPNS^N252E^:Fe:ACV (PDB: 8RDB, 1.37 Å resolution) highlighting interaction of X252 with the H_2_O chain. N252A and N252D have no substantial impact on the position of W2 compared to IPNS^WT^ whereas N252Q and N252E displace W2. Structures shown were determined by cryogenic synchrotron crystallography (MX) at DLS. (**e-i**) Key hydrogen bonding interactions of X252 with the H_2_O chain for (**e**) IPNS^N252D^, (**f**) IPNS^N252A^, (**g**) IPNS^N252Q^ and (**h**) IPNS^N252E^, with (**i**) showing the superimposition of the variants with IPNS^WT^. (**j**) and (**l**) Polder omit map (3.0 σ contour level, carved around IPN, Fe and X252 residue) of the active site of (**j**) IPNS^N252D^:Fe:IPN (PDB: 8P46, 1.77 Å resolution, 30 min O_2_ exposure) and (**l**) IPNS^N252E^:Fe:IPN (PDB: 9HWF, 1.40 Å resolution, 30 min O_2_ exposure) revealing IPN formation *in crystallo*. Note that the Fe is present in reduced occupancy (IPNS^N252D^:Fe:IPN (90%) and IPNS^N252E^:Fe:IPN (30%)) and that W2 is partially present in the product complex of N252E compared to N252E anaerobic. (**k**) and (**m**) Superimposition of (**k**) IPNS^N252D^:Fe:ACV (PDB: 8P48) with IPNS^N252D^:Fe:IPN (PDB: 8P46) and (**m**) IPNS^N252E^:Fe:ACV (PDB: 8RDB) and IPNS^N252E^:Fe:IPN (PDB: 9HWF) structures revealing small differences in the location of the Fe-bound H_2_O chain with W2 missing in the IPNS^N252E^:Fe:ACV vs IPNS^N252E^:Fe:IPN structures. (**n**) Superimposition of IPNS^N252D^:Fe:IPN (PDB: 8P46), IPNS^N252E^:Fe:ACV (PDB: 8RDB) and IPNS:Fe:IPN (PDB: 6ZAQ)^11^ revealing near identical IPN positions with small changes in the H_2_O chain due to the identity of residue X252. Additional biochemical characterisation of the IPNS^N252D^ variant is shown in Supplementary Fig. 6.

A three molecule water chain (Fe–W1–W2–W3) extending away from the active site is stabilized by interactions with the backbone carbonyl groups of T221 and L250, and the side chain of N252, which is positioned to hydrogen bond with W2 (Fig. 4a and Extended Data Fig. 8). As backbone carbonyls are difficult to modify, we explored how the steric and electrostatic properties of residue 252 influence catalysis. We generated four N252 variants (N252A, N252Q, N252E and N252D) and determined anaerobic structures at Diamond Light Source (DLS) using traditional, rotation-based single crystal synchrotron diffraction under cryogenic conditions (MX), rather then tr-SFX, for each variant complexed with Fe(II) and ACV: IPNS^N252A^:Fe:ACV (PDB: 8P47), IPNS^N252Q^:Fe:ACV (PDB: 8PTV), IPNS^N252E^:Fe:ACV (PDB: 8RDB) and IPNS^N252D^:Fe:ACV (PDB: 8P48). In the smaller N252A variant, W2 was slightly displaced, although the three-water chain and hydrogen bonding remained intact. By contrast, both the N252Q and N252E variants, which each have an additional methylene group compared with N252, introduced steric hindrance that ablated W2 binding. The isosteric N252D variant was structurally very similar to wild-type (WT)-IPNS and showed no substantial structural differences in water positioning, including with respect to maintenance of W2 (Fig. 4b–e and Extended Data Fig. 8a–i). Prolonged O_2_ exposure of single crystals of the N252D and N252E variants resulted in IPNS^N252E^:Fe:IPN (PDB: 9HWF, MX) and IPNS^N252D^:Fe:IPN (PDB: 8P46, MX) product complexes (Extended Data Fig. 8j-n).

Turnover assays in solution employing solid-phase extraction mass spectrometry showed N252A, N252Q and N252E produced IPN as the sole-observed product, though the latter two mutants manifested substantially impaired catalytic efficiency (Fig. 4f and Supplementary Fig. 6). By contrast, both with the other N252 mutants and the crystallographically observed IPN formation, in solution, N252D exhibited a marked reduction in catalytic efficiency and did not produce any observed IPN. Instead N252D catalysed production of a hydrated formyl-glycine product (**1**) as the sole characterized product (Fig. 4f and Supplementary Fig. 6). Previous studies with the ACV analogue, in which valine is susbstituted for glycine, and hence is incapable of thiazolidine formation, have shown formation of an analogous aldehyde^34^. Further, as reported^33^ and confirmed by us, the synthetic MCI thiol (derived from the Hg(II) salt) undergoes hydrolysis to give **1** (along with other products, see below).

Given the high structural conservation of the N252 variants and the fact that all produce IPN in crystallo, it is remarkable that the introduction of a negatively charged side chain at residue 252 produces the MCI derived shunt product **1** in solution^33^. Together with the tr-SFX studies showing evidence for mobility of W2 in the 8 s and 10 s datasets (Fig. 2), these results support a critical role for the water chain in stabilizing intermediates, in particular the formation and reaction of the MCI during catalysis before IPN formation.

### H270E reveals Fe-bound water involvement in peroxide cleavage

**Fig. 5:**
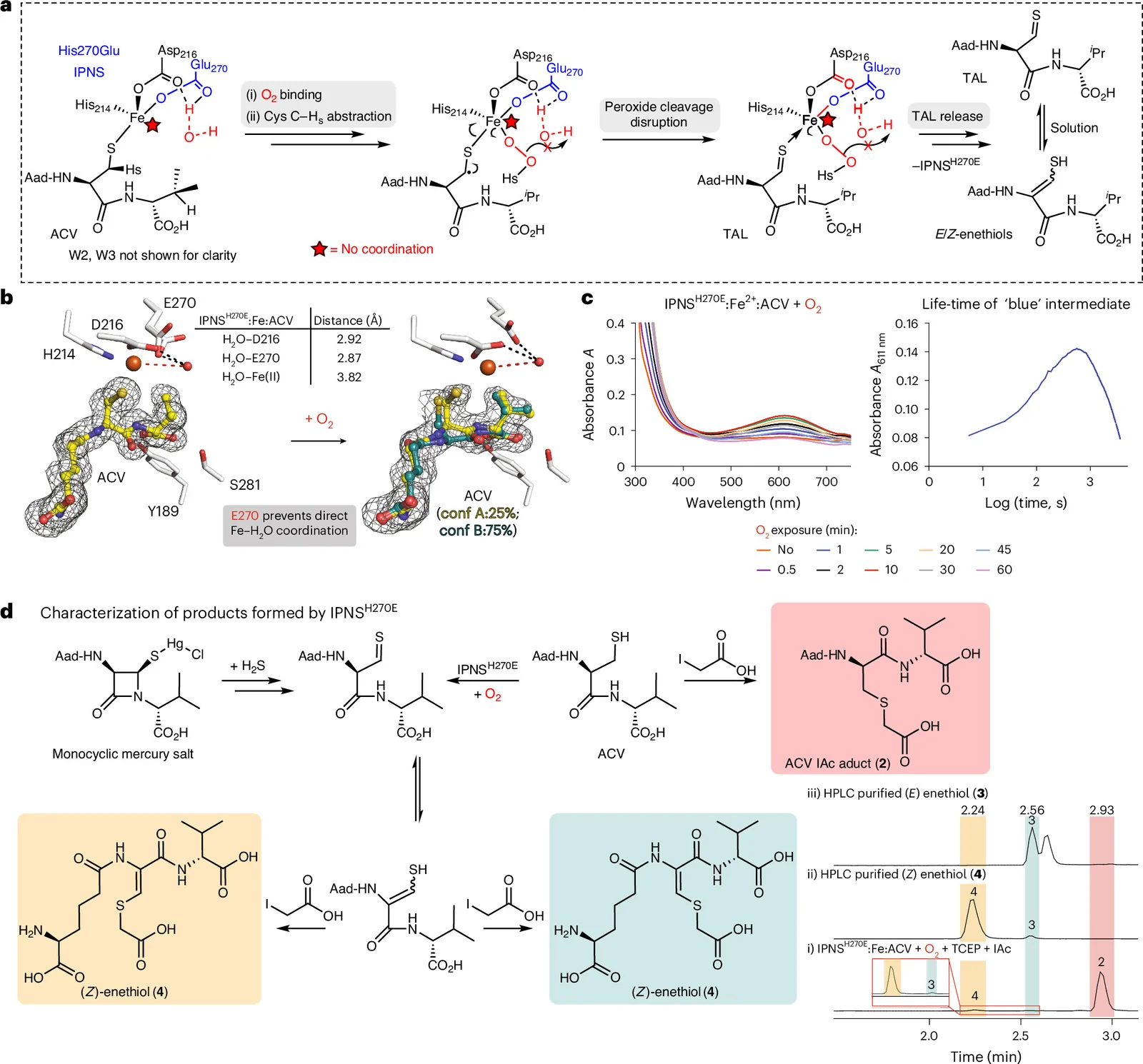
The H270E variant supports a role for the Fe-bound H_2_O in peroxide cleavage and TAL formation. **a**, Different outcomes of IPNS^WT^ and IPNS^H270E^ catalysis reflecting involvement of Fe-bound W1 in IPNS peroxide cleavage (Fig. 1, path b) implying TAL release in the absence of Fe-bound W1 for IPNS^H270E^. **b**, Active-site view of IPNS^H270E^:Fe:ACV (PDB: 7ZOE, 1.50-Å resolution, anaerobic, DLS, cryo single crystal) and IPNS^H270E^:Fe:ACV (PDB: 8ALJ, 1.68-Å resolution, 3 h O_2_ exposure, DLS, cryo single crystal), revealing that E270 prevents direct Fe–W1 coordination (Supplementary Fig. 7). Note, in the second ACV conformation observed in IPNS^H270E^:Fe:ACV after 3 h O_2_ exposure, the ACV S is positioned away from Fe (~3.8 Å to ~4.1 Å), as similarly observed in the 3–6 s O_2_ tr-SFX timepoints (Fig. 2). For additional data see Supplementary Fig. 7. **c**, UV–visible spectra (300–750 nm) of IPNS^H270E^ (150 μM), Fe^2+^ (1 equiv., 150 μM) and ACV (5 equiv., 750 μM) + O_2_ in MOPS buffer (100 mM in H_2_O, pH 7.2) revealing a 611-nm absorption peak. Control experiments are shown in Extended Data Fig. 7. **d**, Left: IPNS^H270E^ produces *E*/*Z* enethiols probably as a consequence of nascent TAL production followed by tautomerization, as shown by comparison with synthetic standards. Right: LC–MS spectra for alkylated derivatives for (i) IPNS^H270E^ (5 μM), FeSO_4_ (20 μM in H_2_O), ACV (100 μM), sodium ascorbate (100 μM), TCEP (100 μM) and iodoacetic acid (250 μM) in Tris buffer (25 mM in H_2_O, pH 8.0); (ii) the HPLC purified (*Z*) enethiol (**4**); and (iii) the HPLC purified (*E*) enethiol (**3**). High-resolution MS spectra are shown in Supplementary Fig. 8. For controls see Supplementary Fig. 8.

Further evidence for the catalytic importance of the water chain comes from mutagenesis studies affecting the Fe-coordinating W1. IPNS^H270E^, in which the Fe-coordinating His270 is substituted with a glutamate, gave particularly interesting results with respect to the TAL intermediate. Although the overall fold of IPNS^H270E^ is conserved, a crystal structure of the Fe(II):ACV complex (PDB: 7ZOE, MX) reveals that the iron exhibits four-coordinate geometry, involving His214, Asp216, Glu270 and the ACV thiolate. Compared with the WT IPNS structures, W1 is apparently ‘displaced’ from the Fe(II), that is, the Fe(II)–W1 distance increases from ~2.1 Å in WT IPNS to ~3.8 Å in the H270E variant. The refined atomic model for IPNS^H270E^:Fe(II):ACV shows that W1 is stabilized by a hydrogen-bonding network involving side chain oxygens of Asp216 and Glu270, with the latter observed in two conformations (Fig. 5a,b and Supplementary Fig. 7a). Non-denaturing mass spectrometry confirmed formation of the IPNS^H270E^:Fe:ACV complex in solution (Supplementary Fig. 8c).

Upon O_2_ addition to IPNS^H270E^:Fe:ACV in solution, a transient blue colour (*λ*_max_ 611 nm, t_1/2_ several minutes) was observed (Fig. 5c and Supplementary Fig. 8a,b), implying formation of a short-lived intermediate^35^. HPLC–MS and NMR analyses of the reaction under both reductive (+TCEP) and non-reductive (no TCEP) conditions confirmed formation of ACV-derived oligomers, without detectable IPN production (Supplementary Fig. 8).

As TALs are known to undergo efficient tautomerization and oligomerization^36,37^, we incubated IPNS^H270E^ with Fe(II)/ACV then treated the mixture with 2-iodoacetic acid. Two new products, both with a mass (420.1446 Da) 2 Da less than of the *S*-alkylated ACV (**2**, 422.1597 Da) were observed (Supplementary Fig. 8d). To investigate these, we synthesized a monocyclic mercury salt^33^ (synthesis shown in Supplementary Fig. 9), which, after treatment with H_2_S then iodoacetic acid, yielded the *E-* and *Z-*enethiol carboxymethyl thioether derivatives (Fig. 5d and Supplementary Figs. 8d, 10 and 11). Comparison with synthetic standards identified the new products as the *S*-alkylated enethiol *E-* and *Z-*diastereomers (**3** and **4**) (Fig. 5d and Supplementary Fig. 8d).

Exposure of IPNS^H270E^:Fe:ACV single crystals to O_2_ for 30 min (PDB: 8ALI, MX) and 3 h (PDB: 8ALJ, MX) resulted in displacement of the ACV-S from the Fe (Fig. 5b and Supplementary Fig. 7c–f). After 30 min, a partial population of ACV adopts a rearranged conformation (~25%), with ~75% remaining bound to Fe (PDB: 8ALI). By 3 h, the rearranged form increases to ~75%, and the Fe-bound ACV drops to ~25% (PDB: 8ALJ). The Fe occupancy declines from 75% to 15%, respectively. No clear electron density indicative of O_2_ or O_2_-derived species was observed; the overall fold/active site is largely unchanged after O_2_ exposure, apart from a minor shift in Phe211. Although not unequivocally identifiable as a TAL, the rearranged ACV-derived species in the O_2_-exposed IPNS^H270E^ structures shows sulfur displacement from the Fe similar to that in the WT IPNS tr-SFX 3–6 s O_2_ timepoints (Fig. 2 and Supplementary Fig. 7k), suggesting TAL formation. The results with IPNS^H270E^ further highlight the importance of active-site waters in catalysis. They also support path b for β-lactam formation^12–14^ (Fig. 1), in which the distal oxygen of the peroxide intermediate deprotonates Fe-bound W1, generating a hydroxide that relays the TAL valine amide NH proton to enable MCI formation. Without a correctly positioned W1, as in IPNS^H270E^, the TAL is released and undergoes tautomerization or dimerization (with itself or with ACV) (Fig. 5a).

### Computational analyses supports transient TAL-S release

**Fig. 6:**
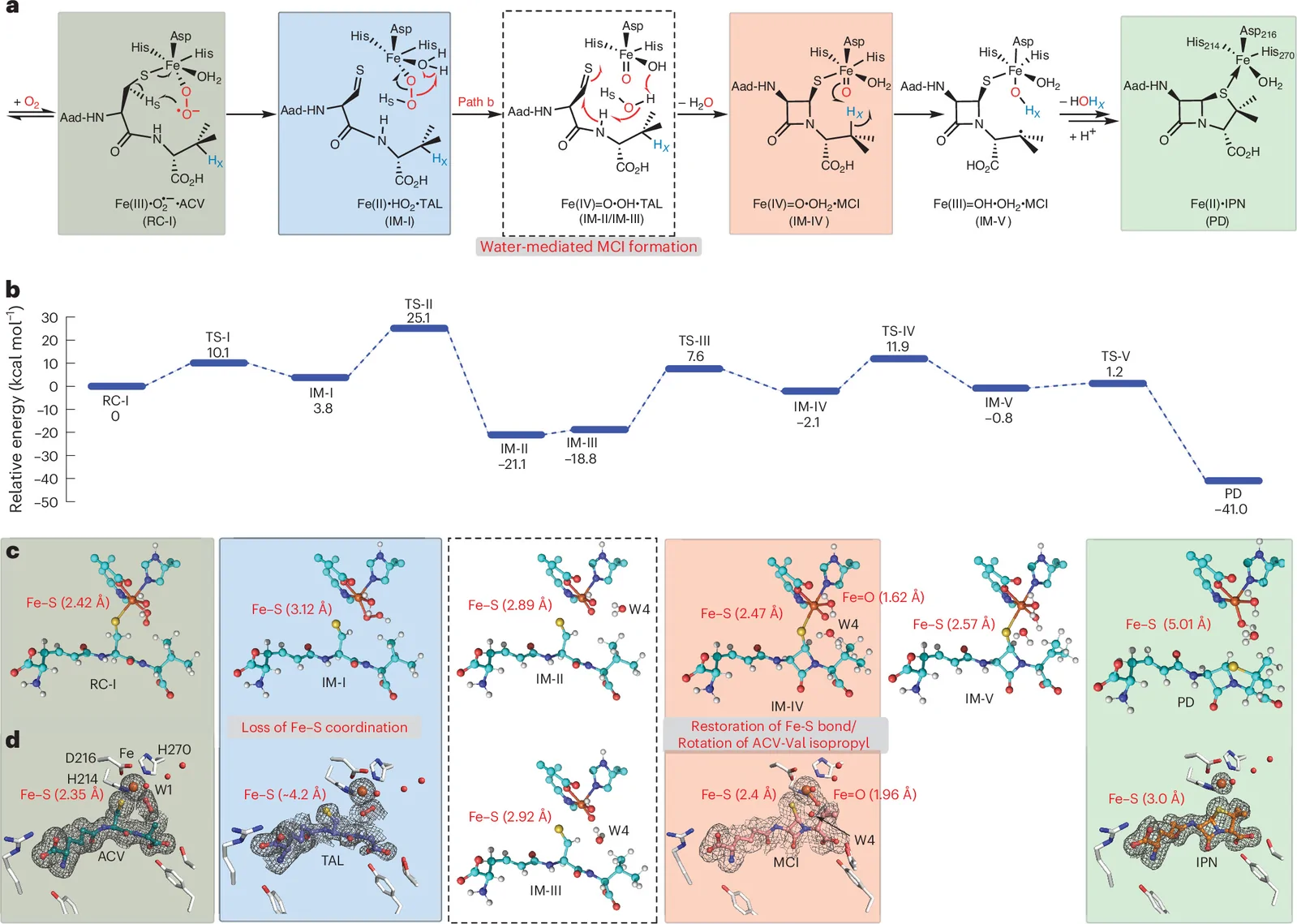
Mechanism of IPNS: energy landscape, intermediates and computational studies. **a**, Key intermediates with MCI formation proceeding via path b (Fig. 1). Note that the ACV-derived valine side chain undergoes reorientation along the reaction coordinate, enabling productive C–H cleavage at the MCI stage. **b**, The potential energy surface along the reaction coordinate at QM(B2 + ZPE (B3) + D3 correction)/MM energy level on the quintet surface. Relative energies are shown in kilocalories per mole. Transition states (TS-I to TS-V) are indicated along the reaction coordinate. **c**, QM/MM-optimized active-site geometries of selected intermediates (bond lengths in angstroms; the complete set is shown in Extended Data Fig. 9). Note, IPN dissociation occurs in the PD complex. **d**, Polder omit maps (3.0 σ contour level, carved around intermediates/IPN/Fe/O_2_). The panels show the main intermediates of IPNS:Fe:ACdV:O_2_ (Fe(III):O_2_^.−^:ACV, PDB: 8PY7, 1.49 Å, 1 s O_2_, pale green), IPNS:Fe:TAL:O_2_ (Fe(II):HO_2_:TAL, PDB: 8PYA, 1.50 Å, 4 s O_2_, blue), IPNS:Fe=O:MCI (PDB: 9I3B, 1.60 Å, 8 s O_2_, red) and IPNS:Fe:IPN (PDB: 9QPH, 1.70 Å, green; note that IPN release is blocked by lattice interactions). The modelling supports transient Fe–S release at the TAL stage with Fe–S restoration at the MCI stage, and MCI formation by Fe-bound W1-mediated peroxide cleavage (path b).

**Extended Data Fig. 9:**
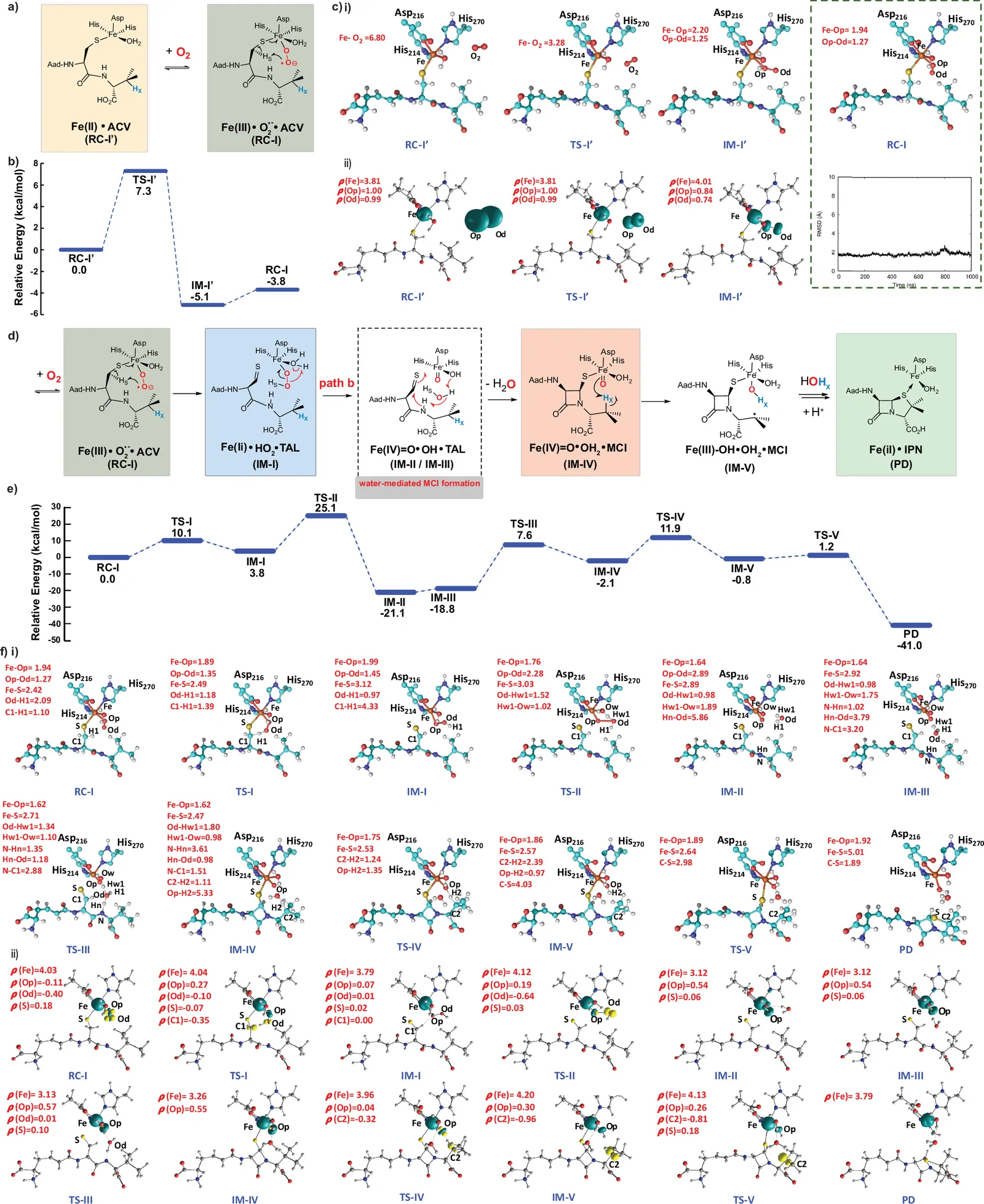
Computational analysis of the IPNS reaction mechanism. (**a**) Intermediates following O_2_ binding to IPNS:Fe:ACV. (**b**) Potential energy surface for O_2_ binding calculated at QM(B2 + ZPE (B3))/MM energy level on the septet surface, relative energies are in kcal/mol. (**c**) (**i**) QM/MM optimized active site geometries and (**ii**) spin density plots with corresponding spin densities (in brackets) during O_2_ binding (bond lengths in Å). TS-I’ during O_2_ binding represents to the highest energy structure along the reaction pathway. Green inset: MD simulation of the Fe(III)-superoxide complex RC-I shows a stable trajectory (average RMSD 1.78 Å). (**d**) Key intermediates proceeding via path b. (**e**) Potential energy surface along the IPNS reaction at the QM(B2 + ZPE (B3) + D3 correction)/MM energy level on the quintet surface, relative energies are in kcal/mol. (**f**) (**i**) QM/MM optimized active site geometries of selected intermediates and transition states (bond lengths in Å). Note IPN dissociation occurs in the PD complex. (**ii**) Spin density plots with spin densities (in brackets) for the transition states and intermediates formed during IPNS-catalysed reactions from RC-I.

We then tested the structurally proposed IPNS mechanism by computational studies. Molecular dynamics (MD) simulation of the Fe(III)–superoxide complex produced a stable trajectory with an average root mean square deviation of 1.78 Å (Extended Data Fig. 9c and Supplementary Figs. 12 and 13). For reaction path calculations, an averaged structure from the equilibrated region of the simulation (RC-I) (Fig. 6) was selected and subsequently refined by quantum mechanics/molecular mechanics (QM/MM) optimization. The resulting spin densities (Fe 4.03, O_2_ −0.51) were consistent with formation of an Fe(III)–superoxo species (Extended Data Fig. 9f(ii)), with the Fe–S bond length (2.42 Å) being in accord with experimental data (Fig. 6 and Extended Data Figs. 2c–g and 9f(i)). Formation of RC-I required an activation energy barrier of 7.3 kcal mol^−1^ (RC-I′) (Extended Data Fig. 9a–c), and O_2_ binds to the Fe(II) centre in an end-on fashion, consistent with experimental observations^11^ (Figs. 2 and 6 and Extended Data Figs. 2c–g and 9a–c). Cysteinyl hydrogen atom abstraction from RC-I yielded the IPNS:Fe(II):HO_2_:TAL intermediate (IM-I). Importantly, and in agreement with the tr-SFX results, the Fe–S bond is elongated to 3.12 Å in IM-I, and a TAL C=S double bond is clearly manifest (Fig. 6 and Extended Data Fig. 9d–f). Further, test calculations were performed to assess the accuracy of the QM/MM results (Supplementary Fig. 14).

Pathways a and b in Fig. 1, corresponding to peroxide cleavage and MCI formation, were investigated starting from the IPNS:Fe(II):O_2_H:W1:TAL intermediate (IM-I). For path a, all attempts to calculate peroxide-mediated NH abstraction and concerted MCI ring formation failed, or were energetically disfavoured, consistent with earlier computational studies^13,38^. For path b, the activation barrier for reaction of IM-I involving hydroperoxide cleavage mediated by W1 and formation of the IPNS:Fe(IV)=O(OH):TAL intermediate (IM-II) was 21.3 kcal mol^−1^, consistent with the experimental IPNS^H270E^ results supporting the importance of W1. In IM-II, the ferryl oxygen is positioned *trans* to Asp_216_ (Fe=O bond length of 1.64 Å) with an Fe–S distance of 2.89 Å (Fig. 6 and Extended Data Fig. 9d–f). Proton transfer reactions from IM-II via a water (W4, derived from the distal oxygen after peroxide cleavage of IM2) yielding the IPNS:Fe(IV)=O:MCI complex (IM-IV) were unsuccessful, indicating that reorganization of the water and the valine methyl groups is required. Reorientation of the ACV valine methyl groups and W4 resulted in IM-III, from which MCI (IM-IV) formation proceeded with a 26.4 kcal mol^−1^ activation barrier, restoring the Fe–S bond (2.47 Å).

The QM/MM studies imply that from IM-IV, valine C–H abstraction by the ferryl oxygen forms an IPNS:Fe(III)–OH intermediate and a ACV-Val C3 radical (IM-V) with a calculated activation energy of 14.0 kcal mol^−1^. Finally, the valine-derived carbon radical of IM-V reacts with the sulfur of the Fe–S species, generating an IPN product complex IPNS:Fe(II):IPN (PD) through a low activation barrier (2.0 kcal mol^−1^) in a highly exothermic reaction (−41.0 kcal mol^−1^). The geometries of the optimized structures with key distances and spin densities are given in Extended Data Fig. 9. However, considering the endothermicity of the IM-IV intermediate, the energy span for IM-V formation relative to IM-III is 30.7 kcal mol^−1^. To investigate whether this relatively high value arises from conformational variability, additional QM/MM calculations were performed using alternative MD-derived snapshots (Supplementary Figs 15–17). The resulting activation and reaction energies show substantial variation, particularly for the formation of IM-II (11.3 kcal mol^−1^), IM-IV (28.3 kcal mol^−1^) and IM-V (17.2 kcal mol^−1^). In particular, the relative energy of IM-IV varies by ~13 kcal mol^−1^, by contrast, the barrier from the transition state (TS-IV) relative to IM-IV varies by only ~3 kcal mol^−1^. Furthermore, single-point calculations using the PBE0 functional indicate that the energetics are sensitive to the level of theory (Supplementary Fig. 18). Overall, these results suggest that the effective barrier is probably lower than that estimated from static QM/MM calculations. Experimental studies reveal correlated motions of the protein during IPNS catalysis^11^ and show that the hydrogen atom transfer step exhibits a substantial KIE^4^, consistent with quantum tunnelling^11,39,40^. These features may not be fully captured in the static QM/MM calculations^41–44^ resulting in an artefactually high barrier for formation of IM-V. Note that key structural parameters modelled by us for intermediates in IPNS catalysis, including Fe–S, Fe–O_2_ and the Fe=O distances, are consistent with those modelled in mechanistically related Fe-dependent enzymes such as cysteine dioxygenase and enzymes involved in ovothiol and ergothioneine biosynthesis^45–49^, their reactions of which are proposed to proceed via Fe(III)-superoxo and Fe(IV) intermediates.

## Discussion

Our findings refine the sequence of reactions during IPNS catalysis (Fig. 6) and reveal how dynamics of both the overall protein and the Fe coordination sphere influence progression through high-energy intermediates (Figs. 2, 3 and 6). Importantly, they reveal how the structures and the reactivity of intermediates are linked to previously undefined roles of water molecules both within the active site and beyond.

Starting from the IPNS:Fe(II):ACV complex, the tr-SFX and tr-XES data reveal sequential formation of Fe(III)-superoxo:ACV, Fe(II)-hydroperoxide:TAL, Fe(IV)=O:MCI and Fe(II):IPN complexes. The combined XES spectral trends and structural results are consistent with prior solution-phase Mössbauer studies of IPNS, which independently identified Fe(III)-superoxo and Fe(IV)=O intermediates^4^. The tr-SFX analyses unexpectedly reveal that at the TAL stage the ACV-derived S atom does not coordinate the Fe(II), an observation supported by mutagenesis (Fig. 5) and consistent with substrate analogue studies^2,50^. Our results further support the formation of a monocyclic β-lactam intermediate (MCI) during IPNS catalysis^8^ via a stereoelectronically favoured 4-exo-trig cyclization of the TAL, which results in restoration of Fe–S coordination (Figs. 1, 2 and 6).

The combined tr-SFX data at room temperature, coupled with solution studies, demonstrate the importance of protein residues and water dynamics away from the immediate active-site vicinity in influencing the reactivity of intermediates in IPNS catalysis. Normalized B-factor analyses demonstrate the important roles of conformational changes involving helices α3 and α10, as well as β-sheet β11 (Fig. 2). It is likely that the extent of the conformational changes in solution is more extensive than observed in crystallographic studies owing to lattice constraints.

A hydrogen bond network involving N252 and a three-water chain extending from the Fe-bound water W1 through W2 and W3 is vital for productive catalysis (Figs. 2 and 4). To interrogate the role of this water network, we examined variant structures determined by conventional cryogenic synchrotron crystallography, which provide static snapshots rather than time-resolved intermediates. The subtle nature of the hydrogen bond network is shown by studies with the A, Q, E and D N252 variants, all of which form IPN in crystallo (Extended Data Fig. 8j,l). The A, Q and E variants also formed IPN in solution, albeit at a reduced rate. Remarkably, however, in solution, the most conservative substitution, N252D, resulted in the formation of a hydrated aldehyde (**1**), a two-electron oxidation product (Fig. 4). Together with the tr-SFX studies showing evidence for mobility of W2 in the 8 s and 10 s datasets (Fig. 2), these results support a critical role for the three-water chain in modulating the reactivity of intermediates, in particular with respect to Fe(IV)-coordination at the MCI stage.

Further evidence for the catalytic importance of W1, comes from studies with the IPNS^H270E^ variant, in which substitution of the Fe-chelating histidine with a glutamate disrupts W1 coordination and stalls the reaction at the TAL stage, as manifested by the observation of tautomeric ene thiol (**3** and **4**) derived products (Fig. 5). These observations support the critical function of W1 in enabling productive hydroperoxide cleavage forming an Fe(IV)=O complex via path b, in which the distal HO group of the hydroperoxide deprotonates the ACV derived valine NH (Fig. 1). Although the peroxide deprotonation and ferryl formation steps were not directly visualized in the XFEL datasets, probably due to their transient nature and/or mixed occupancies, the accumulation of the TAL intermediate in the H270E variant (Fig. 5), together with computational modelling, provides strong indirect support for operation of the proposed path b^2^ (Fig. 6).

The sensitivity of IPNS catalysis to subtle second-shell and metal-bound water perturbations provides a structural framework for engineering of IPNS to more efficiently produce existing (for example, penicillins with hydrophobic side chains^51^) as well as new β-lactam antibiotics. As strikingly demonstrated by the results with the N252 variants, they do, however, highlight the need for an improved understanding of dynamic changes involving both the protein polymer and other components, including water molecules, during catalysis.

The combined results highlight the power of tr-SFX combined with tr-XES to inform on the structures of transient intermediates during enzyme catalysis. At present there is no other experimental or computational method that can provide such high-resolution structural information. Exactly how the multiple dynamic elements involved in catalysis by IPNS and related 2OG-dependent oxygenases^18^, that is, Fe redox/coordination state, conformational changes and the water network, enable the sequential steps of oxygen activation, substrate C–H abstractions, consecutive Fe oxidation–reduction steps and, in the case of IPNS, conformational control of the intermediates including during the β-lactam and penicillin forming steps, is the subject of ongoing investigations employing both computational and experimental methods.

## Methods

Detailed procedures for cloning of IPNS constructs (Supplementary Table 1), expression and purification of IPNS variants, mass spectrometry, NMR and UV–visible experiments, and synthetic procedures are provided in the Supplementary Information.

### Crystallization of variant IPNS proteins and data collection

Crystals were prepared as reported but with slight modifications^11,22^. For single crystals, the IPNS variant (50 μl in 25 mM Tris pH 8.5, *c* = ~50 mg ml^−1^), ACV (1 mg), FeSO_4_·7H_2_O or CdCl_2_·2.5H_2_O and an IPNS:Fe:ACV crystal seed stock (10 μl) were transferred in an anaerobic chamber (Belle Technologies) for 30 min to remove residual O_2_. Crystallization solutions containing Li_2_SO_4_ (1.7 M in H_2_O) and Tris buffer (0.1 M in H_2_O, pH 8.5) for the batch setup were prepared as reported^11^. The IPNS:metal:ACV solutions were prepared by addition of a freshly prepared metal solution, for example, FeSO_4_·7H_2_O (8 equiv., 100 mM stock in H_2_O) or CdCl_2_·2.5H_2_O (4 equiv., 100 mM stock in H_2_O) to the IPNS solution (16 μl in 25 mM Tris pH 8.5, *c* = ~50 mg ml^−1^) followed by equilibration for 10 min. ACV (1 mg) was dissolved in Tris buffer (10 μl, 50 mM Tris in H_2_O pH 8.5) and added carefully in 3 × 3-μl portions to the individual IPNS:metal solutions. Precipitant in the IPNS:Cd:ACV solution was removed by centrifugation. Each individual protein solution (containing ACV and metal, ~22 μl) was added to the crystallization solution (220 μl). The mixture was pipetted into the wells (25 μl or 50 μl) of a 96-well plate (Corning); a 1:1,000; 1:10,000 or a 1:100,000 diluted seed stock solution was then added in a 1:50 ratio of protein containing crystallization solution to diluted seed stock. The plate was carefully sealed (Polyolefin StarSeal, Starlab) and stored in the anaerobic chamber for 24 h. Needle morphology crystals, typically with a 4 μm × 4 μm × 120–240 μm size, were collected for data collection. For crystal collecting, crystals were supplemented with cryo-protectant (crystallization buffer with 20% (v/v) glycerol), then collected with a nylon loop and cryo-cooled by rapid plunging into liquid N_2_ before data collection. Data for single crystals were collected at 100 K using synchrotron radiation at the DLS beamlines I03 and I04, then indexed, integrated and scaled using the Xia2 pipelines^52^ (Supplementary Tables 2 and 3). The crystal structures were determined by molecular replacement using the PHASER subroutine in PHENIX^53^ with the IPNS:Fe:ACV complex (PDB 6ZAE)^11^ as the reference model. Structural models were optimized by iterative cycles of manual re-building in COOT^54^ and crystallographic refinement in phenix.refine^53^. Data collection and refinement statistics are shown in Supplementary Tables 2 and 3.

### IPNS microcrystal preparation

IPNS microcrystals were prepared as reported, with minor modifications^11,22^. Crystallization was conducted within an anaerobic chamber (2 ppm or less O_2_; Belle Technology) with plates, solutions and other equipment used for crystallization deoxygenated in the chamber for at least 24 h. The IPNS solution used to grow crystals was deoxygenated in the anaerobic chamber for 1–2 h before use. IPNS microcrystals were prepared as reported^11,22^, using IPNS (200 μl, *c* = 52 mg ml^−1^), ACV or ACdV (2.1 mg), FeSO_4_·7H_2_O (100 mM stock in H_2_O) or vanadium(IV) oxide (50 mM stock in H_2_O), and seed stock solution (12 μl). Using a 96-well plate (Corning), crystallization solution for 30 wells, comprising Li_2_SO_4_ (1.995 ml, 2.7 M in Milli-Q), Milli-Q (405 μl) and Tris (300 μl, 1.0 M in Milli-Q pH 8.3), was prepared. The enzyme solution was prepared separately by supplementing IPNS (200 μl) with a freshly prepared FeSO_4_ solution (2.8 μl, 1.0 equiv., 100 mM in Milli-Q water) (or V(IV)=O (5.6 μl, 2.0 equiv., 100 mM in Milli-Q water) for the IPNS:V(IV)=O:ACV complex) and ACV or ACdV (2.1 mg dissolved in 40 μl Tris buffer, 25 mM pH 8.3). The protein solution (containing ACV and FeSO_4_, ~240 μl) was added to the crystallization solution. The seed stock (12 μl) was added and the mixture was pipetted into the wells (100 μl). The plate was carefully sealed (Polyolefin StarSeal, Starlab) and crystals were grown for 24–72 h. Needle-shaped microcrystals (*c* = 2 × 10^7^ microcrystals ml^−1^) of homogeneous size, typically with dimensions of 3 μm × 3 μm × 80 μm, were collected for data analysis.

### Setup and sample delivery for serial crystallographic data collection

Room temperature diffraction data for microcrystal slurries were collected at DLS beamline I24 (proposal MX-32727), the macromolecular femtosecond crystallography instrument^55^ of Linac Coherent Light Source (LCLS)^56^ (proposals LV56 and P-10035), the NCI beam line at PAL-XFEL^57,58^ (proposal 2019-2nd-NCI-029) and at the BL2 beamline^59^ at SACLA^60^ (proposal 2019A8088) (Supplementary Table 2). Serial synchrotron crystallography (SSX) experiments at I24 made use of fixed target sample delivery and a Pilatus6M detector (Dectris) to obtain aerobic IPNS:Fe:IPN. Further SSX fixed target data were collected under both anaerobic and aerobic conditions in preparation for all XFEL experiments^22,61^. The drop-on-tape sample delivery method combined with ADE^19^ was used to obtain anaerobic IPNS:Fe(II):ACdV and IPNS:V(IV)=O:ACV complex and time-resolved O_2_-exposed structures. The ejected droplets (~3.5–4 nl, flow rate 7 µl min^−1^) are deposited onto the conveyor belt at room temperature in an anaerobic environment (<500 ppm O_2_). Crystals were passed through a 60-mm reaction chamber, with reaction times controlled by the belt speed. For reaction initiation, the reaction chamber, including the conveyor belt with microdroplets of the anaerobic crystal slurry, was constantly purged with 100% O_2_ gas, resulting in a local >90% O_2_ atmosphere. At LCLS (experiment LV56), data were collected for anaerobic IPNS and for reaction times of 1, 2, 3, 4, 6, 8 and 10 s. In experiment P-10035 (LCLS), data were collected under anaerobic conditions and after 24 h of O_2_ exposure. At PAL-XFEL (experiment 2019-2nd-NCI-029), data were collected anaerobically and after 10 s of O_2_ exposure. At SACLA (experiment 2019A8088), anaerobic data were collected for the IPNS:V(IV)=O:ACV crystals. Detailed information about the O_2_ incubation times (including incubation time in the reaction chamber and additional travel time of microdroplets from the reaction chamber until they arrive in the X-ray interaction zone) are shown in Supplementary Table 2. The X-ray wavelength for experiments conducted under proposal LV56 was set to 1.30 Å (9.537 keV), for P-10035 to 1.282 Å (9.67 keV), for 2019-2nd-NCI-029 to 1.318 Å (9.41 keV) and for 2019A8088 to 1.24 Å (10.00 keV) with a data collection rate of 30 Hz, a pulse duration of ~35 fs and an X-ray beam size at the sample of ~2–3 μm in diameter. X-ray diffraction data were collected using a Rayonix MX340-HS detector for the LV56 and P-10035 beam time, on a RAYONIX MX225-HS detector for the 2019-2nd-NCI-029 beam time and on a RAYONIX MX300-HS detector for the 2019A8088 beam time, all with 4 × 4 binning.

### Data processing, model building and refinement of SFX datasets

Data processing was carried out as reported^20^. In brief, during the SFX experiments, data were monitored and processed using dials.stills_process-based^62^ scripts (PAL-XFEL) and cctbx.xfel (LCLS)^63^, the latter uses dials.stills_process as the core program, to index and integrate diffraction images. The initial estimation of detector geometry (beam centre and distance) was measured from silver(I) behenate (LCLS) and cerium(IV) oxide (PAL-XFEL) powder diffraction patterns. A first round of detector metrology refinement^64^ was performed followed by dials.stills_process-based strong spots indexing. One or more rounds of geometry refinement were carried out, until no substantial further increase of the indexing rate was observed. The detector geometry was checked and refined periodically during the experiments to identify any shifts of the sample or beam position. Integrated data were scaled and merged using cctbx.xfel.merge, with a reference model that has unit cell parameters of *a* = 41.2 Å, *b* = 75.0 Å, *c* = 101.9 Å and *α* = *β* = *γ* = 90° (space group P2_1_2_1_2_1_). The merging resolution was selected on the basis of several criteria, including where the multiplicity drops below tenfold, the CC_1/2_ no longer decreases uniformly and the completeness falls below 99%. Structures were solved by molecular replacement using the PHASER subroutine in PHENIX^53^ with the IPNS:Fe:ACV complex (PDB: 6ZAE)^11^ as the reference model. The structural model was optimized by iterative cycles of manual re-building in COOT^54^ and crystallographic refinement in phenix.refine^53^. The restraint files for the MCI and for the TAL were generated using Grade Webserver (https://grade.globalphasing.org/) after providing the SMILES generated in ChemDraw. The Fe=O restraint was adapted from the V=O (VVO) ligand (https://www.rcsb.org/ligand/VVO) by substituting vanadium with iron. Fe=O bond restraints from 1.64 to 2.10 ± 0.02 Å were tested in successive refinement rounds. Shorter restraints consistently led to Fe–O bond elongation during refinement, indicating they were too restrictive. By contrast, refinements using a 1.96 ± 0.02 Å restraint resulted in minimal residual density and stable bond lengths, supporting this as the Fe–O bond distance. For the IPNS:V(IV)=O:ACV dataset, the same procedure led to a refined V=O bond length of 1.64 Å. Final models were selected on the basis of minimal difference density and consistent bond geometry. Data collection and refinement statistics are presented in Supplementary Table 3.

### Data processing of tr-XES data

The on-demand ADE/tape-drive sample delivery setup enables simultaneous collection of tr-XES and tr-SFX^19^ from the same sample and X-ray pulse to study Fe-oxidation states. We used a wavelength-dispersive von Hamos spectrometer with four cylindrically bent (*R* = 250 mm) germanium (440) crystals orthogonal to the sample with the centre of the crystals located at 75° with respect to the interaction point^65,66^. The spectrometer was mounted vertically during experiments LV56 and P-10035, and horizontally in the experiment 2019-2nd-NCI-029, to minimize the elastic scattering signal from the vertically and horizontally polarized X-ray beam at LCLS and PAL-XFEL, respectively. Each analyser crystal focused the emitted Fe Kα_1,2_ X-ray photons onto an ePix100 detector (during experiment LV56 and P-10035) and a Jungfrau 0.5M detector (during experiment 2019-2nd-NCI-029). To accommodate the spectrometer orientation, the detector was installed horizontally adjacent to the interaction point during LV56 and P-10035, and located vertically below the sample X-ray interaction region during 2019-2nd-NCI-029. The wavelength calibration was performed using aqueous solution of 3 mM Fe(III)Cl_3_.

Detector images were sorted by the sample hit rate as reported^11,17^. For spectral analysis, the spectra were area-normalized in the 6,380–6,415 eV range. Smoothed spectra were calculated using a second-order Savitzky–Golay filter with an 11-point window size. Overlay of the non-smoothed and the smoothed spectra, for both the individual Kα_1,2_ XES and the difference spectra, are shown in Extended Data Fig. 4. The first moment of the Kα_1_ peak was calculated in the 6,395–6,410 eV interval on the smoothed spectra. To calculate error bars, the standard deviation of the residual of the smoothed curve was calculated in the same energy range. This standard deviation was propagated through the calculation of the first moment, and added as error bars. The FWHM and the half-width at half-maximum were calculated on the area-normalized spectra in the 6,395–6,410 eV range. For both FWHM and the first moment, variable spectral ranges were tested for the lower bound (6,395–6,400 eV) and the upper bound (6,407–6,410 eV) to confirm that the calculated trend is not an artefact of the selected spectral range. For the calculation of the IAD, the spectral ranges of 6,382–6,412 eV, 6,395–6,410 eV and 6,386–6,395 eV were selected for consideration of the Kα_1,2_ ensemble, Kα_1_ peak and Kα_2_ peak, respectively.

### Normalized B-factor analysis for tr-SFX data

Each dataset was initially refined against the same model using molecular replacement with model PDB: 8PY6, followed by two rounds of refinement, during which ligand and Fe occupancies were set to 1.0 and automatic water addition was enabled. To assess relative atomic displacement, normalized B-factor analysis was performed using PyMOL. Backbone B-factors (N, CA, C and O) were extracted while excluding solvent and side-chain atoms, whereas for the analysis of the mobile water chain and side-chains, ligands and water molecules were included in a separate analysis. The extracted B factors were normalized using a *Z*-score transformation, calculated as (B−*μ*)/*σ*, where *μ* and *σ* represent the mean and standard deviation of the backbone B factors, respectively. The normalized values were then reassigned to the structure to allow direct comparison across datasets. To visualize spatial distribution of B-factor variations, a colour gradient from blue (low) to red (high) was applied to the backbone, with white representing intermediate values. A corresponding colour legend was generated to indicate the normalized B-factor range (−2 to +2). Finally, the structure was shown in PyMOL to apply the updated B factors and ensure proper visualization.

### System preparation for computational analyses

A crystal structure of the IPNS:Fe:ACdV:O_2_ complex (PDB ID: 8PY7), representing the Fe(III)-superoxo intermediate, was used for the initial modelling. Missing atoms in the ACV valine were modelled using GaussView 6.0. To maintain the linearity of the ACV side chain, a double bond was introduced in the δ-(l-α-aminoadipoyl) residue, as described in previous studies^21^. Amber routines were used to add missing hydrogen atoms to the protein, assigning protonation states for titratable residues^67^. The antechamber module with the GAFF tool was employed to generate topology files for the non-standard residues, including Fe(III), O_2_ and the substrate^68^. Parameters for the Fe(III) centre (high spin, *S* = 2 and *M* = 5) and its coordinating residues: His_214_, Asp_216_, His_270_, O_2_ and ACV, were generated using Metal Center Parameter Builder (MCPB.py)^69^. The Amber FF14SB force field was utilized to parameterize the remaining regions of the protein^70^. The system was solvated with the TIP3P water model, extending 10 Å from the most distant point on the protein surface^71^. The system was neutralized by adding Na^+^ counterions through the Leap module of Amber 18^72^.

### Molecular dynamics simulation

Initially, a two-stage energy minimization was carried out to eliminate unfavourable contacts between water molecules and the protein. In the first stage, solvent molecules were minimized while applying a 100 kcal mol^−1^ Å^−2^ restraint on the solute. In the second stage, the entire system was minimized without restraints. Each minimization stage consisted of 5,000 steps of the steepest descent method followed by 5,000 steps of the conjugate gradient method.

The minimized system was heated from 0 to 300 K under an NVT (constant number of particles (N), volume (V) and temperature (T)) ensemble using a Langevin thermostat over 300 ns, with a restraint of 50 kcal mol^−1^ Å^−2^ applied to the solute molecules^73^. Following heating, the system was maintained at 300 K in an NPT (constant number of particles (N), pressure (P) and temperature (T)) ensemble for 1 ns with a weak restraint on the solute molecule to achieve constant density. Equilibration was performed for 50 ns at 300 K and 1 bar in an NPT ensemble. From the equilibrated structure, a 1-µs production MD simulation was carried out at 300 K and 1 bar in an NPT ensemble without any restraints. Pressure was maintained during the simulations using the Berendsen barostat^74^, and the SHAKE algorithm was applied to constrain hydrogen bonds^75^. Long-range electrostatics were treated with the particle mesh Ewald method^76^, using a 10-Å cutoff for non-bonded interactions. All simulations were run with the GPU-accelerated version of the Amber18-PMEMD package^77^, and periodic boundary conditions were applied throughout the simulations. The stability of the MD simulations was assessed by performing root mean square deviation analysis. The key analyses of the MD trajectories are given in Supplementary Figs. 12 and 13.

### QM/MM calculations

A snapshot from a well-equilibrated region of the MD simulation, representing the average structure, was used for QM/MM studies. The system was initially prepared by removing excess water molecules beyond 12 Å from the surface of the protein. QM/MM calculations were performed using ChemShell software^78^, which integrates Turbomole^79^ for the QM region and DL-POLY^80^ for the MM region. Hydrogen link atoms were added at the QM/MM boundary using the charge-shift model^81^. The QM region comprised first coordination sphere residues: His_214_, Asp_216_, His_270_, O_2_ and the substrate. Facial triad residues (His_214_, Asp_216_ and His_270_) were truncated between Cα and Cβ carbons, retaining only the side chains in the QM region. The MM region included residues within 8 Å of the QM residues, while all residues beyond the MM region were kept fixed. Unrestricted B3LYP (UB3LYP) functional^82^, and the Amber FF14SB force field^70^, were utilized to treat the QM and MM regions, respectively.

Consistent with previous density functional theory (DFT) studies on IPNS, QM/MM calculations were performed in the high-spin quintet state (*S* = 2, *M* = 5)^14,38^. Further, previous studies on non-haem Fe(II)-containing enzymes indicate that dioxygen binds to the Fe(II) centre on the septet surface (*S* = 3, *M* = 7); therefore, all calculations involving dioxygen binding were performed in the septet state^83–86^. For dioxygen binding studies, the starting structure was generated by running a reverse scan for unbinding the dioxygen from the Fe(III)-superoxo complex at the septet surface. All QM/MM optimizations and frequency calculations were performed using UB3LYP with the def2-SVP basis set [QM(B1)/MM]^87^. Relaxed potential energy scans with a 0.1-Å step size, starting from optimized geometries, were used to locate transition states and reaction intermediates. The highest points along the potential energy surface were optimized using the dimer method. Frequency calculations were performed on all QM/MM-optimized geometries to confirm whether they are minima or transition states. Energy corrections were performed for all optimized QM/MM geometries by performing single-point calculations with the def2-TZVP basis set [QM(B2)/MM]^87^, and the reported energies [QM(B3)/MM] included zero-point energy (ZPE) corrections to the [QM(B2)/MM] energies. To assess the accuracy of the QM/MM calculations, additional calculations were performed for the first step of the reaction, that is from the Fe(III)-superoxo complex to the IPNS:Fe(II):HO_2_:TAL intermediate (IM-I), using (1) an extended QM region including W2, W3 and the SCS residue Asp252; and (2) the def2-TZVP basis set. The resulting activation and reaction energies show consistent trends with those obtained using the standard reaction mechanism. Notably, with the def2-TZVP basis set, the formation of IM-I is predicted to be exothermic; however, the overall mechanistic conclusions remain unchanged (Supplementary Fig. 14). To investigate the influence of conformational variability in the starting structure on the activation and reaction energies, QM/MM calculations were performed using an alternative snapshot from the MD simulation (Supplementary Figs. 15–17). Similarly, to evaluate the effect of the computational method on the energetics, single-point calculations were performed using the PBE0 functional (Supplementary Fig. 18).

### Reporting summary

Further information on research design is available in the Nature Portfolio Reporting Summary linked to this article.

## Supplementary information

Supplementary Information Supplementary Methods, Tables 1–3, Figs. 1–18, NMR spectra, Coordinates for QM/MM and molecular dynamics, and References.

Reporting Summary

Peer Review File

## Source data

Source Data Fig. 1 Source data for XES, UV–visible and mass spectrometry data. Worksheet 1: Fig. 2 and Extended Data Fig. 3 XES spectra. Worksheet 2: Fig. 2 and Extended Data Fig. 3 difference spectra. Worksheet 3: Extended Data Fig. 4 spectral analyses. Worksheet 4: Extended Data Fig. 4 reference correlation. Worksheet 5: Extended Data Fig. 5 and Supplementary Fig. 6 SPE MS N252. Worksheet 6: Supplementary Fig. 8 UV–visible spectroscopy. Worksheet 7: Supplementary Fig. 8 native MS H270E.

Source Data Fig. 2 Atomic coordinate file for the initial coordinates of MD simulations.

Source Data Fig. 3 Atomic coordinate file for the final coordinates of MD simulations.

## Data Availability

The atomic coordinates and structure factors are deposited in the PDB with the following accession codes: 8PY6 (IPNS:Fe:ACdV, anaerobic, SFX), 8PY7 (IPNS:Fe:ACdV:O_2_, 1 s O_2_, SFX), 8PY8 (IPNS:Fe:ACdV:O_2_, 2 s O_2_, SFX), 8PY9 (IPNS:Fe:ACdV/TAL:O_2_, 3 s O_2_, SFX), 8PYA (IPNS:Fe:ACdV/TAL:O_2_, 4 s O_2_, SFX), 9I3A (IPNS:Fe:TAL:O_2_, 6 s O_2_, SFX), 9I3B (IPNS:Fe/Fe–O:TAL/MCI, 8 s O_2_, SFX), 9I3C (IPNS:Fe/Fe–O:TAL/MCI, 10 s O_2_, SFX), 9QPH (IPNS:Fe:IPN (from ACdV), 24 h O_2_, SFX), 8PY5 (IPNS:Fe:ACdV, SFX), 9I3D (IPNS:Fe/Fe–O: MCI/IPN, 10 s O_2_, SFX), 6ZAK (IPNS:VO:ACV, SFX),9QPI (IPNS:Fe:ACdV, anaerobic, SFX), 9QPJ (IPNS:Fe:IPN (from AcdV), 24 h O_2_, SSX), 8P48 (IPNS_N252D:Fe:ACV, MX), 8P46 (IPNS_N252D:Fe:IPN, 30 min O_2_, MX), 8P47 (IPNS_N252A:Fe:ACV, MX), 8PTV (IPNS_N252Q:Fe:ACV, MX), 8RDB (IPNS_N252E:Fe:ACV, MX), 9HWF (IPNS_N252E:Fe:IPN, 30 min O_2_, MX), 7ZOE (IPNS_H270E:Fe:ACV, MX), 8A4G (IPNS_H270E:Cd:ACV, MX), 8ALI (IPNS_H270E:Fe:ACV, 30 min O_2_, MX), 8ALJ (IPNS_H270E:Fe:ACV, 3 h O_2_, MX) and 9QPG (IPNS:Fe:IPN (from AcdV), 18 h O_2_, MX). Coordinates for the molecular dynamics and QM/MM calculations are provided in the Supplementary Information. PDB files with coordinates for the structures from the MD simulations initially and after optimization are provided as supplementary data files. All other data supporting the findings of this study are available within the Article and its Supplementary Information. Source data are provided with this paper.
