# Supplementary material for: Unanticipated intermediates during isopenicillin N synthase catalysis identified by time-resolved X-ray free-electron laser studies
Source: Nat Catal. 2026 Oct 9;9(10):1141–57. doi: 10.1038/s41929-026-01618-4 (PMC13690604; doi:10.1038/s41929-026-01618-4)
Supplement: Supplementary file 1 [file 41929_2026_1618_MOESM1_ESM.pdf]

nature catalysis

Supplementary information

https://doi.org/10.1038/s41929-026-01618-4

Unanticipated intermediates during isopenicillin N synthase catalysis identified by time-resolved X-ray free-electron laser studies

In the format provided by the
authors and unedited

Contents:

Supplementary Methods

Supplementary Tables 1 to 3

Supplementary Figures 1 to 18

NMR spectra

Coordinates for QM/MM and molecular dynamics

Supplementary References

Supplementary Methods

Materials and methods

Chemicals for buffers and crystallization screens were from commercial suppliers and were used as supplied except where stated. ACV was synthesized by solid-phase peptide synthesis as reported and in analogy to the procedure for ACdV in the SI.[1] Procedures for the synthesis of deuterated ACdV, ($^{13}C_3$,$^{15}N_2$)ACV and the monocyclic mercury salt are described in the Supplementary Information.

Site-directed mutagenesis of the IPNS H270E variant

The pCOLD I vector (Addgene, UK) containing DNA encoding for *Aspergillus nidulans* IPNS[1] was used to produce IPNS variants with an *N*-terminal $(His)_6$ tag and human rhinovirus C (HRV3C) protease cleavage site. The IPNS H270E and N252X variants were produced using the Q5 site-directed mutagenesis kit (New England Biolabs, USA) using the manufacturer's protocol; primers were generated by Merck Life Science UK and sequences are given in Supplementary Table 1. After PCR reactions were complete, DpnI digestion was performed (37 °C, 3 hours). The plasmid-containing solution was transformed into XL10 Gold ultra-competent cells (Agilent, USA) using heat-shock (42 °C, 30 seconds). After two minutes at 4 °C, cells were incubated in 500 µL 2YT medium (60 min, 37 °C). Cell colonies were grown overnight on 2YT agar plates with kanamycin (50 µg mL^{-1}). Single colonies were picked and used to inoculate 10 mL cultures in 2YT medium with kanamycin (50 µg mL^{-1}). Plasmid DNA was isolated using a GeneJET Plasmid Miniprep kit (Thermo Fisher Scientific, USA); desired variants were confirmed by DNA sequencing (Eurofins, Germany).

Supplementary Table 1: **Primers used for site-directed mutagenesis.**

**IPNS variant**	**Starting template**	**Primer Name**	**Sequence** (melting temp.)
$IPNS^{H270E}$	pCOLD_IPNS[1]	IPNS_H270E_fwd	CCGATTGAACGTGTGAAATGG GTTAATGC (T_m = 70°C)
		IPNS_H270E_rev	CCATTTCACACGTTCAATCGGT GC (T_m = 69°C)
$IPNS^{N252A}$	pCOLD_IPNS[1]	IPNS_N252A_fwd	CTATCTGATTGCTTGTGGTAGC TATATGGCACACC (T_m = 72°C)
		IPNS_N252A_rev	CTACCACAAGCAATCAGATAG CCGGTATCATCTG (T_m = 72°C)
$IPNS^{N252D}$	pCOLD_IPNS[1]	IPNS_N252D_fwd	GCTATCTGATTGATTGTGGTAG CTATATGGCACACC (T_m = 72°C)
		IPNS_N252D_rev	CTACCACAATCAATCAGATAG CCGGTATCATCTGC (T_m = 72°C)
$IPNS^{N252Q}$	pCOLD_IPNS[1]	IPNS_N252Q_fwd	GCTATCTGATTCAATGTGGTAG CTATATGGCACACC (T_m = 72°C)
		IPNS_N252Q_rev	CTACCACATTGAATCAGATAG CCGGTATCATCTGC (T_m = 72°C)
$IPNS^{N252E}$	pCOLD_IPNS[1]	IPNS_N252E_fwd	GCTATCTGATTGAATGTGGTAG CTATATGGCACACC (T_m = 72°C)
		IPNS_N252E_rev	CTACCACATTCAATCAGATAG CCGGTATCATCTG (T_m = 72°C)

Production and Purification of IPNS variants

The production and purification of IPNS WT, H270E and N252X were performed as reported with minor modifications.[1] Plasmids encoding for variants were transformed into *E. coli* BL21 cells using a heat-shock protocol and grown on 2YT agar plates with kanamycin (50 µg mL^{-1}). Single colonies were picked to inoculate a starter culture of 2YT medium (100 mL, 50 µg mL^{-1} kanamycin). Cells were grown overnight with shaking (150 rpm, 37 °C). The starter culture (6 mL, 1:100 v/v) was used to inoculate

large-scale growth (6 or 12 x 600 mL auto induction medium terrific broth, Formedium, UK) containing kanamycin (50 µg mL^{-1}). Cells were grown (150 rpm, 37 °C) until optical density (OD_{600}) of 0.6. The cultures were incubated overnight at 15 °C. Cells were harvested by centrifugation (11,000 × g, 8 minutes, 4 °C); cell pellets were stored at -80 °C.

Proteins were purified by re-suspension of the cell pellet in binding buffer (1:5 w:v; 50 mM tris, 200 mM NaCl, 5 mM imidazole pH 8.0) containing DNAse I (10 µg mL^{-1}), phenylmethylsulfonyl fluoride (PMSF, 10 µg mL^{-1}) and lysozyme (0.2 mg mL^{-1}). Lysis was performed using a cell disruptor (Constant Systems, UK); debris was removed by centrifugation (58,000 × g, 30 min, 4 °C). The supernatant was filtered (2 µm syringe filter) and loaded onto a nickel affinity column (5 mL HisTrap HP, GE Healthcare, USA), pre-equilibrated with binding buffer. The protein-loaded column was washed with binding buffer (20 CV). Elution was achieved by applying a buffer gradient (20 CV) from the binding buffer to the elution buffer (50 mM tris, 200 mM NaCl, 500 mM imidazole pH 8.0). IPNS-containing fractions were identified by SDS-PAGE analysis, then concentrated by centrifugation (10k MWCO, 3,000 × g, 4 °C; Merck Millipore, USA). Proteins (c = ~10 mg mL^{-1}) were treated with 3C protease (1:100, w:w); the mixture was dialysed overnight against binding buffer (1 : 500 dilution, 4 °C, 10 k MWCO, Slide-A-Lyzer®, Thermo Fisher Scientific, UK) to remove imidazole. Proteins were loaded onto a nickel affinity column (5 mL HisTrap HP, GE Healthcare, USA); untagged IPNS was eluted with 5 CV of binding buffer. Cleavage of the hexa-histidine tag was confirmed by MS analysis using a Xevo G2-XS mass spectrometer (Waters, UK). Untagged IPNS was loaded into a dialysis cassette (10 kDa MWCO, Slide-A-Lyzer®, Thermo Fisher Scientific, UK) and buffer exchanged against dialysis buffer (50 mM tris, 200 mM NaCl, 30 mM EDTA, 5 mM 1,10-phenanthroline, pH 8.0, 2 hours, 4 °C) to remove any metal ion. Size-exclusion chromatography (Superdex 75 column, 300 mL, GE, Healthcare, USA) with size exclusion buffer (50 mM tris, 200 mM NaCl pH 8.0, Chelex resin treated, BioRad, US) was carried out for further purification and EDTA removal. *Apo*-IPNS was concentrated to ~50 mg mL^{-1}, aliquoted, flash-frozen in liquid nitrogen and stored at -80 °C.

Activity assays of IPNS with labelled and unlabelled ACV using NMR spectroscopy

IPNS WT or IPNS variants were buffer exchanged into NMR buffer (25 mM Tris-d_{11} in H_2O, pH 7.5) using microspin columns (BioRad, UK) and concentrated using Amicon Ultra Centrifugal Filters (0.5 mL, MWCO: 10 kDa). Stock solutions were as follows: IPNS WT and variants (~1.50 mM stock), $FeSO_4$ x 7 H_2O (50 mM stock in H_2O), *L*-ascorbate (100 mM stock), TCEP (100 mM stock) and ACV (100 mM stock in 25 mM Tris-d_{11} in H_2O, pH 7.5). Turnover experiments were performed using IPNS WT or variants (5 or 150 µM), $FeSO_4$ x 7 H_2O (50 or 450 µM), *L*-ascorbate (1 mM), in the absence or presence of TCEP (1 mM), and ACV (250 µM, 500 µM or 4.8 mM). Typical reactions were performed under ambient O_2; reactions with higher substrate and enzyme concentrations were performed using solutions purged with 100% O_2. Anaerobic controls were done in an anaerobic chamber (Belle Technology, UK, <2 ppm O_2). For experiments using labelled ($^{13}C_3$,$^{15}N_2$)ACV, the conditions used were: IPNS^{H270E} (150 µM), $FeSO_4$ (250 µM), *L*-ascorbate (2 mM), TCEP (2 mM), ($^{13}C_3$,$^{15}N_2$)ACV (2 mM) and iodoacetic acid (5 mM) in Tris-d_{11} in 90% H_2O/10% D_2O (conditions differing from these conditions are given in relevant figure legends). NMR spectra were obtained using a Bruker AVIII 700 MHz NMR spectrometer equipped with an inverse 5mm TCI ^{1}H cryoprobe. Samples were prepared in 3 or 5 mm regular or J Young valve NMR tubes (Norell, USA) at 298 K. Carr-Purcell-Meiboom-Gill (CPMG) NMR spectra were recorded by applying the PROJECT-CPMG sequence.[2] Experimental parameters: total echo time, 40 ms; relaxation delay, 2 s. Water suppression was achieved by pre-saturation. ^{1}H NMR spectra were processed with 3 Hz Lorentzian line broadening using MestReNova 14.1 (MestReLabs, Spain; www.mestrelab.com) and TopSpin 3.6.1 (Bruker, Germany; www.bruker.com).

Sample preparation for UV/Vis experiments

Sample preparation was performed in an anaerobic chamber (2 ppm or less O_2, Belle Technology, UK). Stock solutions of UV/Vis buffer (100 mM MOPS pH 7.2 in H_2O) and solids ($FeSO_4$ x 7 H_2O, ACV) were placed in the anaerobic chamber to remove residual O_2. Stock solutions of *apo*-IPNS H270E (119 mg mL^{-1}, ~3.15 mM) were transferred into the anaerobic chamber prior to use. Solutions of ACV (100 mM in UV/Vis buffer) and $FeSO_4$ x 7 H_2O (50 mM in H_2O), were prepared in the chamber. The total volume of a typical sample was 100 µL (or with simultaneous sample collection for EPR analysis 450 µL) and contained 300 µM IPNS H270E, 300 µM $FeSO_4$ (1 equivalent), 1.50 mM ACV (5 equivalents) and residual UV/Vis buffer. After equilibration, samples were transferred from plastic tubes (0.5 mL) into a quartz cuvette, then sealed with a rubber septum.
For subsequent O_2 additions, a quartz cuvette containing a defined volume of 100 or 450 µL protein solution was transferred into the cuvette holder of a Cary 3500 spectrometer and allowed to equilibrate to 20 °C. Oxygenated UV/Vis buffer (100 mM MOPS pH 7.2 in H_2O + O_2) was prepared by purging the solution with O_2 for 20 minutes. Reactions were initiated by simultaneous removal of the rubber septum and addition of an equal volume of oxygenated UV/Vis buffer; UV/Vis spectra were then recorded, using a Cary 3500 UV/Vis Compact Peltier spectrometer (Agilent, US). Samples were prepared and data collected in a rectangular, quartz, ultra-micro, open top cuvette (2.5 x 5 mm, 10 mm pathlength, minimal volume: 70 µL), sealed with a rubber septum in an anaerobic chamber (< 2 ppm O_2, Belle Technology, UK). Data were recorded at 293 K, with scan range from 200 -800 nm (1 nm data interval), a scan rate of 3,000 nm/min and, averaging time of 0.02 s. UV/Vis spectra were processed using the Cary UV workstation (Agilent, US).

Preparation of IPNS enzyme for nondenaturing MS analyses

Purified wt IPNS or H270E were buffer exchanged into ammonium acetate (1 M) using Micro Bio-Spin® 6 Chromatography Columns (Bio-Rad). Stock solutions of IPNS (250-500 µM in ammonium acetate), $FeCl_2$·x H_2O (1 mM in H_2O) or $CdCl_2$·x 2.5 H_2O (1 mM in H_2O) and ACV (1 mM in ammonium acetate) were prepared. Samples were mixed with the different (co-) substrates as described in relevant figure legends. Solutions containing Fe(II) and ACV were prepared and a 3 µL aliquot was transferred into gold-coated borosilicate capillary (Harvard Apparatus) under anaerobic conditions. The capillary was transported in an anaerobic chamber and mounted on the nano ESI source under minimum exposure to O_2. Non-denaturing MS data were acquired using a Q-Exactive hybrid quadrupole-Orbitrap mass spectrometer (Thermo Fisher Scientific, Bremen, Germany). The instrument settings were: spray voltage 1.2 kV, S-lens RF 200%, argon UHV pressure 3.3 × 10−10 mbar, the capillary temperature was set to 100 °C, and the resolution of the instrument was set to 17,500 at a transient time of 64 ms. The voltages of the ion transfer optics –injection flatapole, inter-flatapole lens, bent flatapole, and transfer multipole were 5, 3, 2, and 30 V respectively. The noise level was set at 3. Proteins were activated by applying 0 V in the high-energy collisional dissociation cell without in-source trapping. Data were visualised and exported for processing using the Qual browser of Xcalibur 4.1.31.9 (Thermo Scientific).

Mass spectrometry turnover assay and trapping experiments of IPNS variants

MS experiments were performed using a HPLC coupled to a Xevo G2-S mass spectrometer or a RapidFire RF 365 high-throughput sampling robot coupled to an iFunnel Agilent 6550 accurate-mass quadrupole time-of-flight mass spectrometer.

Turnover and pH dependency assays (final concentrations: 1 µM for WT and 5 µM for IPNS N252X variants, in 25mM tris buffer, varying pH from pH 6.0 – pH 9.0 in steps of 0.5) using the HPLC were performed after addition of $FeSO_4$ (20 µM in H_2O), ACV (100 µM) and sodium ascorbate (100 µM) in tris buffer (25 mM in H_2O, varying pH from pH 6.0 – pH 9.0 in steps of 0.5) prepared under ambient O_2 conditions for 2 h and 16 h. Samples were diluted by addition of MQ water to give a final ACV concentration of 10 µM and analysed using a Xevo G2-S mass spectrometer (Waters) coupled to an Acquity-UPLC system (Waters), equipped with a ACE Equivalence C18 2.1-mm x 50-mm column,

loaded onto the column in 99% (v/v) water, 1% (v/v) acetonitrile, and 0.1% (v/v) formic acid. Samples were eluted using a gradient to 50% (v/v) water, 50% (v/v) acetonitrile, and 0.1% (v/v) formic acid over 7 min. Data were analysed using MassLynx4.1, with deconvolution using the MaxEnt1 algorithm.

Alkylation reactions with iodoacetic acid to identify products of IPNS H270E were performed with IPNS (5 μM, 200 μL) in tris buffer (25 mM in H_2O, pH 8.0) after addition of $FeSO_4$ (20 μM in H_2O), ACV (100 μM) and sodium ascorbate (100 μM) in presence of absence of TCEP (100 μM) or Iodoacetic acid (250 μM). The reaction mixture was incubated for at least 3h, then quenched with 800 μL acetonitrile. Precipitated protein was removed by centrifugation; samples were analysed using a Xevo G2-S mass spectrometer (Waters) coupled to an Acquity-UPLC system (Waters) as above. Identified compounds were compared to synthetic standards obtained by mercury salt hydrolysis with H_2S followed by iodoacetic acid addition (see general synthetic procedures).

Real-time turnover assays using the RapidFire high-throughput sampling robot[3] were performed using the following cofactor stock solutions: L-ascorbic acid: 100 mM in water; ammonium iron(II) sulfate hexahydrate $(NH_4)_2Fe(SO_4)_2 \cdot 6H_2O$: 100 mM in 20 mM HCl diluted to 10 mM in water, TCEP: 100 mM in water, which were freshly prepared from commercially sourced solids (Sigma–Aldrich). Assays were performed in 2 ml deep well blocks (Greiner Bio-One GmbH) with final concentrations of enzyme (2 μM), ACV (400 μM, from an initial stock solution: 100 mM, in 25 mM Tris, 100 mM NaCl [pH 8.0]), $(NH_4)_2Fe(SO_4)_2$ (100 μM), TCEP (800 μM) and L-ascorbate (800 μM) under ambient O_2 levels. Reactions were initiated by addition of enzyme and monitored in real time using solid-phase extraction coupled to MS.MS analyses were performed using a RapidFire RF 365 high-throughput sampling robot (Agilent) attached to an iFunnel Agilent 6550 accurate-mass quadrupole time-of-flight mass spectrometer operated in the positive ionization mode.

Assay samples were aspirated under vacuum for 0.6 s, then loaded onto a HILIC-Z (type H6) solid phase extraction (SPE) cartridge that had been equilibrated in 0.1% (v/v) aqueous formic acid in 85/15 (v/v) acetonitrile/water. After loading, the HILIC SPE cartridge was washed with 0.1% (v/v) aqueous formic acid in 85/15 (v/v) acetonitrile/water (5.0 s, 1.0 ml min^{-1}). Analytes were eluted from the SPE cartridge with 0.1% (v/v) aqueous formic acid in 50/50 (v/v) acetonitrile/water into the mass spectrometer (4.0 s, 0.5 ml min^{-1}), and the SPE cartridge re-equilibrated with 0.1% (v/v) aqueous formic acid in 85/15 (v/v) acetonitrile/water (0.5 s, 1.0 ml min^{-1}). The mass spectrometer was operated using MassHunter Workstation B.08.00 software (Agilent), with parameters: capillary voltage (3500 V), nozzle voltage (2000 V), fragmentor voltage (175 V), gas temperature (280 °C), gas flow (11 l min−1), sheath gas temperature (350 °C), sheath gas flow (11 l min^{-1}), nebulizer pressure (35 psig). The m/z +1 charge states of the ACV substrate, IPN product or hydrated aldehyde were used to extract ion chromatogram data, peak areas were integrated using RapidFire Integrator 4.3.0 (Agilent). Data were exported into Microsoft Excel and used to calculate the percentage conversion for IPNS variant reaction.

Protein mass spectrometry on $IPNS^{H270E}$ *in crystallo* and in solution

MS analysis of $IPNS^{H270E}$ (100 μM, 50 μL) enzyme in solution was performed after addition of $(NH_4)_2Fe(SO_4)_2$ (1 eq., 100 μM in H_2O) and ACV (5 eq., 500 μM) in MOPS buffer (100 mM in H_2O, pH 7.2) under ambient conditions for defined times. Reactions were initiated by addition of the $(NH_4)_2Fe(SO_4)_2$ solution and prior to injection diluted to a final enzyme concentration of 5 μM. Mass spectrometry on crystals were performed after exposing crystal containing solution (crystallisation described above) to O_2 saturated crystallisation solution, followed by harvesting multiple crystals (>9) and dissolving in H_2O. Samples were analysed using a Xevo G2-S mass spectrometer (Waters) coupled to an Acquity-UPLC system (Waters), equipped with a ProSwift RP-4H 1-mm x 50-mm column (Thermo Fisher Scientific), loaded onto the column in 95% (v/v) water, 5% (v/v) acetonitrile, and 0.1% (v/v) formic acid. Samples were eluted using a gradient to 5% (v/v) water, 95% (v/v) acetonitrile, and 0.1% (v/v) formic acid over 8 min. Data were analysed using MassLynx4.1, with deconvolution using the MaxEnt1 algorithm and data was shown in Supplementary Fig 7m.

Entry	Complex	Description	PDB code	Facility	Experiment	Delivery system	Resolution (Å)	O_2 incubation time (ms)	Total time (ms)	Crystal lattices	Crystal Batch
1	IPNS:Fe:ACdV	-	8PY6	LCLS	LV56	ADE tape drive	1.52	0	0	10327	1
2	IPNS:Fe:ACdV:O_2	1 s O_2	8PY7	LCLS	LV56	ADE tape drive	1.49	400	1000	26959	1
3	IPNS:Fe:ACdV:O_2	2 s O_2	8PY8	LCLS	LV56	ADE tape drive	1.45	800	2000	26959	1
4	IPNS:Fe:ACdV/TAL:O_2	3 s O_2	8PY9	LCLS	LV56	ADE tape drive	1.50	1200	3000	19985	1
5	IPNS:Fe:ACdV/TAL:O_2	4 s O_2	8PYA	LCLS	LV56	ADE tape drive	1.50	1600	4000	23110	1
6	IPNS:Fe:TAL:O_2	6 s O_2	9I3A	LCLS	LV56	ADE tape drive	1.52	2400	6000	24325	1
7	IPNS:Fe/Fe-O:TAL/MCI	8 s O_2	9I3B	LCLS	LV56	ADE tape drive	1.60	3200	8000	12115	1
8	IPNS:Fe/Fe-O:TAL/MCI	10s O_2	9I3C	LCLS	LV56	ADE tape drive	1.70	4000	10000	38451	1
9	IPNS:Fe:IPN (from AcdV)	24h O_2	9QPH	LCLS	P-10035	ADE tape drive	1.70	-	24 h	3667	2
10	IPNS:Fe:ACdV	-	8PY5	PALFEL	2019-2nd-NIC-029	ADE tape drive	1.85	0	0	9118	3
11	IPNS:Fe/Fe-O: MCI/IPN	10s O_2	9I3D	PALFEL	2019-2nd-NIC-029	ADE tape drive	1.68	4000	10000	12925	3
12	IPNS:VO:ACV	-	6ZAK	SACLA	2019A8088	ADE tape drive	1.54	-	-	5964	4
13	IPNS:Fe:ACdV	-	9QPI	LCLS	P-10035	ADE tape drive	1.45	0	0	12175	2
14	IPNS:Fe:IPN (from AcdV)	24h O_2	9QPJ	DLS	mx32727	Fixed target SSX	1.90	-	24 h	6159	2
15	IPNS_N252D:Fe:ACV	-	8P48	DLS	-	Cryo-MX	1.63	-	-	1	-
16	IPNS_N252D:Fe:IPN	30 min	8P46	DLS	-	Cryo-MX	1.77	-	10 min	1	-
17	IPNS_N252A:Fe:ACV	-	8P47	DLS	-	Cryo-MX	1.56	-	-	1	-
18	IPNS_N252Q:Fe:ACV	-	8PTV	DLS	-	Cryo-MX	1.60	-	-	1	-
19	IPNS_N252E:Fe:ACV	-	8RDB	DLS	-	Cryo-MX	1.37	-	-	1	-
20	IPNS_N252E:Fe:IPN	30 min	9HWF	DLS	-	Cryo-MX	1.40	-	60 min	1	-
21	IPNS_H270E:Fe:ACV	-	7ZOE	DLS	-	Cryo-MX	1.50	-	-	1	-
22	IPNS_H270E:Cd:ACV	-	8A4G	DLS	-	Cryo-MX	1.97	-	-	1	-
23	IPNS_H270E:Fe:ACV	30 min	8ALI	DLS	-	Cryo-MX	1.60	-	30 min	1	-
24	IPNS_H270E:Fe:ACV	3 h	8ALJ	DLS	-	Cryo-MX	1.68	-	3 h	1	-
25	IPNS:Fe:IPN (from AcdV)	18h O_2	9QPG	DLS	-	Cryo-MX	1.79	-	18 h	1	-

Supplementary Table 2: Overview of reported crystal structures obtained from rotation-based single crystal cryo data collection and from room temperature serial data collection using the ADE tape drive with acoustic droplet ejection,[4] or room temperature fixed target methods.[5,6]

Supplementary Table 3: Data collection and Refinement statistics.

**Datasets**	**IPNS:Fe:ACV[a]** (PDB ID: 8PY6)	**IPNS:Fe:ACV:O_2 (1 s)[a]** (PDB ID: 8PY7)	**IPNS:Fe:ACV:O_2 (2 s)[a]** (PDB ID: 8PY8)	**IPNS:Fe:ACV/TAL:O_2 (3 s)[a]** (PDB ID: 8PY9)	**IPNS:Fe:ACV/TAL:O_2 (4 s)[a]** (PDB ID: 8PYA)
**Data Collection (T in K)**	SFX (298)	SFX (293)	SFX (298)	SFX (298)	SFX (298)
Beamline (Wavelength, Å)	SLAC LCLS MFX (1.300289)	SLAC LCLS MFX (1.300289)	SLAC LCLS MFX (1.300289)	SLAC LCLS MFX (1.300289)	SLAC LCLS MFX (1.300289)
Detector	RAYONIX MX340-HS	RAYONIX MX340-HS	RAYONIX MX340-HS	RAYONIX MX340-HS	RAYONIX MX340-HS
Data Processing	cctbx.xfel	cctbx.xfel	cctbx.xfel	cctbx.xfel	cctbx.xfel
Space group	$P\ 2_1\ 2_1\ 2_1$	$P\ 2_1\ 2_1\ 2_1$	$P\ 2_1\ 2_1\ 2_1$	$P\ 2_1\ 2_1\ 2_1$	$P\ 2_1\ 2_1\ 2_1$
Cell dimensions					
a,b,c (Å)	41.6, 75.6, 101.7	41.8, 75.4, 101.8	41.7, 75.2, 101.7	41.7, 75.2, 101.7	41.7, 75.2, 101.7
α, β, γ (°)	90, 90, 90	90, 90, 90	90, 90, 90	90, 90, 90	90, 90, 90
No. of molecules/ASU	1	1	1	1	1
No. reflections	50128 (2499)*	53297 (2612*	57610 (2840)	52127 (2555)*	52007 (2527)*
Resolution (Å)	22.58-1.52 (1.55-1.52)*	22.53-1.49 (1.52-1.49)*	22.49-1.45 (1.48-1.45)*	22.50-1.50 (1.53-1.50)*	22.48-1.50 (1.53-1.50)*
R_{split}	0.097 (0.811)*	0.067 (0.861)*	0.087 (1.257)*	0.087 (0.982)*	0.073 (0.866)*
I/σI	4.465 (0.590)	6.379 (0.581)	4.70 (0.110)*	5.122 (0.484)*	5.986 (0.578)*
CC-half	0.992 (0.394)	0.997 (0.329)	0.995 (0.135)*	0.995 (0.242)*	0.996 (0.353)*
Completeness (%)	99.95 (99.88)*	99.95 (99.85)*	99.6 (100)*	99.95 (99.84)*	99.94 (99.61)*
Multiplicity	88.16 (11.96)*	186.07 (12.31)*	153.09 (55.54)*	117.98 (12.71)*	161.62 (12.63)*
Wilson B value ($Å^2$)	20.00	19.04	23.28	20.48	19.82
**Refinement**	PHENIX	PHENIX	PHENIX	PHENIX	PHENIX
R_{work}/R_{free+}	0.1558/0.1720	0.1492 /0.1791	0.1655/0.1911	0.1783/0.2087	0.1807/0.2068
No. atoms	2900	2932	2898	2926	2915
- Enzyme	2659	2680	2658	2685	2670
- ligand	35	45	58	58	57
- Water	206	207	182	183	188
Average B-factors	26.97	27.24	30.32	26.68	26.74
- Enzyme	26.08	26.17	29.52	25.73	25.87
- ligand	38.77	30.98	38.03	38.96	35.45
- Water	36.44	40.28	39.57	36.67	36.36
R.m.s deviations					
- Bond lengths (Å)	0.004	0.007	0.005	0.008	0.014
- Bond angles (°)	0.74	0.99	0.81	0.98	1.06

*Highest resolution shell in parentheses. DLS = Diamond Light Source. a = numbers of lattices merged for each serial room temperature data set is given in Table S2.

Supplementary Table 3 (continued): Data collection and Refinement statistics.

Datasets	IPNS:Fe:TAL:O_2 (6 s)[a] (PDB ID: 9I3A)	IPNS:Fe/Fe-O:TAL/MCI (8s)[a] (PDB ID: 9I3B)	IPNS:Fe-O:TAL/MCI (10 s)[a] (PDB ID: 9I3C)	IPNS:Fe:IPN (24 h)[a] (PDB ID: 9QPH)	IPNS:VO:ACV[a] (PDB ID: 6ZAK)
**Data Collection (T in K)**	SFX (298)	SFX (298)	SFX (298)	SFX (298)	tr-SFX (298)
Beamline (Wavelength, Å)	SLAC LCLS MFX (1.300289)	SLAC LCLS MFX (1.300289)	SLAC LCLS MFX (1.300289)	SLAC LCLS MFX (1.282235)	SACLA BL2 (1.23997)
Detector	RAYONIX MX340-HS	RAYONIX MX340-HS	RAYONIX MX340-HS	RAYONIX MX340-HS	RAYONIX MX300-HS
Data Processing	cctbx.xfel	cctbx.xfel	cctbx.xfel	cctbx.xfel	cctbx.xfel
Space group	$P\ 2_1\ 2_1\ 2_1$	$P\ 2_1\ 2_1\ 2_1$	$P\ 2_1\ 2_1\ 2_1$	$P\ 2_1\ 2_1\ 2_1$	$P\ 2_1\ 2_1\ 2_1$
Cell dimensions					
a,b,c (Å)	41.7, 75.2, 101.7	41.6, 75.0, 101.6	41.6, 75.0, 101.6	41.9, 75.1, 101.8	41.8, 75.6, 101.7
α, β, γ (º)	90, 90, 90	90, 90, 90	90, 90, 90	90, 90, 90	90, 90, 90
No. of molecules/ASU	1	1	1	1	1
No. reflections	51986 (2562)*	42762 (2030)*	35807 (1792)*	35802 (1746)*	48446 (2360)*
Resolution (Å)	22.48-1.50 (1.52-1.50)*	22.45-1.60 (1.64-1.60)*	22.44-1.70 (1.73-1.70)*	22.46-1.70 (1.72-1.70)*	34.41-1.54 (1.566-1.54)*
R_{split}	0.069 (0.869)*	0.102 (0.784)*	0.087 (0.454)*	0.238 (0.998)*	0.239 (1.269)*
I/σI	6.178 (0.552)*	4.231 (0.501)*	5.699 (0.980)*	2.407 (0.428)*	32.94 (0.706)*
CC-half	0.996 (0.320)*	0.985 (0.048)*	0.992 (0.411)*	0.941 (0.292)*	0.936 (0.026)*
Completeness (%)	99.95 (99.84)*	99.95 (100)*	99.94 (100)*	100 (99.9) *	99.98 (99.92)*
Multiplicity	186.32 (13.42)*	107.19 (19.02)*	172.49 (54.99)*	22.40 (14.85)*	35.4 (11.56)*
Wilson B value ($Å^2$)	20.01	21.69	21.84	23.86	20.66
**Refinement**	PHENIX	PHENIX	PHENIX	PHENIX	PHENIX
R_{work}/R_{free+}	0.1618/0.1889	0.1667/0.1916	0.1564 /0.1900	0.1887 /0.2171	0.1737/0.2008
No. atoms	3147	3044	3105	2792	2858
- Enzyme	2891	2811	2870	2609	2629
- ligand	62	39	39	30	31
- Water	194	194	196	153	198
Average B-factors	28.34	29.49	27.86	32.22	27.51
- Enzyme	27.49	28.42	26.91	31.80	26.74
- ligand	35.04	44.39	41.62	41.14	30.08
- Water	38.48	41.19	38.28	37.56	37.28
R.m.s deviations					
- Bond lengths (Å)	0.010	0.008	0.010	0.005	0.011
- Bond angles (º)	1.04	0.91	1.03	0.67	0.99

*Highest resolution shell in parentheses. DLS = Diamond Light Source. a = numbers of lattices merged for serial room temperature data set is given in Table S2.

Supplementary Table 3 (continued): Data collection and Refinement statistics.

Datasets	IPNS_N252D:Fe:ACV[a] (PDB ID: 8P48)	IPNS_N252A:Fe:ACV[a] (PDB ID: 8P47)	IPNS_N252D:Fe:IPN[a] (PDB ID: 8P46)	IPNS:Fe-O: MCI/IPN (10 s)[a] (PDB ID: 9I3D)	IPNS_H270E:Fe:ACV (anaerobic) (PDB ID: 7ZOE)
**Data Collection (T in K)**	cryo-MX (100)	cryo-MX (100)	cryo-MX (100)	tr-SFX (298)	cryo-MX (100)
Beamline (Wavelength, Å)	DLS I03 (0.9763)	DLS I03 (0.9763)	DLS I03 (0.9763)	PALFEL (1.318066)	DLS I03 (0.9763)
Detector	EIGER2 XE 16M	EIGER2 XE 16M	EIGER2 XE 16M	RAYONIX MX225-HS	Eiger2 XE 16M
Data Processing	Xia2	Xia2	Xia2	cctbx.xfel	Xia2
Space group	$P\,2_1\,2_1\,2_1$	$P\,2_1\,2_1\,2_1$	$P\,2_1\,2_1\,2_1$	$P\,2_1\,2_1\,2_1$	$P\,2_1\,2_1\,2_1$
Cell dimensions					
a,b,c (Å)	41.2, 74.8, 101.2	41.7, 74.7, 101.5	41.1, 74.1, 101.0	41.5, 74.8, 101.4	41.4, 71.7, 101.4
α, β, γ (º)	90, 90, 90	90, 90, 90	90, 90, 90	90, 90, 90	90, 90, 90
No. of molecules/ASU	1	1	1	1	1
No. reflections	39910 (2869)*	45988 (2254)*	30908 (1506)*	36772 (1807)*	51282 (2540)*
Resolution (Å)	50.58-1.63 (1.67-1.63)*	50.72-1.56 (1.59-1.56)*	50.48 - 1.77 (1.80-1.77)*	27.28-1.68 (1.71-1.68)*	60.15-1.50 (1.53-1.50)*
R_{split}	0.061 (3.359)*	0.219 (2.544)*	0.285 (2.892)*	0.173 (1.15)*	0.171 (1.571)*
I/σI	9.2 (0.9)*	8.2 (0.6)*	5.9 (0.6)*	3.921 (0.610)	10.8 (1.20)*
CC-half	0.998 (0.545)*	0.997 (0.536)*	0.995 (0.448)*	0.984 (0.096)*	0.998 (0.66)*
Completeness (%)	99.9 (99.9)*	100 (100)*	100 (100)*	99.95 (99.72)*	100 (99.9)*
Multiplicity	13.4 (13.3)*	13.5 (13.9)*	13.3 (13.5)*	93.75 (11.04)*	13.40 (13.70)*
Wilson B value ($Å^2$)	19.12	17.97	21.79	21.82	14.62
**Refinement**	PHENIX	PHENIX	PHENIX	PHENIX	PHENIX
R_{work}/R_{free+}	0.1793/0.2034	0.1812/0.2092	0.1844/0.2245	0.2108 /0.2589	0.1565/0.1867
No. atoms	3085	3155	2963	2905	3197
- Enzyme	2663	2688	2621	2629	2699
- ligand	40	40	45	68	35
- Water	382	427	297	208	463
Average B-factors	24.77	22.93	24.63	26.22	18.94
- Enzyme	23.64	21.55	23.63	25.65	17.14
- ligand	32.76	31.43	39.94	29.43	21.50
- Water	31.88	30.85	31.13	32.54	29.25
R.m.s deviations					
- Bond lengths (Å)	0.004	0.004	0.007	0.006	0.006
- Bond angles (º)	0.66	0.62	0.82	0.75	0.79

*Highest resolution shell in parentheses. DLS = Diamond Light Source. a = numbers of lattices merged for each serial room temperature data set is given in Table S2.

Supplementary Table 3 (continued): Data collection and Refinement statistics.

**Datasets**	**IPNS_H270E:Cd:ACV[a]** (PDB ID: 8A4G)	**IPNS_H270E:Fe:ACV (O$_2$ exposure 30 min)** (PDB ID: 8ALI)	**IPNS_H270E:Fe:ACV (O$_2$ exposure 3 h)** (PDB ID: 8ALJ)	**IPNS_N252Q:Fe:ACV[a]** (PDB ID: 8PTV)	**IPNS:Fe:ACV[a]** (PDB ID: 8PY5)
**Data Collection (T in K)**	MX (100)	MX (100)	MX (100)	cryo-MX (100)	tr-SFX (298)
Beamline (Wavelength, Å)	DLS I03 (0.9763)	DLS I03 (0.9763)	DLS I03 (0.9763)	DLS I04 (0.9537)	PALFEL (1.318066)
Detector	Eiger2 XE 16M	Eiger2 XE 16M	Eiger2 XE 16M	EIGER2 XE 16M	RAYONIX MX225-HS
Data Processing	Xia2	Xia2	Xia2	Xia2	cctbx.xfel
Space group	$P\ 2_1\ 2_1\ 2_1$	$P\ 2_1\ 2_1\ 2_1$	$P\ 2_1\ 2_1\ 2_1$	$P\ 2_1\ 2_1\ 2_1$	$P\ 2_1\ 2_1\ 2_1$
Cell dimensions					
a,b,c (Å)	41.1, 74.1, 101.3	41.1, 74.3, 101.0	41.2, 74.5, 101.2	40.9, 74.3, 100.8	41.7, 75.3, 101.7
α, β, γ (°)	90, 90, 90	90, 90, 90	90, 90, 90	90, 90, 90	90, 90,120
No. of molecules/ASU	1	1	1	1	1
No. reflections	22498 (1602)*	41690 (2041)*	36264 (1788)*	41326 (3001)*	28093 (1381)*
Resolution (Å)	41.8-1.97 (2.02-1.97)*	59.86-1.60 (1.63-1.60)*	59.98-1.68 (1.72-1.68)*	50.38 - 1.60 (1.64-1.60)*	27.94-1.85 (1.88-1.85)*
R_{meas}(I)/ R_{split}	0.289 (2.693)*	0.174 (2.175)*	0.277 (2.936)*	0.163 (3.373)*	0.239 (1.166)*
I/σI	7.2 (1.0)*	9.5 (1.20)*	7.6 (0.65)*	9.9 (0.7)*	3.493 (0.578)*
CC-half	0.996 (0.419)*	0.999 (0.673)*	0.996 (0.476)*	0.999 (0.535)*	0.964 (0.09)*
Completeness (%)	99.8 (99.3)*	100 (99.9)*	100 (100)*	99.9 (100)*	99.96 (100)*
Multiplicity	12.9 (12.1)*	13.50 (11.10)*	13.40 (13.70)*	13.3 (12.6)*	67.58 (16.25)*
Wilson B value (Å^2)	29.65	18.08	19.96	22.4	23.14
**Refinement**	PHENIX	PHENIX	PHENIX	PHENIX	PHENIX
R_{work}/R_{free+}	0.2169/0.2539	0.1935/0.2153	0.1804/0.2299	0.1994/0.2320	0.2135/0.2457
No. atoms	2928	3058	3063	2986	2846
- Enzyme	2673	2682	2662	2617	2633
- ligand	52	69	64	65	35
- Water	203	307	337	304	178
Average B-factors	30.95	25.05	24.88	28.43	26.46
- Enzyme	30.67	24.19	23.95	27.22	26.12
- ligand	37.56	33.32	32.14	42.55	30.11
- Water	32.97	30.65	30.88	35.85	30.72
R.m.s deviations					
- Bond lengths (Å)	0.002	0.004	0.014	0.003	0.003
- Bond angles (°)	0.48	0.69	1.03	0.60	0.57

*Highest resolution shell in parentheses. DLS = Diamond Light Source. a = numbers of lattices merged for each serial room temperature data set is given in Table S2.

Supplementary Table 3 (continued): Data collection and Refinement statistics.

**Datasets**	**IPNS_N252E:Fe:ACV[a]** (PDB ID: 8RDB)	**IPNS_N252E:Fe:IPN** (PDB ID: 9HWF)	**IPNS:Fe:IPN (24h O_2)[a]** (PDB ID: 9QPJ)	**IPNS:Fe:IPN (18h O_2)[a]** (PDB ID: 9QPG)	**IPNS:Fe:ACdV (anaerobic)[a]** (PDB ID: 9QPI)
**Data Collection (T in K)**	cryo-MX (100)	cryo-MX (100)	SSX (298)	cryo-MX (100)	SFX (298)
Beamline (Wavelength, Å)	DLS I03 (0.9763)	DLS I03 (0.9762)	DLS I24 (0.999850)	DLS I03 (0.9763)	SLAC LCLS MFX (1.282235)
Detector	EIGER2 XE 16M	EIGER2 XE 16M	DECTRIS PILATUS3 6M	EIGER2 XE 16M	RAYONIX MX340-HS
Data Processing	Xia2	Xia2	cctbx.xfel	Xia2	cctbx.xfel
Space group	$P\ 2_1\ 2_1\ 2_1$	$P\ 2_1\ 2_1\ 2_1$	$P\ 2_1\ 2_1\ 2_1$	$P\ 2_1\ 2_1\ 2_1$	$P\ 2_1\ 2_1\ 2_1$
Cell dimensions					
a,b,c (Å)	41.3, 74.6, 101.1	41.3, 74.4, 101.2	41.9, 75.2, 101.8	41.0, 74.1, 100.8	41.8, 75.5, 101.8
α, β, γ (º)	90, 90, 90	90, 90, 90	90, 90, 90	90, 90, 90	90, 90, 90
No. of molecules/ASU	1	1	1	1	1
No. reflections	66337 (4804)*	61769 (3024)*	26112 (1258)*	29287 (1448)*	57981 (2827)*
Resolution (Å)	41.83-1.37 (1.41-1.37)*	41.87-1.40 (1.42-1.40)*	60.51 -1.90 (1.93-1.90)*	59.70-1.79 (1.82-1.79)*	22.57 -1.45 (1.48-1.45)*
R_{merge}/R_{split}	0.122 (2.699)*	0.144 (2.153)*	0.261 (0.957)*	0.190 (2.091)*	0.120 (0.795)*
I/σI	12.5 (1.0)*	11.6 (0.8)*	2.003 (0.572)*	7.4 (0.6)*	5.500 (1.064)*
CC-half	0.999 (0.554)*	0.999 (0.569)*	0.931 (0.242)*	0.997 (0.574)*	0.99 (0.39)*
Completeness (%)	99.9 (99.1)*	100 (100)*	99.96 (100)*	98.7 (99.9)*	99.96 (99.93)*
Multiplicity	13.4 (13.0)*	13.6 (13.8)*	31.61 (22.78)*	13.60 (14.0)*	52.48 (12.31)*
Wilson B value ($Å^2$)	15.32	14.86	29.96	24.88	16.98
**Refinement**	PHENIX	PHENIX	PHENIX	PHENIX	PHENIX
R_{work}/R_{free+}	0.1548/0.19404	0.1681/0.2027	0.2037 /0.2381	0.1828 /0.2316	0.1525 /0.1758
No. atoms	3128	3182	2758	2943	2972
- Enzyme	2681	2704	2632	2638	2710
- ligand	45	40	30	40	35
- Water	402	438	96	265	227
Average B-factors	20.77	19.45	36.05	29.00	24.46
- Enzyme	19.00	17.45	35.85	28.10	23.11
- ligand	35.42	34.34	50.53	48.28	43.09
- Water	30.90	30.49	37.11	35.05	37.77
R.m.s deviations					
- Bond lengths (Å)	0.013	0.003	0.002	0.013	0.001
- Bond angles (º)	1.17	0.62	0.50	1.16	1.05

*Highest resolution shell in parentheses. DLS = Diamond Light Source. a = numbers of lattices merged for each serial room temperature data set is given in Table S2.

General synthetic procedures

Unless otherwise stated, reagents were from commercial sources (Sigma-Aldrich, Fluorochem Ltd) and used as received. Anhydrous solvents were from Acros Organics and Sigma-Aldrich and kept under nitrogen. Solvents, liquids, and solutions were transferred using nitrogen-flushed stainless-steel needles and syringes. Milli-Q® Ultrapure (MQ-grade) water was used for buffers. HPLC grade solvents (Sigma-Aldrich Inc.) were used for work-ups, extractions, and purifications. Purifications were performed using a Biotage Isolera One purification machine or a Biotage Selekt purification machine (wavelengths monitored: 254 and 280 nm) equipped with pre-packed Biotage® Sfär Duo flash chromatography cartridges. The cartridge type and size and solvent gradients (in column volumes, CV) used, are specified in individual procedures.

Thin layer chromatography (TLC) used Merck silica gel 60 F254 TLC plates and visualized under UV light. Infrared (IR) spectroscopy was performed using a Bruker Tensor-27 Fourier transform infrared (FT-IR) spectrometer. High-resolution MS (HRMS) was performed using electrospray ionization (ESI) mass spectrometry (MS) in the positive or negative ionization mode employing a Thermo Scientific Exactive mass spectrometer (ThermoFisher Scientific) or a Xevo G2-XS mass spectrometer (Waters, UK); data are presented as a mass-to-charge ratio (m/z).

Nuclear magnetic resonance (NMR) spectroscopy was performed using Bruker Avance III 400 HD nanobay NMR machine equipped with a 9.4T magnet or Bruker Avance III 700 with a $^{1}H(^{13}C/^{15}N)$ inverse TCI cryoprobe equipped with a 16.44T magnet. Chemical shifts for ^{1}H NMR are reported in parts per million (ppm) downfield from tetramethylsilane and are referenced to the residual protium in the NMR solvent (DMSO-d_6: δ = 2.50 ppm; D_2O: δ = 4.79 ppm). For ^{13}C NMR, chemical shifts are reported in the scale relative to the NMR solvent (DMSO-d_6: δ = 39.52 ppm). For ^{19}F NMR, chemical shifts are reported in the scale relative to $CFCl_3$. NMR data are reported as follows: chemical shift, multiplicity (s: singlet, d: doublet, t: triplet, p: pentet (quintet) h: heptet, m: multiplet, br: broad signal), coupling constant (*J*, Hz; accurate to 0.1 Hz), and integration. NMR spectra are shown below.

Synthesis of labelled ACV

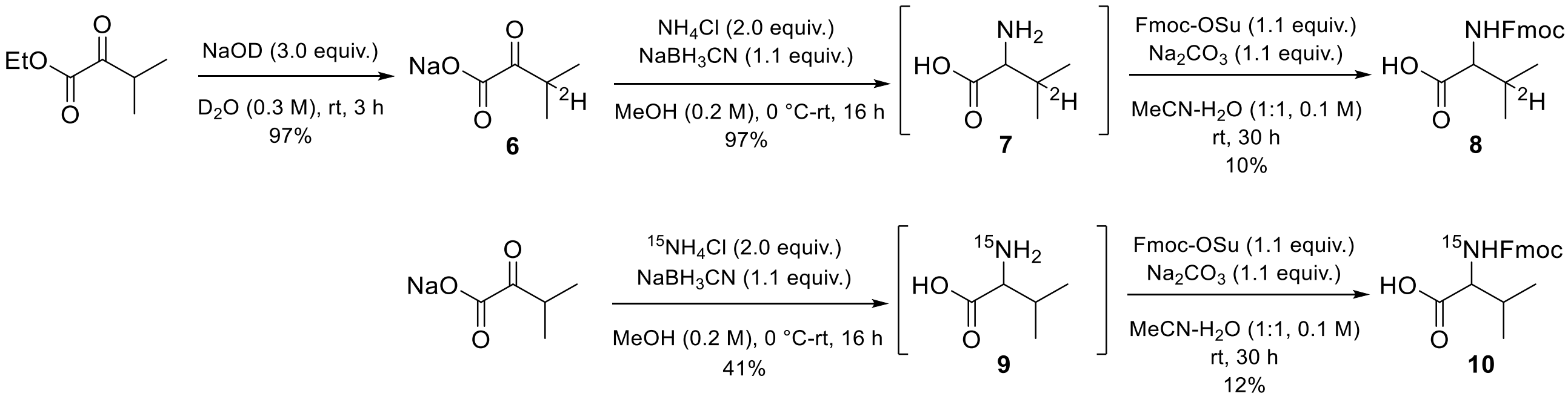

Supplementary Figure 1. Synthesis of ^{2}H- and ^{15}N-labelled valine.

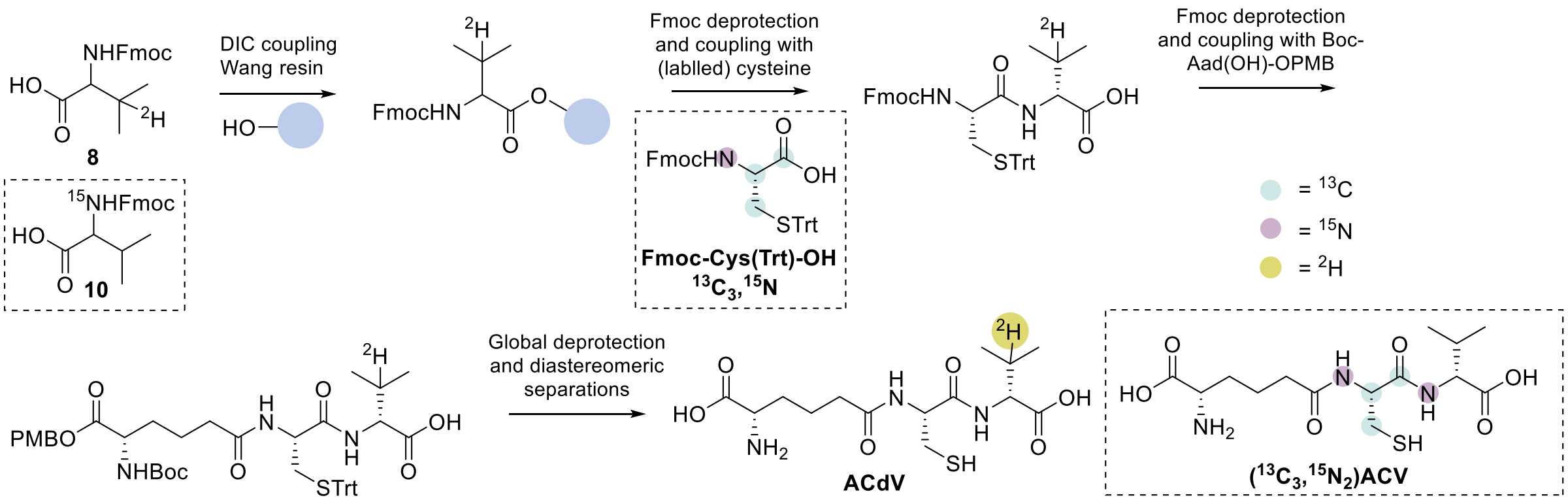

Supplementary Figure 2. General synthetic route for solid phase peptide synthesis to produce labelled ACdV and (^{13}C$_3$,^{15}N$_2$)ACV. For (^{13}C$_3$,^{15}N$_2$)ACV, the compounds in the dashed box were used.

General method for reductive amination[7]

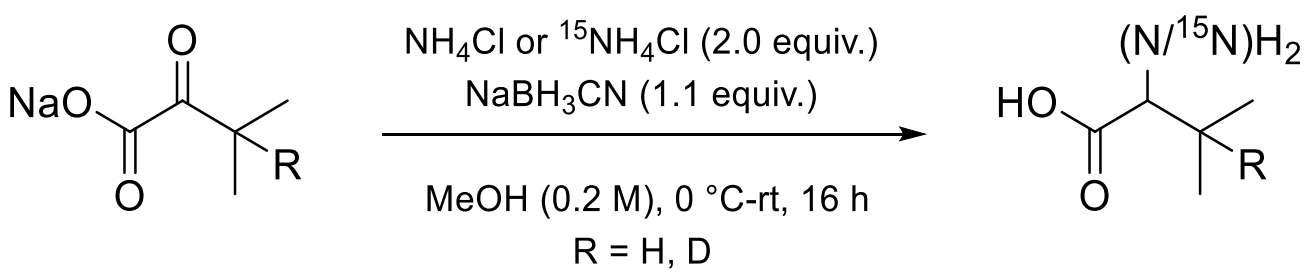

A round bottom flask equipped with magnetic stirrer bar was charged with sodium 3-methyl-2-oxobutanoate or sodium 3-methyl-2-oxobutanoate-3-*d* (1.0 equiv.) and MeOH (0.2 M). To this suspension was added NH_4Cl or $^{15}NH_4Cl$ (2.0 equiv.), then stirred for 15 minutes at 0 °C. $NaBH_3CN$ (1.1 equiv.) was then added at 0 °C; the reaction mixture was allowed to warm to rt and left to stir overnight. Then conc. HCl (5.0 equiv.) was added dropwise with vigorous stirring. The solvent was removed *in vacuo*. The crude white solid was suspended in EtOH (10 mL/mmol) and filtered. The filtrate was concentrated and dried *in vacuo* to obtain a white crude solid containing different combination of labelled valine, which was directly used in the next step.

General method for Fmoc protection

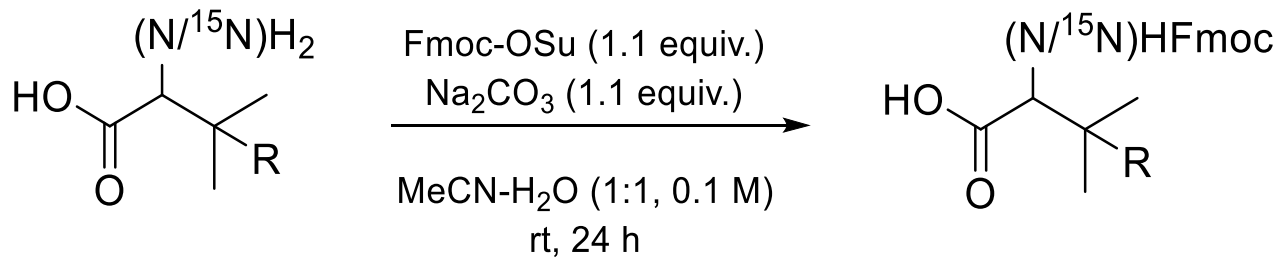

A round bottom flask equipped with magnetic stirrer bar was charged with crude labelled or unlabelled valine (1.0 equiv.) and MeCN-H_2O (0.1 M). To this suspension was added Na_2CO_3 (1.1 equiv.) and Fmoc-OSu (1.1 eq.) sequentially and left stirring at rt for 24 h. The reaction was quenched by the addition of 2 M HCl (2.0 equiv.), and the aqueous layer was extracted with EtOAc (3 times). The combined organic extracts were dried over $MgSO_4$, filtered, then concentrated *in vacuo*. The crude product was purified by column chromatography.

General method for solid phase peptide coupling:

All labelled trimer peptides were prepared by solid phase peptide synthesis using a Liberty Blue - Automated Microwave Peptide Synthesizer (CEM, US).[1] Couplings of amino acids were done sequentially starting from the *C*-terminus, using a manual loading step to load the labelled Fmoc-Val on Wang resin (Sigma-Aldrich), Conditions: Wang resin (1.0 eq, 1.1 mmol/g), Fmoc-Val (1.1 eq.), HOBt (1.5 eq), *N,N′*-diisopropylcarbodiimide (DIC, 1.5 eq.) and DMAP (0.1 eq) in CH_2Cl_2:DMF (5:1, 5mL for 0.5 mM reaction scale, stirred at room temperature overnight).The resin was filtered and washed with MeOH and CH_2Cl_2. Fmoc-L-Cys(Trt)-OH (Sigma-Aldrich) or Fmoc-L-Cys(Trt)-OH,$^{13}C_3$.^{15}N (Sigma-Aldrich) and freshly prepared Boc-Aad(OH)OPMB was used to extend the chain.[1] A typical batch was prepared with 0.50 mmol of resin. Deprotection of the *N*-terminal Fmoc group employed piperidine in DMF (20% v/v). Coupling of Fmoc-l-Cys(Trt)-OH (1.46 g, 2.50 mmol, 5 eq.) in presence of DIC (0.25 mL, 0.50 M, 5 eq.) in DMF, and oxyma[8] (0.250 mL, 1.00 M, 5 eq.) in DMF supplemented with *N,N*-diisopropyldiethylamine (DIPEA, 1.9% v/v, 110 mM, 0.55 eq.), reacted for 10 minutes at 90 °C, followed by piperidine deprotection. Coupling of Boc-Aad(OH)OPMB (382 mg, 1.00 mmol, 2 eq.) was done using DIC (0.250 mL, 1.00 M, 5 eq.), ethyl cyanohydroxyiminoacetate (oxyma, 0.250 mL, 1.00 M, 5 eq.) (20 min, 90 °C). The resin was washed with CH_2Cl_2 and MeOH. After drying on vacuum the resin was treated with a cleavage cocktail (for 0.5 mmol: 2,2,2-trifluoroacetic acid (TFA, 7.125 mL), triisopropylsilane (TIPS, 187.5 µL), H_2O (187.5 µL), 2,2′-(ethylenedioxy)diethanethiol (1.5 mL)) and stirred for 16 h. The solvent was removed under reduced pressure, co-evaporating with CH_2Cl_2. The tripeptide was precipitated using cold Et_2O (40 mL, --80 °C). After centrifugation (4,000 rpm, 2 min, 4 °C) the Et_2O was decanted and the resultant pellet dried under a stream of N_2 (g). The Et_2O layer was evaporated and the precipitation process repeated. The combined crude peptides were purified by semi preparative-HPLC to separate stereoisomers.

Sodium 3-methyl-2-oxobutanoate-3-^{2}*H* (6) [7]

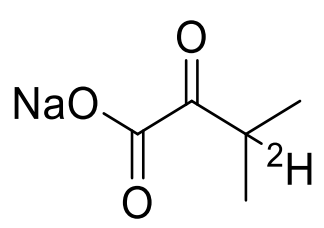

A 250 mL round bottom flask equipped with magnetic stirrer bar was charged with ethyl 3-methyl-2-oxobutanoate (2.88 g, 20.0 mmol) and D_2O (60 mL). To this mixture was added NaOD in D_2O (40% w/v, 2.92 mL, 60.0 mmol). The mixture was stirred at rt for 3 h, then washed with Et_2O (20 mL x 3), then acidified with DCl (35%) to pH 5. The mixture was dried in a lyophilizer to give **6** of 49% purity (determined by quantitative NMR using 3-(trimethylsilyl)-2,2,3,3-tetradeuteropropionic acid as internal standard) as white powder (5.48 g, 18.6 mmol, 97%), which was directly used in the next step; ^{1}H NMR (600 MHz, 300 K, D_2O) δ = 1.15 (s, 6H) ppm; ^{13}C NMR (151 MHz, 300 K, D_2O) δ = 214.4, 175.1, 39.5 (t, *J* = 19.7 Hz), 19.2 ppm. Spectroscopic data were in accord with those reported.[9]

Valine-3-^{2}*H* (7)[7]

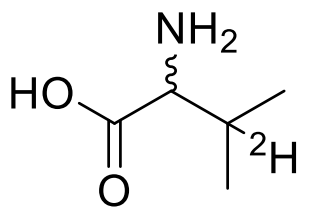

The General Method for reductive amination was followed using **6** (49 wt%, 1.42 g, 5.00 mmol) and NH_4Cl (535 mg, 53.5 mmol) to obtain **7** of 11% purity (determined by quantitative NMR using 3-(trimethylsilyl)-2,2,3,3-tetradeuteropropionic acid as internal standard) as a white solid (2.42 g, 2.25 mmol, 45%), which was directly used in the next step; ^{1}H NMR (500 MHz, 300 K, D_2O) δ = 3.87 (s, 1H), 1.10 (s, 3H), 1.07 (s, 3H) ppm; ^{13}C NMR (126 MHz, 300 K, D_2O) δ = 175.7, 62.1 (d, *J* = 3.12 Hz), 31.6 (t, *J* = 20.1 Hz), 20.5, 19.6 ppm. Spectroscopic data were in accord with those reported.[9]

(((9H-Fluoren-9-yl)methoxy)carbonyl)valine-3-^{2}*H* (8)[7]

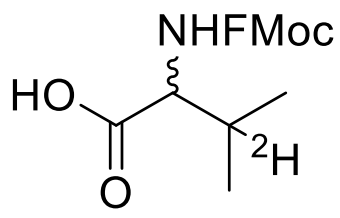

The General Method for Fmoc protection was followed using valine-3-*d* (**7**, 11wt%, 1.41 g, 1.33 mmol) following a flash column chromatography method (50 g Sfär Duo Silica; 120 mL/min using 100% (v/v) CH_2Cl_2 (+ 0.1% (v/v) formic acid) (2 CV), followed by a linear gradient (30 CV): 0%→100% (v/v) (20% (v/v) EtOAc in CH_2Cl_2 (+ 0.1% (v/v) formic acid)) in CH_2Cl_2 (+ 0.1% (v/v) formic acid); eluted at 20%) to obtain the desired compound **8** as a white solid (42 mg, 0.13 mmol, 10%); mp 140 – 142 °C; ^{1}H NMR (600 MHz, DMSO-d_6) δ = 12.58 (brs, 1H), 7.89 (d, *J* = 7.6 Hz, 2H), 7.75 (dd, *J* = 7.6, 2.9 Hz, 2H), 7.59 (d, *J* = 8.7 Hz, 1H), 7.42 (td, *J* = 7.5, 1.1 Hz, 2H), 7.33 (tdd, *J* = 7.4, 3.3, 1.2 Hz, 2H), 4.32 – 4.18 (m, 3H), 3.87 (d, *J* = 8.7 Hz, 1H), 0.90 (s, 6H) ppm; ^{13}C NMR (151 MHz, DMSO-d_6) δ 173.3, 156.4, 143.9, 143.8, 140.7, 140.7, 127.6, 127.1, 127.1, 125.4, 125.4, 120.1, 65.7, 59.5, 46.7, 29.2 (t, *J* = 20.4 Hz), 19.1, 18.0 ppm; IR (film): ṽ = 3315, 2925, 1692, 1528, 1451, 1423, 1322, 1284, 1249, 1092, 1042, 1002, 941, 780, 760, 740, 631 cm^{-1}; HRMS (ESI): m/z calcd for $C_{20}H_{21}{}^{15}NO_4Na$ [M+Na]+: 363.1426, found: 363.1437.

Labelled ACV (*S*)-2-amino-6-(((*R*)-1-(((*R*)-1-carboxy-2-methylpropyl-2-d)amino)-3-mercapto-1-oxopropan-2-yl)amino)-6-oxohexanoic acid

Valine-^{15}*N* (9)[7]

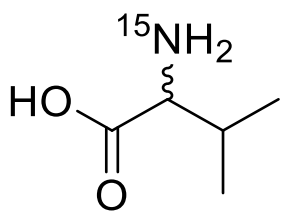

The General Method for reductive amination was followed using sodium 3-methyl-2-oxobutanoate (**6**, 1.86 g, 20.0 mmol) and $^{15}NH_4Cl$ (2.18 g, 40.0 mmol) to give **9** of 18% purity (determined by quantitative NMR using 3-(trimethylsilyl)-2,2,3,3-tetradeuteropropionic acid as internal standard) as a white solid (7.21 g, 8.17 mmol, 41%), which was directly used in the next step; ^{1}H NMR (400 MHz, 300 K, D_2O) δ = 4.04 (d, *J* = 4.4 Hz, 1H), 2.51 – 2.37 (m, 1H), 1.13 (dd, *J* = 9.8, 7.0 Hz, 6H) ppm; ^{13}C NMR (101 MHz, D_2O) δ = 171.9, 58.5 (d, *J* = 6.3 Hz), 29.1, 17.6, 17.0 ppm.

(((9*H*-Fluoren-9-yl)methoxy)carbonyl)valine-^{15}*N* (10)

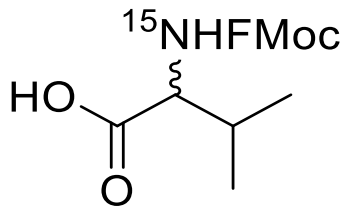

The General Method for Fmoc protection was followed using valine-^{15}*N* (**9**, 18wt%, 7.21 g, 8.17 mmol) following a flash column chromatography method (50 g Sfär Duo Silica; 80 mL/min using 100% (v/v) CH_2Cl_2 (+ 0.1% (v/v) formic acid) (2 CV), followed by a linear gradient (30 CV): 0%→100% (v/v) (20% (v/v) EtOAc in CH_2Cl_2 (+ 0.1% (v/v) formic acid)) in CH_2Cl_2 (+ 0.1% (v/v) formic acid); eluted at 40%) to obtain **10** as a white solid (343 mg, 1.01 mmol, 12%); mp 143 – 145 °C; ^{1}H NMR (700 MHz, 300 K, DMSO-*d*$_6$) δ = 12.57 (brs, 1H), 7.94 – 7.84 (m, 2H), 7.79 – 7.72 (m, 2H), 7.60 (dd, *J* = 92.8, 8.7 Hz, 1H), 7.48 – 7.37 (m, 2H), 7.33 (tdd, *J* = 7.4, 3.7, 1.1 Hz, 2H), 4.32 – 4.18 (m, 3H), 3.88 (ddd, *J* = 8.8, 6.1, 1.4 Hz, 1H), 2.07 (hd, *J* = 6.8, 2.9 Hz, 1H), 0.91 (d, *J* = 6.8 Hz, 6H) ppm; ^{13}C NMR (176 MHz, 300 K, DMSO-*d*$_6$) δ = 173.3, 156.4,156.3, 143.8, 143.8, 140.7, 140.7, 127.6, 127.6, 127.1, 127.0, 125.4, 125.4, 120.1, 120.1, 65.7, 59.6, 59.5, 46.6, 29.6, 19.2, 18.1 ppm; IR (film): ṽ = 3306, 2950, 1703, 1513, 1451, 1426, 1322, 1287, 1272, 1236, 1083, 1026, 996, 915, 760, 740, 713, 631 cm^{-1}; HRMS (ESI): m/z calcd for $C_{20}H_{21}{}^{15}NO_4Na$ [M+Na]+: 363.1333, found: 362.1360.

(*S*)-2-Amino-6-(((*R*)-1-(((*R*)-1-carboxy-2-methylpropyl-2-*d*)amino)-3-mercapto-1-oxopropan-2-yl)amino)-6-oxohexanoic acid (ACdV)

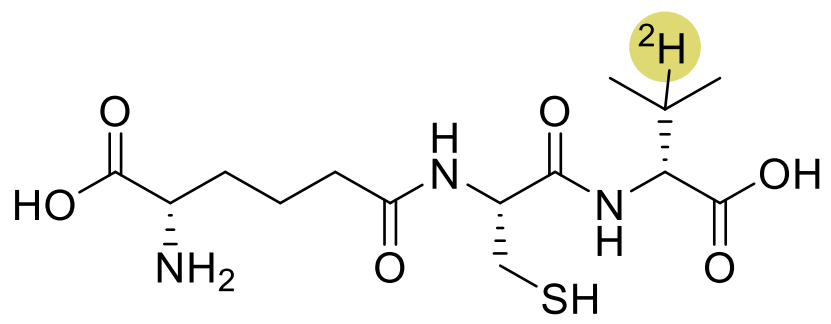

The General Method for solid phase peptide coupling was followed to obtain ACdV.

^{1}H NMR (700 MHz, 298 K, D_2O) δ = 4.59 (dd, *J* = 7.2, 5.5 Hz, 1H), 4.15 (s, 1H), 3.75 (dd, *J* = 6.8, 5.5 Hz, 1H), 2.98 (dd, *J* = 14.1, 5.5 Hz, 1H), 2.93 (dd, *J* = 14.1, 7.3 Hz, 1H), 2.47 – 2.42 (m, 2H), 1.96 – 1.86 (m, 2H), 1.81 – 1.67 (m, 2H), 0.95 (s, *3*H), 0.91 (s, *3*H) ppm;

(*S*)-2-Amino-6-(((*R*)-1-(((*R*)-1-carboxy-2-methylpropyl)amino-^{15}*N*)-3-mercapto-1-oxopropan-2-yl-1,2,3-^{13}*C*$_3$)amino-^{15}*N*)-6-oxohexanoic acid (($^{13}C_3$,$^{15}N_2$)ACV)

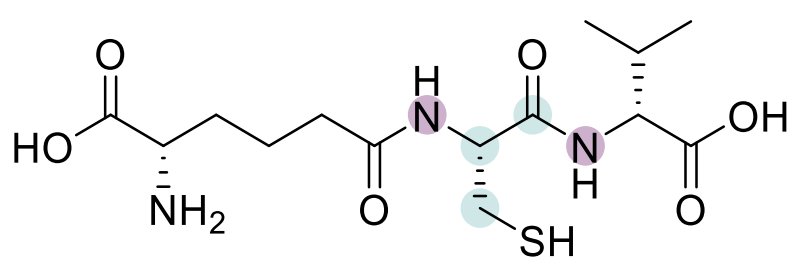

General Method for solid phase peptide coupling was followed to obtain the labelled **(**$^{13}C_3$,$^{15}N_2$)ACV .

^{1}H NMR (700 MHz, 300 K, D_2O) δ = 4.59 (dp, *J* = 144.0, 5.5 Hz, 1H), 4.25 (ddd, *J* = 6.0, 2.8, 1.3 Hz, 1H), 3.80 (dd, *J* = 6.8, 5.5 Hz, 1H), 3.14 – 2.76 (m, 2H), 2.49 – 2.38 (m, 2H), 2.20 (ddt, *J* = 13.5, 9.8, 6.7 Hz, 1H), 2.01 – 1.83 (m, 2H), 1.83 – 1.61 (m, 2H), 0.96 (dd, *J* = 19.5, 6.8 Hz, 6H) ppm; ^{13}C NMR (176 MHz, 300 K, DMSO-*d*$_6$) δ = 176.1, 175.9 (d, *J* = 15.0 Hz), 174.1, 171.9 (dd, *J* = 52.8, 16.2 Hz), 59.2 (d, *J* = 11.4 Hz), 55.7 (m), 54.2, 34.6 (d, *J* = 7.4 Hz), 30.1, 29.7, 25.4 (d, *J* = 36.3 Hz), 20.8, 18.6, 17.2 ppm.

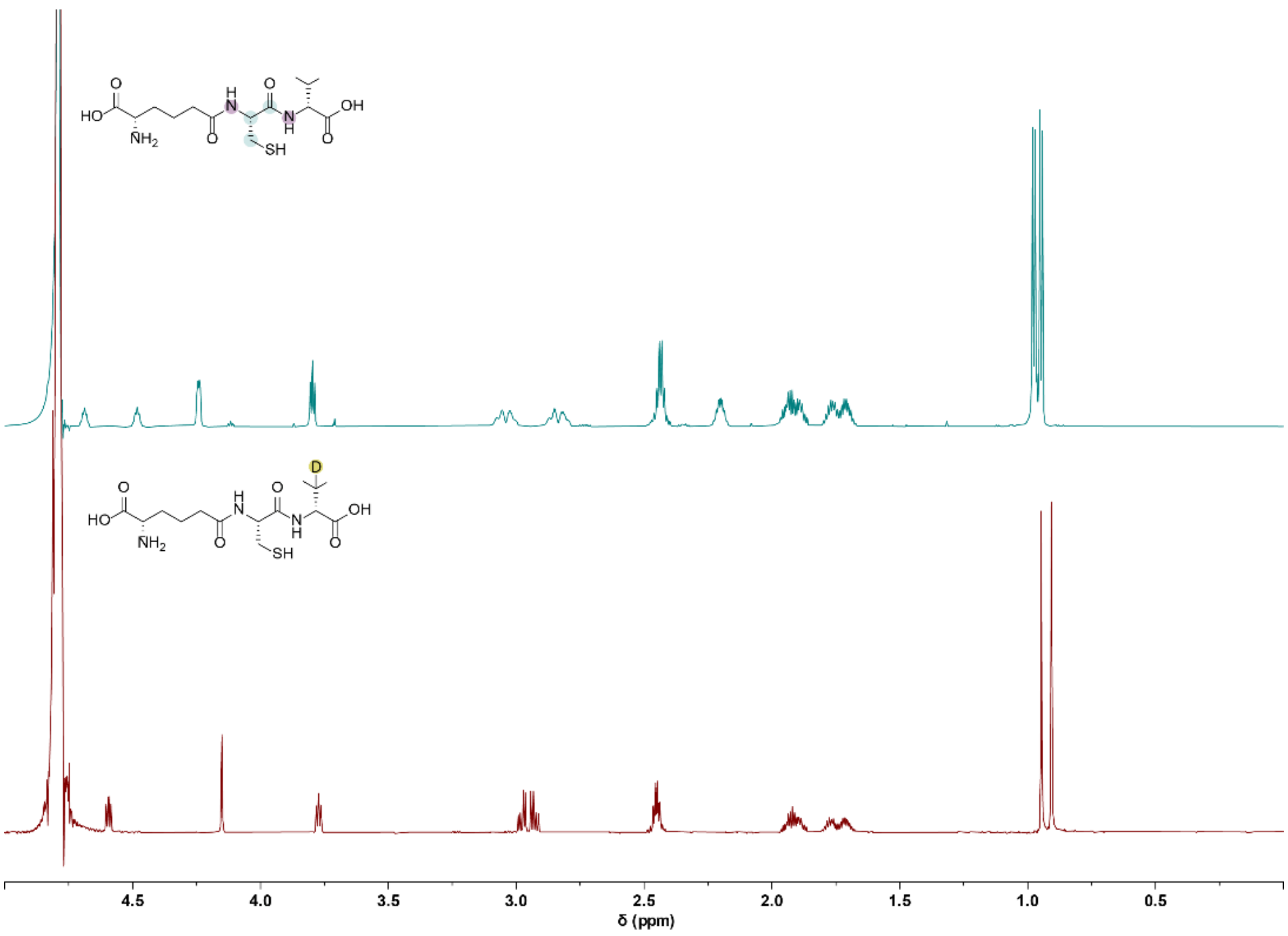

Supplementary Figure 3. ^{1}H NMR spectra of ACdV and ($^{13}C_3$,$^{15}N_2$)ACV.

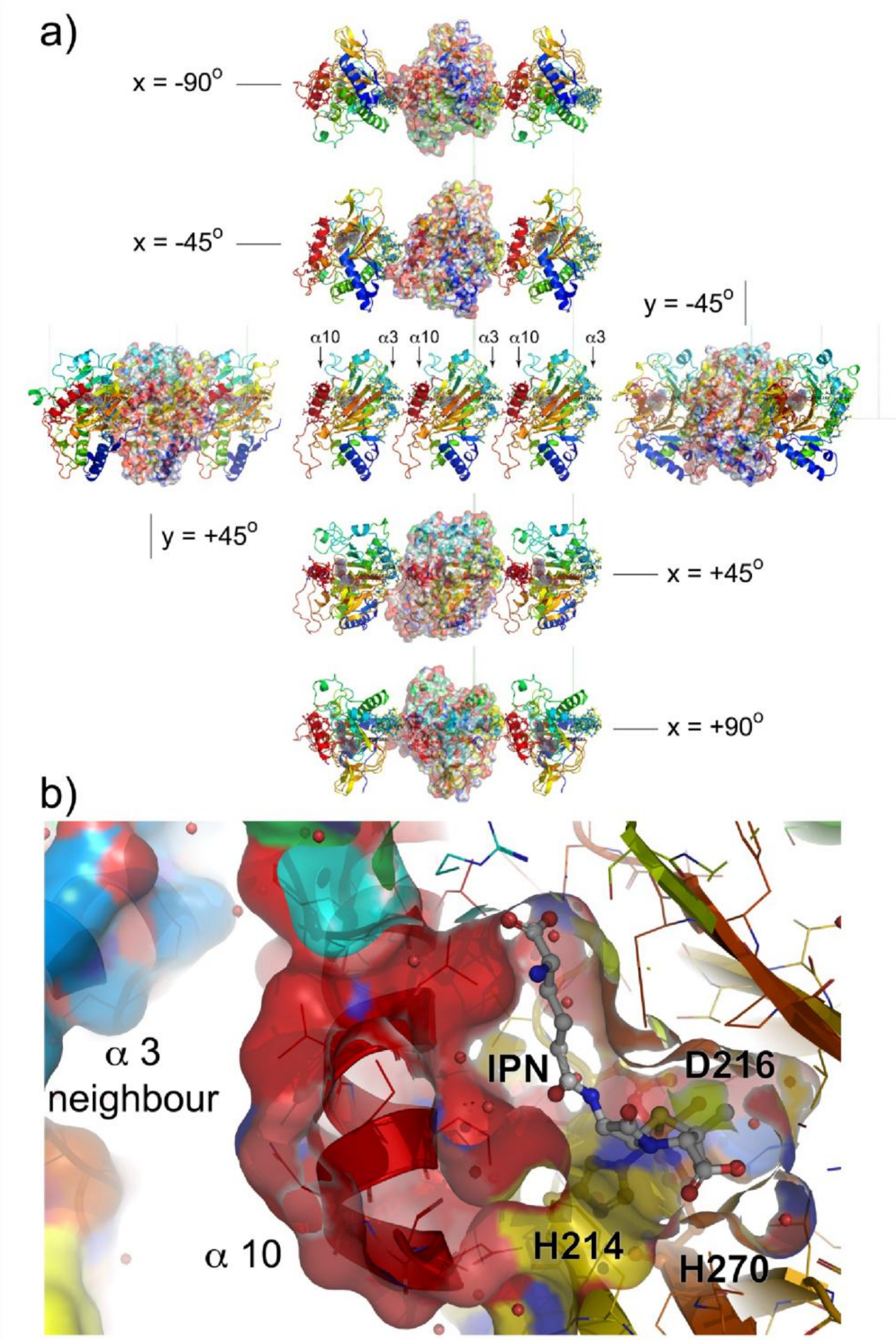

Supplementary Figure 4. Crystal packing in the orthorhombic crystal form of the IPNS:Fe:ACV complex. a) Three molecules of the IPNS:Fe:ACV anaerobic complex (PDB ID: 6ZAE) are shown to illustrate the lattice packing between helix α3 and α10 from neighbouring enzyme molecules. b) A close-up view of the IPNS:Fe:IPN product complex (PDB ID: 8P46) showing how α10 prevents product dissociation because it packs against α3 of a neighbouring enzyme. Each of the three protein chains are coloured in rainbow style from the N-terminus (blue) to the C-terminus (red).

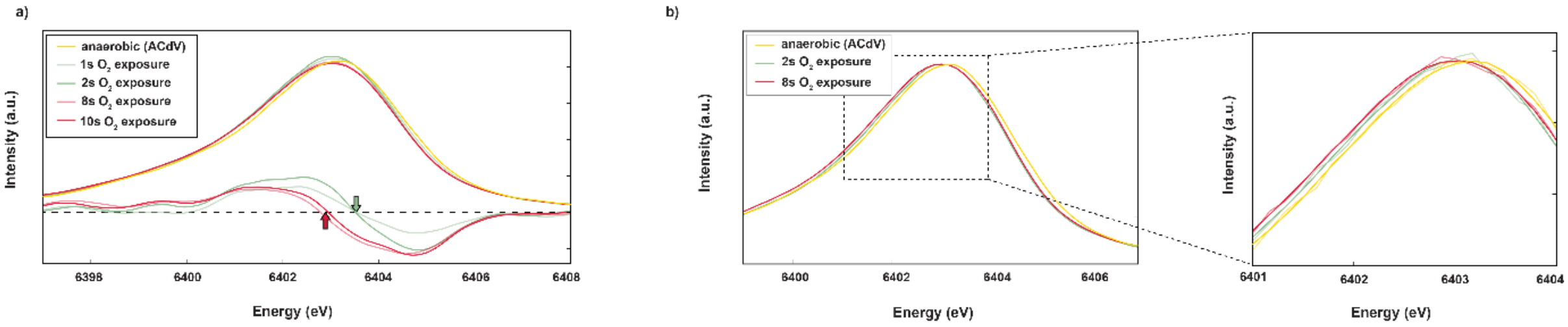

Supplementary Figure 5: Detailed analysis of the Fe Kα_1 emission profile during IPNS catalysis revealing global shift of the emission envelope. (a) Area-normalized Fe Kα_1 emission region highlighting spectral changes upon O_2 exposure with overlaid spectra for anaerobic IPNS:Fe:ACdV and selected time points (1 s, 2 s, 8 s, 10 s) revealing progressive redistribution of intensity across the Kα_1 band. Difference spectra (relative to the anaerobic dataset) emphasise non uniform shifts, with early time points (1–2 s) showing localized changes, whereas later time points (8–10 s) exhibit a more global shift of the emission envelope towards lower energy. These changes are consistent with evolution of the Fe electronic structure along the reaction coordinate rather than simple peak broadening. Green and red arrows indicate the shift in the cross-over points on the difference spectra between early (1–2 s) and later (8–10 s) time points, respectively. **(b)** Expanded view of the Kα_1 maximum highlighting energy shifts of the emission peak. Here the spectra are normalized by the Kα_1 peak intensity. Later time points (8 s, 10 s) show a more pronounced displacement of the peak maximum compared to early intermediates, consistent with a progressive shift of the emission band rather than changes in peak width.

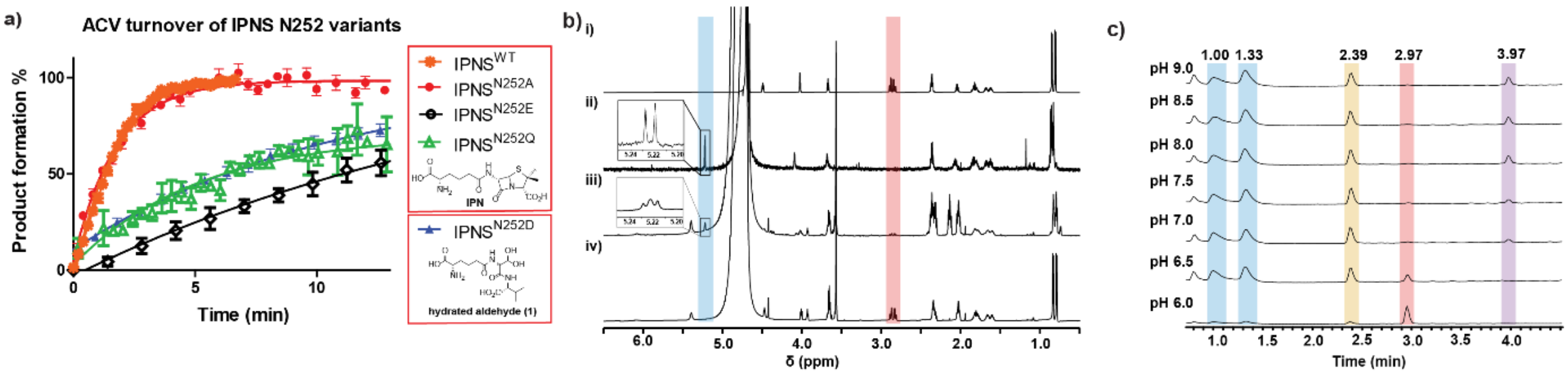

Supplementary Fig. 6. Additional biochemical characterisation of the IPNS^{N252D} variant using SPE-MS, LC-NS and NMR spectroscopy. (a) ACV turnover of IPNS^{N252X} variants (2 μM), ACV (400 μM), $(NH_4)_2Fe(SO_4)_2$ (100 μM), TCEP (800 μM) and L-ascorbate (800 μM) in 25 mM Tris, 100 mM NaCl pH 8.0 under ambient O_2 levels using solid-phase extraction MS with standard error of the mean, n = 3 independent replicates.[3] Note for IPNS^{WT}, IPNS^{N252A}, IPNS^{N252Q} and IPNS^{N252E} IPN formation was observed in solution. By contrast, with IPNS^{N252D} no IPN formation was observed with the hydrated aldehyde (**1**) formation instead. **(b)** ^{1}H-NMR analysis of: (i) ACV (4 mM in D_2O), ii) isolated hydrated aldehyde **1** (~0.1 mM in D_2O), iii) and iv) an assay mixture containing IPNS^{N252D} (10 μM), ACV (250 μM), $(NH_4)_2Fe(SO_4)_2$ (50 μM), TCEP (250 μM) and L-ascorbate (250 μM) in Tris-d_{11} 90% (v/v) H_2O and 10% (v/v) D_2O under: (iii) anaerobic and (iv) O_2 exposed conditions (2.5 h O_2 exposure with ambient O_2). Note that IPNS^{N252D} produces both diastereoisomers of hydrated aldehyde **1**, rather than IPN, as supported by the ^{1}H NMR reference spectrum for **1** (ii). The red shading highlights the Cβ protons of the ACV-derived cysteinyl CH_2, while the blue shading marks the Cβ doublets appearing as a triplet from both diastereoisomers of **1** (iii). In D_2O, the Cα position is deuterated, so the signals appear as two singlets (ii). **(c)** LC-MS analysis on the pH dependency of IPNS^{N252D} (2 μM), ACV (200 μM), $(NH_4)_2Fe(SO_4)_2$ (500 μM), TCEP (400 μM) and L-ascorbate (400 μM) in 25 mM Tris, 100 mM NaCl at varying pH 6.0 - 9.0 under ambient O_2 levels. Both C-a diastereoisomers of hydrated aldehyde **1** (blue boxes) are formed at varying pH values; catalytic efficiency is decreased at pH 6 and 6.5 with ACV (red shading). At higher pH more ACV dimer is formed (purple shading).

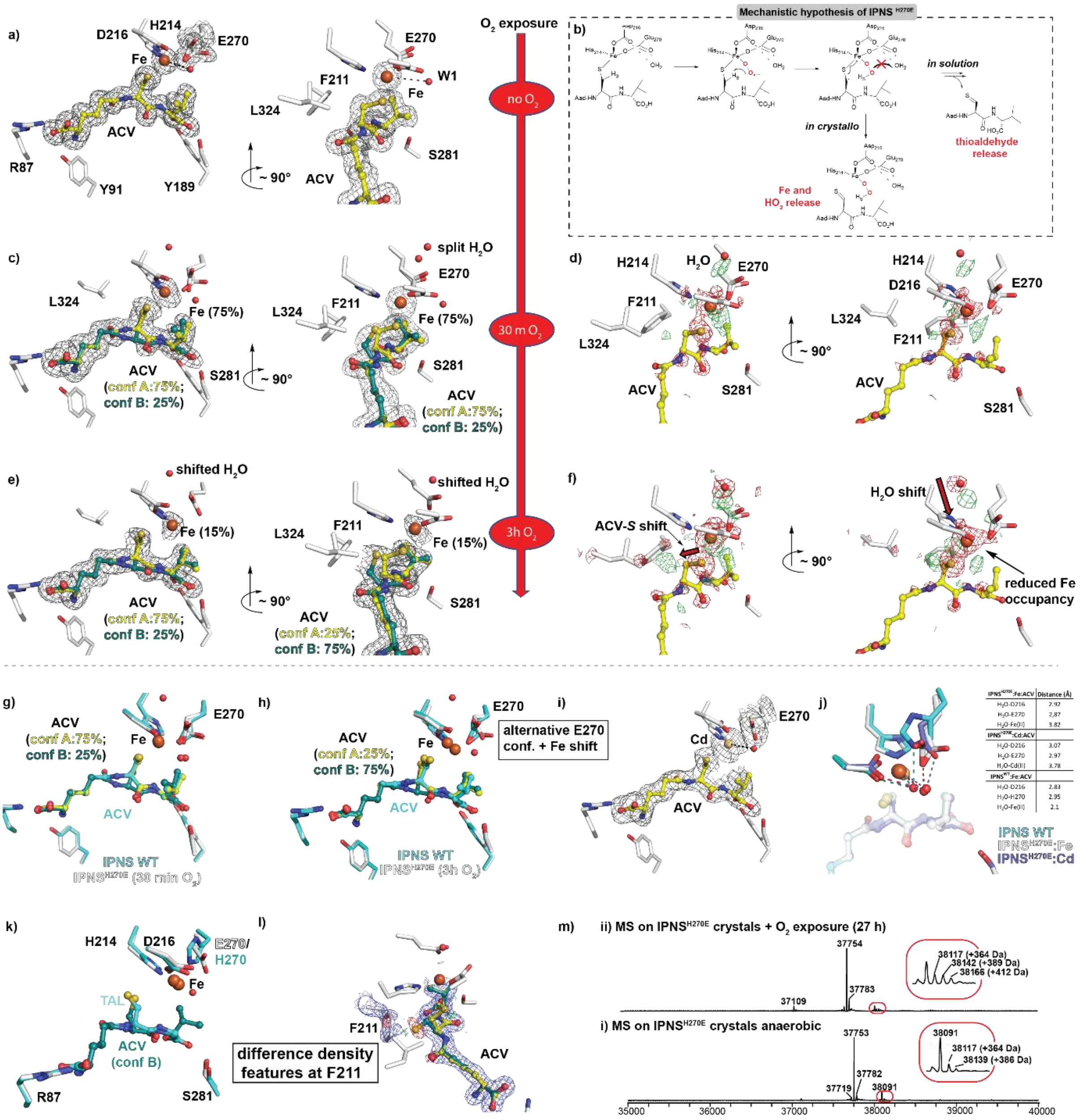

Supplementary Fig. 7. Crystallographic studies on IPNS^{H270E} with Fe(II) or Cd(II) and ACV exposed to O_2. Polder omit electron density maps (3.0 σ contour level, carved around E270, Fe and ACV) for **(a)** Anaerobic IPNS^{H270E}:Fe:ACV (PDB: 7ZOE, 1.50 Å resolution), **(c)** IPNS^{H270E}:Fe:ACV structure (exposed to O_2 for 30 min, PDB: 8ALI, 1.60 Å resolution) and **(e)** IPNS^{H270E}:Fe:ACV structure (exposed to O_2 for 3 hours, PDB: 8ALJ, 1.68 Å resolution), showing key ACV (R87, Y91, Y189 and S281) and metal interactions (H214, D216 and E270). After 30 min O_2 exposure, the Fe occupancy reduces to 75% and ACV adopts two conformations: A (yellow, 75%), as in the original orientation and B (teal, 25%) where the ACV-S is positioned away from Fe (near to Phe211). After 3 h, the Fe occupancy drops further (15%) with refined conformations A (25%) and B (75%) and positional shifts in Fe and E270, respectively. Note, (partial) ACV oxidation to a TAL is possible (Supplementary Fig. 8). These structures were determined by cryogenic synchrotron crystallography (MX) at DLS. **(b)** Schematic for reaction of IPNS^{H270E}:Fe:ACV with O_2. E270 (and D216) form a hydrogen bond with W1, preventing direct Fe coordination. The results imply MCI formation proceeds via Fe-coordinated W1 (path b), rather than direct abstraction of ACV-NH by the peroxide intermediate (path a) (Fig. 1). With IPNS^{H270E}, the inability to homolytically cleave the peroxide due to the absence of W1 stalls reaction at the peroxide/TAL stage, resulting in TAL release into solution. **(d)** $F_{obs} - F_{obs}$ isomorphous

difference map of the active site displayed at +3.0 σ (green) and −3.0 σ (red) contour levels for the IPNS^{H270E}:Fe:ACV structure (anaerobic, PDB: 7ZOE) relative to the IPNS^{H270E}:Fe:ACV structure (exposed to O_2 for 30 min, PDB: 8ALI) revealing reduced Fe occupancy, a partial shift of the ACV-S and a partial movement of H_2O (split H_2O). **(f)**) $F_{obs} - F_{obs}$ isomorphous difference map displayed at +3.0 σ (green) and −3.0 σ (red) contour levels for the IPNS^{H270E}:Fe:ACV structure (anaerobic, PDB: 7ZOE) relative to the IPNS^{H270E}:Fe:ACV structure (exposed to O_2 for 3 hours, PDB: 8ALJ) revealing further reduced Fe occupancy, a shift of the ACV-*S* and a full movement of H_2O. Red arrows indicate movement of the ACV-*S* and H_2O**.** **(g)** Superimposition of IPNS^{H270E}:Fe:ACV structure (anaerobic, cyan) relative to the IPNS^{H270E}:Fe:ACV structure (30 min O_2 exposure) revealing slight differences in ACV orientation upon O_2 exposure. **(h)** Superimposition of IPNS^{H270E}:Fe:ACV structure (anaerobic, cyan) relative to the IPNS^{H270E}:Fe:ACV (3h O_2 exposure) revealing differences in ACV orientation, the re-orientation of the E270 sidechain and of the Fe on extended O_2 exposure. **(i)** Polder omit electron density map (3.0 σ contour level, carved around E270, Fe and ACV) for IPNS^{H270E}:Cd:ACV (PDB: 8A4G, 1.97 Å resolution), which was also obtained in our analyses. **(j)** Superimposition of anaerobic IPNS^{H270E}:Fe:ACV (PDB: 7ZOE), anaerobic IPNS^{H270E}:Cd:ACV (PDB: 8A4G) and anaerobic IPNS^{WT}:Fe:ACV (PDB: 6ZAE)[1] highlighting the different coordination modes of the X270 ligand with Fe and the location of the closest metal(-coordinated) water W1. The table highlights the distance of the water with the closest residues/Fe in Å. **(k)** Superimposition of IPNS^{H270E}:Fe:ACV (3h O_2 exposure, PDB: 8ALJ, ACV conformation B) with IPNS^{WT}:Fe:ACV (4s O_2 exposure using the tape drive, PDB: 8PYA) highlighting the different conformations of the ACV/TAL-S during release from the Fe centre. **(l)** $2mF_o$-dF_c (blue: 1.0 σ contour level) and mF_o-dF_c (green: +3.0 σ and red: -3.0 σ) density maps of an alternative model of the IPNS^{H270E}:Fe:ACV structure exposed to O_2 for 3 hours with the ACV-*S* positioned as observed in tr-SFX analyses. Although we cannot exclude the possibility of the model containing the ACV-S is flipped by 180° as in our time-resolved SFX structures after 3s and 4s O_2, as implied by the mF_o-DF_c density features the difference features suggest that the ACV-S has moved away from the Fe without rotation. **(m)** MS of IPNS^{H270E}:Fe:ACV crystals exposed to O_2. Crystals were exposed to an O_2 saturated crystallisation solution for a defined time, followed by harvesting of multiple crystals (>9) and dissolving in H_2O and analysis. Deconvoluted mass spectrum of IPNS^{H270E}:Fe:ACV crystals dissolved in H_2O: (i) under anaerobic conditions revealing the mass of IPNS^{H270E} with 37753 Da with an unknown low-level modification of +339 Da (38091 Da) and(ii) after 27 h O_2 exposure revealing low level degradationof the IPNS^{H270E} (37109 Da). These experiments were performed in the same way as those involving crystallographic data collection and besides the low levels of degradation, they imply a lack of a (sufficiently stable) covalent modification of the IPNS^{H270} crystals.

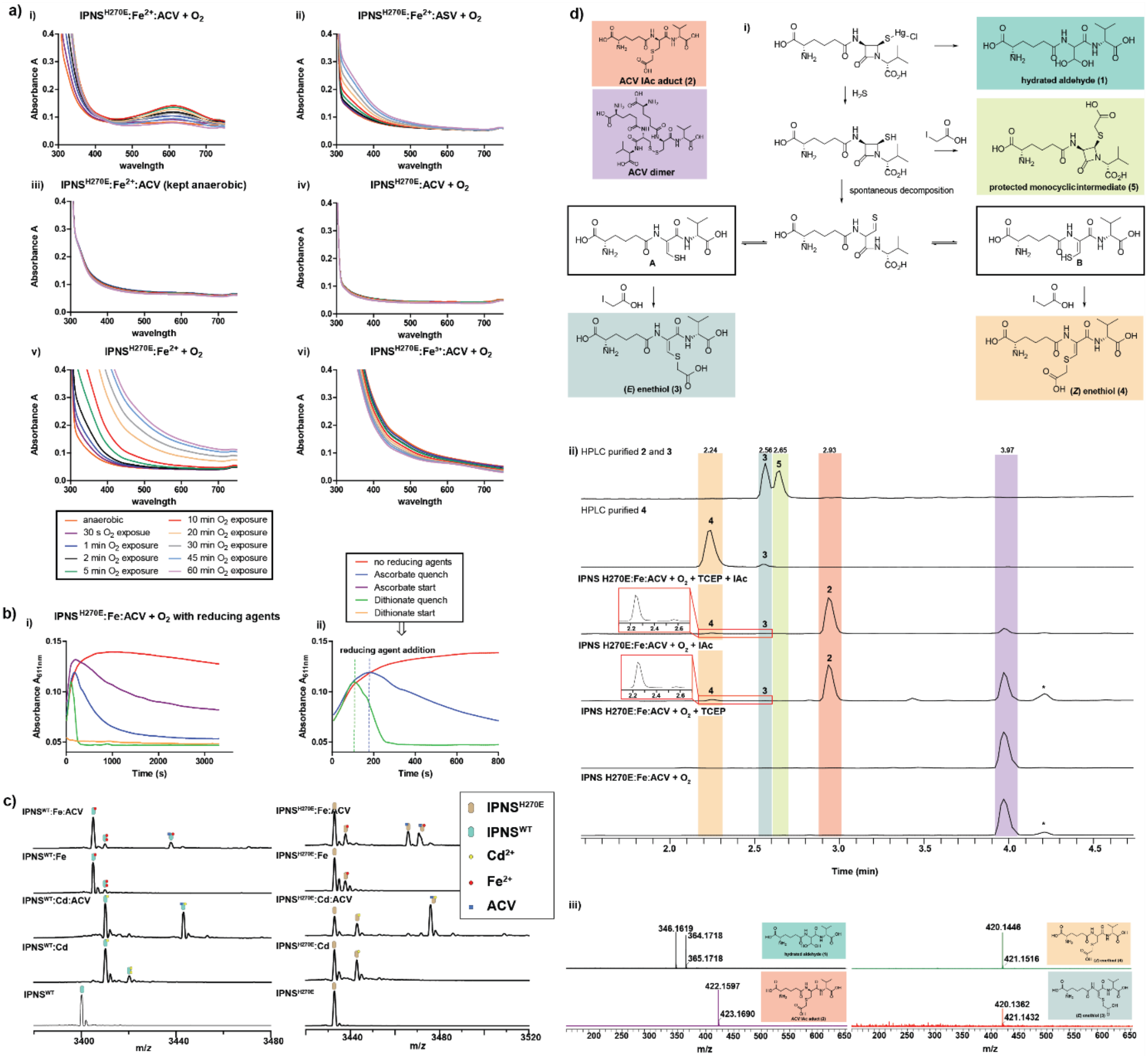

Supplementary Fig. 8. Characterisation of the IPNS^{H270E} Variant and Identification of Products. (a) UV/Vis (300 -750 nm) analysis of IPNS^{H270E} (300 µM, 75 µL) ± Fe^{2+}/ Fe^{3+} (1 eq., 300 µM) ± ACV/ASV (5 eq., 1.50 mM) in MOPS buffer (100 mM in H_2O, pH 7.2) under anaerobic conditions in a quartz cuvette sealed with a rubber septum. Reaction was initiated by mixing with O_2 saturated buffer (100 mM MOPS in H_2O, pH 7.2, 75 µL with ~1.0 mM O_2, determined with an Oxygraph machine) demonstrating the formation of a species with an absorption peak maximum at 611 nm: (i) IPNS^{H270E} + $FeSO_4$ + ACV + O_2, (ii) IPNS^{H270E} + $FeSO_4$ + ASV + O_2, (iii) IPNS^{H270E} + $FeSO_4$ + ACV no O_2, (iv) IPNS^{H270E} + ACV + O_2, (v) IPNS^{H270E} + $FeSO_4$ + O_2 and (vi) IPNS^{H270E} + Fe(III), ACV + O_2. Absorption spectra were recorded under anaerobic conditions and after 30 s, 1 min, 2 min, 5 min, 10 min, 15 min, 20 min, 30 min, 45 min and 60 min O_2 exposure. Note: The shift of the baseline in panel v) is likely caused by precipitation of the oxidized IPNS^{H270E}complex. **(b)** UV/Vis spectra of IPNS^{H270E}:Fe(II):ACV + O_2 treated with different reducing agents. i) Time-course (at 611 nm) of IPNS^{H270E} (150 µM), Fe^{2+} (1 eq., 150 µM), ACV (5 eq., 750 µM) in MOPS buffer (100 µL, 100 mM in H_2O, pH 7.2) prepared under anaerobic conditions, followed by addition of O_2 saturated buffer (100 mM MOPS in H_2O, pH 7.2) as above. The reducing agent (either sodium dithionate or sodium ascorbate, 50 eq., final concentration 7.5 mM, 1 µL) was either added prior to O_2 exposure ("start") or after formation of the semi-stable blue-coloured intermediate by mixing. ii) Zoom on the UV/Vis spectrum in (i) revealing the speed of the quench reaction of the absorption peak at 611 nm by sodium dithionate (addition time 2.50 min, green dashed line) and sodium ascorbate (addition time 3 min, blue dashed line). **(c)** Non-denaturing mass spectra of IPNS^{WT} and IPNS ^{H270E} ± Fe(II) ± Cd(II) ± ACV under ambient O_2 levels.

The +11 charge state is shown. Note: IPNS^{WT} (mass: 37522) was produced with a different construct, as reported[1], compared to IPNS^{H270E} (mass: 37752 Da), Left: Mass spectra of i) IPNS^{WT}, ii) IPNS^{WT} with Cd^{2+} (25 eq., 62.5 μM), iii) IPNS^{WT}:Cd with ACV (125 eq., ~300 μM), iv) IPNS^{WT} with Fe^{2+} (25 eq., 62.5 μM) and v) IPNS^{WT}:Fe with ACV (125 eq., ~300 μM). Right: Mass spectrum of i) IPNS^{H270E}, ii) IPNS^{H270E} with Cd^{2+} (25 eq., 62.5 μM), iii) IPNS^{H270E}:Cd with ACV (125 eq., ~300 μM), iv) IPNS^{H270E} with Fe^{2+} (25 eq., 62.5 μM) and v) IPNS^{H270E}:Fe with ACV (125 eq., ~300 μM). Note more of the IPNS^{H270E}:Fe:ACVspecies was observed compared to IPNS^{WT} suggesting less efficient H270E turnover.**(d**) i) The monocyclic mercury salt was treated with H_2S (see Supplementary Information); residual H_2S was removed and the mixture treated with iodoacetic acid. After treatment with H_2S the monocyclic thiol is formed, which decomposes to the thioaldehyde and tautomerises to the enethiols, which react with iodoacetic acid. HPLC purification yielded both diastereoisomers of the protected enethiol (**3** (*E*) green and **4** (Z) orange), the hydrated aldehyde (**1**, light blue) and the monocyclic derivative (**5**, light green, for full NMR characterisation see Supplementary Information).Note: enethiol **3** was not separated from the monocyclic derivatve , but both were characterised using 1D and 2D NMR and MS. Note that without iodoacetic acid the underivatised enethiols were observed in crude NMR mixtures, but we were unable to isolate them. (ii) LC-MS spectra of isolated reference compounds compared to IPNS^{H270E} products. Spectra (shown bottom to top) were recorded in tris buffer (25 mM in H_2O, pH 8.0) under the following conditions: IPNS^{H270E} (5 μM) + $FeSO_4$ (20 μM in H_2O) + ACV (100 μM) + sodium ascorbate (100 μM); as above + TCEP (100 μM)); as above + iodoacetic acid (250 μM); as above + TCEP (100 μM) + Iodoacetic acid (250 μM); isolated (*Z*)-enethiol (**4**) with minor impurity of (*E*)-enethiol (**3**) and (*E*)-enethiol (**3**) with inseparable impurity of the protected monocyclic intermediate (**5**). A minor peak at ~4.3 min (marked *) corresponds to m/z 543.7171 (2+ charge state, positive mode), consistent with formation of a higher-mass oligomeric species under oxidative conditions, likely arising from partial coupling of three molecules of ACV and/or the corresponding thioaldehyde. Its precise structure could not be unambiguously assigned. An additional peak at ~3.4 min (marked as #, m/z 454.1316, positive mode) is approximately 32 Da higher than iodoacetamide-protected ACV (422.1596 Da), consistent with further oxidative modification; however, the structure of this species was not assigned. iii) HR-MS spectra of (*E*)-enethiol (**3**), (*Z*)-enethiol (**4**), hydrated aldehyde (**1**) and IAc- protected ACV (**2**) formed by IPNS^{H270E}.

Synthesis of monocyclic mercury salt:[10,11]

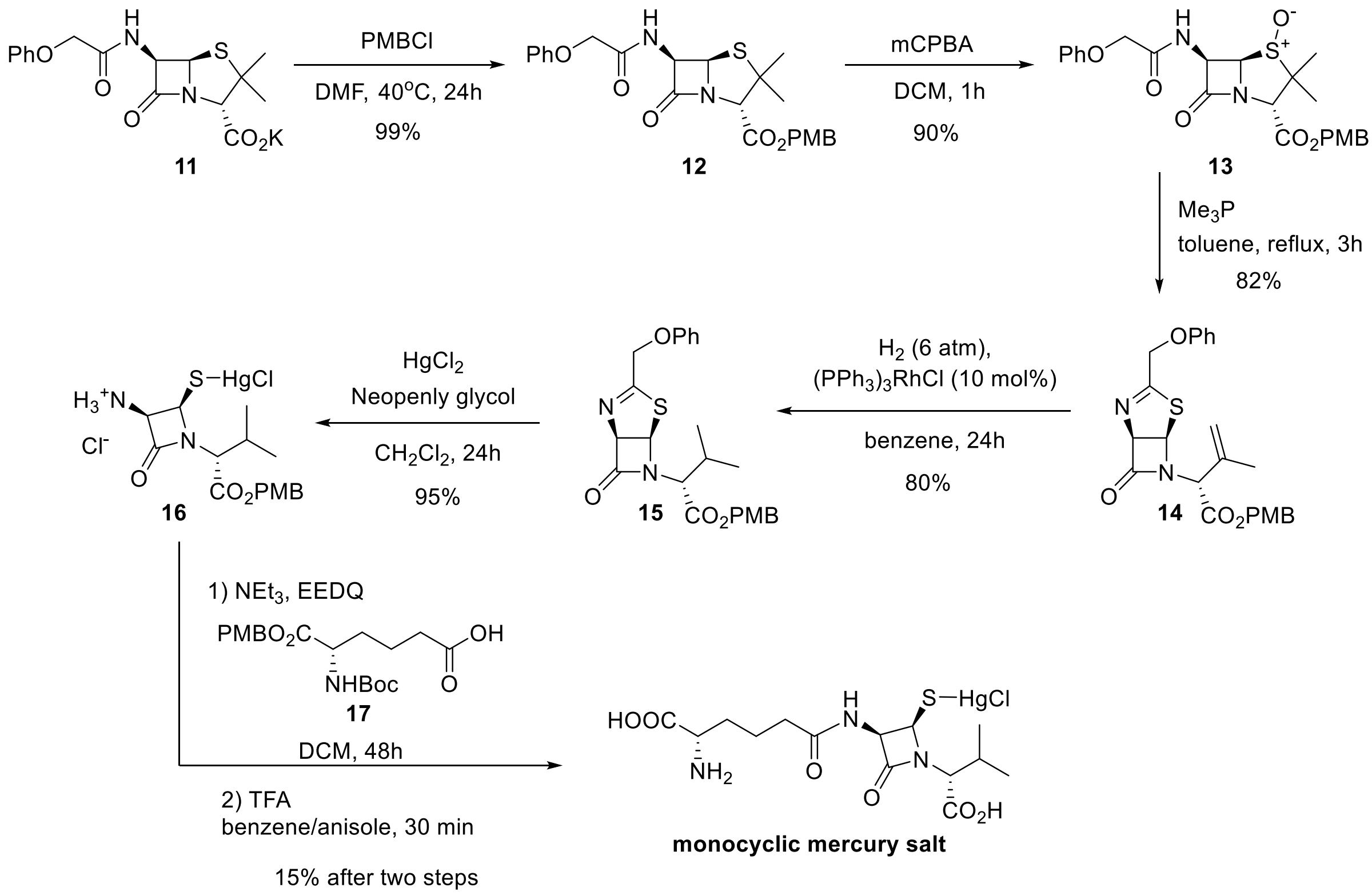

Supplementary Figure 9. The synthesis of the monocyclic mercury salt followed reported procedures.[10,11]

4-Methoxybenzyl (2*S*,5*R*,6*R*)-3,3-dimethyl-7-oxo-6-(2-phenoxyacetamido)-4-thia-1-azabicyclo [3.2.0]heptane-2-carboxylate (12)

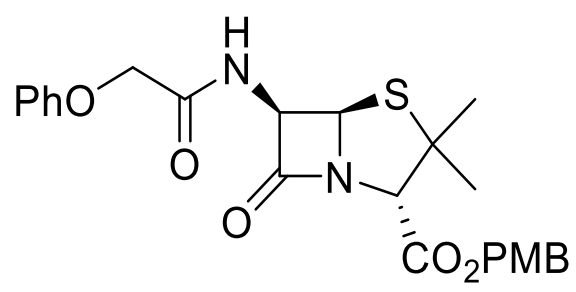

The potassium salt of penicillin V (**11**, 10.1 g, 26.0 mmol) and PNBCl (4.27 g, 28.3 mmol, 1.05 equiv.) were dissolved in DMF (60 mL); the resulting mixture was stirred at 40°C for 24h. Reaction mixture was concentrated under reduced pressure and the residue was poured into water (100 mL). The solution was extracted with EtOAc (60 mL x3). The combined organic layers were washed with water (100 mL), brine (100 mL), dried over anhydrous Na_2SO_4 and concentrated under reduced pressure. Product **12** (12.1 g, 99% yield) was obtained as yellowish oil and was used in the next step without further purification.

^{1}H NMR (300 MHz, $CDCl_3$) δ 7.42 – 7.27 (m, 5H), 7.12 – 6.99 (m, 1H), 6.95 – 6.86 (m, 4H), 5.73 (dd, *J* = 9.2, 4.3 Hz, 1H), 5.57 (d, J = 4.3 Hz, 1H), 5.24 – 5.07 (m, 2H), 4.60 – 4.49 (m, 2H), 4.46 (s, 1H), 3.82 (s, 2H), 1.55 (s, 3H), 1.39 (s, 3H). Spectroscopic data were in accord with those reported.[11]

4-Methoxybenzyl (2*S*,4*R*,5*R*,6*R*)-3,3-dimethyl-7-oxo-6-(2-phenoxyacetamido)-4-thia-1-azabicyclo [3.2.0]heptane-2-carboxylate 4-oxide (13)

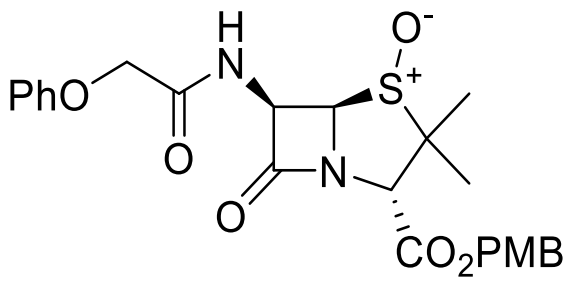

Ester **12** (5.40 g, 11.5 mmol) was dissolved in CH_2Cl_2 (50mL) and the resulting solution was cooled to 0ºC. mCPBA (2.77 g, 75%, 12.05 mmol, 1.05 equiv.) was added portionwise. The reaction mixture was warmed to room temperature and stirred for 1h. The mixture was poured into saturated $NaHCO_3$ solution (50 mL), layers were separated and the aqueous layer was extracted with CH_2Cl_2 (30 mL x2). Combined organic layers were washed with brine (50 mL), dried over anhydrous Na_2SO_4 and concentrated under reduced pressure. Purification by flash column chromatography on silica (30% to 60% EtOAc in hexanes) provided the desired sulfoxide **13** (4.89 g, 90% yield) as a white amorphous solid.

^{1}H NMR (400 MHz, $CDCl_3$) δ 8.25 (d, J = 10.5 Hz, 1H), 7.35 – 7.27 (m, 4H), 7.04 – 6.97 (m, 1H), 6.96 – 6.87 (m, 4H), 6.09 (dd, J = 10.5, 4.6 Hz, 1H), 5.26 (d, J = 11.7 Hz, 1H), 5.10 (d, J = 11.7 Hz, 1H), 5.01 (d, J = 4.6 Hz, 1H), 4.67 (s, 1H), 4.53 (s, 2H), 3.82 (s, 3H), 1.66 (s, 3H), 1.05 (s, 3H). Spectroscopic data were in accord with those reported.[11]

4-Methoxybenzyl (2*R*)-3-methyl-2-(7-oxo-3-(phenoxymethyl)-4-thia-2,6-diazabicyclo[3.2.0]hept-2-en-6-yl)but-3-enoate (14)

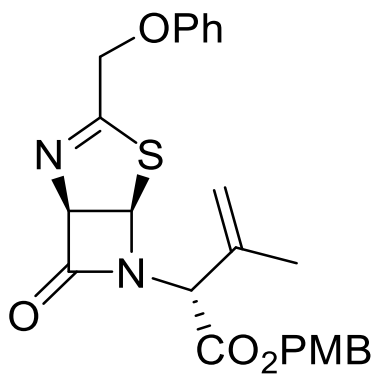

The sulfoxide **13** (4.00 g, 8.22 mmol) and trimethyl phosphite (1.07 mL, 9.05 mmol, 1.1 equiv.) were dissolved in dry toluene (50 mL) and the resulting solution was refluxed using Dean and Stark apparatus for 3h. Reaction mixture was cooled and then concentrated under reduced pressure. Residue was purified by flash column chromatography on silica (20% to 50% EtOAc in hexanes). Additional purification by recrystallization from hexane/CH_2Cl_2 provided **14** (3.05 g, 82% yield) as colorless prisms.

^{1}H NMR (300 MHz, $CDCl_3$) δ 7.35 – 7.23 (m, 4H), 7.03 – 6.96 (m, 1H), 6.94 – 6.84 (m, 4H), 6.00 (dt, J = 4.1, 1.3 Hz, 1H), 5.91 (d, J = 4.1 Hz, 1H), 5.17 – 5.06 (m, 2H), 5.06 – 5.02 (m, 1H), 5.00 – 4.86 (m, 2H), 4.86 – 4.80 (m, 2H), 3.81 (s, 3H), 1.72 (s, 3H). Spectroscopic data were in accord with those reported.[11]

4-Methoxybenzyl (R)-3-methyl-2-((1R,5R)-7-oxo-3-(phenoxymethyl)-4-thia-2,6-diazabicyclo [3.2.0]hept-2-en-6-yl)butanoate (15)

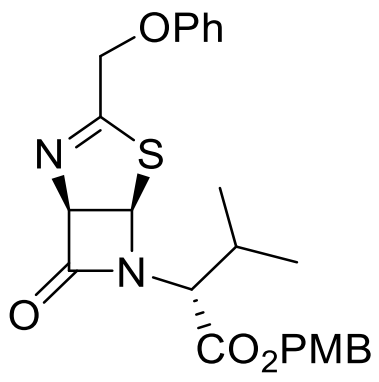

Alkene **14** (2.50 g, 5.52 mmol) and Wilkinson's catalyst (0.51 g, 0.52 mmol, 0.1 equiv.) were dissolved in dry, degassed benzene. Resulting solution was stirred under 6 atm of hydrogen for 24h. Reaction mixture was concentrated under reduced pressure and the residue was purified by flash column chromatography on silica (20% to 40% EtOAc in hexanes). Additional purification by recrystallization from hexane/CH_2Cl_2 provided **15** (2.00 g, 80% yield) as white needles.

^{1}H NMR (300 MHz, $CDCl_3$) δ 7.34 – 7.26 (m, 4H), 7.05 – 6.82 (m, 5H), 5.96 (dt, *J* = 4.1, 1.3 Hz, 1H), 5.82 (d, *J* = 4.1 Hz, 1H), 5.19 – 5.03 (m, 2H), 5.01 – 4.81 (m, 2H), 4.15 (d, *J* = 8.6 Hz, 1H), 3.82 (s, 3H), 2.28 (dsept, *J* = 8.6, 6.7 Hz, 1H), 0.92 (d, *J* = 6.7 Hz, 3H), 0.88 (d, *J* = 6.7 Hz, 3H). Spectroscopic data were in accord with those reported.[11]

((((2*R*,3*R*)-3-Ammonio-1-((*R*)-1-((4-methoxybenzyl)oxy)-3-methyl-1-oxobutan-2-yl)-4-oxoazetidin-2-yl)thio)mercury(II)) monochloride (16)

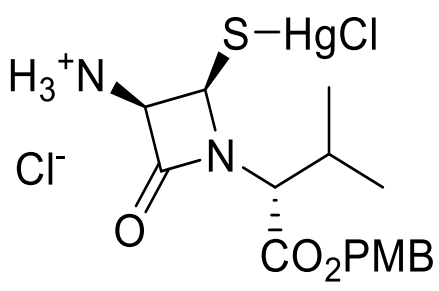

Thiazoline **15** (2.20 g, 4.84 mmol), neopentyl glycol (2.52 g, 24.2 mmol, 5 equiv.) and $HgCl_2$ (1.33 g, 4.88 mmol, 1.01 equiv.) were dissolved in dry CH_2Cl_2 and the resulting solution was stirred at room temperature for 72 h. White precipitate was filtered and washed with cold CH_2Cl_2 to provide mercury salt **16** (2.79 g, 95% yield) as colourless needles.

^{1}H NMR (300 MHz, DMSO-d_6) δ 8.64 (s, 2H), 7.44 – 7.27 (m, 2H), 6.98 – 6.86 (m, 2H), 5.68 (d, *J* = 4.7 Hz, 1H), 5.21 – 5.01 (m, 2H), 4.63 (d, *J* = 4.8 Hz, 1H), 3.94 (d, *J* = 8.9 Hz, 1H), 3.75 (s, 3H), 2.48 – 2.40 (m, 1H), 1.00 (d, *J* = 6.7 Hz, 3H), 0.84 (d, *J* = 6.7 Hz, 3H). Spectroscopic data were in accord with those reported.[11]

(*S*)-5-((tert-butoxycarbonyl)amino)-6-((4-methoxybenzyl)oxy)-6-oxohexanoic acid (17)

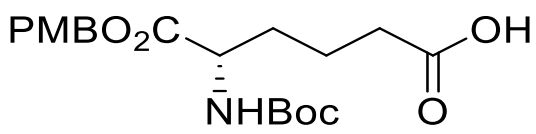

Boc-*L*-aminoadipic acid (1.00 g, 3.83 mmol) was dissolved in DMF (10 mL), PMBCl (0.57 mL, 4.21 mmol, 1.1 equiv) and TEA (0.64 mL, 4.59 mmol, 1.2 equiv) were added. The resulting solution was stirred at 40ºC for 24h. Reaction mixture was concentrated under reduced pressure and the residue was poured into water (30 mL). The solution was extracted with EtOAc (20 mL x3), combined organic layers were washed with water (30 mL), brine (20 mL), dried over anhydrous Na_2SO_4 and concentrated under reduced pressure. Purification by reverse phase flash column chromatography (30% to 90% MeCN in 0.1% aq. TFA) provided the desired product **17** (750 g, 51% yield) as a yellowish oil.

^{1}H NMR (300 MHz, $CDCl_3$) δ 7.33 – 7.26 (m, 2H), 6.91 – 6.84 (m, 2H), 5.17 – 5.01 (m, 3H), 4.37 – 4.27(m, 1H), 3.80 (s, 3H), 2.40 – 2.30 (m, 2H), 1.91 – 1.76 (m, 1H), 1.74 – 1.56 (m, 3H), 1.42 (s, 9H). Spectroscopic data were in accord with those reported.[11]

(((2*R*,3*R*)-3-((*S*)-5-Amino-5-carboxypentanamido)-1-((*R*)-1-carboxy-2-methylpropyl)-4-oxoazetidin-2-yl)thio)mercury(II) chloride (monocyclic mercury salt)

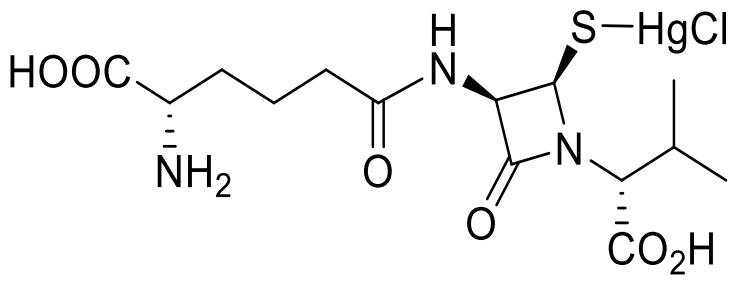

Mercury salt **16** (200 mg, 0.33 mmol), the protected aminoadipic acid (125 mg, 0.33 mmol, 1 equiv.) and EDDQ (81 mg, 0.33 mmol, 1 equiv) were dissolved in CH_2Cl_2 (5 mL), then TEA (0.048 mL, 0.34 mmol, 1.05 equiv) was added. The resulting solution was stirred at room temperature for 48 h, then concentrated under reduced pressure. The residue was dissolved in benzene:anisole:TFA (20:1:4) mixture and the resulting mixture stirred for 30 min. The mixture concentrated under reduced pressure, coevaporating with toluene (5mL) three times. The solid was washed with CH_2Cl_2 (20 mL), then suspended in water (30mL) (ultrasonic bath) and the suspension was washed with CH_2Cl_2 (20 mL). The aqueous solution was filtered through plug of cotton and then freeze dried to give 100 mg of crude product as an off white foam.

Sephadex G10 (10g) was poured into hot water (~25 mL, 90dC) and left to swell for 2h; the suspension was allowed to cool to r.t. The suspension was then transferred into a column and left to settle for 30 min (bed height:14 cm; volume: 25 mL). 20 mg sample of the crude product was dissolved in 1.5 mL of water. The slightly cloudy solution was loaded into column, then eluted with water with approximate rate of 1 mL/min. Note that the retention factor of the desired product is close to 1. The presence of dissolved organic material was detected by $KMnO_4$ staining. Product containing fractions were lyophilized to yield the desired monocyclic mercury salt (6 mg, 15% yield) as a white foam.

^{1}H NMR (400 MHz, D_2O) δ 5.65 (d, J = 4.5 Hz, 1H), 5.22 (d, J = 4.5 Hz, 1H), 3.76 (t, J = 6.1 Hz, 1H), 3.70 (d, J = 10.4 Hz, 1H), 2.56 – 2.38 (m, 3H), 2.02 – 1.87 (m, 2H), 1.86 – 1.67 (m, 2H), 1.05 (d, J = 6.5 Hz, 3H), 0.95 (d, J = 6.5 Hz, 3H). Spectroscopic data were in accord with those reported.[11]

Synthesis of enethiol diastereoisomers and hydrated formyl glycine tripeptide aldehyde standard

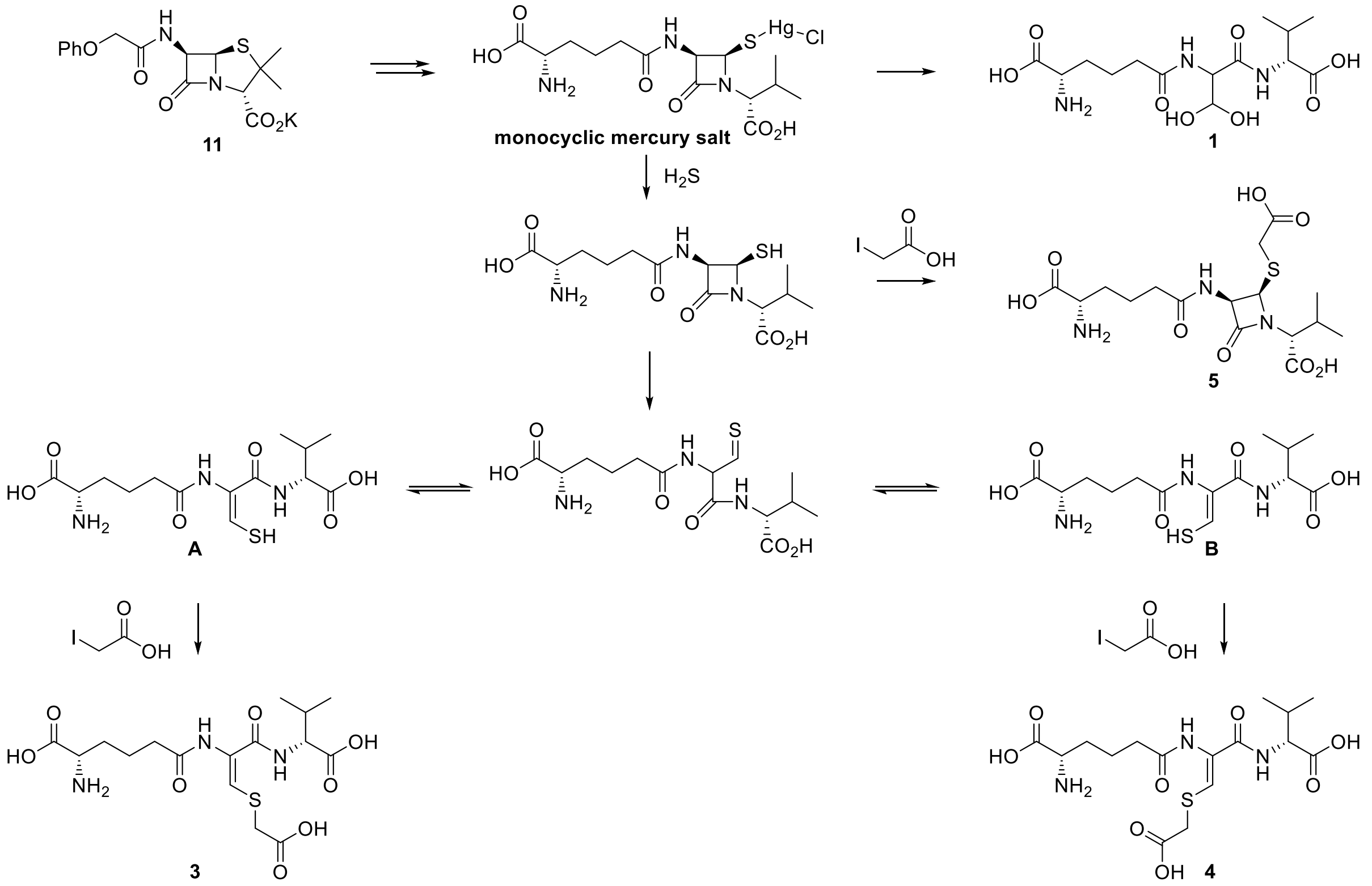

Supplementary Figure 10. Overview scheme of synthesis of enethiol diastereoisomers and hydrated formyl glycine tripeptide aldehyde standard.

The method was adapted from that reported.[12] The monocyclic mercury salt (1 mg, 0.016 mmol) was dissolved in H_2O (0.25 mL); the solution was acidified to pH 2 with dilute HCl. H_2S (0.8 M in THF, 0.032 mmol. 4 µL, 2 eq.) was added resulting in a color change of the solution to black. The reaction mixture was stirred for 1 min. The black precipitant was removed by centrifugation and the remaining liquid was purged with N_2 until residual H_2S and solvent were removed. The remaining pale yellow precipitant was dissolved in 50 mM Tris in D_2O, pD 8 supplemented with iodoacetic acid (0.85 mg, 0.048 mmol, 3eq) and the reaction was followed by NMR. For compound isolation, the reaction mixture was purified by HPLC resulting in both diastereomers of the enethiolds **3** and **4**, the hydrated aldehyde **1** as well as the protected monocyclic intermediate **5**. The compounds were characterized by 1D and 2D NMR using a 700 MHz, 750 MHz and 950 MHz spectrometer.

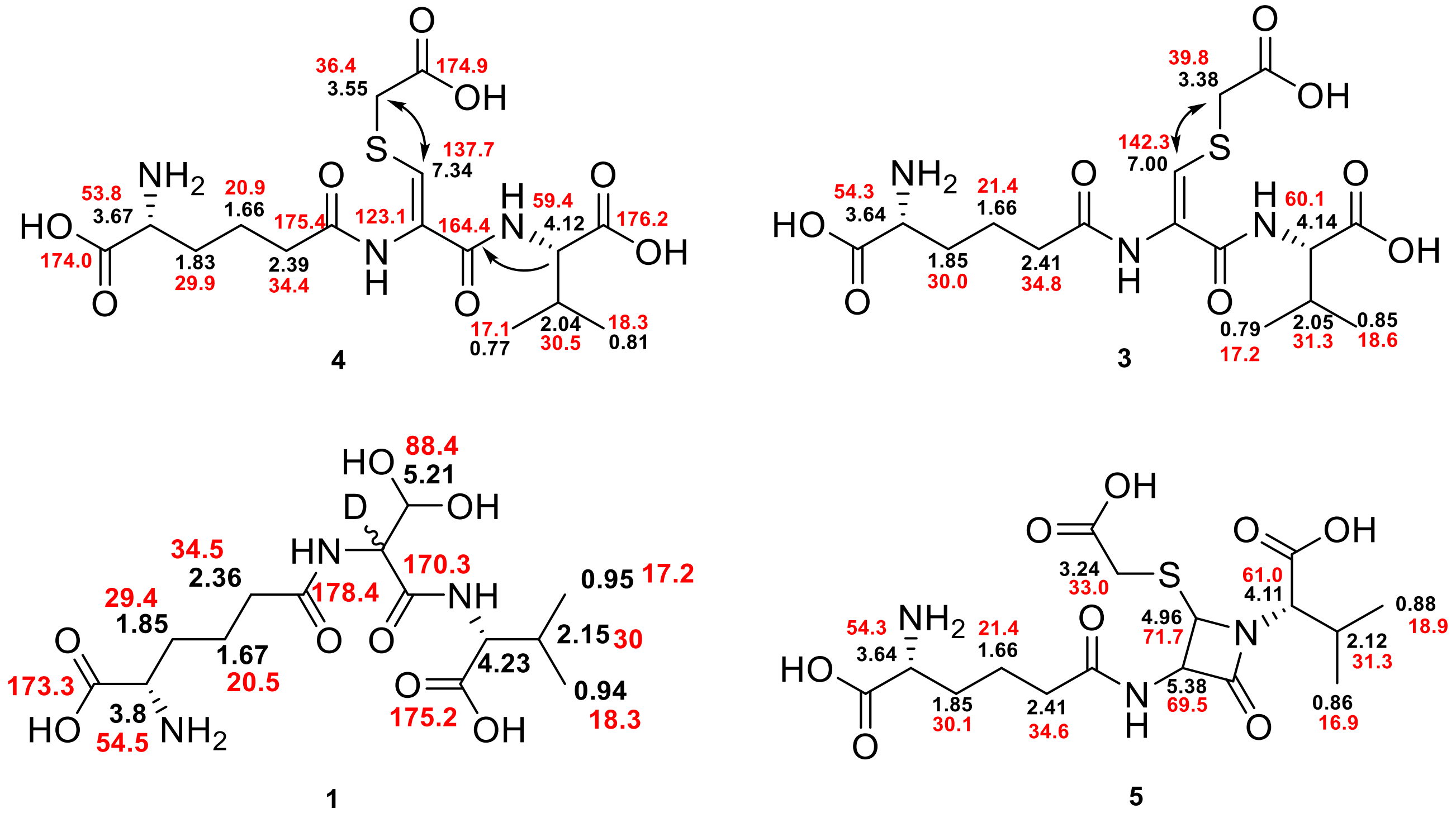

Supplementary Figure 11. Isolated compound characterisation by NMR with ^{1}H and ^{13}C chemical shifts in black and red, respectively.

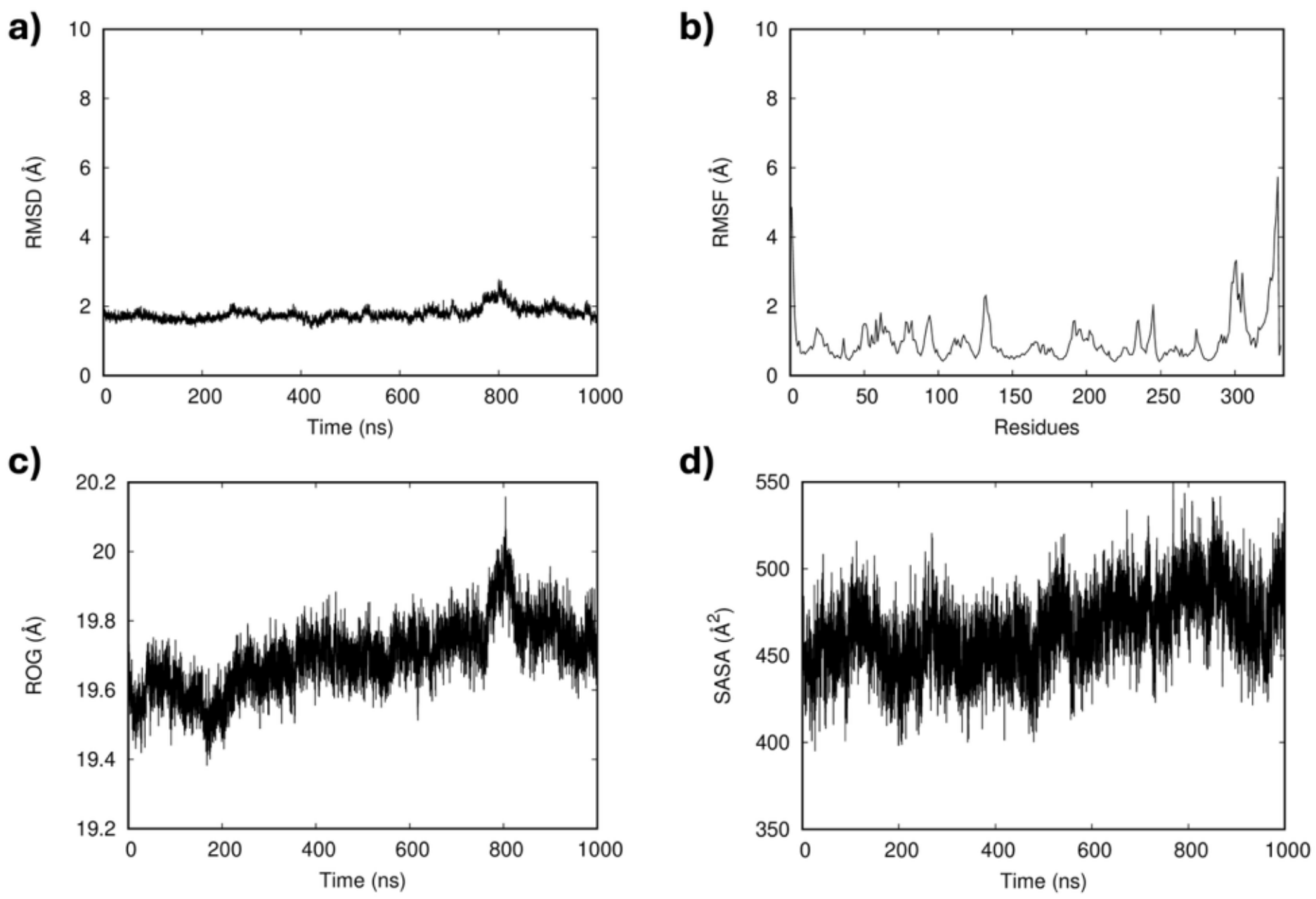

Supplementary Figure 12. MD simulation of the Fe(III)–superoxo complex over 1 µs: (a) root mean square deviation (RMSD) of backbone atoms, (b) root mean square fluctuations (RMSF), (c) radius of gyration (RoG), and (d) solvent-accessible surface area (SASA).

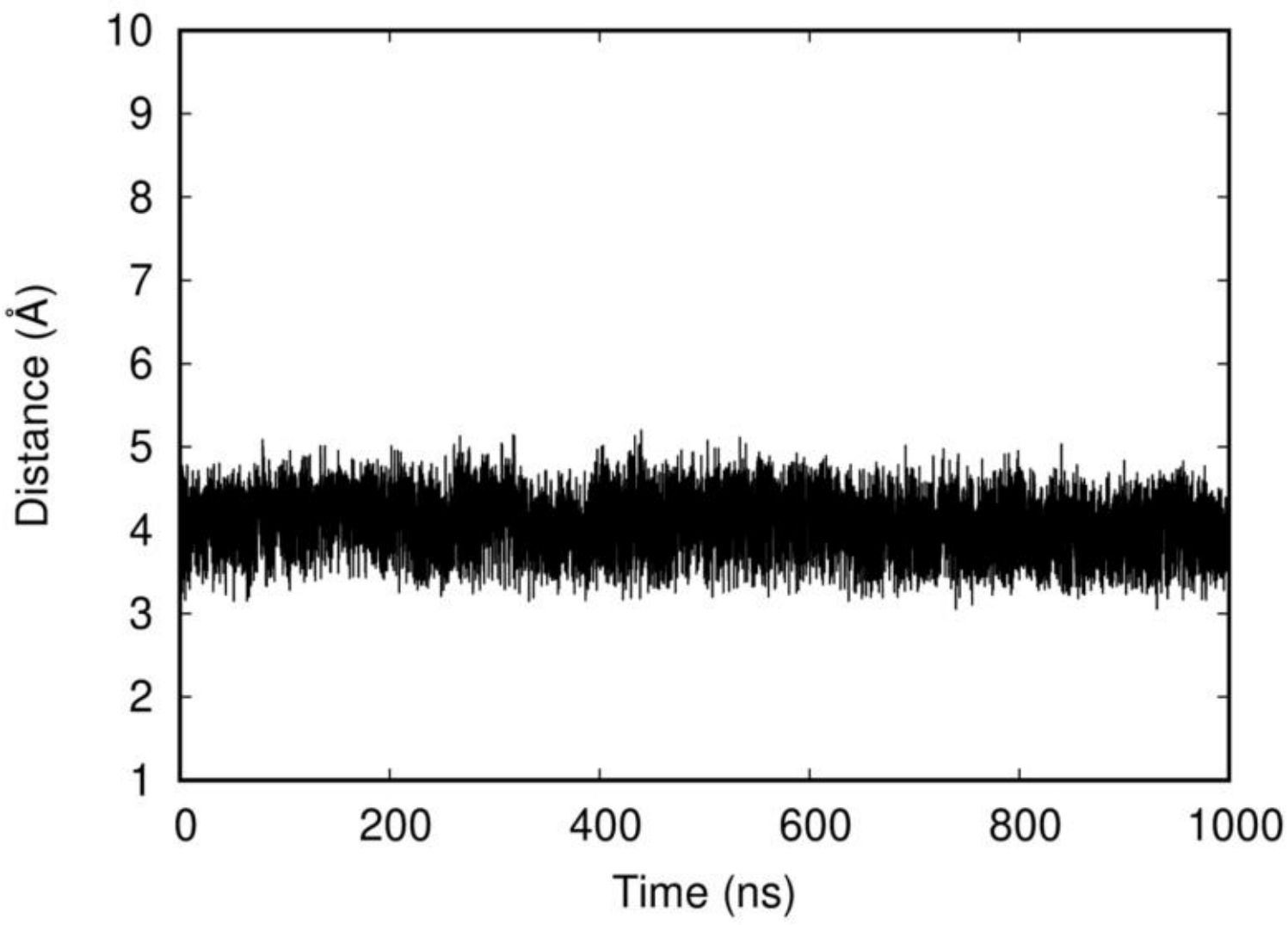

Supplementary Figure 13. Time-dependent fluctuations of the distance between the distal oxygen (Od) and the ACV cysteinyl C3 carbon involved in the hydrogen atom abstraction step during the MD simulation of the Fe(III)-superoxo complex.

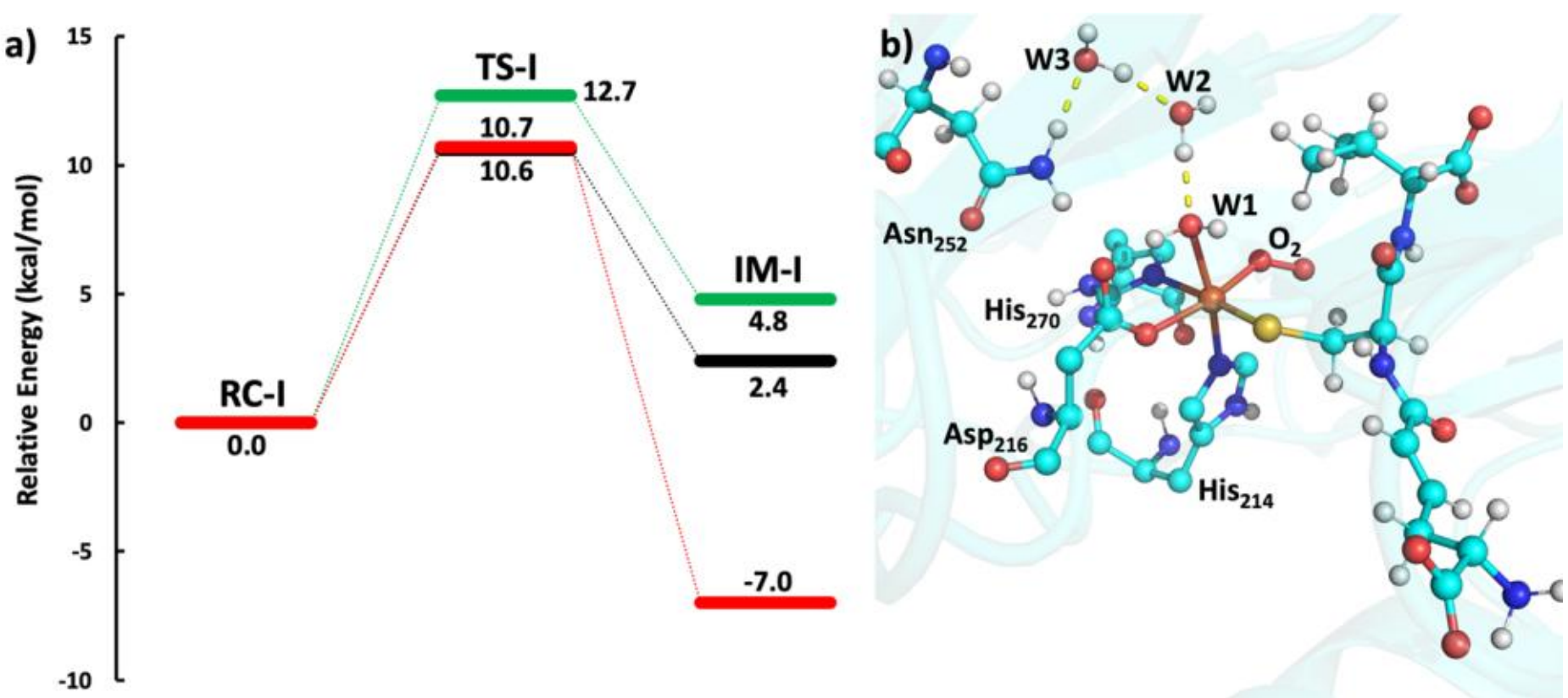

Supplementary Figure 14. (a) Potential energy surface for hydrogen atom transfer from the Fe(III)–superoxo complex (RC-I) leading to formation of the IPNS:Fe(II):HO_2:TAL intermediate (IM-I), comparing (i) standard QM region (black), (ii) extended QM region including W2, W3, and Asn252 (green), and (iii) def2-TZVP basis set (red). (b) Representation of the QM region used in the extended calculations, including W2, W3, and Asn252.

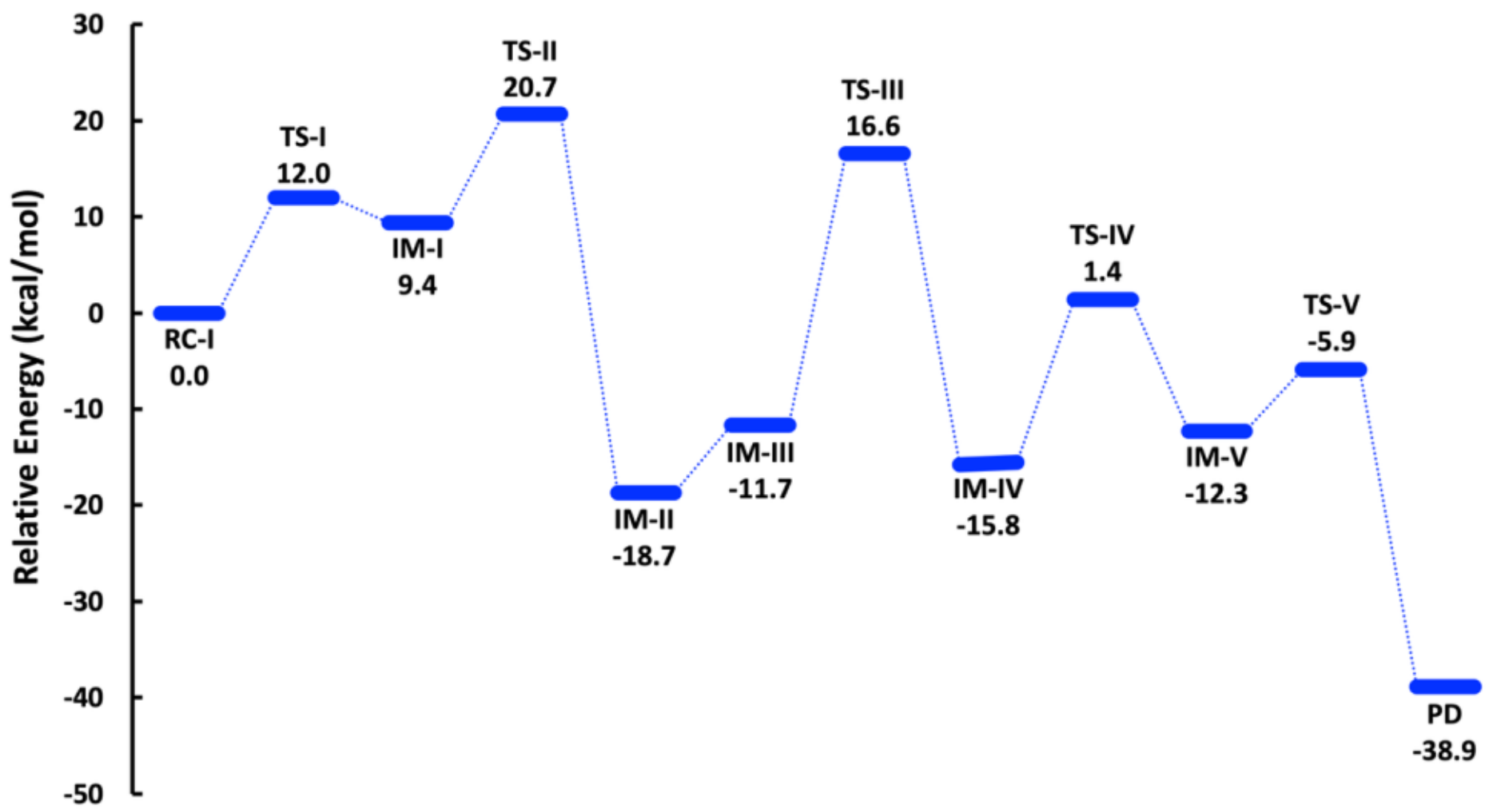

Supplementary Figure 15. Potential energy surface along the reaction coordinate at QM(B2+ZPE (B3)+D3 correction)/MM energy level on the quintet surface, relative energies are given in kcal/mol for snapshot 2.

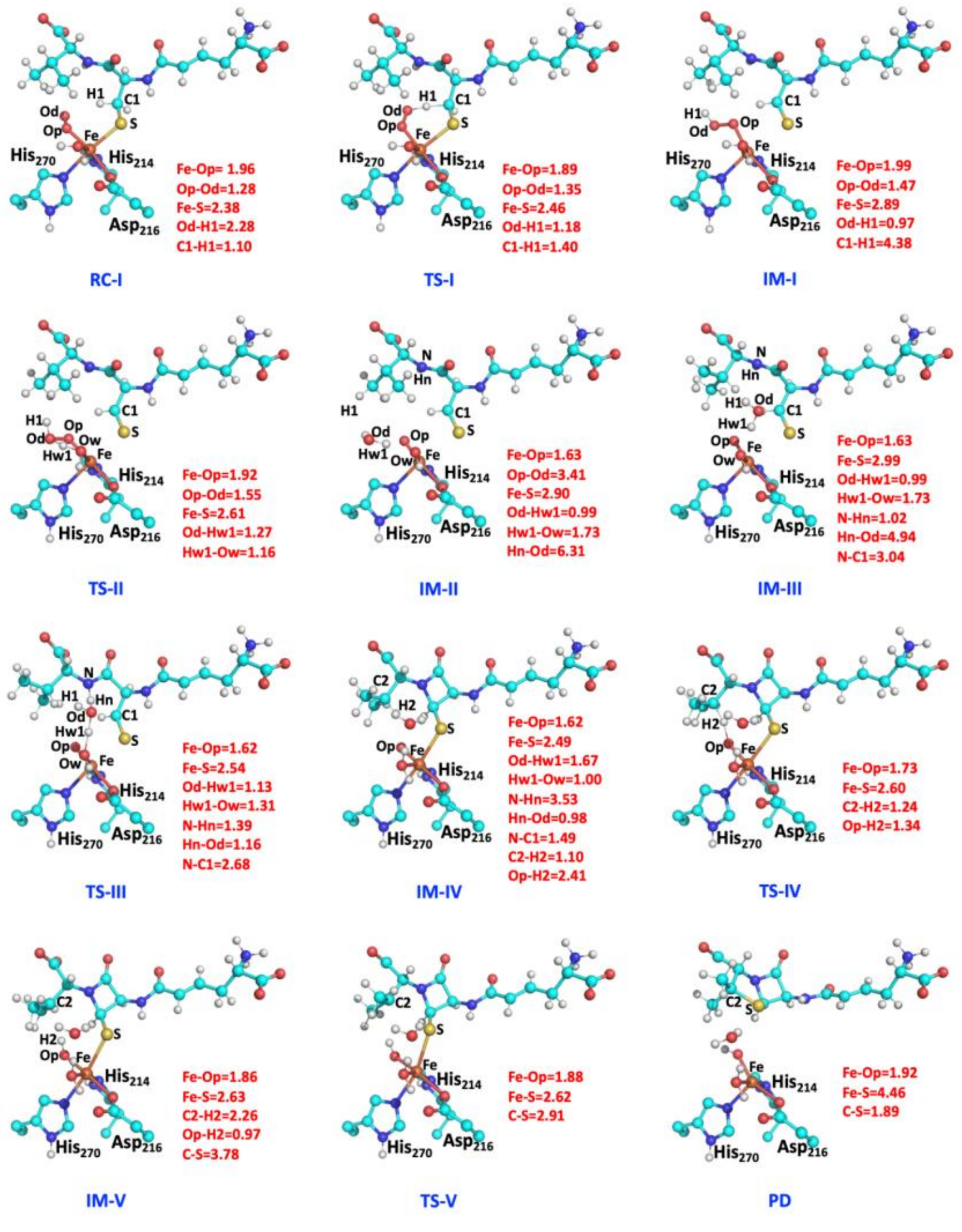

Supplementary Figure 16. QM/MM optimized active-site geometries of selected intermediates and transition states (bond lengths in Å) for snapshot 2. Note that in the product (PD) complex IPN dissociates.

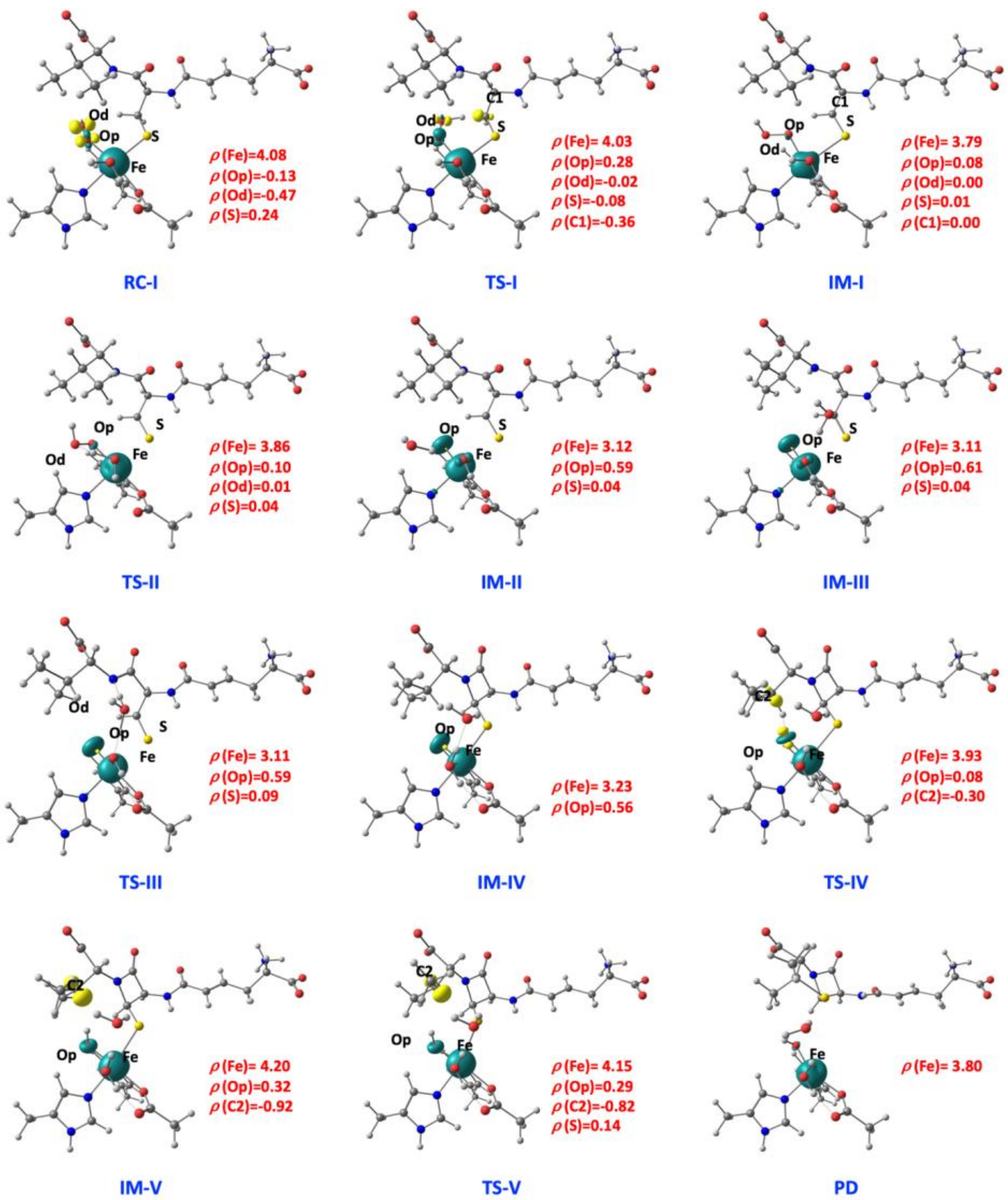

Supplementary Figure 17. Spin density plots with corresponding spin densities (in brackets) for the transition states and intermediates formed during IPNS-catalyzed reactions from RC-I for snapshot 2.

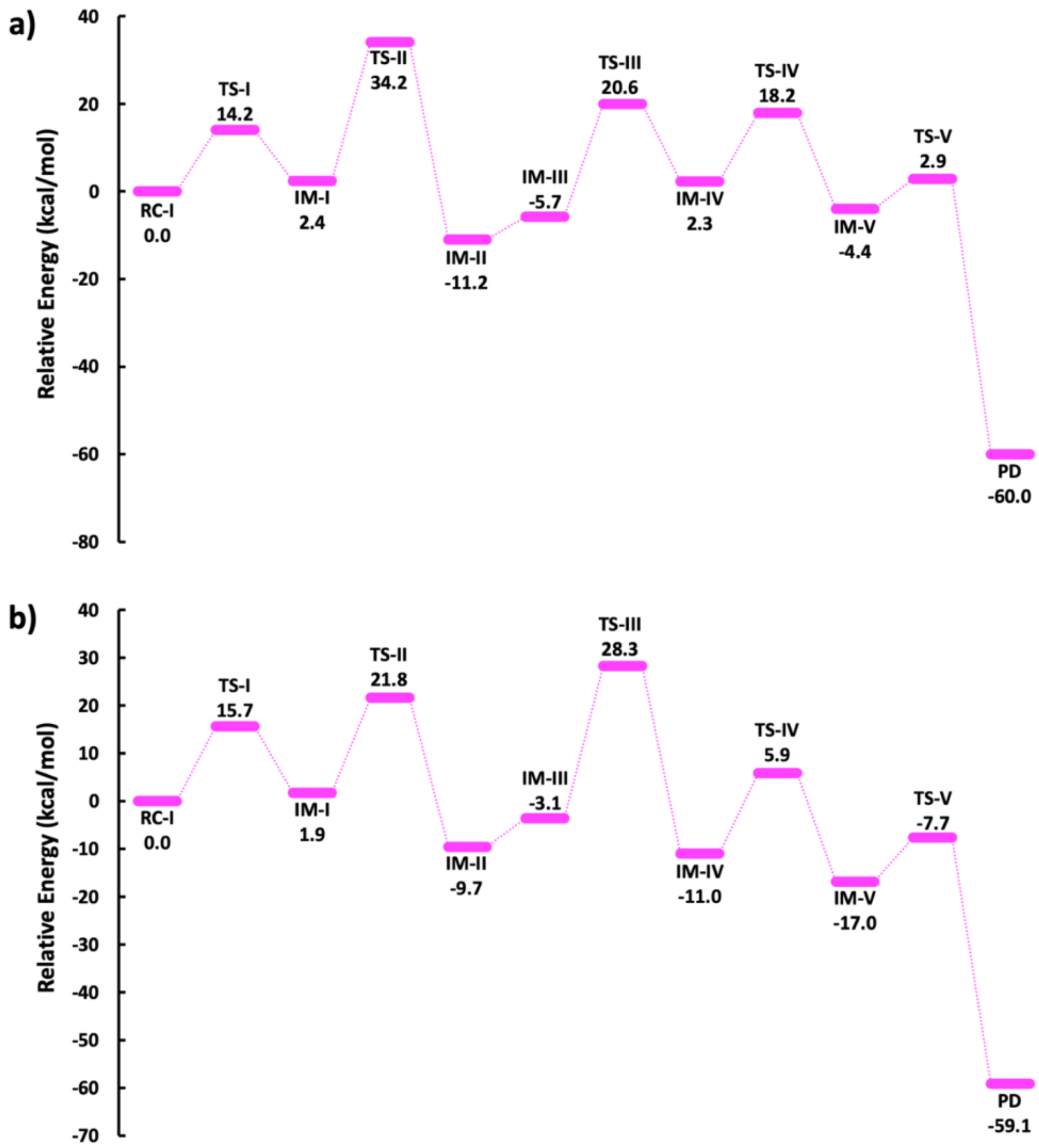

Supplementary Figure 18. Potential energy surface along the reaction coordinate computed using single-point calculations with the PBE0 functional on the quintet surface. Relative energies are reported in kcal/mol for (a) snapshot 1 and (b) snapshot 2.

(((9H-Fluoren-9-yl)methoxy)carbonyl)valine-3-^{2}H (8) [1]

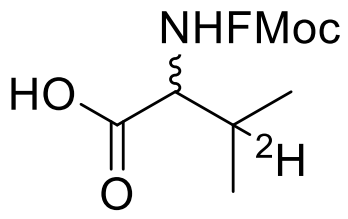

^{1}H NMR (600 MHz, 300 K, DMSO-d_6)

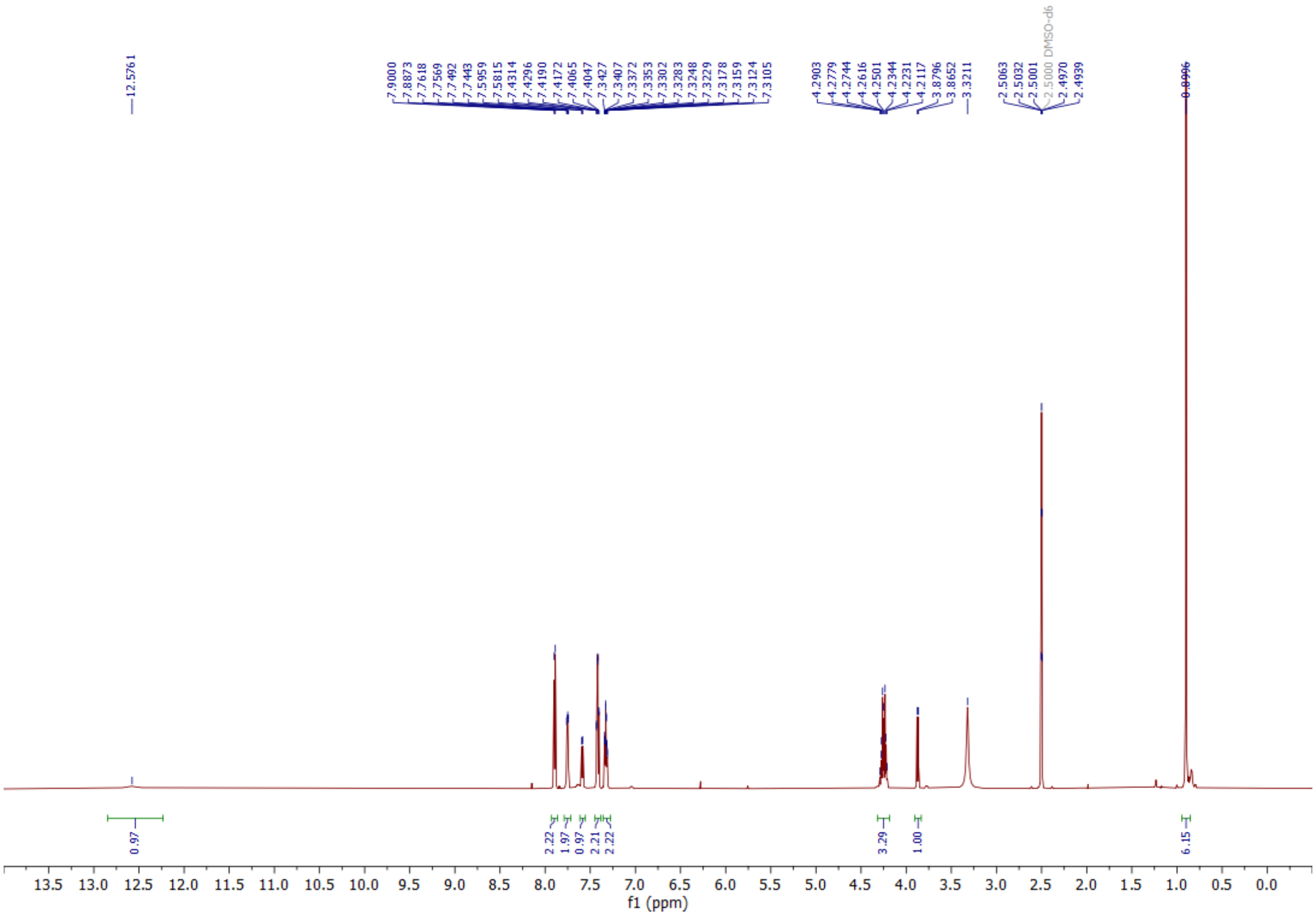

^{13}C NMR (151 MHz, 300 K, DMSO-d_6)

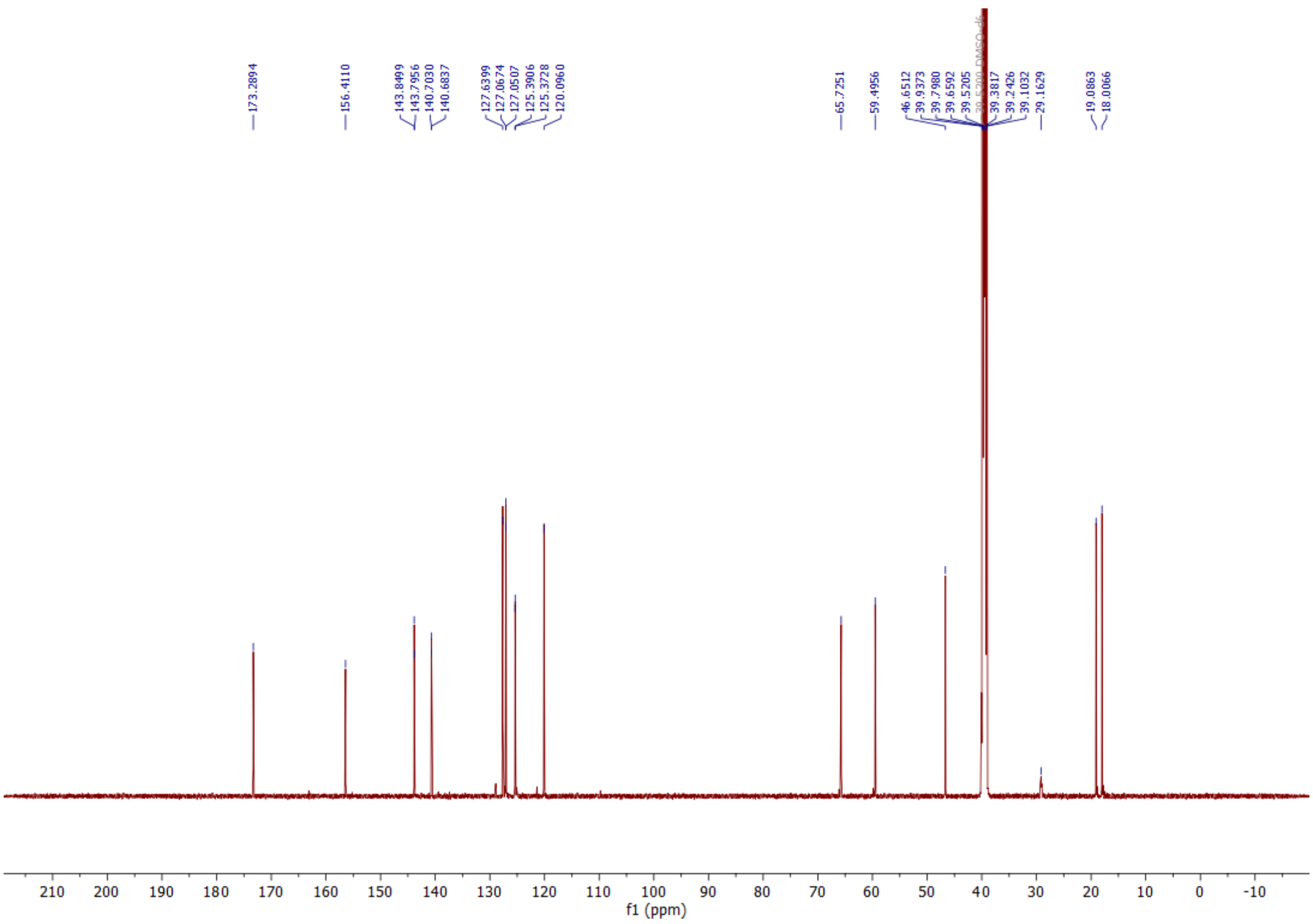

(((9*H*-Fluoren-9-yl)methoxy)carbonyl)valine-^{15}*N* (10)

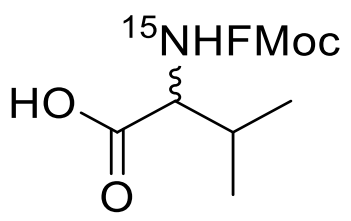

^{1}H NMR (700 MHz, 300 K, DMSO-d_6)

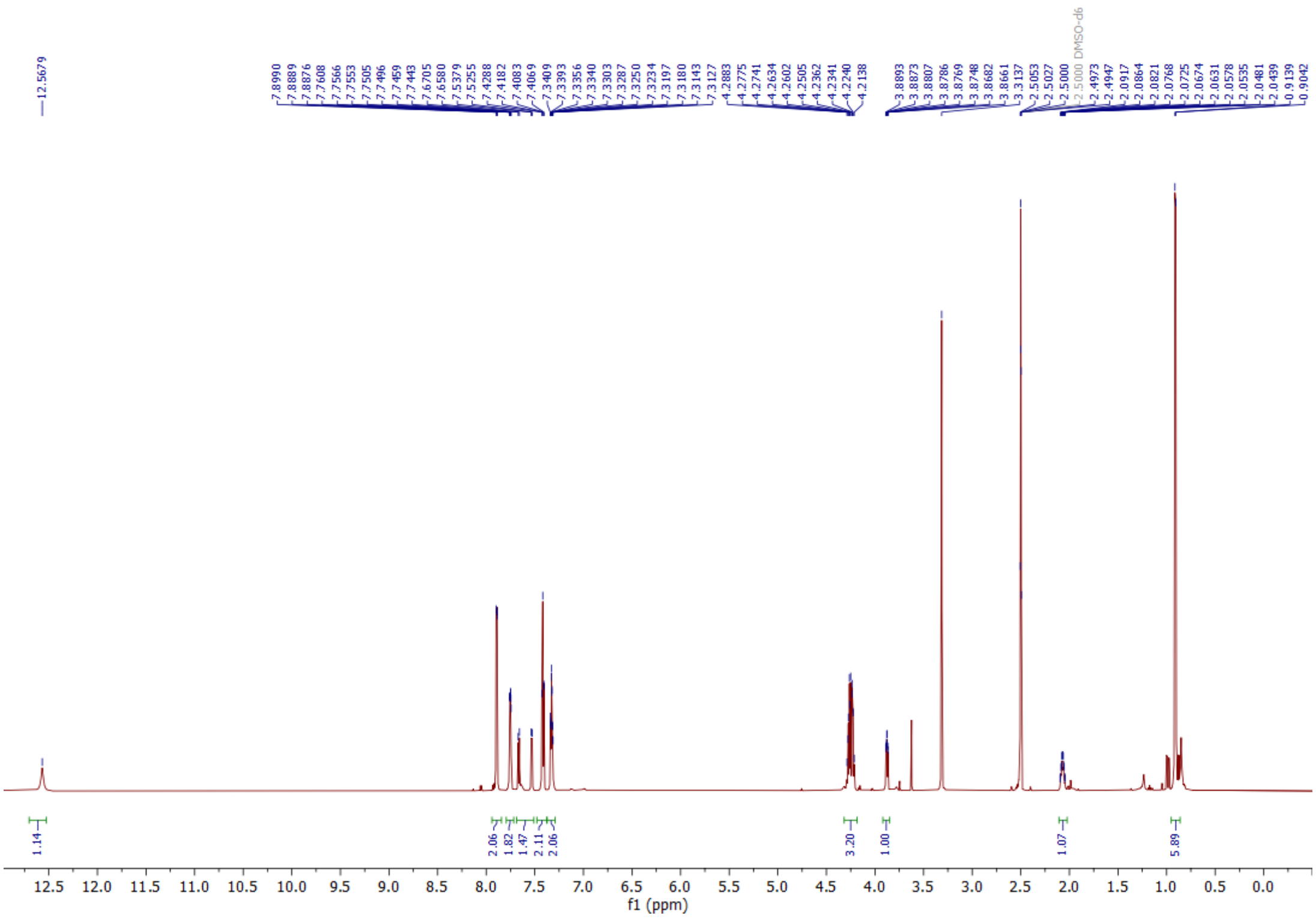

^{13}C NMR (176 MHz, 300 K, DMSO-d_6)

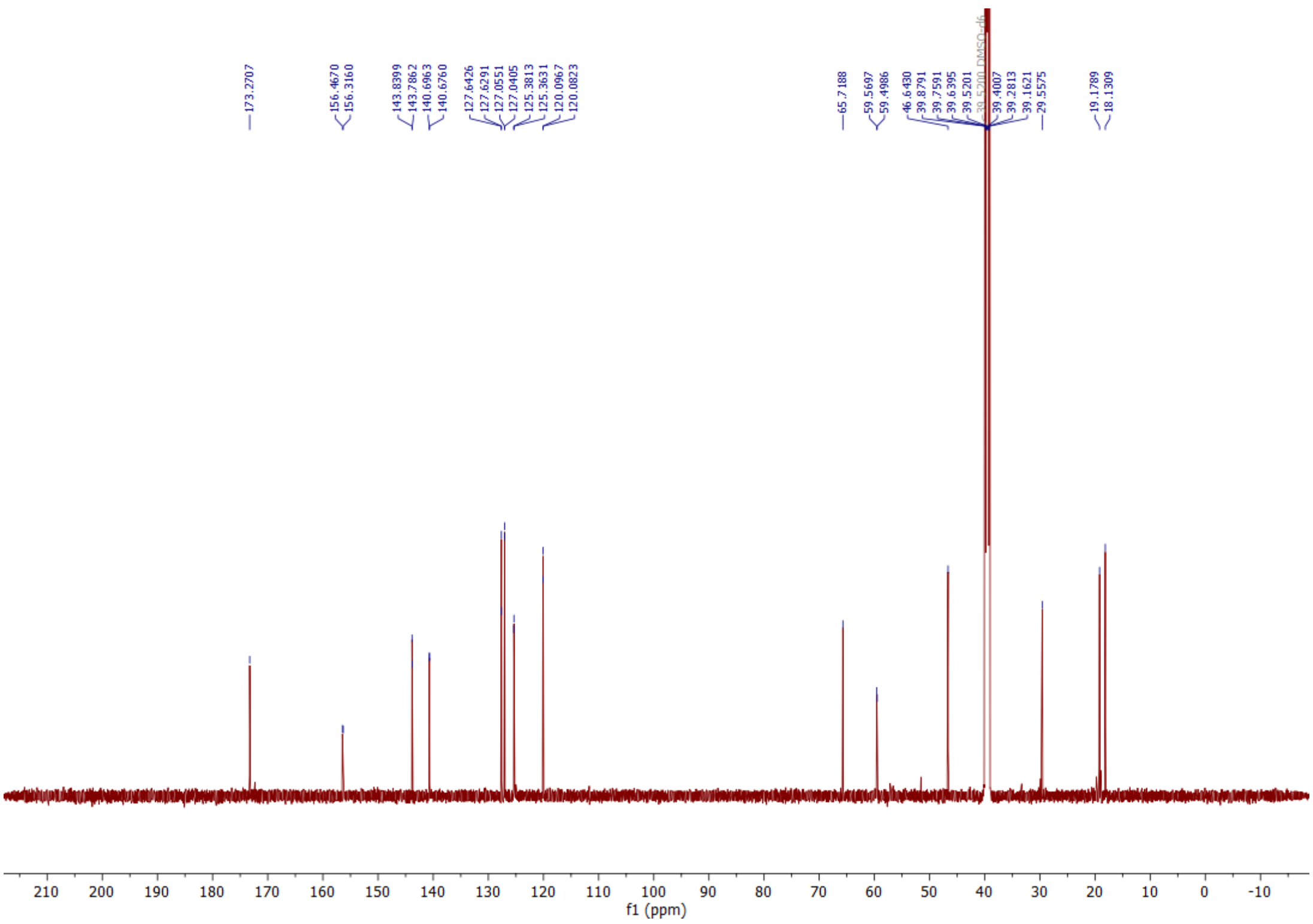

Labelled ACV (*S*)-2-amino-6-(((*R*)-1-(((*R*)-1-carboxy-2-methylpropyl)amino-^{15}N)-3-mercapto-1-oxopropan-2-yl-1,2,3-$^{13}C_3$)amino-^{15}N)-6-oxohexanoic acid (($^{13}C_3$,$^{15}N_2$)ACV)

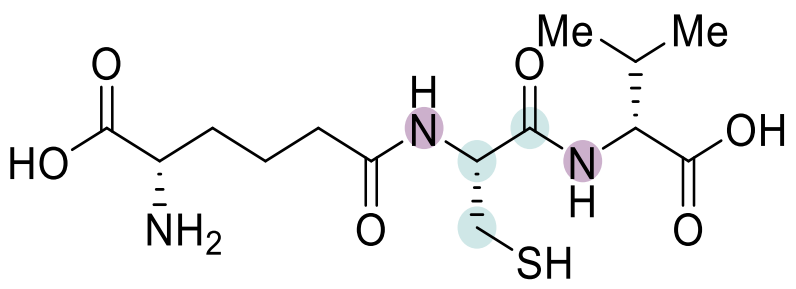

^{1}H NMR (700 MHz, 300 K, D_2O)

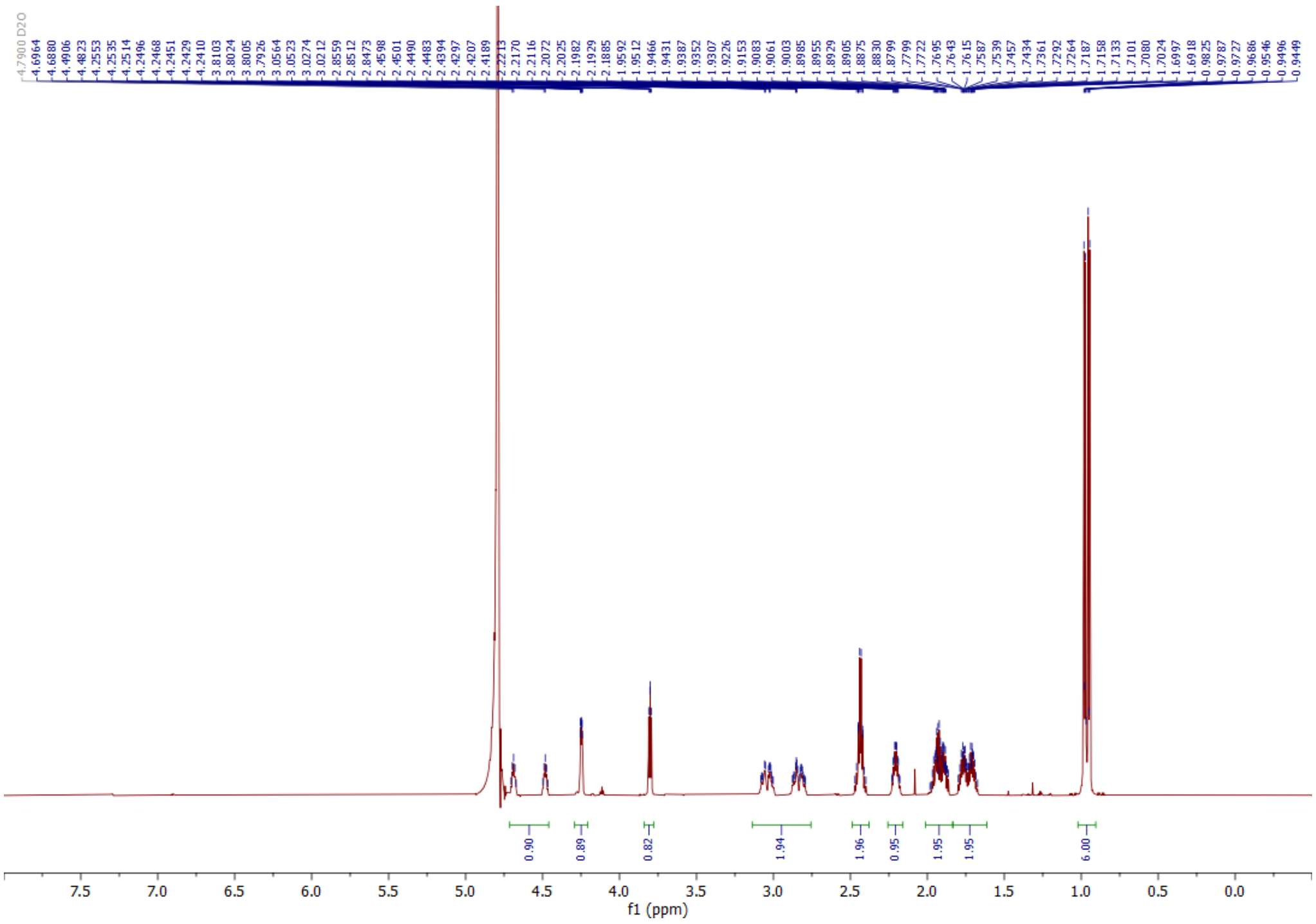

^{1}H NMR (^{13}C,^{15}N decoupled) (700 MHz, 300 K, D_2O)

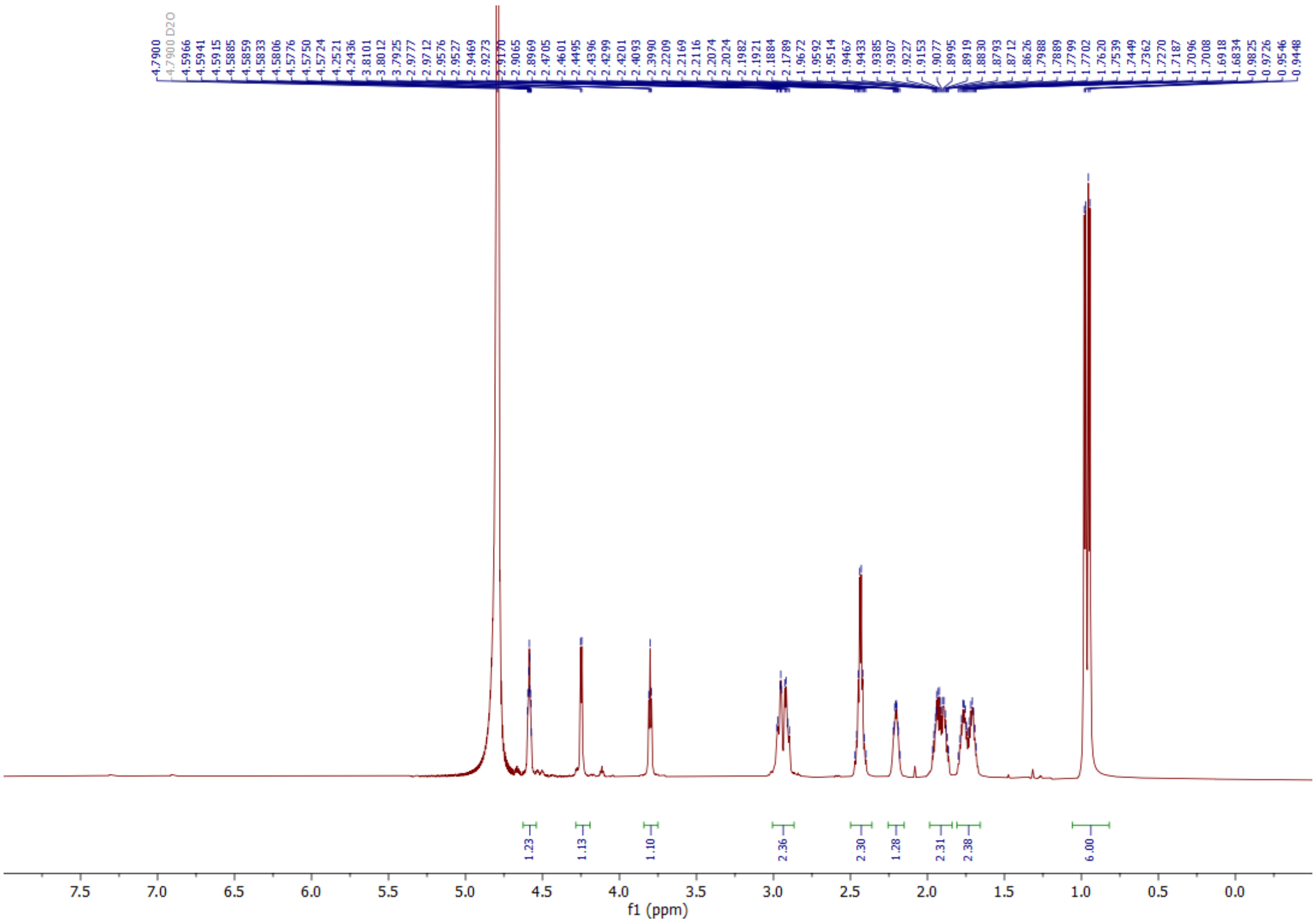

^{13}C NMR (176 MHz, 300 K, D_2O)

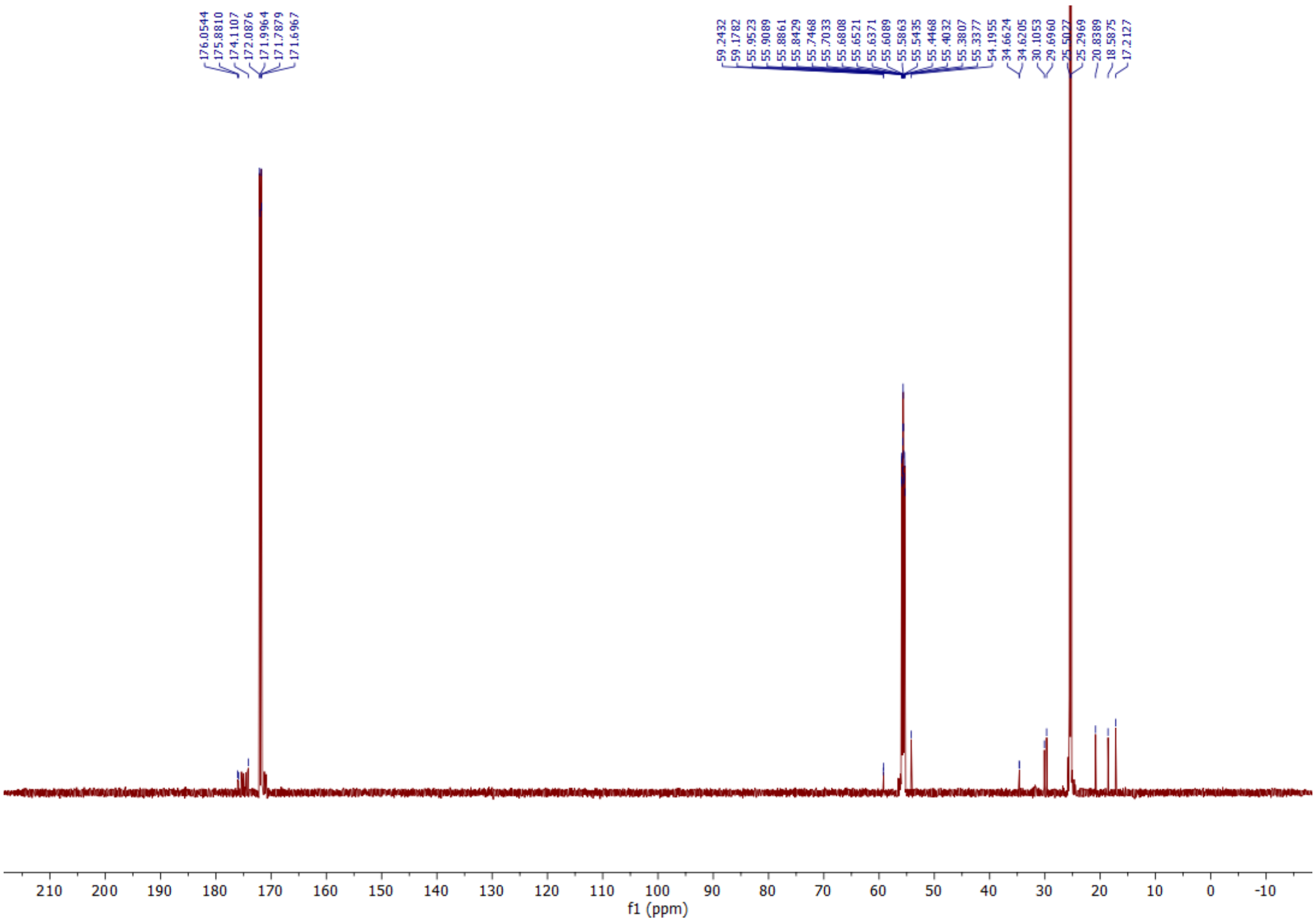

^{1}H NMR spectrum (750 MHz, D_2O) of the S-alkylated enethiol (**4**).

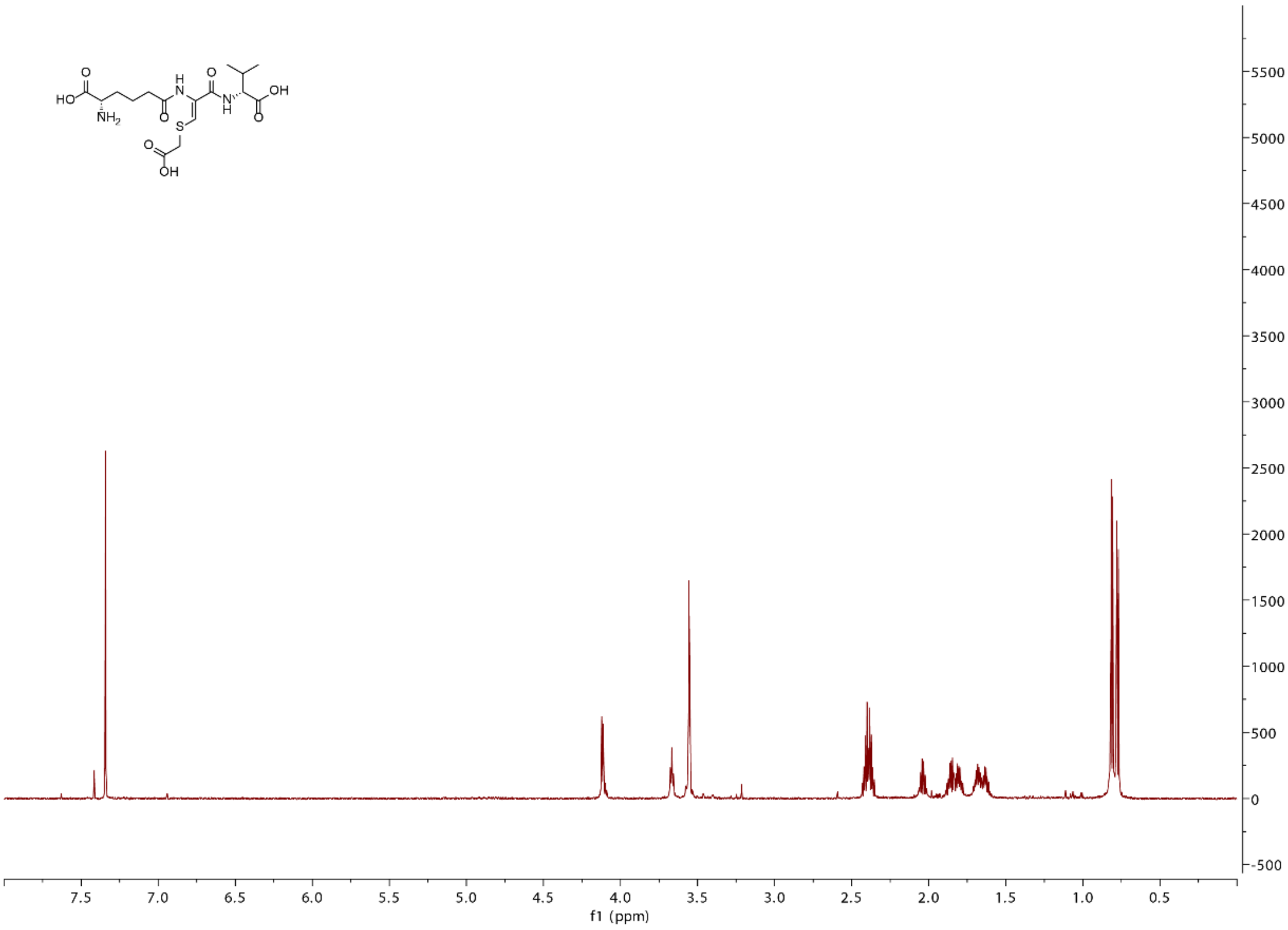

^{1}H,^{1}H COSY NMR spectrum (700 MHz, D_2O) of the S-alkylated enethiol (**4**).

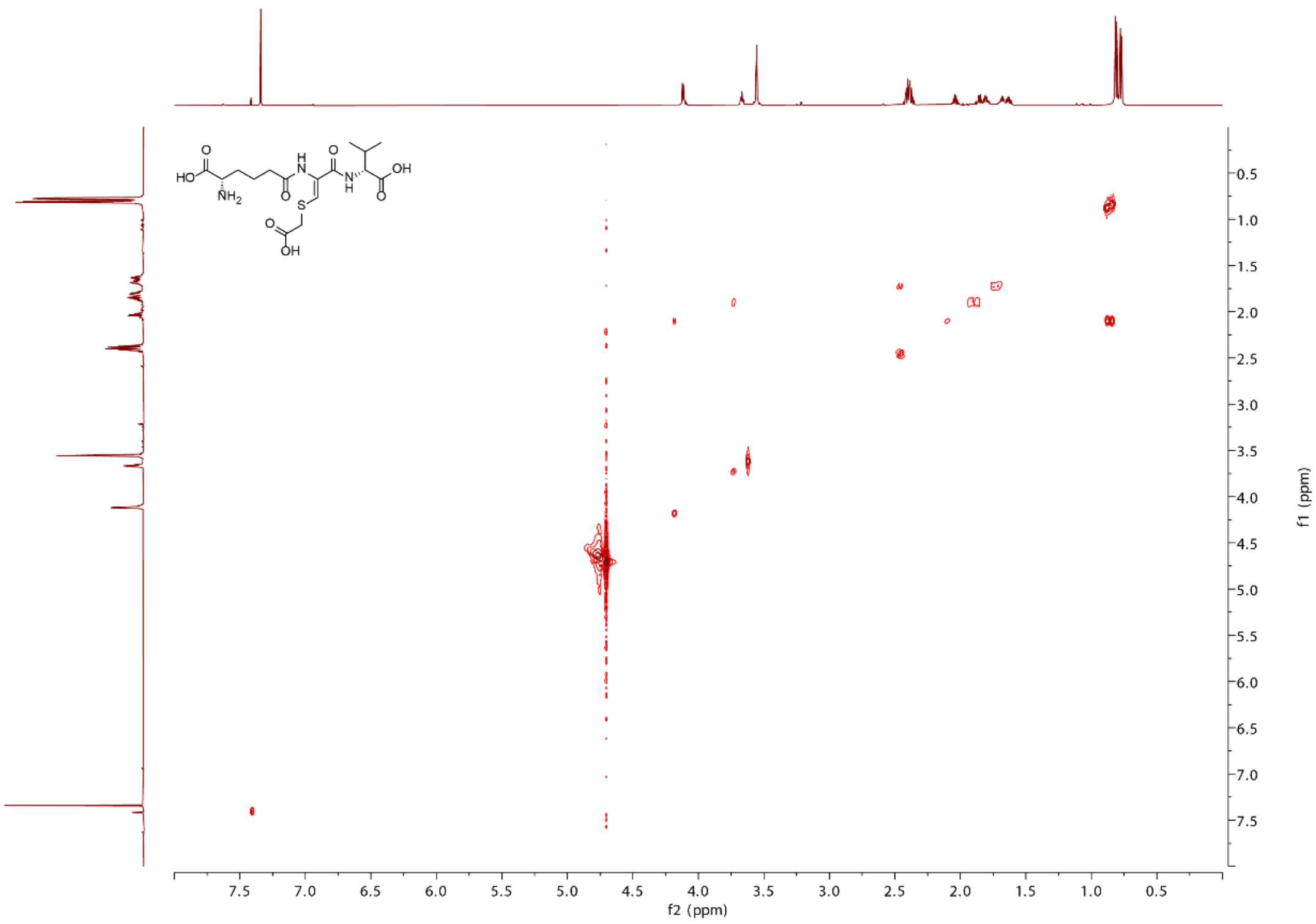

$^{1}H,^{13}C$ HSQC NMR spectrum (750 MHz, D_2O) of the S-alkylated enethiol (**4**).

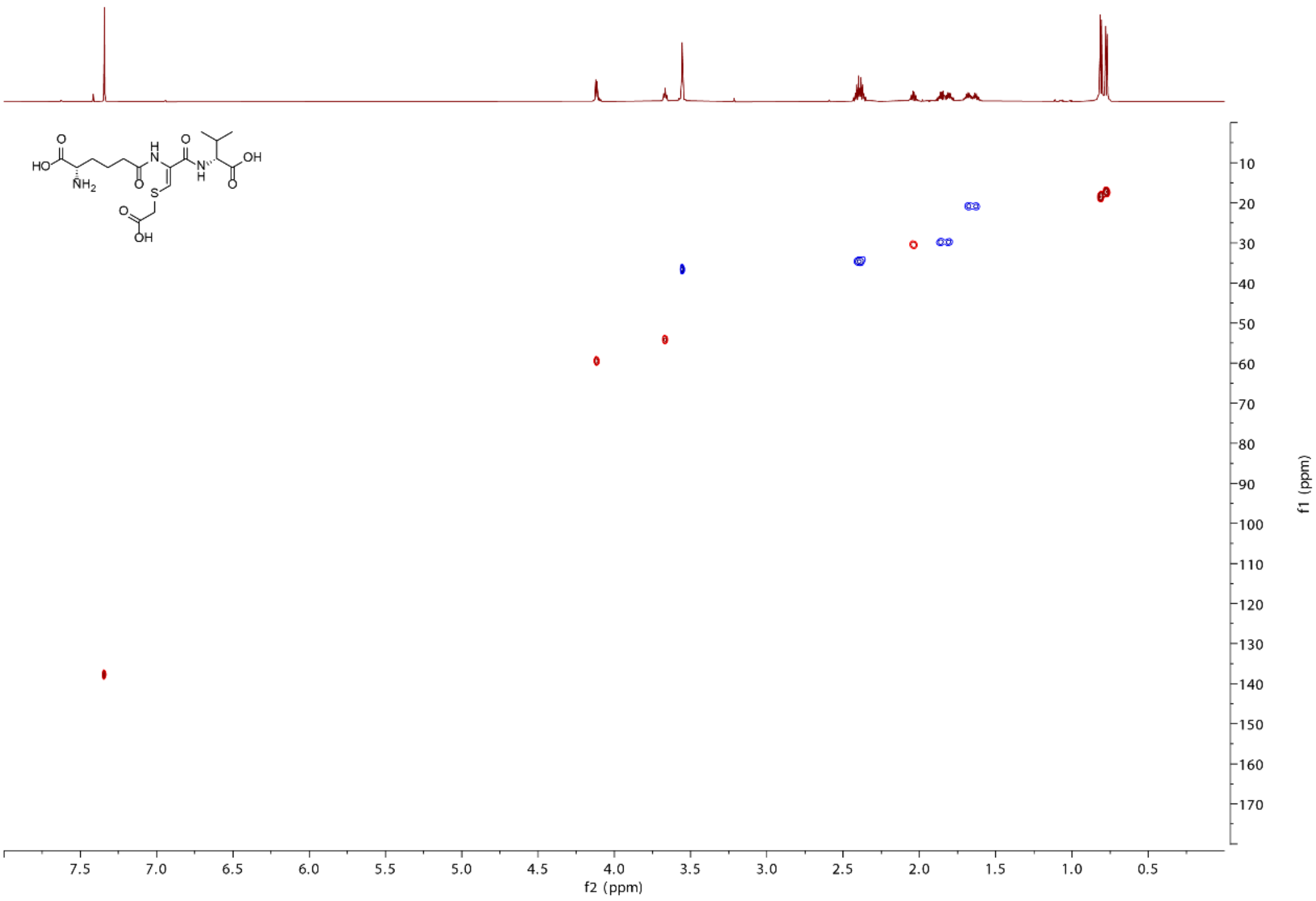

$^{1}H,^{13}C$ HMBC NMR spectrum (750 MHz, D_2O) of the S-alkylated enethiol (**4**).

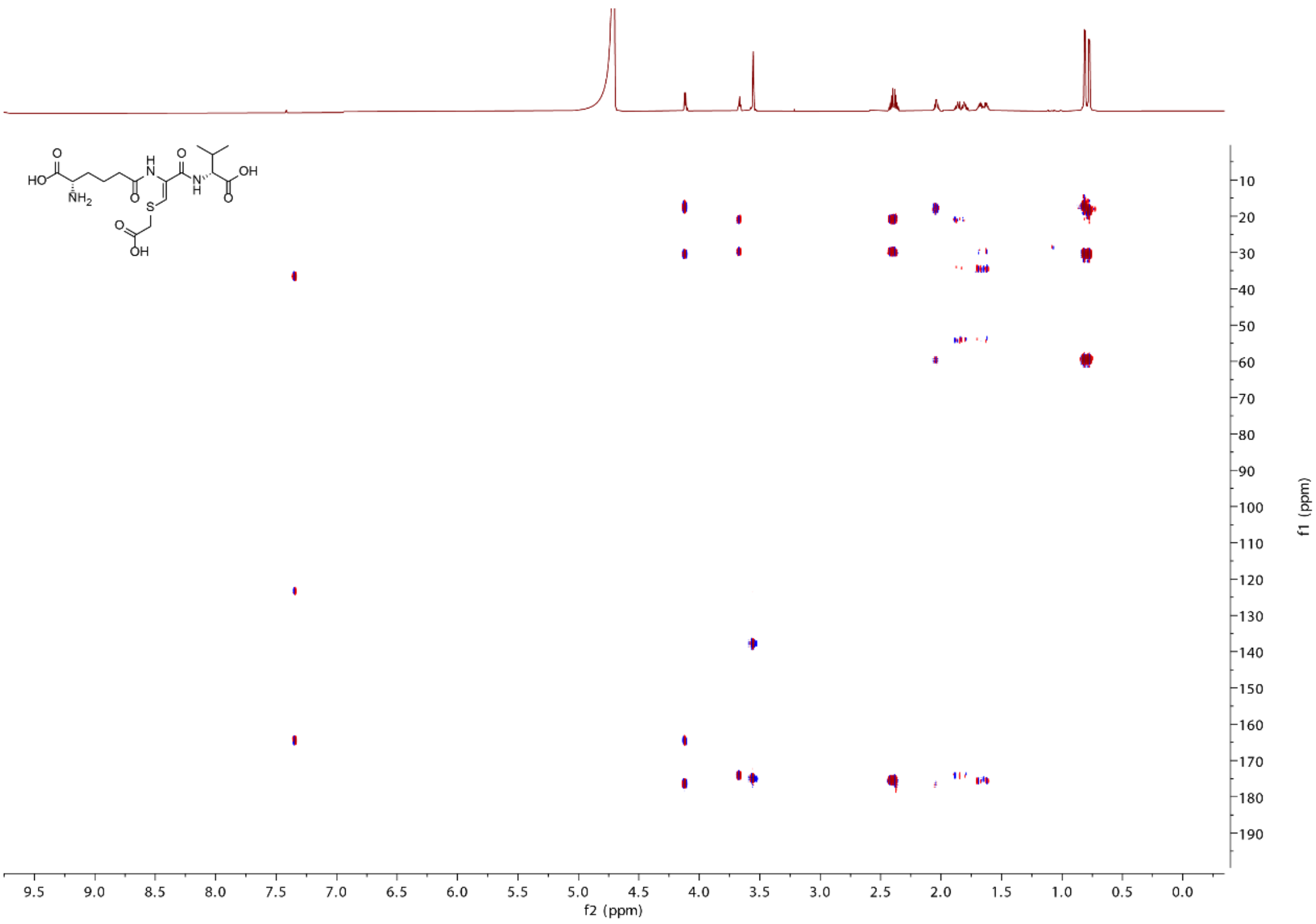

Selective 1D NOE NMR spectrum (700 MHz, D_2O) of the singlet at 3.55 ppm of enethiol (**4**) revealing clear NOE with singlet at 7.34 ppm supporting S-alkylation of enethiol-S.

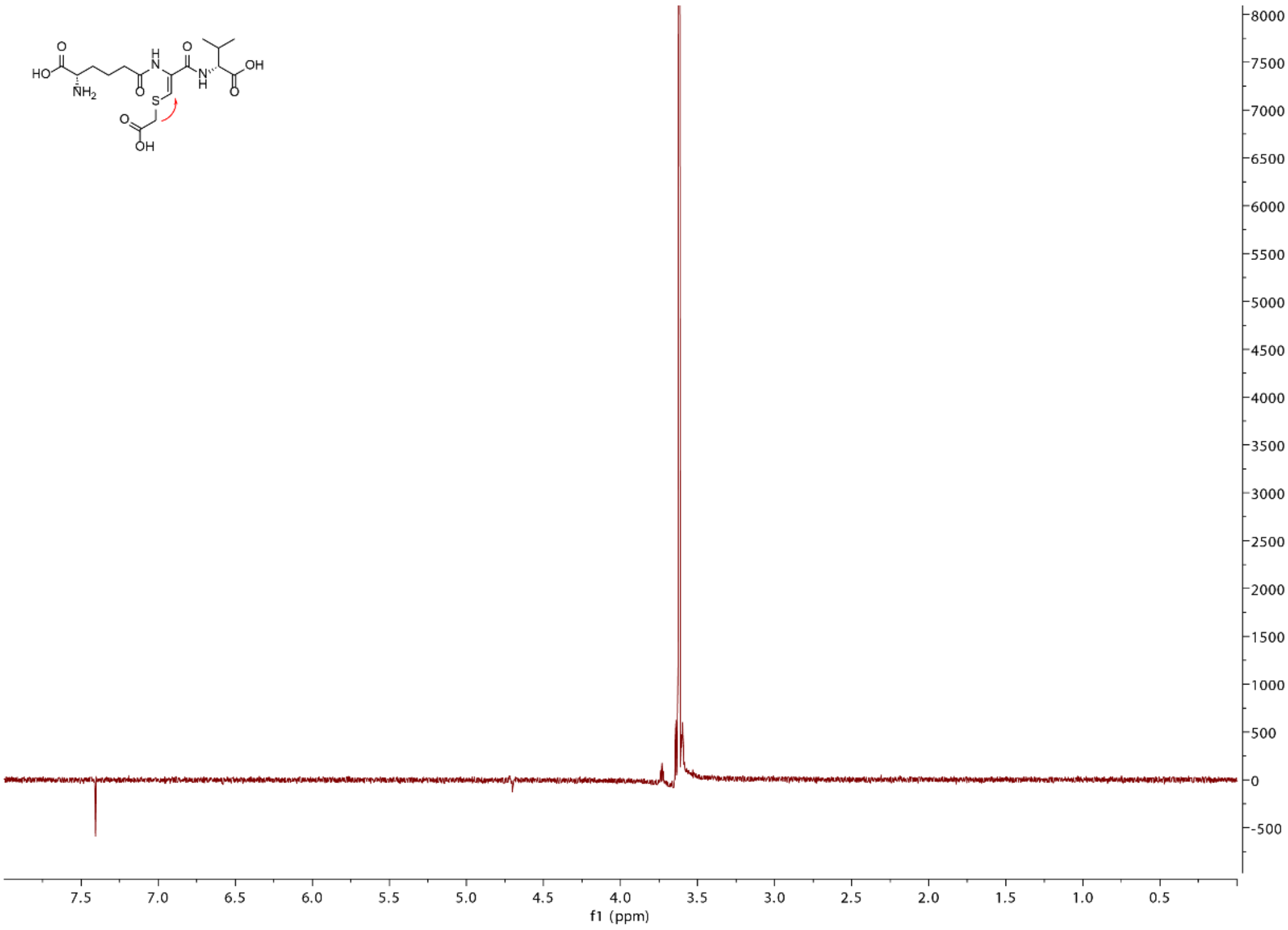

^{1}H NMR spectrum (700 MHz, D_2O) of the enethiol (**3**) and the S-alkylated monocyclic intermediate (**5**).

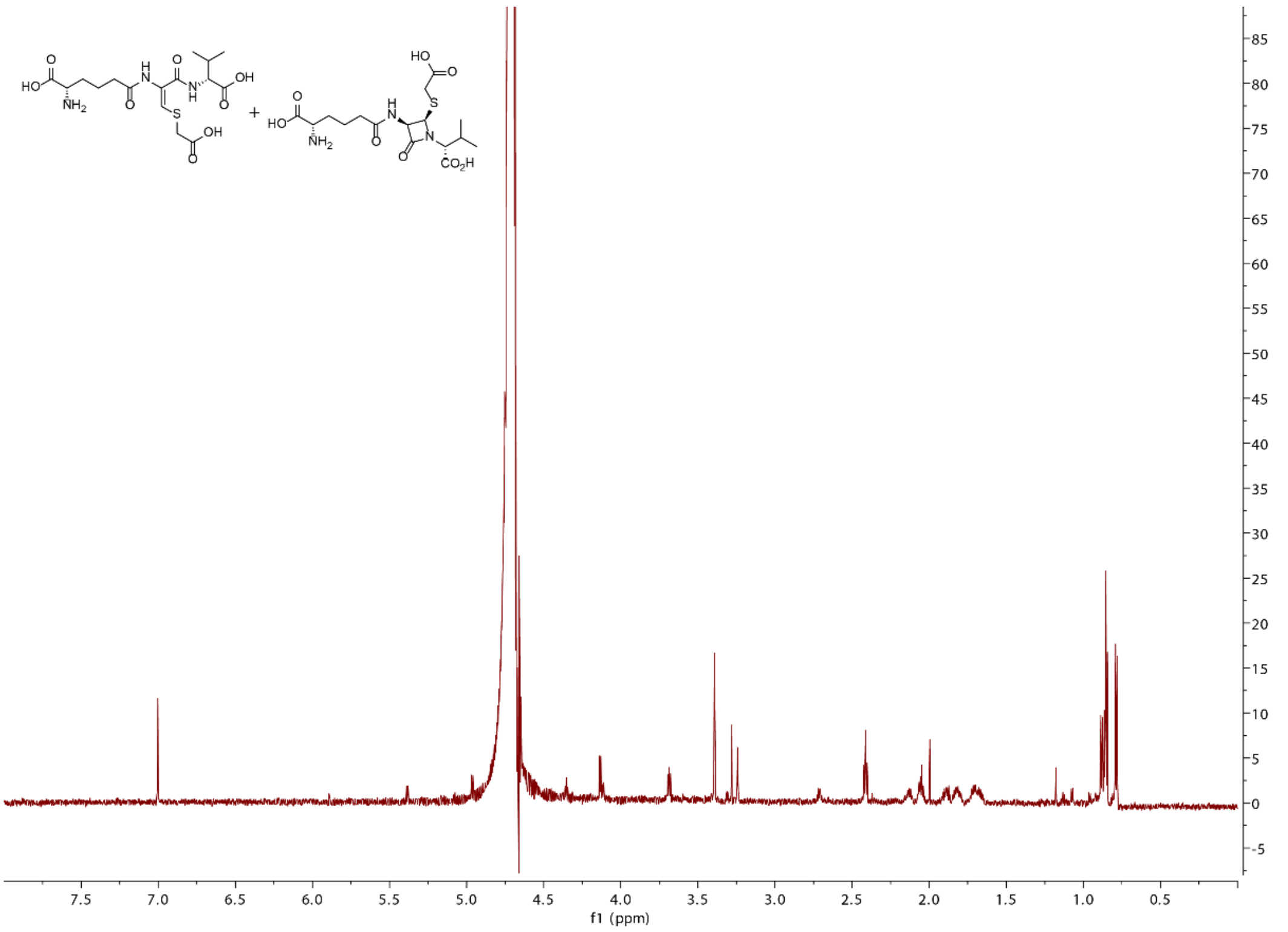

$^{1}H,^{1}H$ COSY NMR spectrum (700 MHz, D_2O) of the S-alkylated enethiol (**3**) and the S-alkylated monocyclic intermediate (**5**).

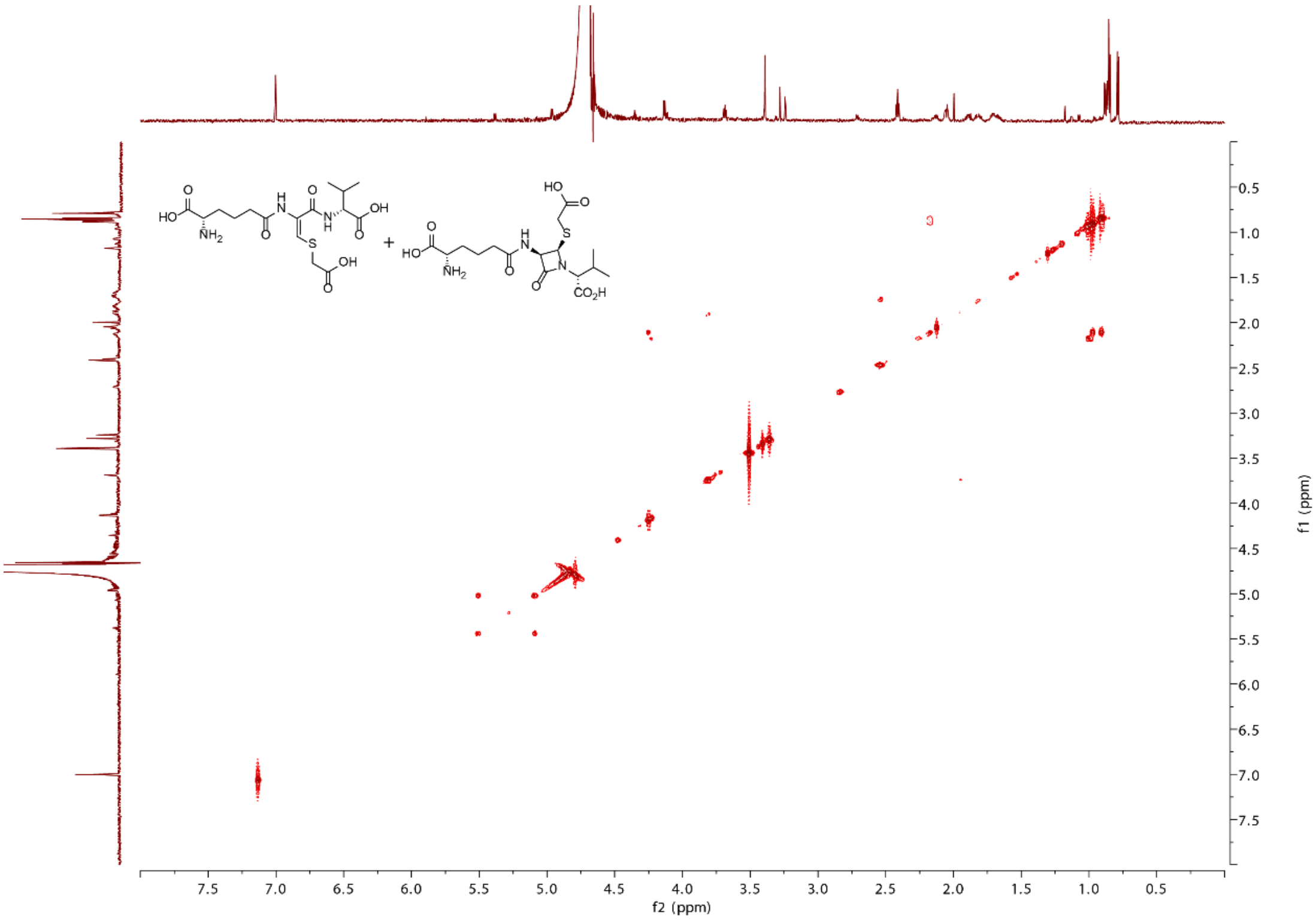

$^{1}H,^{1}H$ TOCSY NMR spectrum (700 MHz, D_2O) of the S-alkylated enethiol (**3**) and the S-alkylated monocyclic intermediate (**5**).

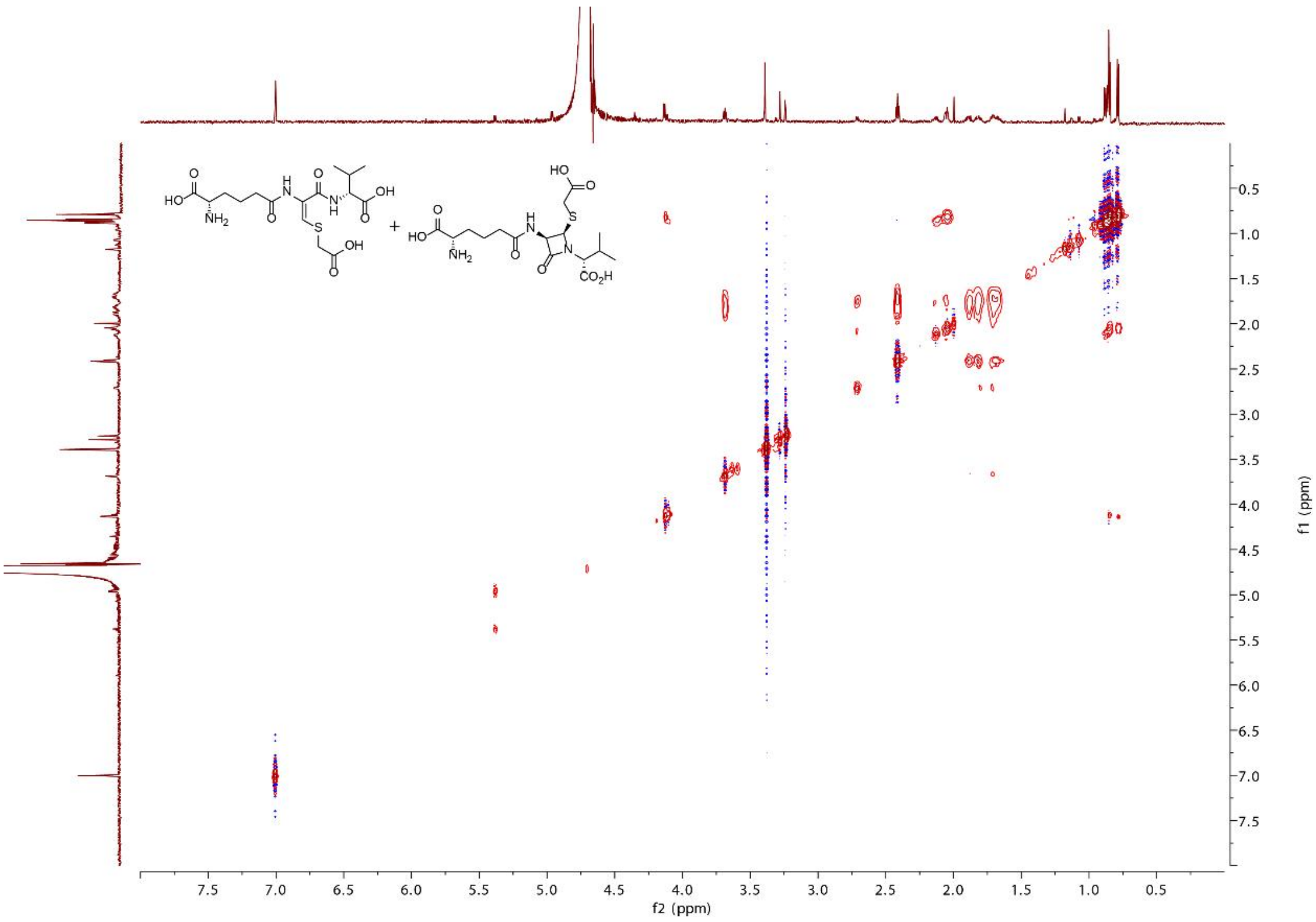

^{1}H,^{13}C HSQC NMR spectrum (700 MHz, D_2O) of the S-alkylated enethiol (**3**) and the S-alkylated monocyclic intermediate (**5**).

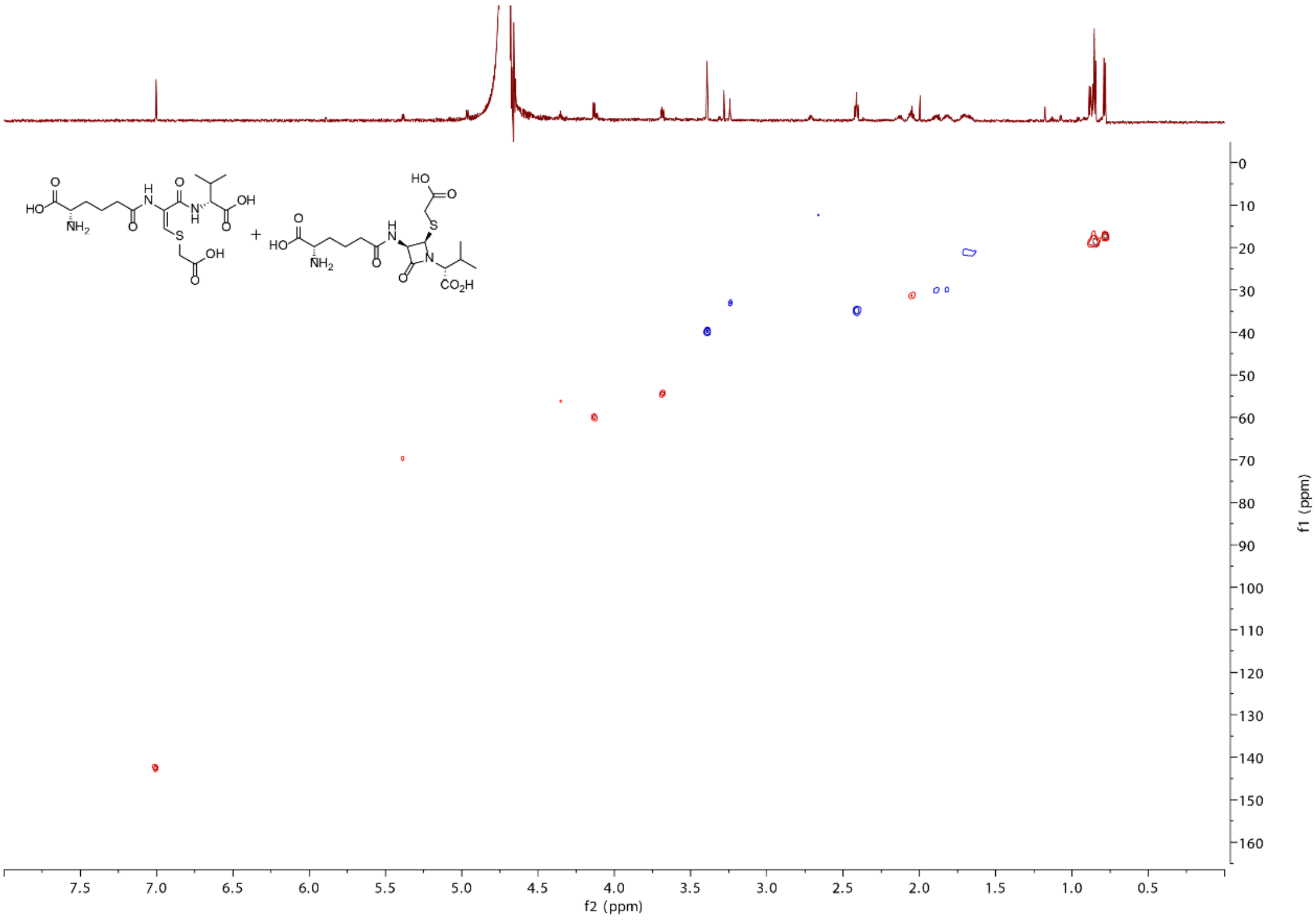

Selective 1D NOE NMR spectrum (700 MHz, D_2O) of the singlet at 3.37 ppm of enethiol (**3**) revealing clear NOE with singlet at 7.00 ppm supporting alkylation of the enethiol-*S*.

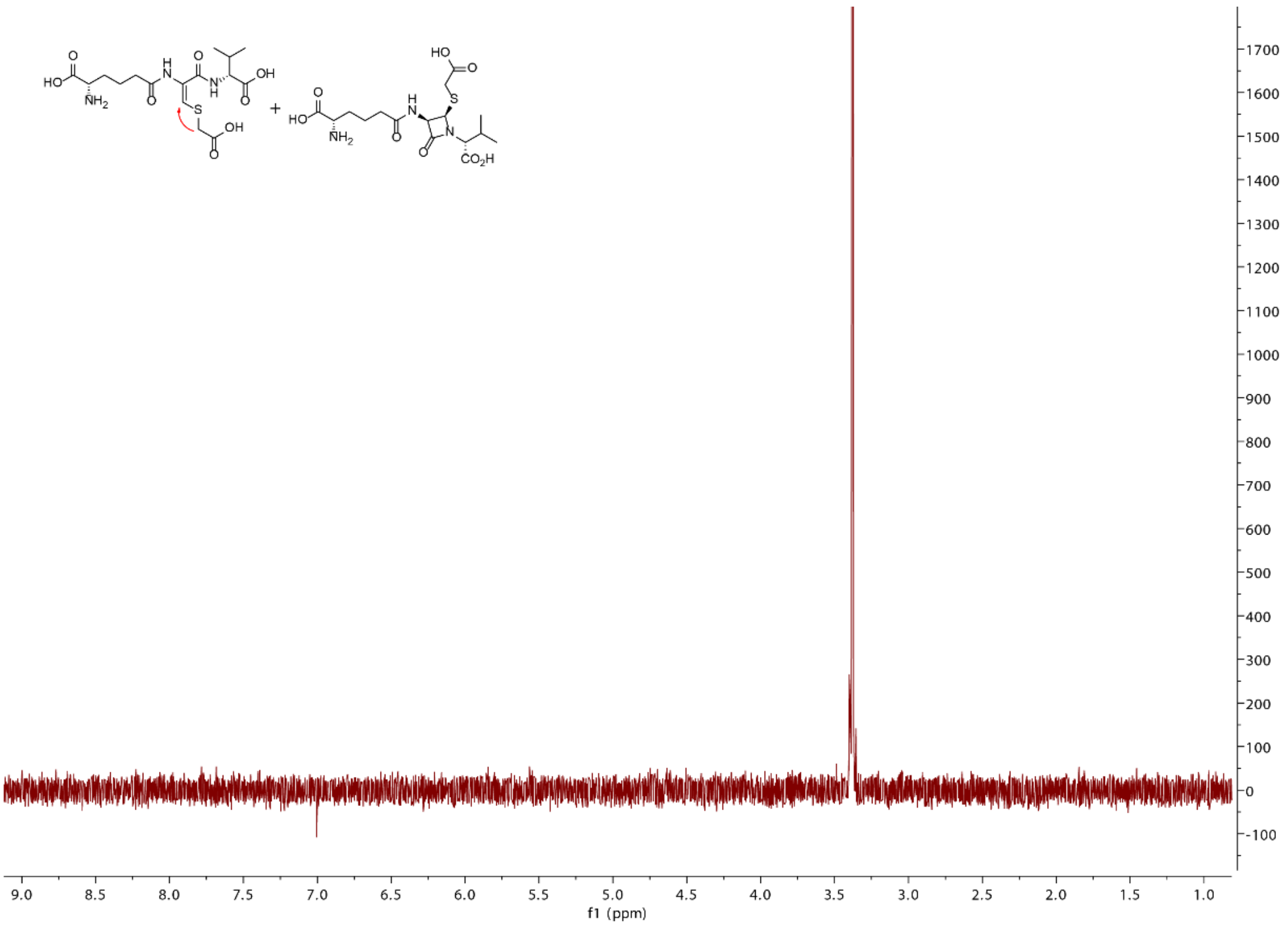

^{1}H NMR spectrum (700 MHz, D_2O) of the hydrated aldehyde (**1**).

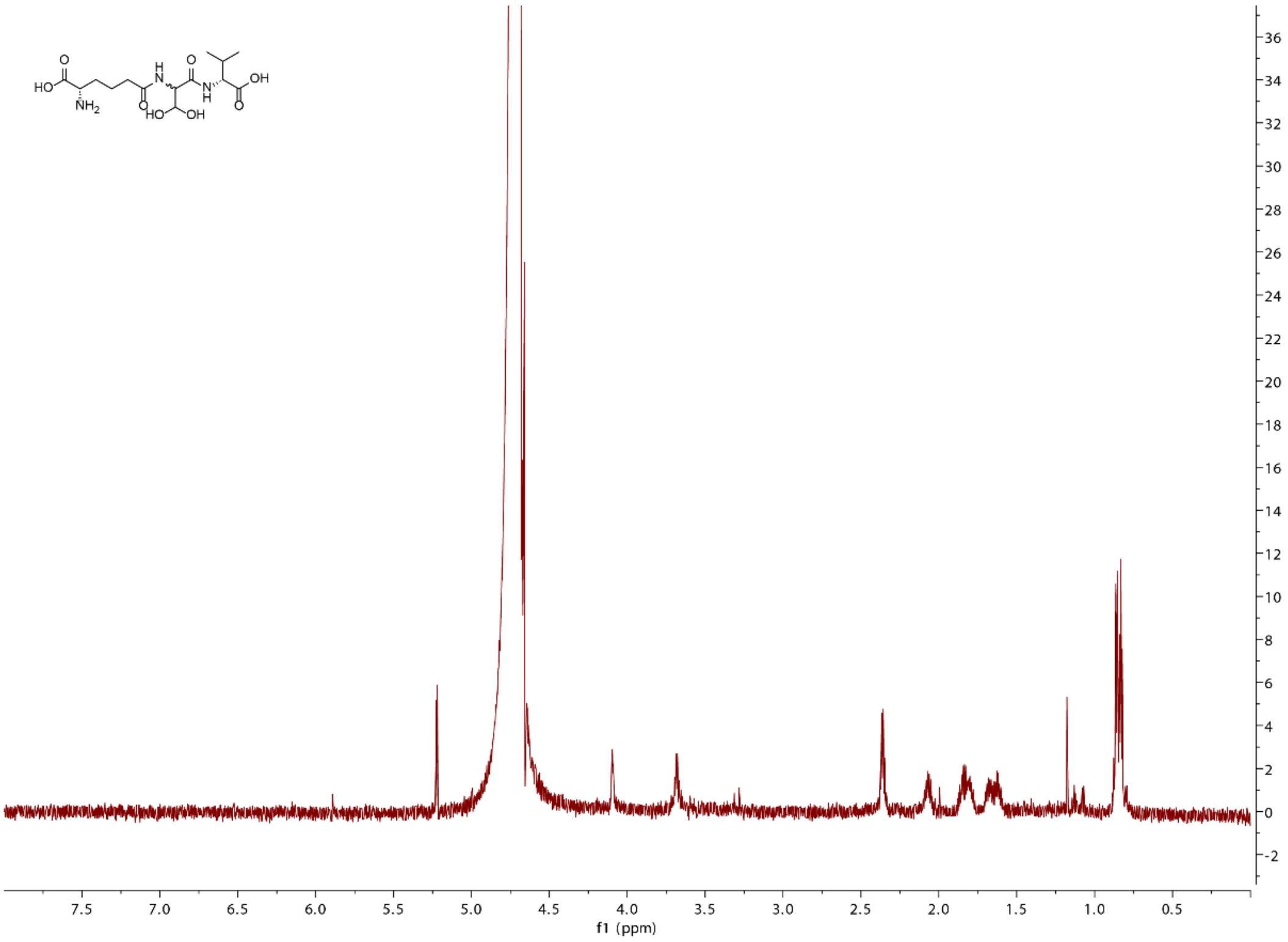

$^{1}H,^{1}H$ TOCSY NMR spectrum (700 MHz, D_2O) of the hydrated aldehyde (**1**). Note, the presence of Tris buffer.

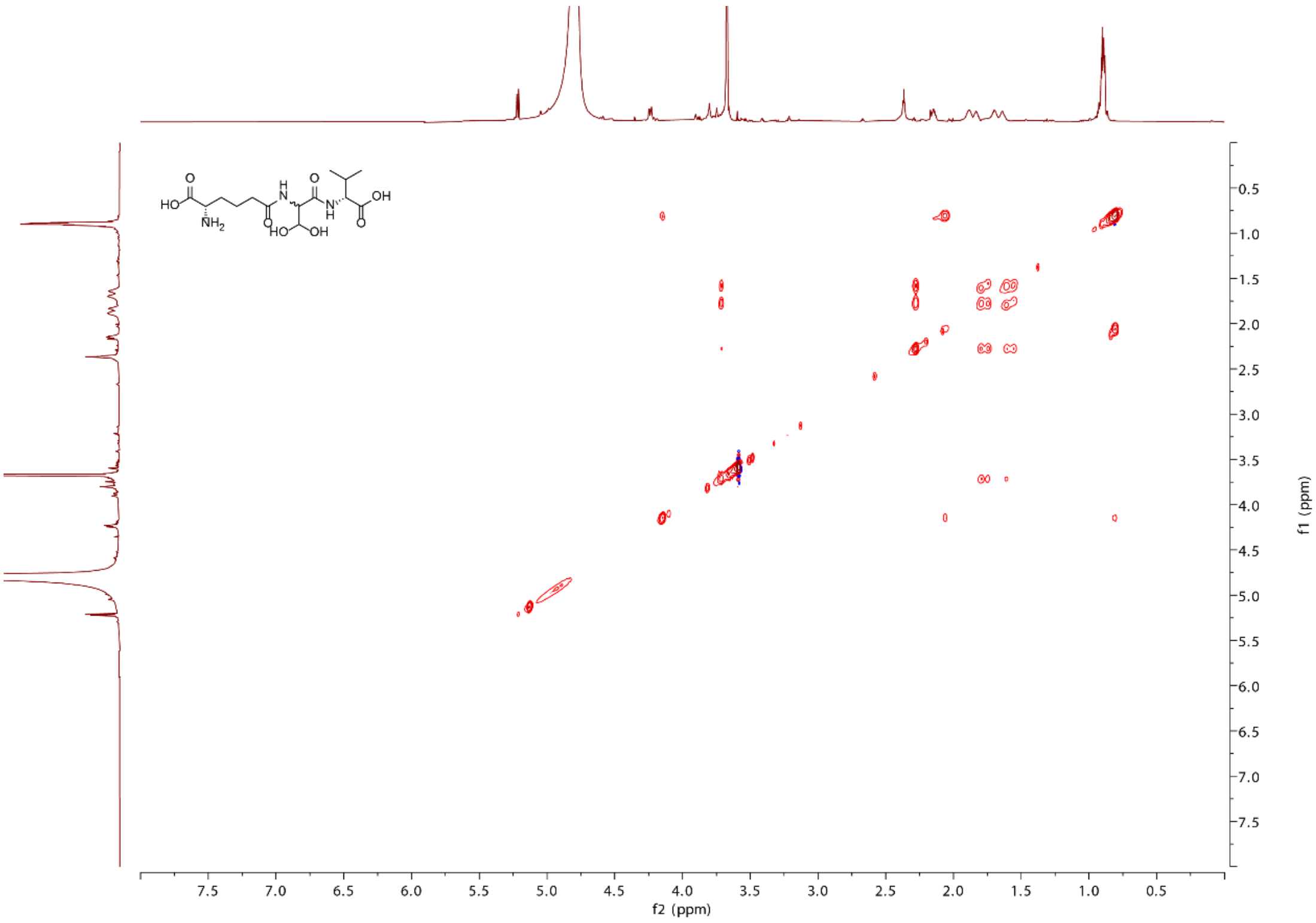

$^{1}H,^{13}C$ HSQG NMR spectrum (700 MHz, D_2O) of the hydrated aldehyde (**1**). Note, the presence of Tris buffer.

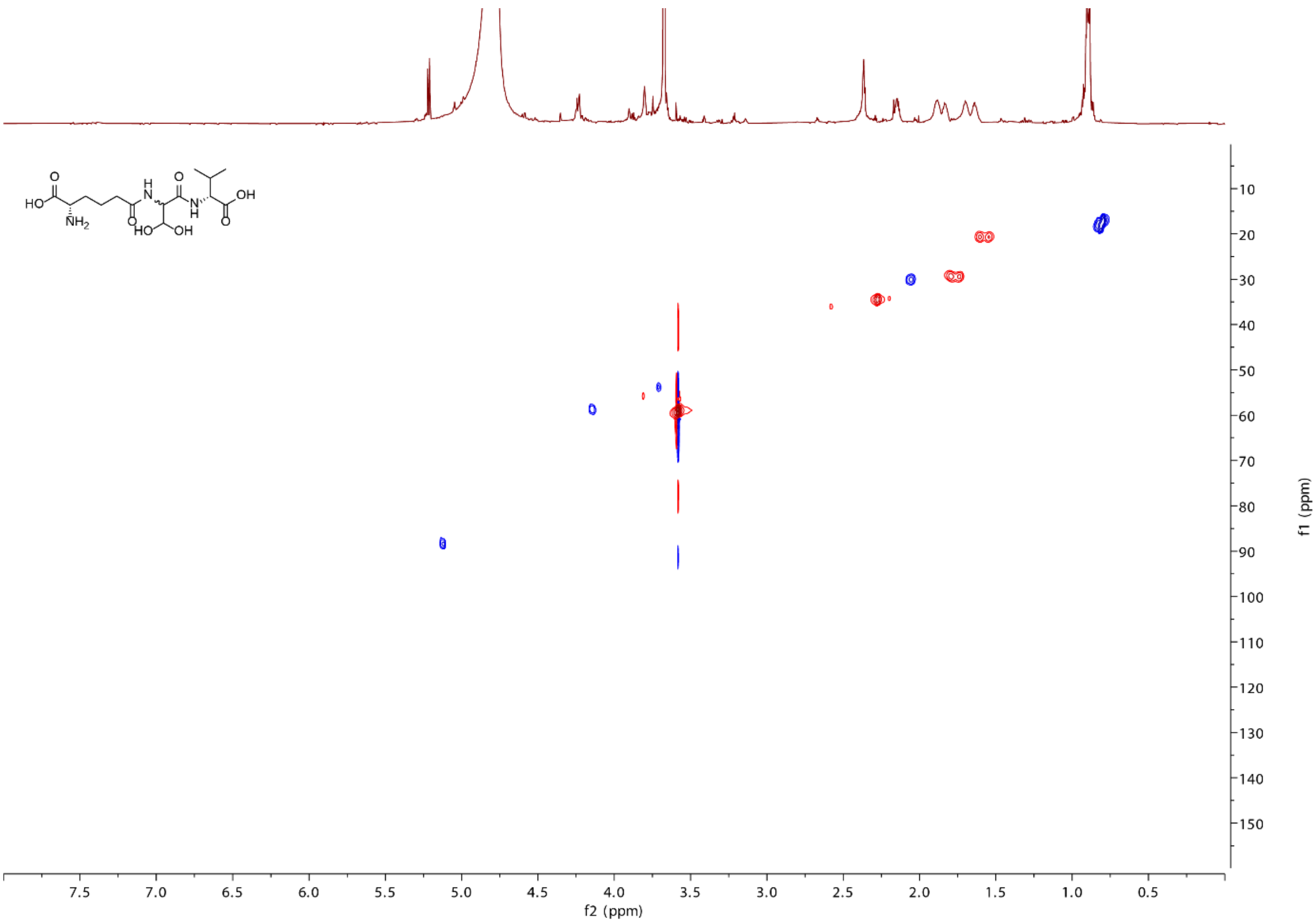

$^{1}H,^{13}C$ HMBC NMR spectrum (700 MHz, D_2O) of the hydrated aldehyde (**1**). Note, the presence of Tris buffer.

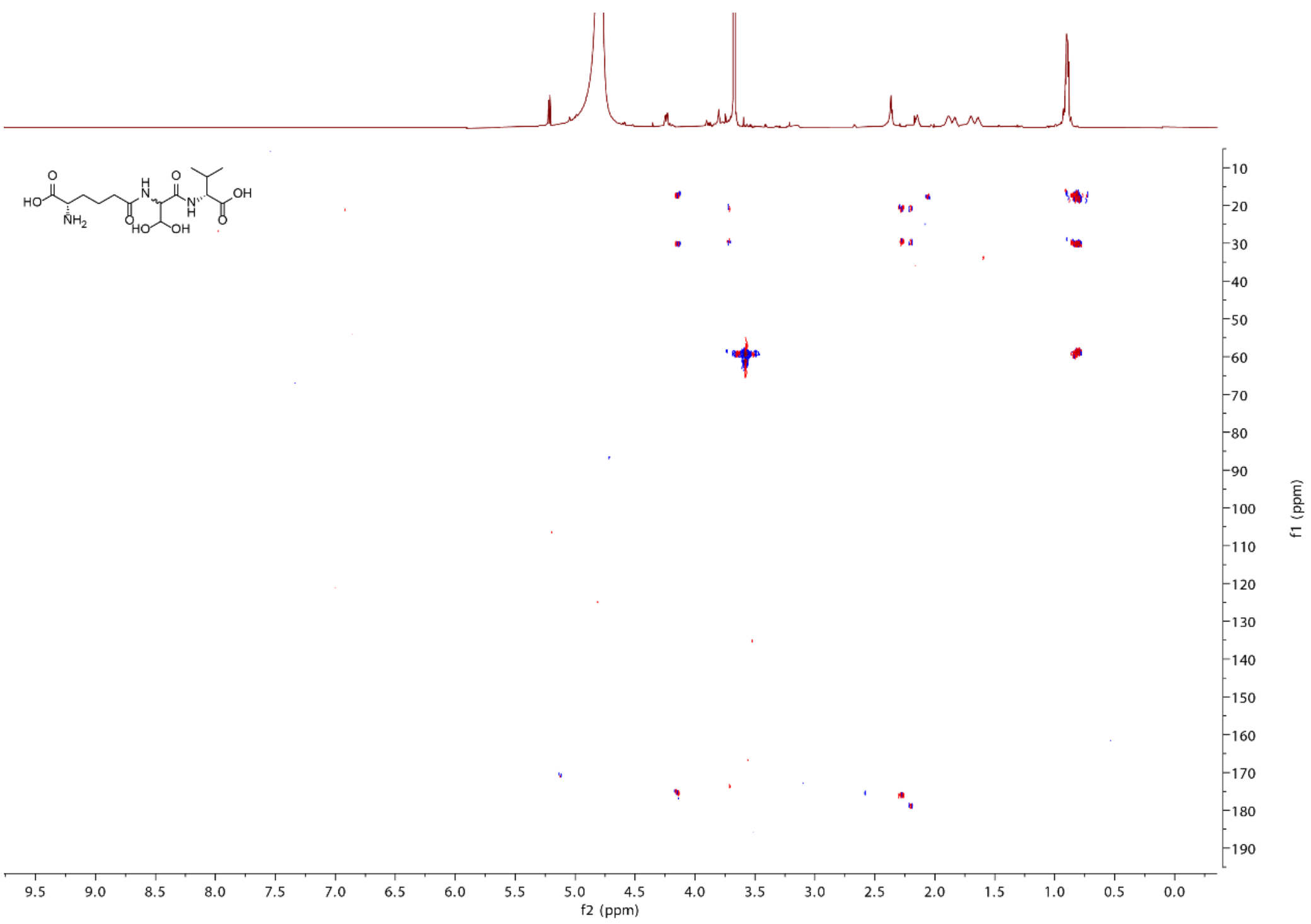

Coordinates

Snapshot 1

RC-I

C	26.2349887	37.0241303	30.8724233
C	26.4206946	37.7539050	32.1669875
N	25.4741129	38.5919357	32.7314297
C	27.4496676	37.7129743	33.0804883
C	25.9301422	39.0030066	33.9322855
N	27.1260804	38.4842198	34.1740937
H	25.7750493	37.6644826	30.1021895
H	27.2201254	36.7179398	30.4892035
H	28.3925641	37.1767521	33.0291827
H	25.3879084	39.6639758	34.6062705
H	24.5663975	38.8464819	32.3334196
C	31.6722468	35.8337167	35.1296746
C	30.6627388	36.6201574	35.9653986
O	29.6381150	37.1445385	35.4214310
O	30.9495557	36.7209356	37.1871190
H	31.8966141	34.8858445	35.6448613
H	32.6132679	36.4108463	35.1657580
C	23.6604645	34.9021266	36.9575104
C	25.0557554	35.3619488	36.6764786
N	26.0521471	34.5851706	36.1160613
C	25.6358860	36.5938859	36.8773317
C	27.1535645	35.3539227	35.9753298
N	26.9369194	36.5777709	36.4322069
H	23.6451519	33.8623905	37.3250916
H	23.2300782	35.5244648	37.7539619
H	25.1923420	37.4923186	37.2994248
H	28.0780534	35.0042012	35.5236793
H	25.9599696	33.6148627	35.7913254
Fe	28.1396066	38.4434463	36.0982035
O	25.9582779	40.4092092	36.6143573
O	26.6711273	39.4011653	36.9300964
C	36.9855774	44.0544091	33.7820743
C	30.9877371	43.4507124	34.5829221
C	29.0846466	43.1777528	36.1785433
C	29.0201915	43.2169402	37.7270796
C	28.4829609	41.8779519	35.5861788
C	35.4896991	44.4811470	33.9623319
C	34.5909211	43.6102670	33.0759981
C	27.5512000	43.5980275	39.7014168
C	26.6955512	44.8560583	39.9964764
C	26.8436377	42.3334712	40.2749755
C	27.6813914	41.0703644	40.0466727
C	26.4708847	42.4771082	41.7565040
C	33.1093590	43.5695545	33.3402351
C	32.4691814	43.6494411	34.5166439
N	30.4774257	43.4124908	35.8348705
N	35.4344305	45.9292570	33.5773035
N	27.8068579	43.4791762	38.2656667
O	30.2869174	43.3282086	33.5690797
O	30.0348996	43.0113005	38.3886374
O	37.7102091	44.8865523	33.1976349
O	37.2795214	42.9186888	34.2088964
O	26.9407619	45.4525392	41.0733724
O	25.7809681	45.1451565	39.1825056
S	29.5472531	40.3871558	35.7928276
H	28.4675257	44.0012661	35.7749113
H	27.4992826	41.7106328	36.0508710
H	28.3274209	42.0417045	34.5118059
H	35.2286852	44.3786771	35.0242762
H	34.9679436	42.5771350	33.1720762
H	34.7688762	43.8855208	32.0221935
H	28.5258602	43.7148638	40.1955958
H	25.9038381	42.2279980	39.7072736
H	32.4767639	43.3842262	32.4642862
H	33.0006729	43.8165839	35.4595944
H	31.0937793	43.3532935	36.6463627
H	27.0288524	43.7518404	37.6701785
H	34.6807010	46.1523692	32.8973757
H	36.3926102	46.0641575	33.1492139
H	35.3234877	46.5964656	34.3647248
H	27.1432405	40.1778531	40.4052618
H	27.8971583	40.9111039	38.9813746
H	28.6422411	41.1312013	40.5850419
H	25.7757720	43.3052398	41.9451382
H	27.3656728	42.6430818	42.3799427
H	25.9767273	41.5579199	42.1087564
O	29.0262127	38.2204137	38.0653035
H	29.8132523	37.6185150	37.8706699
H	29.4152075	39.1049165	38.1764646
H	25.5934403	36.1469539	30.9563545
H	31.4146831	35.6051795	34.0955089
H	22.9835374	34.9103827	36.1032454

TS-I

C	26.2404106	37.0417796	30.8900440
C	26.4370509	37.7748253	32.1812452
N	25.5131266	38.6428148	32.7399710
C	27.4685056	37.7194048	33.0910476
C	25.9875376	39.0579509	33.9340763
N	27.1707423	38.5111946	34.1756850
H	25.7782959	37.6816676	30.1207324
H	27.2233878	36.7335344	30.5026300
H	28.3977493	37.1593307	33.0440249
H	25.4796956	39.7613365	34.5917640
H	24.6082799	38.9094551	32.3433412
C	31.6790188	35.8318037	35.1326886
C	30.6733611	36.6268727	35.9676045
O	29.6437701	37.1403753	35.4311861
O	30.9761204	36.7412463	37.1872357
H	31.8955183	34.8811361	35.6464759
H	32.6252527	36.4001023	35.1705854
C	23.6409745	34.9279717	36.9696975
C	25.0354401	35.3926029	36.6936581
N	26.0353534	34.6074352	36.1499781

C	25.6144101	36.6275970	36.8832393
C	27.1390833	35.3708178	36.0084197
N	26.9196291	36.6001673	36.4501771
H	23.6289635	33.8907248	37.3442095
H	23.2024710	35.5537769	37.7587843
H	25.1761911	37.5375149	37.2886540
H	28.0668145	35.0155193	35.5680178
H	25.9435491	33.6340718	35.8334265
Fe	28.1027930	38.4350078	36.1476003
O	26.4233156	40.8469483	36.5028506
O	26.7811712	39.5882212	36.8447529
C	36.9672162	44.0594767	33.7891589
C	30.9503979	43.4955864	34.6069498
C	29.0492059	43.1012972	36.1797762
C	28.9714453	43.1469281	37.7319580
C	28.6272881	41.7522687	35.5653233
C	35.4746116	44.4978771	33.9829560
C	34.5609776	43.6248064	33.1152364
C	27.5090607	43.5547898	39.7026926
C	26.6730483	44.8280713	39.9905635
C	26.7892224	42.3049036	40.2930741
C	27.6103460	41.0300014	40.0685754
C	26.4361502	42.4691516	41.7773522
C	33.0812101	43.5863419	33.3844738
C	32.4257329	43.7420883	34.5455870
N	30.4278293	43.4671019	35.8566414
N	35.4266335	45.9424338	33.5848447
N	27.7613670	43.4183317	38.2673487
O	30.2641581	43.3139704	33.5926435
O	29.9881701	42.9511954	38.3938925
O	37.6917302	44.8858448	33.1961221
O	37.2594597	42.9234531	34.2159019
O	26.9320945	45.4282295	41.0625805
O	25.7600624	45.1255789	39.1785496
S	29.6549612	40.3535099	35.7947428
H	28.3682540	43.8601262	35.7577676
H	27.4099558	41.3530139	36.0971341
H	28.2832207	41.8854188	34.5300850
H	35.2260388	44.4077089	35.0493692
H	34.9315096	42.5896353	33.2176766
H	34.7317935	43.8859649	32.0561487
H	28.4872679	43.6647251	40.1913264
H	25.8410540	42.2041504	39.7373201
H	32.4593008	43.3209951	32.5213853
H	32.9407494	43.9897930	35.4799923
H	31.0407189	43.4229155	36.6722817
H	26.9750521	43.6517814	37.6673132
H	34.6747152	46.1621912	32.9018656
H	36.3860552	46.0671417	33.1547667
H	35.3181058	46.6164615	34.3666613
H	27.0764062	40.1511837	40.4652335
H	27.7847094	40.8438102	39.0004887
H	28.5880036	41.0964289	40.5756242
H	25.7509663	43.3055459	41.9649809
H	27.3398907	42.6342380	42.3881783
H	25.9375329	41.5589820	42.1459295
O	29.0393337	38.1957311	38.0821291
H	29.8465788	37.6228265	37.8687159
H	29.3954758	39.0786059	38.2726067
H	25.5991227	36.1646112	30.9760238
H	31.4200736	35.6058725	34.0982952
H	22.9709917	34.9267523	36.1099366

IM-I

C	26.2293685	37.0838430	30.9183420
C	26.4483392	37.7703871	32.2313411
N	25.5224089	38.5826221	32.8627221
C	27.5151160	37.7073536	33.1007356
C	26.0375930	38.9571123	34.0600162
N	27.2438012	38.4403912	34.2317157
H	25.7443748	37.7466534	30.1825661
H	27.2077974	36.8030712	30.4994618
H	28.4529398	37.1695711	32.9891545
H	25.5113180	39.5640374	34.7952584
H	24.6004979	38.8402382	32.5049464
C	31.7739060	35.7045156	35.1758644
C	30.7867483	36.4969911	36.0375198
O	29.7351781	36.9855799	35.5296157
O	31.1289827	36.6278676	37.2451963
H	31.9613032	34.7275392	35.6509198
H	32.7382228	36.2389077	35.2360633
C	23.5678700	34.8117304	36.9464842
C	24.9789892	35.2326345	36.6918693
N	25.9621897	34.4436623	36.1280973
C	25.5837213	36.4464224	36.9240717
C	27.0832075	35.1898198	36.0151175
N	26.8889091	36.4069868	36.4939179
H	23.5113727	33.7687605	37.3018356
H	23.1488205	35.4398783	37.7441824
H	25.1672999	37.3643204	37.3316559
H	28.0063064	34.8275062	35.5697080
H	25.8586171	33.4842205	35.7798021
Fe	28.1343951	38.1581223	36.2542497
O	25.7823519	39.5142889	37.2873399
O	27.1567481	39.7742209	36.8955577
C	37.0054156	44.0953562	33.7556458
C	31.0334571	43.5541060	34.6335695
C	29.2322193	43.1394479	36.2560829
C	29.0724997	43.3188581	37.8074999
C	28.9977671	41.6669068	36.0079071
C	35.5147948	44.5402889	33.9434231
C	34.6007713	43.6853729	33.0598456
C	27.5094563	43.6880809	39.6907146
C	26.6728513	44.9637511	39.9538393
C	26.7450646	42.4457134	40.2412261
C	27.5040071	41.1403547	39.9714947
C	26.4200883	42.5800272	41.7354662
C	33.1292491	43.6199071	33.3514453
C	32.4860584	43.8681665	34.5029014

N	30.5516973	43.6097575	35.9061133
N	35.4778961	45.9908171	33.5662974
N	27.8275252	43.5585170	38.2653341
O	30.3148694	43.2492205	33.6760338
O	30.0686616	43.1818205	38.5044776
O	37.7369614	44.9242866	33.1761736
O	37.2866755	42.9537612	34.1745796
O	26.9232021	45.5756436	41.0205119
O	25.7650982	45.2428313	39.1293005
S	30.1571155	40.5174542	35.9425130
H	28.4566182	43.7061664	35.7149122
H	25.8176654	39.7373744	38.2308170
H	27.9447162	41.3365081	36.0120651
H	35.2604628	44.4347663	35.0068622
H	34.9776848	42.6490993	33.1266641
H	34.7534102	43.9737736	32.0045164
H	28.4666980	43.7833278	40.2204727
H	25.7849605	42.4095137	39.6957964
H	32.5106924	43.2436713	32.5281130
H	33.0101332	44.2243663	35.3959943
H	31.1940044	43.6592097	36.6953387
H	27.0748166	43.7900302	37.6208143
H	34.7191155	46.2292533	32.8977641
H	36.4359326	46.1137539	33.1318198
H	35.3831139	46.6534606	34.3596182
H	26.9536999	40.2845293	40.3997549
H	27.6393734	40.9401803	38.8971564
H	28.4988920	41.1579874	40.4487454
H	25.7989036	43.4546338	41.9645658
H	27.3424881	42.6576991	42.3356953
H	25.8644241	41.6944049	42.0807545
O	29.1592025	38.0302395	38.2483633
H	29.9819316	37.5148139	37.9950949
H	29.4579812	38.9372257	38.4026338
H	25.6006040	36.1958368	30.9828966
H	31.5027231	35.5280871	34.1349994
H	22.9088339	34.8532065	36.0792943

TS-II

C	26.2293685	37.0838430	30.9183420
C	26.4483392	37.7703871	32.2313411
N	25.5224089	38.5826221	32.8627221
C	27.5151160	37.7073536	33.1007356
C	26.0375930	38.9571123	34.0600162
N	27.2438012	38.4403912	34.2317157
H	25.7443748	37.7466534	30.1825661
H	27.2077974	36.8030712	30.4994618
H	28.4529398	37.1695711	32.9891545
H	25.5113180	39.5640374	34.7952584
H	24.6004979	38.8402382	32.5049464
C	31.7739060	35.7045156	35.1758644
C	30.7867483	36.4969911	36.0375198
O	29.7351781	36.9855799	35.5296157
O	31.1289827	36.6278676	37.2451963
H	31.9613032	34.7275392	35.6509198
H	32.7382228	36.2389077	35.2360633
C	23.5678700	34.8117304	36.9464842
C	24.9789892	35.2326345	36.6918693
N	25.9621897	34.4436623	36.1280973
C	25.5837213	36.4464224	36.9240717
C	27.0832075	35.1898198	36.0151175
N	26.8889091	36.4069868	36.4939179
H	23.5113727	33.7687605	37.3018356
H	23.1488205	35.4398783	37.7441824
H	25.1672999	37.3643204	37.3316559
H	28.0063064	34.8275062	35.5697080
H	25.8586171	33.4842205	35.7798021
Fe	28.1343951	38.1581223	36.2542497
O	25.7823519	39.5142889	37.2873399
O	27.1567481	39.7742209	36.8955577
C	37.0054156	44.0953562	33.7556458
C	31.0334571	43.5541060	34.6335695
C	29.2322193	43.1394479	36.2560829
C	29.0724997	43.3188581	37.8074999
C	28.9977671	41.6669068	36.0079071
C	35.5147948	44.5402889	33.9434231
C	34.6007713	43.6853729	33.0598456
C	27.5094563	43.6880809	39.6907146
C	26.6728513	44.9637511	39.9538393
C	26.7450646	42.4457134	40.2412261
C	27.5040071	41.1403547	39.9714947
C	26.4200883	42.5800272	41.7354662
C	33.1292491	43.6199071	33.3514453
C	32.4860584	43.8681665	34.5029014
N	30.5516973	43.6097575	35.9061133
N	35.4778961	45.9908171	33.5662974
N	27.8275252	43.5585170	38.2653341
O	30.3148694	43.2492205	33.6760338
O	30.0686616	43.1818205	38.5044776
O	37.7369614	44.9242866	33.1761736
O	37.2866755	42.9537612	34.1745796
O	26.9232021	45.5756436	41.0205119
O	25.7650982	45.2428313	39.1293005
S	30.1571155	40.5174542	35.9425130
H	28.4566182	43.7061664	35.7149122
H	25.8176654	39.7373744	38.2308170
H	27.9447162	41.3365081	36.0120651
H	35.2604628	44.4347663	35.0068622
H	34.9776848	42.6490993	33.1266641
H	34.7534102	43.9737736	32.0045164
H	28.4666980	43.7833278	40.2204727
H	25.7849605	42.4095137	39.6957964
H	32.5106924	43.2436713	32.5281130
H	33.0101332	44.2243663	35.3959943
H	31.1940044	43.6592097	36.6953387
H	27.0748166	43.7900302	37.6208143
H	34.7191155	46.2292533	32.8977641
H	36.4359326	46.1137539	33.1318198
H	35.3831139	46.6534606	34.3596182

H	26.9536999	40.2845293	40.3997549
H	27.6393734	40.9401803	38.8971564
H	28.4988920	41.1579874	40.4487454
H	25.7989036	43.4546338	41.9645658
H	27.3424881	42.6576991	42.3356953
H	25.8644241	41.6944049	42.0807545
O	29.1592025	38.0302395	38.2483633
H	29.9819316	37.5148139	37.9950949
H	29.4579812	38.9372257	38.4026338
H	25.6006040	36.1958368	30.9828966
H	31.5027231	35.5280871	34.1349994
H	22.9088339	34.8532065	36.0792943

IM-II

C	26.3030429	37.0791982	30.9931740
C	26.5314130	37.7196411	32.3286871
N	25.6070789	38.5313247	32.9664088
C	27.5862481	37.6217425	33.2105685
C	26.1007134	38.8675798	34.1771626
N	27.2998636	38.3320719	34.3549293
H	25.8397899	37.7823715	30.2816845
H	27.2743314	36.7846792	30.5687367
H	28.5221320	37.0819932	33.1092158
H	25.5782942	39.4636975	34.9211261
H	24.6922281	38.8044528	32.6032816
C	31.6318501	35.7293617	35.1325838
C	30.5957703	36.4084179	36.0327537
O	29.5937725	37.0124104	35.5077398
O	30.8454026	36.3682093	37.2557000
H	31.9155241	34.7706777	35.5942175
H	32.5399929	36.3563960	35.1868529
C	23.5617135	34.8052287	36.9587100
C	24.9742667	35.2242370	36.7102352
N	25.9319214	34.4559385	36.0723979
C	25.6004401	36.4169620	36.9888486
C	27.0548818	35.1952652	35.9570258
N	26.8848221	36.3855366	36.5055925
H	23.5000377	33.7601883	37.3075931
H	23.1379531	35.4287532	37.7569703
H	25.2301615	37.2758462	37.5400287
H	27.9593912	34.8529332	35.4611847
H	25.8109483	33.5133647	35.6882812
Fe	28.1305651	38.1708506	36.3447670
O	26.6156188	38.2821013	39.5273477
O	26.9842590	39.2417705	36.8241024
C	36.9802196	44.0496145	33.7337116
C	31.0076413	43.4277851	34.5681831
C	29.2012486	43.0648068	36.2286461
C	29.0979918	43.3412685	37.7744667
C	28.8578167	41.6030314	36.0538437
C	35.4832861	44.4771400	33.9129293
C	34.5843405	43.6227916	33.0112996
C	27.5871001	43.7399674	39.6927557
C	26.6770980	44.9588669	39.9600507
C	26.9492441	42.4324189	40.2521828
C	27.9214537	41.2529882	40.1465620
C	26.4363269	42.5767908	41.6912793
C	33.1091604	43.5429946	33.2895950
C	32.4646085	43.7299505	34.4510291
N	30.5390077	43.4361747	35.8430215
N	35.4377075	45.9302599	33.5458668
N	27.8729761	43.6054258	38.2604313
O	30.2810546	43.1757158	33.6006509
O	30.1226196	43.2450559	38.4349963
O	37.7028793	44.8832816	33.1494734
O	37.2751467	42.9153713	34.1623072
O	26.8920396	45.5698621	41.0344733
O	25.7614551	45.2054107	39.1323525
S	29.9147073	40.3848978	35.8422133
H	28.4492616	43.6561561	35.6778679
H	26.4159098	38.9139637	38.8161884
H	27.7933413	41.3511544	36.1816264
H	35.2195761	44.3603982	34.9727378
H	34.9700107	42.5892132	33.0717535
H	34.7446703	43.9257185	31.9613205
H	28.5463522	43.9138295	40.1985571
H	26.0728111	42.2237021	39.6114916
H	32.4924889	43.2153369	32.4444232
H	32.9892579	44.0349780	35.3626189
H	31.1793707	43.5015741	36.6339341
H	27.0942011	43.8134288	37.6387999
H	34.6645203	46.1745031	32.8969921
H	36.3887513	46.0561533	33.0966291
H	35.3608097	46.5885418	34.3449806
H	27.4340197	40.3140501	40.4454593
H	28.2838340	41.0869650	39.1220983
H	28.8040117	41.4212165	40.7860810
H	25.6403615	43.3269013	41.7933929
H	27.2504519	42.8496365	42.3829429
H	26.0154380	41.6171537	42.0281720
O	28.9261926	38.1224707	37.9737397
H	29.6775465	37.4852087	37.9004548
H	27.5210194	38.0400622	39.2405902
H	25.6479687	36.2084547	31.0205752
H	31.3773295	35.5393541	34.0899029
H	22.9097109	34.8539617	36.0865948

IM-III

C	26.3187157	37.0681628	30.9990942
C	26.5656948	37.6909637	32.3398845
N	25.6612125	38.5139703	32.9913169
C	27.6213726	37.5602368	33.2163369
C	26.1673008	38.8255864	34.2045234
N	27.3541271	38.2607638	34.3705930
H	25.8520983	37.7819564	30.3006769
H	27.2837302	36.7723342	30.5612271
H	28.5460259	37.0036239	33.1057357
H	25.6630978	39.4299680	34.9546235

H	24.7508589	38.8076314	32.6346650
C	31.6941721	35.6395817	35.0515925
C	30.6613909	36.2852103	35.9732262
O	29.6303512	36.8627776	35.4759356
O	30.9381269	36.2433524	37.1910202
H	31.9977841	34.6769579	35.4918485
H	32.5935350	36.2787257	35.1074189
C	23.5448394	34.8296599	36.9479864
C	24.9658241	35.2190000	36.7008302
N	25.9238762	34.4006962	36.1306924
C	25.6100400	36.4125778	36.9338604
C	27.0671166	35.1078505	36.0139474
N	26.9083852	36.3263901	36.4971728
H	23.4664924	33.7936416	37.3186798
H	23.1261471	35.4763970	37.7305774
H	25.2275594	37.3322862	37.3707896
H	27.9774069	34.7233922	35.5619178
H	25.7918184	33.4483792	35.7746194
Fe	28.2128374	38.0493489	36.3431577
O	28.5578893	40.3775310	39.0078822
O	27.0870266	39.1124302	36.8795622
C	36.9533094	44.0398503	33.7576366
C	31.0007855	43.3885272	34.7272293
C	29.2606041	42.9276403	36.4484124
C	29.1709542	43.2689354	37.9860811
C	28.9547128	41.4612446	36.2661068
C	35.4617199	44.4812756	33.9561253
C	34.5391156	43.6211148	33.0845462
C	27.6764616	43.8040355	39.8865678
C	26.7646246	45.0355439	40.0642897
C	27.0753675	42.5525704	40.6090402
C	25.8136080	42.0255232	39.9245274
C	26.8061019	42.7857588	42.0962030
C	33.0693014	43.5477718	33.3962832
C	32.4557697	43.6977342	34.5801517
N	30.5744403	43.3389225	36.0169025
N	35.4182268	45.9301410	33.5725354
N	27.9447950	43.5443578	38.4689836
O	30.2505762	43.1785209	33.7675079
O	30.2100882	43.2650676	38.6267817
O	37.6798032	44.8706224	33.1735832
O	37.2414670	42.8998316	34.1759592
O	26.9590277	45.6996139	41.1131489
O	25.8599524	45.2443925	39.2126477
S	29.9913359	40.2984477	35.7854612
H	28.4790101	43.4840101	35.8978407
H	29.0306212	39.5514080	38.7596986
H	27.9213158	41.1677383	36.4946573
H	35.2176905	44.3809410	35.0224173
H	34.9238703	42.5877212	33.1471555
H	34.6772911	43.9129846	32.0288704
H	28.6436409	44.0318264	40.3528649
H	27.8433789	41.7708452	40.5125221
H	32.4292942	43.2525381	32.5565724
H	33.0074046	43.9664980	35.4870848
H	31.2352349	43.4184689	36.7899749
H	27.1637836	43.7065717	37.8389872
H	34.6610045	46.1608193	32.8993496
H	36.3760395	46.0535580	33.1379305
H	35.3208236	46.5988297	34.3605834
H	25.4383939	41.1327412	40.4499719
H	25.0059617	42.7735179	39.9184320
H	26.0335882	41.7263031	38.8889340
H	26.0575446	43.5699657	42.2788540
H	27.7210491	43.0596197	42.6394456
H	26.4119826	41.8621243	42.5451906
O	29.1040719	38.0137252	37.9227807
H	29.8522144	37.3746605	37.8257105
H	27.6841010	40.1557221	38.6470684
H	25.6594896	36.2005026	31.0246632
H	31.4239548	35.4668926	34.0098496
H	22.8995507	34.8680847	36.0703799

TS-III

C	26.2615383	36.9714458	30.8337822
C	26.4814514	37.7115206	32.1169932
N	25.5628339	38.5844973	32.6773784
C	27.5132367	37.6478570	33.0264019
C	26.0335882	38.9905183	33.8719373
N	27.2149803	38.4373441	34.1132372
H	25.8059040	37.6143727	30.0635582
H	27.2334774	36.6390679	30.4396687
H	28.4397868	37.0863439	32.9834907
H	25.5070982	39.6554395	34.5512438
H	24.6591061	38.8580815	32.2815478
C	31.5576484	35.8825738	35.1542467
C	30.5742294	36.7337695	35.9628196
O	29.5000160	37.1703322	35.4071980
O	30.9129278	36.9668487	37.1415508
H	31.7258084	34.9370757	35.6960942
H	32.5240181	36.4134051	35.2032531
C	23.5812212	34.8484012	36.9394761
C	24.9739687	35.3083586	36.6525049
N	25.9780241	34.5200532	36.1224931
C	25.5321886	36.5581796	36.7867954
C	27.0655614	35.2932464	35.9311212
N	26.8264828	36.5332719	36.3292668
H	23.5631845	33.8071229	37.3010198
H	23.1517990	35.4700938	37.7364710
H	25.0864431	37.4757815	37.1633388
H	27.9933768	34.9369001	35.4921747
H	25.8974454	33.5371776	35.8328534
Fe	27.9922459	38.3232180	36.0853976
O	28.0189177	40.4274906	38.8492001
O	26.7714975	39.2852843	36.5582887
C	36.8991647	44.0740955	33.8052795
C	30.8813744	43.4791157	34.7714566
C	29.0633580	43.0904518	36.4471944
C	28.8724302	43.5731079	38.0073416

C	28.6818330	41.6914028	36.2120597
C	35.4146475	44.5351825	34.0338589
C	34.4636306	43.6645268	33.2060346
C	27.6238026	43.4887432	40.0392420
C	26.7815945	44.8068017	40.0868128
C	26.8720713	42.3960907	40.8685003
C	25.5216896	42.0229382	40.2499626
C	26.6849247	42.7376412	42.3449478
C	32.9936413	43.5985047	33.5195162
C	32.3277825	43.8641749	34.6555230
N	30.3947278	43.4811085	36.0410087
N	35.3798067	45.9777688	33.6274135
N	27.9452778	42.9401886	38.7055549
O	30.2120858	43.1213682	33.7953275
O	29.6058889	44.5143779	38.3060441
O	37.6232942	44.8925597	33.2010687
O	37.1869270	42.9340016	34.2244608
O	27.0029101	45.5123418	41.1127931
O	25.8933868	45.0440030	39.2338539
S	29.6345661	40.4407291	35.7166155
H	28.2881326	43.7027740	35.9422420
H	28.5924831	39.3271622	38.3476280
H	27.6273244	41.4720639	36.4199937
H	35.1987085	44.4584397	35.1084069
H	34.8411407	42.6300094	33.2924108
H	34.5924573	43.9196506	32.1390651
H	28.5592524	43.7383252	40.5709403
H	27.5162931	41.5045090	40.8208090
H	32.3867177	43.1784292	32.7083697
H	32.8263017	44.2600626	35.5465512
H	30.8797628	43.9901163	36.7827074
H	27.9950256	41.5932350	38.7023553
H	34.6291005	46.2002110	32.9440744
H	36.3377661	46.0875318	33.1883278
H	35.2897775	46.6548239	34.4092279
H	25.0714382	41.1644453	40.7752832
H	24.8106515	42.8586286	40.3128959
H	25.6447332	41.7686622	39.1898742
H	26.0638262	43.6289289	42.4934710
H	27.6451548	42.9097726	42.8522228
H	26.1725479	41.9059039	42.8512410
O	28.7858027	38.3568355	37.8615581
H	29.7059745	37.9931623	37.8211024
H	27.0913558	40.1784453	38.7280917
H	25.6017078	36.1100820	30.9374313
H	31.3179420	35.6361463	34.1198959
H	22.9152932	34.8667075	36.0767642

IM-IV

C	26.3017263	37.2101881	31.1237275
C	26.4740636	37.9157866	32.4355510
N	25.5222913	38.7474350	33.0022475
C	27.5164128	37.9041430	33.3376256
C	25.9935931	39.1812360	34.1916861
N	27.2010214	38.6885370	34.4245479
H	25.8459696	37.8718658	30.3684496
H	27.2933774	36.9235713	30.7431121
H	28.4671971	37.3831743	33.2793000
H	25.4411793	39.8216061	34.8748221
H	24.6080903	38.9787954	32.6067421
C	31.5817966	36.1288666	35.2955191
C	30.5595185	36.8438806	36.1906174
O	29.6004258	37.5181652	35.6865091
O	30.7957619	36.7446525	37.4196473
H	31.8980050	35.2111452	35.8145057
H	32.4764256	36.7778581	35.2785371
C	23.6613687	34.9531080	37.0135126
C	25.0436790	35.4619119	36.7517409
N	26.0776480	34.7089785	36.2266753
C	25.5777930	36.7153850	36.9497279
C	27.1574533	35.5119936	36.0998352
N	26.8868214	36.7325659	36.5353428
H	23.6787360	33.9092278	37.3685913
H	23.2027094	35.5507576	37.8124076
H	25.1083406	37.6095763	37.3533085
H	28.1033466	35.1839505	35.6784437
H	26.0141133	33.7368401	35.9008788
Fe	28.0156773	38.6161585	36.5014092
O	30.1659893	39.8396946	39.7553967
O	26.7206577	39.4674260	36.9886010
C	36.9130533	44.0297951	33.7628985
C	30.8780453	43.3441959	34.3888469
C	28.9008179	43.3865610	35.9253508
C	28.6394875	44.1997930	37.2015924
C	28.2763643	42.1707663	36.7313971
C	35.4026265	44.4215777	33.9271706
C	34.5373865	43.5442848	33.0130922
C	27.7922715	43.2131890	39.3180213
C	26.7776125	44.3573710	39.7176982
C	27.3460259	41.8658111	39.9357995
C	25.9554642	41.4004894	39.5013200
C	27.4463300	41.8998235	41.4661390
C	33.0520835	43.4452050	33.2343825
C	32.3471242	43.6179186	34.3628811
N	30.3137422	43.4069828	35.6258920
N	35.3264210	45.8745196	33.5649857
N	28.1328721	43.1366885	37.8852608
O	30.2191925	43.0734716	33.3813791
O	28.8981586	45.3335515	37.5349427
O	37.6212121	44.8712700	33.1728649
O	37.2346455	42.9086553	34.2082208
O	26.9610896	44.7711996	40.8959211
O	25.8704751	44.7116507	38.9395000
S	29.3657583	40.6680257	36.7214838
H	28.3285978	43.6681426	35.0294719
H	29.0626428	38.6269283	39.0138799
H	27.2963856	41.8395343	36.3608830
H	35.1296286	44.2959488	34.9834407

H	34.9374308	42.5181427	33.0992102
H	34.7278659	43.8377017	31.9657691
H	28.7275457	43.5076782	39.8263188
H	28.0648457	41.1142684	39.5722212
H	32.4750330	43.1212423	32.3601378
H	32.8225800	43.9300527	35.2991057
H	30.9300220	43.6327444	36.3992680
H	30.1187356	40.2907635	38.8829145
H	34.5660475	46.0999289	32.8930854
H	36.2785592	46.0277532	33.1303664
H	35.2227722	46.5265273	34.3663419
H	25.6603716	40.5088960	40.0785014
H	25.1980294	42.1807783	39.6711846
H	25.9602927	41.1036862	38.4451627
H	26.6991406	42.5762116	41.9027449
H	28.4343841	42.2561254	41.8027620
H	27.2747726	40.8901001	41.8672620
O	28.6969877	37.9276765	38.4245736
H	29.5112928	37.4021196	38.1775745
H	29.6970787	40.4405570	40.3506104
H	25.6680840	36.3238575	31.1550746
H	31.3236699	35.8525799	34.2732161
H	22.9886857	34.9491944	36.1558708

TS-IV

C	26.2964814	37.3292805	31.3310360
C	26.4240529	37.9741381	32.6799436
N	25.4541179	38.7793717	33.2544061
C	27.4454153	37.9336105	33.6043411
C	25.8954731	39.1727641	34.4711468
N	27.0998997	38.6769469	34.7124097
H	25.8438776	38.0231050	30.6031678
H	27.3034567	37.0854778	30.9616104
H	28.4011805	37.4198639	33.5526525
H	25.3303531	39.7925843	35.1634159
H	24.5528258	39.0210321	32.8372855
C	31.6377170	36.0538002	35.3000847
C	30.6210028	36.7437917	36.2188103
O	29.6275116	37.3842980	35.7594014
O	30.9090512	36.6482952	37.4439618
H	31.9639192	35.1241873	35.7915445
H	32.5296707	36.7063760	35.2889505
C	23.6284468	34.9475420	37.0701840
C	25.0195665	35.4560863	36.8641036
N	26.0577062	34.7226290	36.3202135
C	25.5682880	36.6838963	37.1634095
C	27.1528159	35.5130663	36.2788344
N	26.8912807	36.7065044	36.7902788
H	23.6325046	33.9017482	37.4213169
H	23.1480103	35.5418913	37.8576320
H	25.1003532	37.5590464	37.6088118
H	28.1036000	35.2035485	35.8538127
H	25.9931688	33.7701099	35.9422032
Fe	28.0380056	38.6136479	36.7135933
O	30.6843244	40.1053651	39.3099470
O	26.8161216	39.6169891	37.4676486
C	36.9184866	44.0499076	33.7621358
C	30.8807695	43.4727226	34.4691774
C	28.9267576	43.4637993	36.0240631
C	28.6378940	43.9914810	37.4380209
C	28.3177044	42.1003614	36.5561209
C	35.4123718	44.4494210	33.9428947
C	34.5284294	43.5670843	33.0534119
C	27.5962198	42.5755995	39.2141437
C	26.7873920	43.8116428	39.8239656
C	26.7716773	41.2752179	39.4532970
C	25.2981770	41.4921163	39.7701940
C	27.4187453	40.3980220	40.5126026
C	33.0453615	43.4925102	33.2940155
C	32.3513890	43.7394201	34.4155210
N	30.3270345	43.5880670	35.7068434
N	35.3369310	45.8987519	33.5663566
N	28.0408995	42.8345677	37.8390209
O	30.2116060	43.1665500	33.4792093
O	28.9354775	45.0082068	38.0244301
O	37.6238313	44.8864070	33.1611187
O	37.2402264	42.9293181	34.2081497
O	26.9570127	43.9729295	41.0583540
O	26.0084498	44.4316191	39.0736230
S	29.4821521	40.6808528	36.4537965
H	28.3150847	43.9027744	35.2211177
H	29.5406983	38.7844684	38.9179665
H	27.3879818	41.8054249	36.0524683
H	35.1544088	44.3362229	35.0045654
H	34.9171234	42.5377271	33.1509993
H	34.7101318	43.8402864	31.9986771
H	28.5065293	42.5038138	39.8354272
H	26.7911928	40.5748909	38.4254020
H	32.4590470	43.1218794	32.4446609
H	32.8382001	44.0998489	35.3283763
H	30.9421578	43.8203053	36.4791658
H	30.4292553	40.4909158	38.4350782
H	34.5734649	46.1190451	32.8961963
H	36.2878707	46.0444692	33.1252779
H	35.2377977	46.5570391	34.3628925
H	24.7316999	40.5543243	39.6855460
H	25.1891290	41.8578583	40.8051544
H	24.8617863	42.2591683	39.1275662
H	27.4533799	40.9665715	41.4600075
H	28.4413718	40.1154081	40.2362158
H	26.8442720	39.4736819	40.6784253
O	29.0324159	38.0467991	38.5060228
H	29.7619417	37.4174964	38.2094148
H	30.4096570	40.7717263	39.9539424
H	25.6850735	36.4277272	31.2929694
H	31.3596570	35.8061594	34.2756708
H	22.9769051	34.9463268	36.1963653

IM-V

C	26.2768007	37.2572199	31.1904955
C	26.4292332	37.9161552	32.5291001
N	25.4603059	38.7107758	33.1207971
C	27.4646842	37.8827827	33.4375008
C	25.9134071	39.1038671	34.3308454
N	27.1274797	38.6192209	34.5522207
H	25.8170962	37.9406950	30.4572689
H	27.2753110	36.9977614	30.8083702
H	28.4242831	37.3787808	33.3685529
H	25.3548601	39.7026281	35.0462435
H	24.5489873	38.9465913	32.7221664
C	31.5803571	36.0119707	35.3043222
C	30.5152226	36.6590179	36.1988758
O	29.6260470	37.4225099	35.7055513
O	30.6562004	36.4210077	37.4295498
H	31.9209881	35.0835320	35.7863372
H	32.4494474	36.6952782	35.3127270
C	23.6114818	34.9174485	37.0262758
C	25.0082044	35.4018732	36.8031009
N	26.0325224	34.6502888	36.2549238
C	25.5707571	36.6282053	37.0752079
C	27.1345814	35.4307377	36.1891094
N	26.8894269	36.6331202	36.6849173
H	23.5988482	33.8723833	37.3796268
H	23.1488960	35.5216156	37.8174131
H	25.1233088	37.5230787	37.5016144
H	28.0799748	35.0982113	35.7686681
H	25.9551899	33.6974748	35.8842174
Fe	27.9894678	38.5968846	36.5731548
O	30.8867409	39.9431658	39.0499310
O	26.4815561	39.4794439	37.2268287
C	36.8945723	44.0366311	33.7587722
C	30.8331375	43.4886613	34.4063340
C	28.8463529	43.5774719	35.9296940
C	28.5837470	44.2531852	37.2841634
C	28.1995684	42.2894911	36.5999784
C	35.3878252	44.4394269	33.9368770
C	34.5051430	43.5571921	33.0458525
C	27.5918074	43.0593042	39.2417616
C	26.7120727	44.2733380	39.7587075
C	26.9188514	41.7607904	39.6505993
C	25.4778591	41.7562204	40.0588817
C	27.7824938	40.7221174	40.2944690
C	33.0196003	43.4838497	33.2721023
C	32.3058874	43.7518927	34.3763545
N	30.2570030	43.6282479	35.6311266
N	35.3175516	45.8889046	33.5604936
N	28.0057615	43.1470273	37.8246897
O	30.1826595	43.1652739	33.4097649
O	28.8852622	45.3280931	37.7511109
O	37.6028254	44.8728527	33.1607441
O	37.2143152	42.9149981	34.2036020
O	26.8826810	44.4903747	40.9859719
O	25.8883713	44.8280312	39.0016262
S	29.3035277	40.8100087	36.5391502
H	28.2631209	43.9521086	35.0750138
H	29.5640078	38.7748950	38.7491866
H	27.2365274	41.9966455	36.1584440
H	35.1272048	44.3272243	34.9979711
H	34.8912221	42.5273650	33.1492089
H	34.6945827	43.8261636	31.9912894
H	28.5202026	43.1423792	39.8365246
H	26.6124733	40.1979872	37.8683070
H	32.4466377	43.0939846	32.4221245
H	32.7746979	44.1361150	35.2891232
H	30.8638588	43.8892867	36.4007054
H	30.5249307	40.4866133	38.3072778
H	34.5587920	46.1106716	32.8855128
H	36.2710500	46.0323123	33.1239920
H	35.2159634	46.5480602	34.3561022
H	25.0205315	40.7618702	39.9278838
H	25.3825858	42.0125739	41.1334001
H	24.8919065	42.5078043	39.5188681
H	27.9245494	40.9893734	41.3640132
H	28.7753341	40.6570526	39.8343661
H	27.3317830	39.7168148	40.2640078
O	28.9404623	38.0937563	38.4010615
H	29.5617526	37.3378242	38.1564612
H	30.8838494	40.5282145	39.8176599
H	25.6555318	36.3616488	31.1838203
H	31.3264969	35.7736766	34.2714437
H	22.9576909	34.9281195	36.1542032

TS-V

C	26.3199403	37.2781497	31.1918020
C	26.4849118	37.9646852	32.5155903
N	25.5188299	38.7759535	33.0883337
C	27.5312795	37.9749380	33.4143195
C	25.9836393	39.2192799	34.2761253
N	27.2058703	38.7559040	34.5066847
H	25.8732687	37.9595123	30.4483686
H	27.3140669	37.0000064	30.8122757
H	28.4874945	37.4625783	33.3553630
H	25.4187184	39.8183267	34.9856922
H	24.5998665	38.9879474	32.6946810
C	31.5394152	36.0630136	35.3280416
C	30.4840292	36.7254273	36.2293327
O	29.6010652	37.4981096	35.7473481
O	30.6345934	36.4842915	37.4605832
H	31.8719873	35.1339868	35.8144422
H	32.4179331	36.7341672	35.3336593
C	23.5951151	34.8880048	36.9979360
C	24.9844262	35.3846543	36.7584463
N	26.0229460	34.6406999	36.2277076
C	25.5149877	36.6329054	36.9822070
C	27.1038863	35.4487314	36.1270593
N	26.8296825	36.6600904	36.5819865

H	23.5909295	33.8381440	37.3364562
H	23.1405984	35.4809388	37.8026001
H	25.0481331	37.5334793	37.3730061
H	28.0566038	35.1259828	35.7160472
H	25.9619648	33.6801897	35.8749701
Fe	27.9212341	38.6297163	36.6567551
O	30.1858911	39.6467166	40.0940189
O	26.2620701	39.4278564	37.0834989
C	36.9140026	43.9886344	33.7584052
C	30.8757508	43.1490253	34.3253981
C	28.8921587	43.2902127	35.8068066
C	28.6871608	44.3679558	36.8990128
C	28.1045181	42.3425472	36.7979121
C	35.3986695	44.3599293	33.9166465
C	34.5488653	43.4490049	33.0153783
C	27.8209992	43.6136546	39.1426807
C	26.7162177	44.6131372	39.7075513
C	27.5923226	42.2620771	39.7656003
C	26.2553128	41.6121762	39.5953169
C	28.2404935	42.0383882	41.0937039
C	33.0489939	43.4118210	33.1963227
C	32.3657334	43.2402208	34.3393314
N	30.2891394	43.0497920	35.5521384
N	35.3009053	45.8063604	33.5341954
N	27.9584833	43.5228994	37.6926177
O	30.2066944	43.1655365	33.2880594
O	29.1160962	45.4830939	37.0724077
O	37.6118488	44.8325797	33.1598286
O	37.2502776	42.8754705	34.2136874
O	26.8587636	44.8261557	40.9409497
O	25.8217139	45.0577255	38.9655733
S	28.9713884	40.9033001	37.5034771
H	28.3891603	43.4766574	34.8476222
H	29.1772735	38.5515225	39.0919008
H	27.1426302	41.9883565	36.3962880
H	35.1195067	44.2469850	34.9726280
H	34.9321808	42.4280780	33.1790830
H	34.7846833	43.6943472	31.9667176
H	28.7669186	44.0303342	39.5312232
H	26.3620688	40.0757808	37.7911143
H	32.4477323	43.4748767	32.2824857
H	32.8736986	43.1612630	35.3074048
H	30.8935232	42.9788166	36.3632033
H	30.1265863	40.3309744	39.3961309
H	34.5312686	46.0154400	32.8678726
H	36.2476200	45.9666316	33.0918268
H	35.1965711	46.4663067	34.3293881
H	26.3101981	40.5227245	39.7577640
H	25.5232866	42.0089228	40.3296619
H	25.8412200	41.8097246	38.6018020
H	27.7726962	42.7068533	41.8430228
H	29.3155471	42.2774643	41.0840449
H	28.0840903	41.0057661	41.4465579
O	28.7397346	37.9309528	38.4631867
H	29.4720649	37.2887368	38.1931744
H	29.6068280	40.0001715	40.7831955
H	25.6844997	36.3925578	31.1887279
H	31.2886256	35.8190488	34.2957369
H	22.9358231	34.9116897	36.1302724

PD

C	26.2332828	37.0814215	30.9009848
C	26.4930011	37.7827565	32.1993915
N	25.6092476	38.6443155	32.8286881
C	27.5900176	37.7342598	33.0323442
C	26.1834037	39.0612337	33.9862982
N	27.3831959	38.5246907	34.1373472
H	25.7552637	37.7484813	30.1640163
H	27.1995647	36.7735530	30.4724756
H	28.5129414	37.1751945	32.9071886
H	25.7230693	39.7271295	34.7145860
H	24.6777262	38.9008062	32.4975181
C	31.6834326	35.6461216	35.1457859
C	30.6831811	36.3705760	36.0528239
O	29.7734852	37.0864018	35.5364653
O	30.8826456	36.2356152	37.2900963
H	31.9348694	34.6706187	35.5894226
H	32.6178466	36.2358929	35.1792435
C	23.5214402	34.8710865	36.9332587
C	24.9263420	35.2953345	36.6534650
N	25.9002460	34.4814411	36.1088883
C	25.5341918	36.5212873	36.8105200
C	27.0170369	35.2249540	35.9353712
N	26.8276345	36.4618666	36.3554001
H	23.4757236	33.8355225	37.3101104
H	23.0988316	35.5119280	37.7185543
H	25.1195873	37.4463802	37.2076103
H	27.9360984	34.8392277	35.5013374
H	25.7889020	33.5117719	35.7963515
Fe	28.1326045	38.1751452	36.2640194
O	30.2083245	39.7332940	39.6510882
O	27.2717212	39.8213488	36.7437539
C	37.0212679	44.0269500	33.7565733
C	31.0069529	43.6557725	34.4024788
C	29.0317960	44.0640448	35.8382010
C	28.5515123	45.1995417	36.7729364
C	28.4456174	43.1316628	36.9481366
C	35.5142968	44.4237951	33.9087422
C	34.6554049	43.5029397	33.0367337
C	27.8374520	44.3873576	39.0548592
C	26.5604014	44.9140109	39.7734764
C	28.3134231	42.9817651	39.5349412
C	27.1770526	41.9503137	39.5393809
C	29.0073156	42.9879017	40.8907994
C	33.1774408	43.4269065	33.2735468
C	32.4721736	43.9035014	34.3090745
N	30.4376003	44.0985743	35.5614652
N	35.4275780	45.8624007	33.4982565
N	27.8432230	44.3265903	37.5844569

O	30.3491702	43.0952468	33.5232509
O	28.8171203	46.3711039	36.8697415
O	37.7359044	44.8649655	33.1698888
O	37.3298285	42.9029507	34.2042496
O	26.6612759	44.8968021	41.0293599
O	25.6062216	45.3441202	39.0982571
S	29.6118917	42.5344846	38.2342849
H	28.4783572	43.9750696	34.8918106
H	29.2843028	38.3810231	38.8461120
H	27.7756578	42.3125287	36.6435361
H	35.2424743	44.3325413	34.9690968
H	35.0584042	42.4829849	33.1712650
H	34.8410683	43.7398679	31.9726420
H	28.6100219	45.1116464	39.3494420
H	27.0769291	39.8739722	37.6879478
H	32.6096206	42.8707796	32.5183147
H	32.9424996	44.4769650	35.1144365
H	31.0367243	44.5297083	36.2566546
H	31.1524262	39.5681914	39.5094252
H	34.6630775	46.0652972	32.8230247
H	36.3810316	46.0135579	33.0676125
H	35.3066932	46.5388840	34.2769850
H	27.5647695	40.9590868	39.8192114
H	26.4229209	42.2484710	40.2853150
H	26.6828598	41.8660185	38.5625056
H	28.2556189	43.1965960	41.6615284
H	29.7982047	43.7541936	40.9358145
H	29.4497195	41.9974868	41.0691146
O	28.8713403	37.6707746	38.3125457
H	29.6296578	37.0763500	38.0703825
H	29.9844133	40.4202256	38.9876089
H	25.5858173	36.2071886	30.9686045
H	31.3867408	35.4861731	34.1092254
H	22.8652565	34.8907164	36.0631409

RC-I’

C	26.2348644	37.0528895	30.8816720
C	26.4237873	37.7777471	32.1783296
N	25.4986090	38.6375571	32.7417494
C	27.4542228	37.7165870	33.0895624
C	25.9782802	39.0424221	33.9405019
N	27.1595594	38.4987240	34.1822351
H	25.7649130	37.6919039	30.1167901
H	27.2214658	36.7587539	30.4921969
H	28.3833671	37.1547780	33.0328425
H	25.4432984	39.7282452	34.5947969
H	24.5929140	38.9049659	32.3473798
C	31.8135457	35.6485898	35.1155096
C	30.7907509	36.4290431	35.9437133
O	29.7432340	36.8791789	35.3847301
O	31.0764261	36.5849391	37.1601571
H	32.0120356	34.6786261	35.6001526
H	32.7653484	36.2036749	35.1842959
C	23.6498190	34.9477590	36.9662741
C	25.0470446	35.4060480	36.6938181
N	26.0382976	34.6203538	36.1393450
C	25.6429125	36.6312648	36.9026237
C	27.1512376	35.3750028	36.0119343
N	26.9481741	36.6011192	36.4704739
H	23.6341898	33.9124620	37.3460116
H	23.2090001	35.5775285	37.7511685
H	25.1985634	37.5260353	37.3359528
H	28.0784796	35.0191451	35.5693709
H	25.9381707	33.6509309	35.8144681
Fe	28.3447816	38.2701637	36.0185242
O	30.5700505	36.0340031	42.0475994
O	29.4403329	35.8319783	42.3960898
C	36.9369396	44.0442465	33.7908403
C	30.9163362	43.4286682	34.6050330
C	29.0007170	43.1643159	36.1873466
C	28.9223077	43.2359305	37.7304619
C	28.4213496	41.8255350	35.6517576
C	35.4408248	44.4722205	33.9817601
C	34.5327647	43.5911889	33.1145812
C	27.4346605	43.5988622	39.6847358
C	26.6116586	44.8756793	39.9803653
C	26.6938048	42.3502038	40.2533208
C	27.4719090	41.0572699	39.9785414
C	26.3839781	42.4801550	41.7510954
C	33.0494760	43.5538721	33.3779916
C	32.4000412	43.6317812	34.5499239
N	30.3943643	43.4025778	35.8518018
N	35.3854617	45.9159617	33.5803413
N	27.6938393	43.4811313	38.2510130
O	30.2246360	43.2974645	33.5860608
O	29.9260402	43.0457932	38.4102230
O	37.6552750	44.8725305	33.1920455
O	37.2380850	42.9139407	34.2251354
O	26.8712293	45.4697254	41.0547116
O	25.7037719	45.1837426	39.1636895
S	29.5433182	40.3875327	35.9300993
H	28.3797364	43.9666254	35.7519019
H	27.4450338	41.6702502	36.1377847
H	28.2447420	41.9437827	34.5739160
H	35.1905946	44.3829788	35.0475674
H	34.9083376	42.5591838	33.2252678
H	34.7091752	43.8497207	32.0563789
H	28.4102241	43.6882883	40.1827386
H	25.7266768	42.2986985	39.7209390
H	32.4213761	43.3670835	32.4990551
H	32.9246158	43.7981685	35.4972224
H	31.0055054	43.3350203	36.6663544
H	26.9413534	43.8098754	37.6502386
H	34.6280569	46.1330823	32.9029377
H	36.3422666	46.0406023	33.1437179
H	35.2850532	46.5922442	34.3613053
H	26.9119134	40.1807493	40.3448314
H	27.6685002	40.9013077	38.9074451
H	28.4482393	41.0706430	40.4911259

H	25.7387261	43.3368358	41.9827719
H	27.3110405	42.5906805	42.3389100
H	25.8604014	41.5794328	42.1081686
O	29.1421724	38.1571032	38.0774083
H	29.9059451	37.5367866	37.8842294
H	29.5445106	39.0455678	38.0521789
H	25.6034736	36.1683784	30.9656536
H	31.5533994	35.4655213	34.0729757
H	22.9825446	34.9423444	36.1044256

TS-I’

C	26.2399987	37.0805479	30.9444188
C	26.4198645	37.7856808	32.2536751
N	25.4903329	38.6371874	32.8219342
C	27.4482580	37.7203275	33.1679896
C	25.9659471	39.0322550	34.0265748
N	27.1484438	38.4910398	34.2677007
H	25.7751362	37.7320706	30.1867492
H	27.2294836	36.7935781	30.5571935
H	28.3792070	37.1614895	33.1081873
H	25.4260962	39.7098854	34.6856734
H	24.5867903	38.9063202	32.4244335
C	31.8398227	35.6397608	35.0860743
C	30.8482945	36.4244946	35.9433015
O	29.7625256	36.8491822	35.4390771
O	31.2054833	36.6048791	37.1370971
H	32.0333303	34.6637139	35.5608209
H	32.7995149	36.1814784	35.1430173
C	23.6183477	34.8932503	36.9307697
C	25.0156562	35.3525041	36.6607459
N	26.0303954	34.5623491	36.1590247
C	25.5921074	36.5864009	36.8566292
C	27.1427329	35.3209495	36.0562475
N	26.9155215	36.5555277	36.4821318
H	23.6037680	33.8514028	37.2911000
H	23.1849418	35.5095204	37.7301750
H	25.1218793	37.4843345	37.2496766
H	28.0864323	34.9623183	35.6528839
H	25.9447855	33.5887235	35.8417780
Fe	28.3351436	38.2154200	36.1229013
O	26.9684891	37.6254748	39.8717630
O	26.7093328	38.3642181	38.9625985
C	36.9268091	44.0355322	33.7933010
C	30.9086858	43.3912810	34.6205363
C	28.9967508	43.1640326	36.2203532
C	28.9379839	43.2750407	37.7636600
C	28.3936368	41.8205897	35.7226558
C	35.4293562	44.4598983	33.9862956
C	34.5219785	43.5762486	33.1204196
C	27.4774166	43.7055252	39.7274556
C	26.6365007	44.9734724	39.9937954
C	26.7712757	42.4594908	40.3390445
C	27.6373777	41.2069766	40.1777274
C	26.3773704	42.6416737	41.8105130
C	33.0397092	43.5260862	33.3886261
C	32.3910969	43.6049547	34.5610069
N	30.3903489	43.3784920	35.8691941
N	35.3707714	45.9035188	33.5849250
N	27.7193163	43.5416435	38.2949409
O	30.2161118	43.2415068	33.6047988
O	29.9499849	43.0994779	38.4346632
O	37.6427298	44.8651356	33.1936812
O	37.2315632	42.9060569	34.2277393
O	26.8926572	45.5941016	41.0538798
O	25.7189890	45.2469608	39.1758898
S	29.4865788	40.3736155	36.0545131
H	28.3813472	43.9658200	35.7726114
H	27.4140475	41.6962573	36.2110853
H	28.2216880	41.9091456	34.6410687
H	35.1809208	44.3703504	35.0525370
H	34.9048273	42.5462443	33.2256424
H	34.6915682	43.8396582	32.0622536
H	28.4563617	43.8290302	40.2110501
H	25.8373664	42.3228169	39.7628150
H	32.4119368	43.3265581	32.5121529
H	32.9149513	43.7844246	35.5062701
H	31.0044883	43.3314957	36.6830376
H	26.9561445	43.8479892	37.6965462
H	34.6134296	46.1187107	32.9068122
H	36.3271905	46.0301887	33.1480928
H	35.2692812	46.5805286	34.3651357
H	27.1186731	40.3294858	40.5905719
H	27.8788139	40.9901077	39.1269686
H	28.5921665	41.3220358	40.7170250
H	25.6792590	43.4725488	41.9706933
H	27.2632847	42.8200886	42.4422851
H	25.8771943	41.7325272	42.1791545
O	29.3128815	38.0976443	38.1251241
H	30.0991198	37.5264190	37.8756494
H	29.6504521	39.0119761	38.1027449
H	25.6093501	36.1936331	31.0055061
H	31.5590892	35.4647163	34.0475102
H	22.9456618	34.9078734	36.0732459

IM-I’

C	26.2304094	37.0646444	30.8867534
C	26.4148618	37.7886717	32.1844501
N	25.4777789	38.6322818	32.7554523
C	27.4500492	37.7418422	33.0898365
C	25.9493009	39.0415072	33.9526073
N	27.1424125	38.5161264	34.1856730
H	25.7636243	37.7057290	30.1215682
H	27.2184975	36.7710652	30.5011441
H	28.3885637	37.1976797	33.0280594
H	25.4084311	39.7026423	34.6257530
H	24.5686153	38.8908408	32.3624780
C	31.7365747	35.7203845	35.1659457
C	30.7285253	36.5025872	36.0080446

O	29.6866193	36.9952970	35.4685177
O	31.0256934	36.6352940	37.2235212
H	31.9385183	34.7531951	35.6545046
H	32.6885292	36.2765890	35.2265633
C	23.6485007	34.8825682	36.9499398
C	25.0397126	35.3548322	36.6712094
N	26.0413955	34.5955798	36.0982522
C	25.6053575	36.5914092	36.8806097
C	27.1324183	35.3791959	35.9589237
N	26.9045991	36.5985142	36.4267304
H	23.6386007	33.8375302	37.3021738
H	23.2159834	35.4921661	37.7550381
H	25.1487681	37.4653460	37.3361449
H	28.0620080	35.0452185	35.5053693
H	25.9585313	33.6266381	35.7675782
Fe	28.2378327	38.3743412	36.0511677
O	26.0405687	39.4865607	38.0670495
O	26.6060660	39.5377504	36.9583508
C	36.9693809	44.0528714	33.7693729
C	30.9613407	43.4446155	34.5344284
C	29.0572188	43.2009205	36.1384766
C	29.0058725	43.2979333	37.6835278
C	28.4670698	41.8639633	35.6150576
C	35.4711520	44.4772458	33.9422156
C	34.5776516	43.6066587	33.0501771
C	27.5456199	43.6551897	39.6632602
C	26.6504768	44.8786287	39.9723517
C	26.9144462	42.3484105	40.2352120
C	27.8947842	41.1754796	40.1176532
C	26.4151548	42.4906131	41.6793970
C	33.0946317	43.5564270	33.3054695
C	32.4413494	43.6627874	34.4725552
N	30.4460022	43.4374450	35.7831742
N	35.4177929	45.9255022	33.5573033
N	27.7905090	43.5428604	38.2255265
O	30.2676421	43.2826646	33.5208943
O	30.0300097	43.1366893	38.3423323
O	37.6941196	44.8850484	33.1847882
O	37.2652766	42.9199118	34.2015707
O	26.8799510	45.4629965	41.0590635
O	25.7313669	45.1600881	39.1594098
S	29.5769463	40.4140256	35.8729428
H	28.4286511	43.9986043	35.7052084
H	27.5039144	41.6905182	36.1184591
H	28.2755311	41.9792613	34.5397777
H	35.2050070	44.3736604	35.0027700
H	34.9574135	42.5741364	33.1437085
H	34.7593423	43.8853764	31.9977154
H	28.5189781	43.8172352	40.1471942
H	26.0382358	42.1258080	39.6002834
H	32.4731639	43.3357274	32.4296305
H	32.9625030	43.8651919	35.4145548
H	31.0603419	43.4067368	36.5983078
H	27.0078920	43.8140322	37.6346769
H	34.6638104	46.1502543	32.8783842
H	36.3767077	46.0578147	33.1290937
H	35.3100866	46.5930733	34.3448768
H	27.4145072	40.2325853	40.4217068
H	28.2525773	41.0398163	39.0878669
H	28.7781360	41.3404615	40.7571166
H	25.6091940	43.2295559	41.7830595
H	27.2305737	42.7837189	42.3613855
H	26.0098553	41.5279427	42.0295677
O	29.1048513	38.2243958	38.0407303
H	29.8714963	37.5922332	37.8860187
H	29.5182366	39.1036728	38.1050466
H	25.5979683	36.1803936	30.9654036
H	31.4781112	35.5298020	34.1243401
H	22.9732871	34.9017616	36.0944952

Snapshot 2

RC-I

C	24.2666622	33.7443727	39.6606849
C	25.0756273	34.9251814	40.0934906
N	24.5186467	36.0637554	40.6455366
C	26.4363161	35.1317816	40.1412566
C	25.5146650	36.8882900	41.0166576
N	26.6918686	36.3536790	40.7228180
H	23.3391779	34.0707576	39.1666145
H	24.8380450	33.1527737	38.9301944
H	27.2421400	34.4863357	39.8033374
H	25.3728856	37.8656100	41.4729070
H	23.5201208	36.2733056	40.7630706
C	31.2942348	33.6775521	40.2684864
C	30.7470558	34.8617449	41.0647539
O	29.6025413	35.3377275	40.8061019
O	31.5503064	35.3488843	41.9076302
H	31.8999468	33.0394500	40.9292543
H	32.0003243	34.1140682	39.5388230
C	25.8339433	35.6709433	46.1569228
C	26.8038908	35.6468268	45.0193415
N	27.4311921	34.5080419	44.5475343
C	27.2499594	36.6588641	44.1989970
C	28.1860558	34.8509648	43.4826362
N	28.1009422	36.1482117	43.2460323
H	26.1862439	35.0576589	47.0032592
H	25.7352696	36.7003952	46.5311714
H	26.9953506	37.7157226	44.2257679
H	28.7569147	34.1338171	42.8994032
H	27.3204299	33.5489034	44.8907624
Fe	28.6133025	37.1788663	41.2964433
O	26.7307164	39.4628802	41.7959952
O	27.7743630	38.7712481	42.0769381
C	33.3948253	39.2590058	30.9806231
C	29.2185861	40.6442451	35.6042451
C	28.1128599	40.3758525	37.8312074
C	28.9054207	41.4173253	38.6756294
C	27.6512282	39.1431485	38.6423364

C	32.3748459	40.1971998	31.7096652
C	31.4675434	39.4185602	32.6620388
C	28.7736944	43.0986631	40.4847916
C	27.8035969	44.2943477	40.6624147
C	29.2915763	42.6096939	41.8722921
C	28.1657635	42.3164641	42.8716100
C	30.2516151	41.4250352	41.7306288
C	30.5010187	40.2270929	33.4858762
C	30.2677188	39.9738065	34.7809504
N	28.9258788	39.9354567	36.7205302
N	31.6067460	40.8891151	30.6251567
N	28.2029648	42.0428033	39.6582460
O	28.6392569	41.6892484	35.2792101
O	30.0838150	41.6282842	38.4419837
O	34.1769292	38.6151649	31.7029255
O	33.2952785	39.2569165	29.7315213
O	26.6544242	44.2459010	40.1544812
O	28.2412232	45.2458302	41.3653143
S	29.0437753	38.0222860	39.1162277
H	27.2105848	40.8698522	37.4361376
H	27.1284963	39.4796492	39.5487004
H	26.9386864	38.5678639	38.0325201
H	32.9433286	40.9684891	32.2503056
H	32.1211027	38.8146801	33.3088762
H	30.8902279	38.6947551	32.0526364
H	29.6526326	43.4686103	39.9350448
H	29.8685197	43.4598731	42.2671596
H	29.9160140	41.0161973	32.9964251
H	30.8276726	39.1682590	35.2696577
H	29.5489722	39.1659332	36.9529280
H	27.1918457	41.9574473	39.6638020
H	32.1948028	40.6919671	29.7749605
H	31.4378411	41.8945817	30.8110611
H	30.6840125	40.4550483	30.4296863
H	28.5923341	41.9910894	43.8343343
H	27.5557026	43.2118779	43.0728070
H	27.5089567	41.5112875	42.5095658
H	31.0628481	41.6500541	41.0232347
H	29.7392626	40.5231048	41.3660881
H	30.6942400	41.1713367	42.7064727
O	30.4504678	37.6996230	42.1768603
H	30.5681948	38.0484380	43.0944498
H	30.9885430	36.8547788	42.1637259
H	23.9505152	33.1147298	40.4923507
H	30.5735899	33.0499207	39.7442565

TS-I

C	24.2546456	33.7290167	39.6591057
C	25.0681090	34.9102637	40.0819494
N	24.5181083	36.0570289	40.6247913
C	26.4299858	35.1095408	40.1281184
C	25.5208658	36.8786380	40.9866848
N	26.6943468	36.3338229	40.6975351
H	23.3249150	34.0534774	39.1682620
H	24.8214685	33.1330361	38.9285273
H	27.2318962	34.4557910	39.7966123
H	25.3767638	37.8662538	41.4160899
H	23.5206083	36.2716956	40.7428114
C	31.2997140	33.6687767	40.2669044
C	30.7509642	34.8592824	41.0551139
O	29.6126867	35.3384770	40.7871740
O	31.5528408	35.3451620	41.9029788
H	31.9002246	33.0310018	40.9326752
H	32.0113140	34.0980214	39.5382387
C	25.8246391	35.6902633	46.1558014
C	26.7953182	35.6731024	45.0188609
N	27.4374425	34.5370243	44.5593179
C	27.2311245	36.6833499	44.1903256
C	28.1928659	34.8771923	43.4949307
N	28.0933952	36.1719347	43.2480448
H	26.1812085	35.0795152	47.0020685
H	25.7180947	36.7187127	46.5303597
H	26.9668783	37.7390065	44.2004958
H	28.7747936	34.1621157	42.9197205
H	27.3320082	33.5794328	44.9091045
Fe	28.5935263	37.1838847	41.3385717
O	26.9016724	39.5575135	41.0408736
O	27.7177513	38.7910870	41.8021593
C	33.3868454	39.2595272	30.9828181
C	29.2151978	40.6719331	35.6086584
C	28.1425382	40.3480087	37.8667087
C	28.9248397	41.4215468	38.6864017
C	27.7801858	39.0813341	38.6649683
C	32.3696326	40.2041540	31.7080194
C	31.4570537	39.4346113	32.6626649
C	28.7774289	43.0992086	40.4999013
C	27.8039404	44.2919912	40.6786275
C	29.2755706	42.5932229	41.8878346
C	28.1338273	42.2572851	42.8555349
C	30.2603802	41.4291439	41.7444114
C	30.4889764	40.2501646	33.4788144
C	30.2670836	40.0114781	34.7783374
N	28.9471561	39.9521800	36.7244783
N	31.6042841	40.8946739	30.6209451
N	28.2192151	42.0528024	39.6573712
O	28.6195177	41.7082626	35.2884863
O	30.0955119	41.6519816	38.4328046
O	34.1683682	38.6171711	31.7068231
O	33.2865179	39.2530132	29.7336831
O	26.6571516	44.2428978	40.1676577
O	28.2397590	45.2409252	41.3869755
S	29.0838687	37.9791319	39.0625156
H	27.2098059	40.8068670	37.5028558
H	27.2747201	39.4132185	39.9274390
H	26.8721715	38.5882573	38.2848557
H	32.9415632	40.9751927	32.2455044
H	32.1068060	38.8342465	33.3167083
H	30.8794694	38.7082356	32.0563231
H	29.6618874	43.4740959	39.9635704

H	29.8255998	43.4475221	42.3116163
H	29.8932039	41.0256145	32.9810426
H	30.8408670	39.2167177	35.2682716
H	29.5950501	39.1996689	36.9377865
H	27.2226475	41.8838883	39.7307924
H	32.1926798	40.6958275	29.7717243
H	31.4357938	41.9003013	30.8060056
H	30.6813835	40.4608571	30.4255713
H	28.5468185	41.9255468	43.8216522
H	27.4988201	43.1351797	43.0573556
H	27.5041430	41.4441109	42.4632069
H	31.0757518	41.6778662	41.0495908
H	29.7696174	40.5218589	41.3643972
H	30.6960216	41.1724408	42.7224848
O	30.4564427	37.6974588	42.1675698
H	30.5957408	38.0553903	43.0786991
H	30.9911212	36.8498514	42.1497541
H	23.9431977	33.1038821	40.4959295
H	30.5782159	33.0414897	39.7434365
H	24.8384280	35.2906932	45.9195847

IM-I

C	24.2377852	33.7624255	39.6548430
C	25.0695317	34.9177921	40.1125846
N	24.5404875	36.0683510	40.6679271
C	26.4354245	35.0720141	40.2020469
C	25.5614259	36.8500331	41.0771702
N	26.7229560	36.2729261	40.8053157
H	23.3120310	34.1135566	39.1741126
H	24.7954270	33.1790620	38.9066425
H	27.2271384	34.3992014	39.8821361
H	25.4481104	37.8071229	41.5848366
H	23.5465361	36.3052041	40.7681293
C	31.3444108	33.5752825	40.2779019
C	30.7941420	34.7679584	41.0732860
O	29.6333741	35.2013305	40.8751542
O	31.6393684	35.2951988	41.8613499
H	31.9183131	32.9181437	40.9494673
H	32.0829405	33.9947422	39.5710943
C	25.7553446	35.6112690	46.2808136
C	26.7751789	35.6165185	45.1893774
N	27.4420161	34.4948751	44.7261839
C	27.2436473	36.6522533	44.4137690
C	28.2439142	34.8723800	43.7076307
N	28.1498692	36.1733750	43.4960926
H	26.0719811	34.9834443	47.1309824
H	25.6391941	36.6334978	46.6688015
H	26.9483064	37.6994658	44.4016600
H	28.8529298	34.1799566	43.1312238
H	27.3234673	33.5258844	45.0350772
Fe	28.5709866	37.1048712	41.5831386
O	26.6806854	39.1682299	42.6828230
O	27.7539017	38.9191411	41.7109604
C	33.3912693	39.2623797	30.9526518
C	29.2299026	40.6862981	35.5686683
C	28.1937160	40.3811390	37.8226220
C	28.9562683	41.5515365	38.5749063
C	28.1041627	39.2856836	38.8475755
C	32.3711460	40.2111296	31.6690833
C	31.4393995	39.4405992	32.6039460
C	28.7699975	43.2221661	40.3909894
C	27.7821481	44.3964818	40.5882243
C	29.2106541	42.6668251	41.7813818
C	28.0144113	42.3481957	42.6864093
C	30.1549849	41.4662432	41.6599590
C	30.4852820	40.2631411	33.4284013
C	30.2451384	39.9997548	34.7201538
N	28.9344697	39.9552971	36.6732365
N	31.6259237	40.9140006	30.5759622
N	28.2242803	42.1998111	39.5074408
O	28.6884716	41.7639726	35.2973301
O	30.1309878	41.7335169	38.3341325
O	34.1601440	38.6139663	31.6844255
O	33.3045357	39.2608970	29.7029579
O	26.6279677	44.3220271	40.0956151
O	28.2076307	45.3408736	41.3045535
S	28.9796998	37.9068231	38.8413279
H	27.1761751	40.7253783	37.5650326
H	26.9376238	40.0440462	43.0060006
H	27.5080343	39.4799881	39.7568024
H	32.9414267	40.9734809	32.2205641
H	32.0737802	38.8160771	33.2504720
H	30.8510507	38.7364706	31.9821335
H	29.6712164	43.6158395	39.8998135
H	29.7672362	43.4939856	42.2471299
H	29.9246917	41.0725874	32.9441445
H	30.7874438	39.1737614	35.1941300
H	29.4702292	39.1024208	36.8295165
H	27.2106159	42.1327137	39.4554910
H	32.2211309	40.7086163	29.7322960
H	31.4680511	41.9210892	30.7619343
H	30.6991450	40.4917438	30.3738866
H	28.3579967	41.8840427	43.6250055
H	27.4444834	43.2513179	42.9558644
H	27.3333181	41.6443647	42.1819615
H	30.9529538	41.6501083	40.9255718
H	29.6084417	40.5550895	41.3729908
H	30.6219112	41.2549001	42.6345024
O	30.5633166	37.6857063	42.0311075
H	30.7714509	38.0800624	42.9124043
H	31.0757330	36.8244648	42.0277184
H	23.9221764	33.1190165	40.4761121
H	30.6197518	32.9660392	39.7377547
H	24.7787014	35.2213794	45.9940711

TS-II

C	24.2128679	33.8273763	39.6400774
C	25.0426986	34.9833508	40.0996534

N	24.5310365	36.0998940	40.7321369
C	26.4089787	35.1594082	40.1103523
C	25.5676002	36.8741186	41.1205644
N	26.7176114	36.3353492	40.7515184
H	23.2778983	34.1751043	39.1741977
H	24.7679070	33.2618879	38.8766265
H	27.1849670	34.5086638	39.7139193
H	25.4664831	37.8032580	41.6769777
H	23.5402725	36.3219999	40.8841658
C	31.3886332	33.5886110	40.2390076
C	30.8237593	34.7666402	41.0508369
O	29.7103087	35.2693617	40.6904251
O	31.5518325	35.1913311	41.9733550
H	31.9880986	32.9402734	40.8952366
H	32.0974632	34.0258263	39.5116775
C	25.8582249	35.6279855	46.1333703
C	26.8500578	35.5612647	45.0155538
N	27.4586757	34.4018553	44.5690095
C	27.3723563	36.5581237	44.2223610
C	28.2812583	34.7248995	43.5461278
N	28.2550651	36.0229553	43.3122417
H	26.1909376	35.0330030	47.0012927
H	25.7740587	36.6679950	46.4822333
H	27.1713686	37.6256299	44.2457655
H	28.8650615	33.9919370	42.9945179
H	27.3084987	33.4468634	44.9051358
Fe	28.7071805	36.9990061	41.3406716
O	28.9012135	39.0308076	43.1928267
O	27.9690631	38.6400728	42.0194838
C	33.3953621	39.2616740	30.9523098
C	29.2286999	40.6623540	35.5676353
C	28.1730571	40.3894151	37.8090220
C	28.9249606	41.5675471	38.5504641
C	28.0654159	39.3098127	38.8542285
C	32.3741860	40.2064721	31.6721119
C	31.4465993	39.4306621	32.6065170
C	28.7262796	43.2436016	40.3569008
C	27.7270081	44.4039835	40.5790274
C	29.2075112	42.6904121	41.7306235
C	28.0517278	42.2097092	42.6170877
C	30.2952060	41.6235199	41.5732882
C	30.4925919	40.2477593	33.4355385
C	30.2455516	39.9739082	34.7239906
N	28.9328964	39.9424279	36.6792504
N	31.6260418	40.9095114	30.5810659
N	28.1828336	42.2146018	39.4814235
O	28.6827207	41.7361853	35.2877079
O	30.1025793	41.7553739	38.3293549
O	34.1664563	38.6134735	31.6819059
O	33.3056197	39.2617505	29.7028271
O	26.5727115	44.3282504	40.0869745
O	28.1506955	45.3428563	41.3028062
S	29.0093344	37.9718346	38.9345146
H	27.1628971	40.7299696	37.5200056
H	28.9515277	39.9923828	43.0628024
H	27.3928846	39.5313197	39.6905806
H	32.9430351	40.9691613	32.2242648
H	32.0832320	38.8046323	33.2492764
H	30.8581623	38.7280614	31.9832146
H	29.6104442	43.6602159	39.8528114
H	29.6608006	43.5553278	42.2345111
H	29.9361181	41.0635181	32.9571677
H	30.7815446	39.1421138	35.1954003
H	29.4786048	39.1004988	36.8535899
H	27.1689702	42.1763352	39.3971670
H	32.2203479	40.7041224	29.7363544
H	31.4679927	41.9164974	30.7670460
H	30.6991349	40.4873624	30.3797085
H	28.4277266	41.9293542	43.6163519
H	27.3043777	43.0010391	42.7855204
H	27.5404047	41.3335726	42.1829271
H	31.1172730	41.9847813	40.9400655
H	29.9130904	40.6989242	41.1143133
H	30.7191495	41.3516180	42.5528627
O	30.5606022	37.8573154	41.9624057
H	29.9309140	38.4583915	42.7280285
H	31.0174496	37.1045274	42.3785259
H	23.9134485	33.1651812	40.4524276
H	30.6624949	32.9708151	39.7106910
H	24.8628769	35.2461019	45.9064026

IM-II

C	24.3065022	33.7344443	39.6629453
C	25.1530573	34.8844850	40.1076209
N	24.6372827	36.0487628	40.6482610
C	26.5214105	35.0247184	40.1850235
C	25.6649363	36.8248301	41.0401608
N	26.8197871	36.2323700	40.7710263
H	23.3873449	34.0923455	39.1759350
H	24.8593951	33.1364892	38.9234852
H	27.3069561	34.3438059	39.8714544
H	25.5674438	37.7957419	41.5184952
H	23.6437533	36.2923222	40.7506072
C	31.2502309	33.6753392	40.1702084
C	30.6920314	34.8189396	41.0209711
O	29.5156505	35.2692446	40.7957907
O	31.4929140	35.2970790	41.8510455
H	31.9065996	33.0565872	40.8001991
H	31.9121425	34.1542022	39.4259773
C	25.8139689	35.6503682	46.1982843
C	26.8097522	35.6101292	45.0845121
N	27.4714340	34.4672453	44.6716927
C	27.2605717	36.6008328	44.2425720
C	28.2521986	34.7830822	43.6191881
N	28.1462244	36.0688285	43.3368605
H	26.1482070	35.0437351	47.0562113
H	25.7103882	36.6829916	46.5624920
H	26.9952861	37.6549717	44.2186177
H	28.8610754	34.0605135	43.0825317

H	27.3640645	33.5162355	45.0374750
Fe	28.6433850	37.0291374	41.4590637
O	30.3072850	39.2498619	44.0228890
O	27.8649030	38.4283549	41.7874327
C	33.4144147	39.2765878	30.9120184
C	29.2085422	40.7017031	35.4783891
C	28.1527996	40.3837430	37.7172080
C	28.9282441	41.5489900	38.4811735
C	28.0371838	39.2811740	38.7268882
C	32.3941910	40.2336953	31.6159580
C	31.4474831	39.4654304	32.5369230
C	28.7486164	43.1845804	40.3332139
C	27.7469494	44.3399623	40.5825647
C	29.2340872	42.5769103	41.6833770
C	28.0872115	42.0546639	42.5555954
C	30.3154788	41.5126511	41.4822552
C	30.4841414	40.2867826	33.3499427
C	30.2342511	40.0221384	34.6395823
N	28.8915499	39.9563602	36.5694500
N	31.6659092	40.9435120	30.5159518
N	28.1994617	42.1823976	39.4242185
O	28.6724835	41.7834378	35.2160573
O	30.0999547	41.7269313	38.2304897
O	34.1697984	38.6235380	31.6538757
O	33.3382966	39.2721639	29.6622435
O	26.5950382	44.2782775	40.0790566
O	28.1644951	45.2624918	41.3291719
S	28.8420416	37.8631331	38.6925955
H	27.1413096	40.7469350	37.4592721
H	29.3820035	39.5226710	44.0304796
H	27.4352688	39.5097080	39.6164251
H	32.9627411	40.9911608	32.1754476
H	32.0721416	38.8384496	33.1904533
H	30.8646786	38.7648550	31.9061352
H	29.6315019	43.6148815	39.8385097
H	29.6884551	43.4262678	42.2165187
H	29.9242879	41.0925936	32.8591862
H	30.7782860	39.1992643	35.1175637
H	29.4264395	39.1033904	36.7188129
H	27.1842567	42.1402140	39.3583542
H	32.2698646	40.7437123	29.6781677
H	31.5066929	41.9493635	30.7068755
H	30.7406236	40.5237445	30.3026518
H	28.4885537	41.7294656	43.5277854
H	27.3322763	42.8319989	42.7561583
H	27.5954746	41.1797284	42.0991484
H	31.1698171	41.9180092	40.9215009
H	29.9330134	40.6378228	40.9342751
H	30.6628912	41.1202356	42.4478202
O	30.2962432	37.7273088	41.7701859
H	30.3677812	38.7122882	43.1963449
H	30.8945343	36.9537771	41.8983340
H	23.9812453	33.1026957	40.4894855
H	30.5456700	33.0180025	39.6607287
H	24.8301993	35.2570381	45.9422454

IM-III

C	24.3538647	33.8126440	39.6773617
C	25.2038112	34.9498982	40.1495130
N	24.6898932	36.1167140	40.6872291
C	26.5721375	35.0785525	40.2459872
C	25.7200213	36.8837744	41.0938545
N	26.8736662	36.2818089	40.8395397
H	23.4404367	34.1856008	39.1897802
H	24.9085844	33.2253415	38.9304409
H	27.3549531	34.3920307	39.9380174
H	25.6253261	37.8550414	41.5718494
H	23.6955953	36.3630054	40.7766633
C	31.2775407	33.7123355	40.2016773
C	30.7298417	34.8211322	41.1043397
O	29.5566211	35.2872627	40.9050973
O	31.5346908	35.2558874	41.9551843
H	31.9528183	33.0788972	40.7960068
H	31.9148485	34.2199771	39.4548080
C	25.7588752	35.6364097	46.2633121
C	26.7679977	35.6047559	45.1624825
N	27.4111355	34.4589228	44.7298329
C	27.2365415	36.6045311	44.3405419
C	28.1970737	34.7796137	43.6837317
N	28.1121200	36.0714261	43.4268395
H	26.0780839	35.0231156	47.1222481
H	25.6504993	36.6666901	46.6319161
H	26.9980473	37.6656808	44.3398793
H	28.7916460	34.0572365	43.1307589
H	27.2916916	33.5029295	45.0769180
Fe	28.6928349	37.0381010	41.5947549
O	31.1577984	39.6870936	40.5026991
O	27.9369501	38.4537456	41.8575117
C	33.4704534	39.2892605	30.8584635
C	29.2340195	40.5906084	35.3987616
C	28.2238819	40.3041493	37.6412706
C	28.9792859	41.5310576	38.3110285
C	28.1830285	39.2639165	38.7223235
C	32.4342529	40.2246849	31.5616511
C	31.4786669	39.4231422	32.4432711
C	28.6921622	43.0872602	40.2177269
C	27.7502939	44.2739364	40.5310328
C	28.9134770	42.1635630	41.4583143
C	29.8663488	42.7542858	42.4907191
C	27.5991060	41.7365184	42.1143558
C	30.5044125	40.2192729	33.2647061
C	30.2573197	39.9279691	34.5482993
N	28.9297036	39.8387545	36.4887327
N	31.7163557	40.9546683	30.4672237
N	28.1919555	42.2745147	39.1158523
O	28.6870442	41.6710012	35.1502149
O	30.1685577	41.6593025	38.1308589
O	34.2217171	38.6344943	31.6039617
O	33.4067216	39.2945194	29.6087700

O	26.5916794	44.2786911	40.0369020
O	28.2006693	45.1404878	41.3273733
S	28.9732861	37.8395972	38.7331574
H	27.1883825	40.6166175	37.4074046
H	30.8684546	38.9819320	41.1284137
H	27.6407612	39.5548585	39.6320488
H	32.9849889	40.9724884	32.1509906
H	32.0973289	38.7823942	33.0887108
H	30.9101478	38.7378120	31.7834571
H	29.6693039	43.4900841	39.9174776
H	29.4049120	41.2635666	41.0543317
H	29.9410129	41.0314258	32.7886225
H	30.8056495	39.0996922	35.0115696
H	29.4848462	39.0011275	36.6506358
H	27.1815694	42.2056213	39.0119324
H	32.3262887	40.7756640	29.6304430
H	31.5535224	41.9560789	30.6777173
H	30.7921048	40.5389128	30.2418866
H	30.0934137	42.0077715	43.2667200
H	30.8118127	43.0785286	42.0268352
H	29.4088144	43.6244780	42.9775517
H	26.8593620	41.4029386	41.3686757
H	27.1570857	42.5683911	42.6892657
H	27.7712195	40.8888636	42.7923832
O	30.3327102	37.6702283	42.1256762
H	31.4670848	40.3908929	41.0859077
H	30.9191081	36.8780261	42.1667282
H	24.0172499	33.1649728	40.4868551
H	30.5680163	33.0634106	39.6883171
H	24.7804809	35.2451126	45.9845679

TS-III

C	24.2457440	33.6847082	39.6314279
C	25.0668177	34.8763985	40.0067408
N	24.5274642	36.0421795	40.5209251
C	26.4301212	35.0719419	40.0211188
C	25.5352173	36.8743294	40.8344343
N	26.7015022	36.3143584	40.5443654
H	23.3071028	33.9976908	39.1512092
H	24.7997720	33.0700354	38.9067431
H	27.2263754	34.4071757	39.7002334
H	25.4122565	37.8586536	41.2775845
H	23.5318318	36.2566564	40.6620331
C	31.2209455	33.8243074	40.1691233
C	30.6582944	35.0598787	40.8829093
O	29.4916474	35.4900399	40.5804050
O	31.4436829	35.6156272	41.6803636
H	31.8232612	33.2430751	40.8835721
H	31.9389971	34.2189413	39.4274244
C	25.9109982	35.7415218	46.0511445
C	26.8507379	35.7081854	44.8880646
N	27.4731108	34.5619303	44.4217783
C	27.2657001	36.7076152	44.0369487
C	28.1928895	34.8844784	43.3283807
N	28.0895713	36.1776744	43.0709680
H	26.2921287	35.1427388	46.8951348
H	25.8131945	36.7748672	46.4152861
H	27.0239360	37.7680564	44.0491515
H	28.7517863	34.1585791	42.7440215
H	27.3779411	33.6113507	44.7929573
Fe	28.5017535	37.1948079	41.1820304
O	30.6244460	40.1677826	40.5372615
O	27.6575278	38.5141505	41.5835363
C	33.4658950	39.2420621	30.8886822
C	29.4486263	40.7483729	35.5330961
C	28.2929032	40.5791802	37.7382666
C	28.5162146	41.9664172	38.5411810
C	28.0304742	39.4776664	38.6927142
C	32.4408473	40.1612884	31.6254335
C	31.5726710	39.3586541	32.5943867
C	28.8652793	42.8806515	40.7995366
C	27.8695170	44.0860864	40.7204290
C	28.6967368	42.2046526	42.2027633
C	29.1563562	43.0617853	43.3708366
C	27.2612992	41.7423730	42.4373015
C	30.6092227	40.1601786	33.4181064
C	30.5083759	40.0517154	34.7495105
N	29.3523073	40.2768943	36.8082507
N	31.6400587	40.8386181	30.5558524
N	28.7877136	41.7800485	39.8326866
O	28.7058528	41.6132559	35.0736719
O	28.3725980	42.9944237	37.8982758
O	34.2575035	38.6075284	31.6102021
O	33.3571988	39.2370177	29.6420239
O	26.7245668	43.9661077	40.2336566
O	28.2887520	45.1284529	41.3124221
S	28.8789644	38.0652336	38.8243517
H	27.3617251	40.7969264	37.1825606
H	30.4322961	39.1673198	41.0286331
H	27.2277237	39.6615397	39.4131933
H	33.0001467	40.9443230	32.1583946
H	32.2569464	38.7832929	33.2347866
H	31.0044555	38.6153198	32.0009675
H	29.8706591	43.3458033	40.7627422
H	29.3228259	41.3019792	42.1844077
H	29.9147535	40.8416072	32.9101512
H	31.1666541	39.3578511	35.2842911
H	29.9731364	39.5210468	37.0829116
H	29.7167551	40.8061240	40.1918619
H	32.2142568	40.6552774	29.6941713
H	31.4589366	41.8415842	30.7465381
H	30.7170849	40.3959666	30.3813374
H	29.0866357	42.4939886	44.3110399
H	30.1978118	43.4004109	43.2505440
H	28.5061745	43.9440970	43.4453402
H	26.8477873	41.2508885	41.5459456
H	26.6297627	42.6094506	42.6881889
H	27.2207445	41.0258967	43.2698956
O	30.1796206	38.0324979	41.6296829

H	31.0642722	40.6992255	41.2178069
H	30.8552308	37.3185962	41.6956360
H	23.9496566	33.0803120	40.4888104
H	30.5290660	33.1425884	39.6745232
H	24.9181887	35.3383860	45.8514496

IM-IV

C	24.3904918	33.8539993	39.6026055
C	25.2057898	35.0437532	40.0014161
N	24.6549104	36.2015083	40.5202259
C	26.5662377	35.2662683	39.9959898
C	25.6555298	37.0505232	40.8192537
N	26.8297441	36.5159141	40.5110094
H	23.4600070	34.1772285	39.1113537
H	24.9539264	33.2543022	38.8726111
H	27.3644912	34.6093361	39.6630559
H	25.5174925	38.0258793	41.2775457
H	23.6555292	36.3935381	40.6667242
C	31.2938745	33.9504089	40.1615755
C	30.7454340	35.1180219	40.9884845
O	29.6973475	35.7324502	40.5966898
O	31.4484091	35.4442684	41.9757296
H	31.9608897	33.3494663	40.7958112
H	31.9291531	34.4069436	39.3799844
C	25.9254662	35.7011966	46.0568483
C	26.8626160	35.6709065	44.8916217
N	27.4742096	34.5306261	44.3996480
C	27.2760902	36.6821335	44.0573475
C	28.1879478	34.8684281	43.3069158
N	28.0878473	36.1664190	43.0742862
H	26.2922472	35.0774808	46.8888032
H	25.8510503	36.7293856	46.4404726
H	27.0446305	37.7430203	44.0981094
H	28.7513143	34.1509094	42.7175065
H	27.3764505	33.5736867	44.7537683
Fe	28.6431644	37.3691952	41.3608805
O	31.7281677	39.8029232	41.6167863
O	27.7516561	38.5996976	41.9165845
C	33.3031686	39.2052020	31.1209663
C	29.0207690	40.6277503	35.6660770
C	27.8298628	40.6571440	37.8737504
C	28.0858950	42.1006487	38.3650978
C	28.1424508	40.2426499	39.3614787
C	32.2711002	40.1185102	31.8738222
C	31.3854775	39.3228217	32.8383079
C	28.8679785	42.4670471	40.7862700
C	28.1075192	43.8153027	41.0601092
C	28.7850753	41.5880604	42.0625405
C	29.7206859	42.0677454	43.1680916
C	27.3548201	41.4616657	42.5832278
C	30.3904771	40.1176807	33.6448192
C	30.0830479	39.8723515	34.9286425
N	28.7812523	40.1398522	36.9132712
N	31.4839927	40.8057323	30.7997096
N	28.4183496	41.6868395	39.6236721
O	28.4203656	41.5959648	35.1946647
O	27.9968190	43.1970633	37.8616080
O	34.1064725	38.5684187	31.8245706
O	33.1924985	39.2203492	29.8718973
O	26.9058926	43.9333262	40.7443811
O	28.7770042	44.6577475	41.7147343
S	29.4284297	38.9178162	39.5758931
H	26.7857304	40.4739890	37.5643375
H	30.9542472	38.4729186	42.2644331
H	27.2625353	39.8854027	39.9119482
H	32.8342720	40.8966334	32.4105995
H	32.0526087	38.7386864	33.4900035
H	30.8291821	38.5789713	32.2336975
H	29.9238617	42.7432162	40.6169252
H	29.1138371	40.5830825	41.7661383
H	29.8340854	40.9205426	33.1437493
H	30.5960431	39.0650922	35.4641301
H	29.2982045	39.3203997	37.2332560
H	31.1356288	39.7920451	40.8320986
H	32.0726477	40.6184691	29.9458813
H	31.3030724	41.8099626	30.9839144
H	30.5691398	40.3594961	30.5944555
H	29.7190876	41.3541336	44.0063205
H	30.7549233	42.1946820	42.8040070
H	29.4080907	43.0501100	43.5392249
H	26.6530731	41.2855310	41.7562290
H	27.0449462	42.3825257	43.1025306
H	27.2733946	40.6122270	43.2725525
O	30.3608664	37.7452149	42.5943176
H	31.4019347	40.5394522	42.1559611
H	30.8790762	36.8956919	42.5306171
H	24.0731083	33.2248092	40.4341430
H	30.5889174	33.2689515	39.6854258
H	24.9245542	35.3250978	45.8452070

TS-IV

C	24.5047827	34.1038449	39.6431974
C	25.3200890	35.2625658	40.1299487
N	24.7780541	36.4027090	40.6970768
C	26.6826527	35.4693389	40.1425120
C	25.7899020	37.2280265	41.0385797
N	26.9594928	36.6926300	40.7129395
H	23.5894707	34.4622313	39.1459395
H	25.0850879	33.5496604	38.8901862
H	27.4772014	34.8196305	39.7853821
H	25.6612351	38.1872990	41.5341484
H	23.7772748	36.5876500	40.8421120
C	31.3942111	33.8483636	40.1667829
C	30.8868893	35.0212057	41.0096535
O	29.8154535	35.6277736	40.7000247
O	31.6629286	35.3606766	41.9427365
H	32.0413809	33.2151296	40.7905453
H	32.0485424	34.2955734	39.3956026

C	25.9098299	35.7458317	46.1068784
C	26.8871562	35.7275698	44.9749402
N	27.4963243	34.5865310	44.4809624
C	27.3692545	36.7517752	44.1928424
C	28.2737346	34.9366709	43.4370261
N	28.2185893	36.2426421	43.2374759
H	26.2571403	35.1242382	46.9491979
H	25.8155472	36.7722965	46.4907167
H	27.1701233	37.8184610	44.2545775
H	28.8466133	34.2234573	42.8506273
H	27.3699213	33.6234142	44.8079715
Fe	28.8592332	37.4093733	41.5103014
O	31.7728739	39.8036418	41.5179746
O	28.2206693	38.8982747	42.1075420
C	33.3153463	39.2318271	31.0854965
C	29.0723001	40.7159885	35.6473540
C	27.9015976	40.6492882	37.8646245
C	28.1205186	42.0505269	38.4778242
C	28.1785174	40.1093472	39.3195879
C	32.2899511	40.1624582	31.8256956
C	31.3941266	39.3857158	32.7951044
C	28.8025445	42.2062795	40.9522802
C	28.1693918	43.6530693	41.2022221
C	28.4961332	41.3993069	42.2289425
C	29.4599939	41.6849217	43.3662119
C	27.0478974	41.4522464	42.6638256
C	30.4027499	40.1943281	33.5923972
C	30.1389292	39.9861208	34.8918070
N	28.8722987	40.2101042	36.8906599
N	31.5106101	40.8454218	30.7438246
N	28.3559972	41.5396139	39.7268916
O	28.4317505	41.6686146	35.1918518
O	28.0799085	43.1779624	38.0423133
O	34.1117929	38.5955418	31.7974860
O	33.2073891	39.2350008	29.8363106
O	27.0008339	43.8670872	40.8279245
O	28.8824532	44.4236046	41.8905707
S	29.5386533	38.8667410	39.4647598
H	26.8669754	40.4858649	37.5142901
H	31.2104702	38.4054346	42.2306056
H	27.2912671	39.6645694	39.7867269
H	32.8582188	40.9409581	32.3565546
H	32.0557312	38.8054540	33.4556135
H	30.8342314	38.6387191	32.1978623
H	29.8931750	42.3600090	40.8844999
H	28.5804920	40.1817432	42.0098071
H	29.8144961	40.9627728	33.0739821
H	30.6898024	39.2084937	35.4331198
H	29.4439226	39.4240671	37.2097247
H	31.1656317	39.7236037	40.7422554
H	32.1007108	40.6536003	29.8929868
H	31.3299689	41.8498802	30.9258543
H	30.5959017	40.3985008	30.5394161
H	29.4341284	40.8881717	44.1225143
H	30.4972226	41.8087111	43.0153959
H	29.1950128	42.6376425	43.8501251
H	26.3918260	41.2624622	41.8026270
H	26.7961899	42.4413761	43.0807026
H	26.8530643	40.6861003	43.4253660
O	30.6676860	37.6542401	42.5951224
H	31.3505257	40.4733814	42.0758016
H	31.1796462	36.8006729	42.4806919
H	24.1641148	33.4179886	40.4188351
H	30.6637792	33.2026037	39.6794119
H	24.9181926	35.3602312	45.8701615

IM-V

C	24.4457738	33.9692651	39.6193071
C	25.2707405	35.1377965	40.0621511
N	24.7347203	36.2881578	40.6154983
C	26.6328194	35.3454097	40.0553173
C	25.7464006	37.1196156	40.9313662
N	26.9147599	36.5797414	40.6009486
H	23.5230803	34.3168725	39.1287107
H	25.0108064	33.3913699	38.8728351
H	27.4221749	34.6876871	39.7022435
H	25.6341644	38.0822004	41.4236955
H	23.7360813	36.4817761	40.7650041
C	31.3771982	33.8462014	40.1772120
C	30.8438001	35.0266196	40.9946153
O	29.7892196	35.6367824	40.6322850
O	31.5779758	35.3705197	41.9591842
H	32.0201973	33.2249148	40.8170022
H	32.0382687	34.2870333	39.4080793
C	25.9001589	35.6620186	46.1095678
C	26.8651045	35.6221820	44.9676652
N	27.4709659	34.4768497	44.4800577
C	27.3225958	36.6357070	44.1596888
C	28.2245601	34.8155336	43.4142846
N	28.1564973	36.1173383	43.1947449
H	26.2426883	35.0393106	46.9528940
H	25.8253365	36.6924080	46.4870103
H	27.1075326	37.7001642	44.1903442
H	28.7949068	34.0952891	42.8333979
H	27.3500308	33.5170529	44.8176120
Fe	28.7445934	37.3677692	41.4712824
O	31.8516866	39.8229959	41.8658036
O	27.7839333	38.7694950	42.2183501
C	33.3014197	39.2163907	31.1310682
C	29.0903233	40.6827214	35.7316721
C	27.9267812	40.7396677	37.9440071
C	28.1203915	42.1962005	38.4266136
C	28.2244458	40.3433274	39.4419900
C	32.2780332	40.1347421	31.8906861
C	31.4106864	39.3492868	32.8801363
C	28.8691249	42.5721914	40.8632743
C	28.1627090	43.9767547	41.0969605
C	28.6232173	41.8543826	42.1656664
C	29.6459210	41.9285450	43.2499022

C	27.1990915	41.6935869	42.5860666
C	30.4229983	40.1488157	33.6912860
C	30.1569443	39.9370551	34.9904614
N	28.9060918	40.2435871	37.0050650
N	31.4735867	40.8087754	30.8212138
N	28.4121262	41.8018304	39.7036270
O	28.4318335	41.6014525	35.2367439
O	28.0264270	43.2825757	37.9038123
O	34.1115714	38.5822731	31.8291571
O	33.1790403	39.2266250	29.8829902
O	26.9885893	44.1488135	40.7198777
O	28.8356920	44.7584452	41.8143013
S	29.5964528	39.1391501	39.7288101
H	26.8954544	40.5215247	37.6142524
H	31.1187938	38.3946579	42.3066739
H	27.3439812	39.9453064	39.9633230
H	32.8480961	40.9203628	32.4091023
H	32.0907696	38.7777906	33.5294954
H	30.8495141	38.5939363	32.2948261
H	29.9459104	42.7824581	40.7502882
H	28.2405261	39.6257535	42.1757865
H	29.8338647	40.9223712	33.1813087
H	30.7020227	39.1577564	35.5350140
H	29.4963644	39.4965238	37.3774309
H	31.2534987	39.9133595	41.0875900
H	32.0545698	40.6209892	29.9622147
H	31.2865892	41.8127242	31.0010994
H	30.5611036	40.3534588	30.6258709
H	29.6907791	40.9931383	43.8361010
H	30.6430251	42.1766062	42.8565338
H	29.3907225	42.7228596	43.9761975
H	26.5538424	41.5308748	41.7122609
H	26.8503540	42.6177523	43.0915454
H	27.0574558	40.8604521	43.2871326
O	30.5189442	37.6487852	42.5865580
H	31.4750856	40.4289675	42.5213898
H	31.0295597	36.7884077	42.4945793
H	24.1151273	33.3100129	40.4218821
H	30.6563752	33.1898519	39.6896807
H	24.9008548	35.2927186	45.8791858

TS-V

C	24.4255003	33.8669981	39.6060782
C	25.2261198	35.0641623	40.0135586
N	24.6521510	36.2116502	40.5316697
C	26.5812846	35.3159272	40.0064614
C	25.6321801	37.0819254	40.8297589
N	26.8207069	36.5744251	40.5193390
H	23.5009339	34.1863645	39.1006611
H	25.0014429	33.2711589	38.8826440
H	27.3943465	34.6754076	39.6772657
H	25.4898216	38.0450697	41.3113083
H	23.6501767	36.3854933	40.6792451
C	31.2991891	33.8959668	40.1710050
C	30.7309287	35.0783301	40.9684660
O	29.7093845	35.7033216	40.5503052
O	31.4179117	35.4011623	41.9786237
H	31.9514317	33.2981375	40.8233361
H	31.9499935	34.3377230	39.3936322
C	25.8840773	35.6192736	46.1209205
C	26.8380793	35.5992510	44.9697042
N	27.4693919	34.4737422	44.4687288
C	27.2410829	36.6251828	44.1511269
C	28.1873798	34.8390046	43.3854556
N	28.0703936	36.1376747	43.1657585
H	26.2286949	34.9807232	46.9512682
H	25.8133033	36.6437438	46.5150254
H	26.9687508	37.6761006	44.1694395
H	28.7721412	34.1373464	42.7964276
H	27.3751420	33.5097742	44.8025780
Fe	28.5712954	37.4726574	41.4533286
O	32.0624708	39.6050665	41.6252591
O	27.3830270	38.6999476	42.2290209
C	33.2850654	39.1920461	31.1902560
C	28.9205045	40.3928346	35.7248881
C	27.7300255	40.6595827	37.8716874
C	27.8961395	42.1719900	38.1737462
C	27.9351325	40.4785015	39.4261238
C	32.2364034	40.0616571	31.9740151
C	31.4081334	39.2191054	32.9524894
C	28.7323480	42.7614929	40.5164949
C	27.9620481	44.0491152	40.9991057
C	29.0472688	41.9273212	41.7322530
C	30.3727357	42.1640836	42.3729911
C	27.9286938	41.6720529	42.7008601
C	30.3515301	39.9400779	33.7487057
C	30.0764585	39.7246544	35.0466981
N	28.7788289	40.0990095	37.0506320
N	31.4064288	40.7312354	30.9211993
N	28.0586881	41.9498580	39.5083617
O	28.1421790	41.1451696	35.1418477
O	27.9311403	43.1877151	37.5129974
O	34.1148115	38.5657063	31.8720468
O	33.1566084	39.2232742	29.9430998
O	26.7490825	44.1731695	40.7436964
O	28.6575833	44.8307486	41.6969631
S	29.3724270	39.5574064	40.0725379
H	26.7293355	40.3769327	37.5041598
H	31.0508264	38.3618360	42.1422862
H	27.0443014	40.0887476	39.9392410
H	32.7804107	40.8557344	32.5073647
H	32.1160806	38.6896973	33.6074655
H	30.9063218	38.4298454	32.3579046
H	29.6785302	43.1244826	40.0822513
H	27.7901456	39.5670434	42.3322952
H	29.6989823	40.6496184	33.2222226
H	30.6742582	39.0116663	35.6254601
H	29.4570214	39.5148777	37.5388373
H	31.4458667	39.9493003	40.9480030

H	31.9894435	40.5767786	30.0565176
H	31.1935568	41.7274701	31.1163126
H	30.5072133	40.2556841	30.7131477
H	30.5819847	41.3956566	43.1335397
H	31.1872576	42.2221114	41.6349418
H	30.3582140	43.1375136	42.8994685
H	27.0066623	41.3327537	42.2098742
H	27.6755217	42.6036819	43.2480459
H	28.2313092	40.9435629	43.4673978
O	30.3849626	37.7131504	42.4937596
H	31.9670257	40.2290868	42.3588890
H	30.8366860	36.8101092	42.4612297
H	24.0943793	33.2337079	40.4291028
H	30.5962233	33.2129533	39.6941414
H	24.8828383	35.2582238	45.8859084

PD

C	24.2740137	33.6509063	39.6060102
C	25.1476795	34.8146848	39.9480107
N	24.6816020	36.0286052	40.4172835
C	26.5197408	34.9375938	39.9412833
C	25.7528437	36.8129123	40.6824518
N	26.8820602	36.1785742	40.4036603
H	23.3352866	33.9898138	39.1425877
H	24.7903427	33.0175959	38.8690505
H	27.2620536	34.2040125	39.6392712
H	25.7110728	37.8190543	41.0940184
H	23.6990714	36.2897627	40.5646932
C	31.4478023	33.4625948	40.1355343
C	30.9100319	34.6580259	40.9195466
O	29.9006307	35.2930095	40.4829122
O	31.5882774	34.9790582	41.9320032
H	32.0596167	32.8302547	40.7934240
H	32.1334959	33.8756549	39.3727576
C	25.8049134	35.6143022	46.1504211
C	26.7781611	35.5092787	45.0204827
N	27.4303647	34.3426068	44.6740788
C	27.1984908	36.4357683	44.0920463
C	28.1779709	34.5819359	43.5755539
N	28.0612625	35.8406197	43.2021239
H	26.1399187	35.0327707	47.0252817
H	25.7221426	36.6610447	46.4781295
H	26.9231744	37.4832536	43.9861553
H	28.7856883	33.8223880	43.0887971
H	27.3184717	33.4139598	45.0888889
Fe	28.7091207	36.8387597	41.4265755
O	31.6769699	39.7140161	42.1753445
O	27.9571043	38.5969023	41.5759387
C	33.3330440	39.2424291	31.0740951
C	28.8614679	40.2488683	35.4811897
C	27.8459454	40.8615424	37.6340641
C	27.7286897	42.4002397	37.7503961
C	28.0647232	40.9194537	39.1732984
C	32.2518074	40.0598681	31.8621186
C	31.4368716	39.1435141	32.7844429
C	28.5435073	43.2638429	40.0163432
C	27.7760932	44.3646027	40.8024737
C	29.3443623	42.3065718	40.9532525
C	30.6553610	42.8752255	41.4795813
C	28.4977184	41.8046947	42.1338289
C	30.2714894	39.7620132	33.5012564
C	30.1167565	39.7780571	34.8346489
N	28.9464238	40.3905379	36.8369176
N	31.4162804	40.7536393	30.8311682
N	27.7618642	42.3745388	39.1294210
O	27.8101800	40.4683339	34.8781772
O	27.7319194	43.3052443	36.9454644
O	34.1661566	38.6231738	31.7608326
O	33.2143488	39.2894208	29.8293696
O	26.5451585	44.4777989	40.6468846
O	28.5011083	45.0761498	41.5438127
S	29.7814016	40.8704961	39.8080707
H	26.9119524	40.3683779	37.3239464
H	31.1732081	37.9331306	42.1886925
H	27.4308311	40.2836493	39.8076865
H	32.7578014	40.8436307	32.4451429
H	32.1484321	38.6905120	33.4898119
H	31.0423311	38.3114987	32.1693285
H	29.2627128	43.8040099	39.3799813
H	28.3605666	39.1313621	42.2701480
H	29.4454361	40.1626468	32.8990662
H	30.9038228	39.3784821	35.4836546
H	29.8644724	40.3985406	37.2719817
H	30.8777427	39.8436850	41.6331236
H	32.0068160	40.6842867	29.9662764
H	31.1539689	41.7240980	31.0867764
H	30.5394363	40.2541814	30.5841301
H	31.3112697	42.0514122	41.7909124
H	31.1696483	43.4899614	40.7315617
H	30.4526157	43.4876321	42.3646022
H	27.5955075	41.2594247	41.8272335
H	28.1997977	42.6691557	42.7477586
H	29.1016122	41.1425004	42.7721973
O	30.5693722	37.2693160	42.5808777
H	31.3231372	39.6649950	43.1038302
H	31.0360623	36.3914483	42.4936562
H	23.9793295	33.0515858	40.4674295
H	30.7000889	32.8355424	39.6499449
H	24.8126072	35.2281050	45.9175110

Supplementary References

1 Rabe, P. *et al.* X-ray free-electron laser studies reveal correlated motion during isopenicillin N synthase catalysis. *Sci Adv* **7** (2021). https://doi.org/10.1126/sciadv.abh0250

2 Aguilar, J. A., Nilsson, M., Bodenhausen, G. & Morris, G. A. Spin echo NMR spectra without J modulation. *Chemical Communications* **48**, 811–813 (2012). https://doi.org/10.1039/C1CC16699A

3 Rabe, P. *et al.* Spectroscopic studies reveal details of substrate-induced conformational changes distant from the active site in isopenicillin N synthase. *J Biol Chem* **298**, 102249 (2022). https://doi.org/10.1016/j.jbc.2022.102249

4 Fuller, F. D. *et al.* Drop-on-demand sample delivery for studying biocatalysts in action at X-ray free-electron lasers. *Nat Methods* **14**, 443–449 (2017). https://doi.org/10.1038/nmeth.4195

5 Owen, R. L. *et al.* Low-dose fixed-target serial synchrotron crystallography. *Acta Crystallogr D Struct Biol* **73**, 373–378 (2017). https://doi.org/10.1107/S2059798317002996

6 Rabe, P. *et al.* Anaerobic fixed-target serial crystallography. *IUCrJ* **7**, 901–912 (2020). https://doi.org/10.1107/S2052252520010374

7 Baldwin, J. E. & Wan, T. S. Penicillin biosynthesis: Retention of configuration at c-3 of valine during its incorporation into the arnstein tripeptide. *Tetrahedron* **37**, 1589–1595 (1981). https://doi.org/https://doi.org/10.1016/S0040-4020(01)92100-3

8 Subirós-Funosas, R., Prohens, R., Barbas, R., El-Faham, A. & Albericio, F. Oxyma: An Efficient Additive for Peptide Synthesis to Replace the Benzotriazole-Based HOBt and HOAt with a Lower Risk of Explosion[1]. *Chemistry – A European Journal* **15**, 9394–9403 (2009). https://doi.org/https://doi.org/10.1002/chem.200900614

9 Arakawa, K., Cao, Z., Suzuki, N. & Kinashi, H. Isolation, structural elucidation, and biosynthesis of 15-norlankamycin derivatives produced by a type-II thioesterase disruptant of Streptomyces rochei. *Tetrahedron* **67**, 5199–5205 (2011). https://doi.org/https://doi.org/10.1016/j.tet.2011.05.047

10 Cooper, R. D. G. & Jose, F. L. Structural studies on penicillin derivatives. IV. Novel rearrangement of penicillin V sulfoxide. *Journal of the American Chemical Society* **92**, 2575–2576 (1970). https://doi.org/10.1021/ja00711a075

11 Abraham, E. P. *et al.* Monocyclic β-lactam tripeptide, 1-(D-carboxy-2-methylpropyl)-3-L-(δ-L-2-aminoadipamido)-4-L-mercaptoazetidin-2-one, a putative intermediate in penicillin biosynthesis. *Journal of the Chemical Society, Chemical Communications*, 1130–1132 (1982). https://doi.org/10.1039/C39820001130

12 Baldwin, J. E., Bradley, M., Adlington, R. M., Norris, W. J. & Turner, N. J. Further evidence for the involvement of a monocyclic β-lactam in the enzymatic conversion of δ-L-α-aminoadipoyl-L-cysteinyl-D-valine into isopenicillin N. *Tetrahedron* **47**, 457–480 (1991). https://doi.org/https://doi.org/10.1016/S0040-4020(01)90503-4
