# Supplementary material for: Unanticipated intermediates during isopenicillin N synthase catalysis identified by time-resolved X-ray free-electron laser studies
Source: Nat Catal. 2026 Oct 9;9(10):1141–57. doi: 10.1038/s41929-026-01618-4 (PMC13690604; doi:10.1038/s41929-026-01618-4)
Supplement: Supplementary file 2 [file 41929_2026_1618_MOESM2_ESM.pdf]

nature portfolio

Corresponding author(s): Dr. Patrick Rabe, Dr. Allen Orville, Dr. Jan Kern, Prof. Dr. Christopher Schofield,

Last updated by author(s): Jun 29, 2026



# Reporting Summary

Nature Portfolio wishes to improve the reproducibility of the work that we publish. This form provides structure for consistency and transparency in reporting. For further information on Nature Portfolio policies, see our Editorial Policies and the Editorial Policy Checklist.

## Statistics

For all statistical analyses, confirm that the following items are present in the figure legend, table legend, main text, or Methods section.

| n/a | Confirmed | |
|---|---|---|
| ☐ | ☒ | The exact sample size ($n$) for each experimental group/condition, given as a discrete number and unit of measurement |
| ☐ | ☒ | A statement on whether measurements were taken from distinct samples or whether the same sample was measured repeatedly |
| ☐ | ☒ | The statistical test(s) used AND whether they are one- or two-sided<br>*Only common tests should be described solely by name; describe more complex techniques in the Methods section.* |
| ☐ | ☒ | A description of all covariates tested |
| ☒ | ☐ | A description of any assumptions or corrections, such as tests of normality and adjustment for multiple comparisons |
| ☒ | ☐ | A full description of the statistical parameters including central tendency (e.g. means) or other basic estimates (e.g. regression coefficient) AND variation (e.g. standard deviation) or associated estimates of uncertainty (e.g. confidence intervals) |
| ☒ | ☐ | For null hypothesis testing, the test statistic (e.g. $F$, $t$, $r$) with confidence intervals, effect sizes, degrees of freedom and $P$ value noted<br>*Give P values as exact values whenever suitable.* |
| ☒ | ☐ | For Bayesian analysis, information on the choice of priors and Markov chain Monte Carlo settings |
| ☒ | ☐ | For hierarchical and complex designs, identification of the appropriate level for tests and full reporting of outcomes |
| ☒ | ☐ | Estimates of effect sizes (e.g. Cohen's $d$, Pearson's $r$), indicating how they were calculated |

*Our web collection on statistics for biologists contains articles on many of the points above.*

## Software and code

Policy information about availability of computer code

| | |
|---|---|
| Data collection | DIALS ver. 3.24.3, cctbx.xfel, TopSpin 4.4.1, MassLynx 4.2, Amber18, ChemShell ver 3.7.0., Turbomole 7.2.1., DL_POLY as implemented in ChemShell 3.7.0. |
| Data analysis | WinCOOT ver. 0.9.8.7, PHENIX ver 1.21.1.5286, MassLynx 4.2, GraphPad Prism ver. 10.2, MestReNova 17.0.0, PyMOL ver 3.1.8, OriginPro 2026, RapidFire Integrator 4.3.0 , MassHunter Workstation B.08.00, CPPTRAJ V6.24.0, VMD ver 1.9.3, Open source PyMOL ver 3.1.0. |

For manuscripts utilizing custom algorithms or software that are central to the research but not yet described in published literature, software must be made available to editors and reviewers. We strongly encourage code deposition in a community repository (e.g. GitHub). See the Nature Portfolio guidelines for submitting code & software for further information.

## Data

Policy information about availability of data

All manuscripts must include a data availability statement. This statement should provide the following information, where applicable:

- Accession codes, unique identifiers, or web links for publicly available datasets
- A description of any restrictions on data availability
- For clinical datasets or third party data, please ensure that the statement adheres to our policy

The atomic coordinates and structure factors are deposited in the PDB with the following accession codes: 8PY6 (IPNS:Fe:ACdV, anaerobic, SFX), 8PY7 (IPNS:Fe:ACdV:O2, 1 s O2, SFX), 8PY8 (IPNS:Fe:ACdV:O2, 2 s O2, SFX), 8PY9 (IPNS:Fe:ACdV/TAL:O2, 3 s O2, SFX), 8PYA (IPNS:Fe:ACdV/TAL:O2, 4 s O2, SFX), 9I3A

(IPNS:Fe:TAL:O2, 6 s O2, SFX), 9I3B (IPNS:Fe/Fe-O:TAL/MCI, 8 s O2, SFX), 9I3C (IPNS:Fe/Fe-O:TAL/MCI, 10s O2, SFX), 9QPH (IPNS:Fe:IPN (from ACdV), 24h O2, SFX), 8PY5 (IPNS:Fe:ACdV, SFX), 9I3D (IPNS:Fe/Fe O: MCI/IPN, 10s O2, SFX), 6ZAK (IPNS:VO:ACV, SFX), 9QPI (IPNS:Fe:ACdV, anaerobic, SFX), 9QPJ (IPNS:Fe:IPN (from AcdV), 24h O2, SSX), 8P48 (IPNS_N252D:Fe:ACV, MX), 8P46 (IPNS_N252D:Fe:IPN, 30 min O2, MX), 8P47 (IPNS_N252A:Fe:ACV, MX), 8PTV (IPNS_N252Q:Fe:ACV, MX), 8RDB (IPNS_N252E:Fe:ACV, MX), 9HWF (IPNS_N252E:Fe:IPN, 30 min O2, MX), 7ZOE (IPNS_H270E:Fe:ACV, MX), 8A4G (IPNS_H270E:Cd:ACV, MX), 8ALI (IPNS_H270E:Fe:ACV, 30 min O2, MX), 8ALJ (IPNS_H270E:Fe:ACV, 3 h O2, MX), 9QPG (IPNS:Fe:IPN (from AcdV), 18h O2, MX). Coordinates for the molecular dynamics and QM/MM calculations are provided in the Supplementary Information. Raw data for the XES, UV/Vis and mass spectrometry experiments are provided in the Source Data file. All other data supporting the findings of this study are available within the paper and Supplementary Information.

## Research involving human participants, their data, or biological material

Policy information about studies with human participants or human data. See also policy information about sex, gender (identity/presentation), and sexual orientation and race, ethnicity and racism.

| | |
|---|---|
| Reporting on sex and gender | NA |
| Reporting on race, ethnicity, or other socially relevant groupings | NA |
| Population characteristics | NA |
| Recruitment | NA |
| Ethics oversight | NA |

Note that full information on the approval of the study protocol must also be provided in the manuscript.

# Field-specific reporting

Please select the one below that is the best fit for your research. If you are not sure, read the appropriate sections before making your selection.

☒ Life sciences ☐ Behavioural & social sciences ☐ Ecological, evolutionary & environmental sciences

For a reference copy of the document with all sections, see nature.com/documents/nr-reporting-summary-flat.pdf

# Life sciences study design

All studies must disclose on these points even when the disclosure is negative.

| | |
|---|---|
| Sample size | For crystallographic data, all structures except the time-resolved experiments were collected in at least duplicate; mass spectrometry assays were performed in biological triplicates. |
| Data exclusions | Obvious outliers (<5%) in mass spectrometry turnover measurements were excluded. For serial crystallography and X-ray emission spectroscopy data, a very small fraction of outlier reflections (typically 1–2 per >10,000 images) were removed during standard processing. |
| Replication | Crystallographic datasets (except time-resolved data) were reproduced independently; mass spectrometry assays were performed in triplicate with consistent results. |
| Randomization | Randomization was not applicable to this study, as the experiments involve purified proteins and crystallographic assays. |
| Blinding | Blinding was not applicable, as data collection and analysis relied on automated crystallographic and spectrometric pipelines. |

# Reporting for specific materials, systems and methods

We require information from authors about some types of materials, experimental systems and methods used in many studies. Here, indicate whether each material, system or method listed is relevant to your study. If you are not sure if a list item applies to your research, read the appropriate section before selecting a response.

## Materials & experimental systems

| n/a | Involved in the study |
|---|---|
| ☒ | ☐ Antibodies |
| ☒ | ☐ Eukaryotic cell lines |
| ☒ | ☐ Palaeontology and archaeology |
| ☒ | ☐ Animals and other organisms |
| ☒ | ☐ Clinical data |
| ☒ | ☐ Dual use research of concern |
| ☒ | ☐ Plants |

## Methods

| n/a | Involved in the study |
|---|---|
| ☒ | ☐ ChIP-seq |
| ☒ | ☐ Flow cytometry |
| ☒ | ☐ MRI-based neuroimaging |

# Plants

| | |
|---|---|
| Seed stocks | NA |
| Novel plant genotypes | NA |
| Authentication | NA |
