# Supplementary material for: Unanticipated intermediates during isopenicillin N synthase catalysis identified by time-resolved X-ray free-electron laser studies
Source: Nat Catal. 2026 Oct 9;9(10):1141–57. doi: 10.1038/s41929-026-01618-4 (PMC13690604; doi:10.1038/s41929-026-01618-4)
Supplement: Supplementary file 3 [file 41929_2026_1618_MOESM3_ESM.pdf]

nature portfolio

Peer Review File

Unanticipated Intermediates During Isopenicillin N Synthase Catalysis Identified by Time-Resolved X-ray Free Electron Laser Studies

Corresponding Author: Professor Christopher Schofield

This manuscript has been previously reviewed at another journal. This document only contains information relating to versions considered at Nature Catalysis.

Version 0:

Reviewer comments:

Reviewer #1

(Remarks to the Author)
The authors use XES to characterize the oxidation state of Fe. It is well established that the Kbeta1,3 first moment or integrated absolute difference (IAD) as well as the Fe Kalpha1 full width at half maximum (FWHM) can be linked to the electron spin density on Fe and thus the formal oxidation state. I am surprised to see that the authors analyzed instead the Kalpha1 first moment. While it is known that the Kalpha1 first moment shows a chemical dependence, there is to this reviewer's knowledge no established mechanism that links the Fe Kalpha1 first moment energy to the oxidation state of Fe.

So at this point the authors may observe changes in the Fe electronic structure but it is unclear how to interpret the changes. It is not possible to assign oxidation states using the Fe Kalpha1 first moment. Consequently, this reviewer rejects the XES analysis and interpretation. In case the authors reanalyze their data and use the Fe Kalpha1 FWHM, they should keep on mind that any assignment of oxidation state must be based on a set of suitable reference compounds.

The authors show error bars for the Fe Kalpha1 first moment below 0.01 eV. The error bar is deduced from the counting statistics. It would be important to discuss systematic errors as well, such as stability of the XES energy calibration. Ideally, the authors should show repeated measurements over time of the same compound to provide an estimate of the stability of the XES energy calibration.

Finally, it would be important to show all spectra without smoothing in the extended data.

Reviewer #2

(Remarks to the Author)
The IPNS enzyme whose biosynthetic reaction oxidizes the peptide ACV to the bicyclic penicillin backbone provides the substrate from which many clinical antibiotics are derived. Understanding the catalytic formation of the two rings of the penicillin backbone has been elusive. This superb manuscript provides convincing evidence that this involves a ferryl iron with a key role for an active site water. Surprisingly the thioaldehyde intermediate observed does not coordinate the iron. The manuscript contains 25 crystal structures (13 of which are determined by time-resolved serial crystallography supported by trXES). Mutagenesis, mass spectrometry, organic synthesis, NMR and molecular dynamics validate hypotheses that arise from the crystal structures. The data are excellent and every conceivable control has been carried out. This is a very complex manuscript, but the authors do a first-rate job of walking the reader through the data and what these support in terms of mechanism. In particular, the figures provided were comprehensive, and they convincingly supported the manuscript’s conclusions. This is an important manuscript on a medically important enzyme. All my specific comments below are minor being typographical or simple fixes.

Ln 74: “studied reaction” should be “studied the reaction”

Lns 107 and 110: Fig 1 is not split into panels (a) and (b), so presumably these labels should be “Fig.1 path a” and Fig. 1 path b”

Lns 203 and 715: “anaerobicic” should be “anaerobic”

Ln 224: “ib” should be “at”?

Ln 245: There is no extended fig. 2 “2div” panel. I think the authors mean “2aiv”?

Ln 285: There is no extended data fig 2ciii. However, Fig. 2biii in the body of the paper shows increased Fobs difference density for the ring sulfur after 10s compared to the 8s structure. This supports partial formation of the IPN in PDB 913D. Do they mean this figure?

Ln 291: What is shunt product (1)? The hydrated aldehyde of extended fig. 5o?

Ln 293-5: Extended Fig. 3(o,p) should be associated with this sentence.

Ln 296: “Fig2c” should be “Fig. 2c”

Ln 318: None of the ribbon figures in fig. 2 and extended data fig. 1 show where the active site is located in reference to alpha10, so one cannot envision the relationship between the two. Are there direct lattice packing interactions involving helix 10?

Ln 325: Sentence ends with a comma.

Ln 353: “susbtitued” should be “substituted”.

Ln 390: “timpoints” should be “timepoints”

Ln 411: Point to panel a of extended data fig. 6 rather than the whole of the figure. “Extended Data Fig. 6a”.

Ln 423: I believe “Fig. 4j” should be “Fig. 3j”

Ln 425: I believe “Fig3j” should be “Fig. 3j”

Ln 427: I believe “Fig. 4h” should be Fig. 3h”

Ln 435: “Fig. 2” should reference the specific panel a, i.e. “Fig. 2a”

Ln 441: “(Fig. 3g)” should be “(Fig. 3g))”

Ln 450: I believe “Extended Data Fig. 2.8fi” should be “Extended Data Figs. 2c-g, 8fi”

Ln453: I believe “Extended Data Fig. 2,S8a-c” should be “Extended Data Figs. 2c-g, 8a-c”

Ln 488: “interactions.” Should be “interactions).”

Ln 600: “os” should be “of”

Ln 660: Missing space between “O2” and “with”

Ln 670: The “2” of “10s O2” should be subscript

Ln 767: “occupanc” should be “occupancy”

Ln 831: “The +11 charge state was shown” should be “The +11 charge state is shown”

Fig. 2bi: The label box around “Sulfur release from Fe” needs to be extended to cover all the text.
Extended Fig. 7dii. The top trace is labelled as showing “HPLC purified 2 and 3” but the trace only contains 3 and 5.

SUPPORTING INFORMATION

Lns 151, 155, 160: “Carry” should be “Cary”

Ln 157: “anerobic” should be “anaerobic”

Ln 347-8: “Assay samples were aspirated under vacuum for 0.6 s and loaded onto a” should be removed as the complete sentence starts the next paragraph that immediately follows.

Reviewer #3

(Remarks to the Author)
Isopenicillin N synthase (IPNS) plays a pivotal role in the biosynthesis of penicillin and cephalosporin antibiotics, making it a subject of significant interest in biochemical research. The enzyme's unique catalytic mechanism, which involves the formation and transformation of high-energy intermediates, has been challenging to characterize due to the fleeting nature of these transient states. Previous studies have contributed valuable information regarding the structure and function of IPNS; however, important gaps remained, particularly in the detailed understanding of the intermediates that arise during catalysis. To address these gaps, the authors emphasize the necessity of advanced experimental methods, such as time-resolved crystallography, which can capture the rapid and subtle changes that occur within the enzyme during its reaction cycle. This is a valuable approach to tackle the problem to understand the enzymatic process of IPNS. It would be appreciated if the authors describe what practical implications that may have to the development of new antibiotics in this field and /or the improvement of current synthesis.
Experimental Approach
To investigate the catalytic mechanism of IPNS, the authors utilized a combination of time-resolved serial femtosecond crystallography and X-ray emission spectroscopy (XES). These methods enabled the observation of both structural transformations and changes in the redox state of iron within the enzyme as the reaction progressed. The experimental setup involved reacting anaerobic IPNS·Fe(II)·ACV microcrystals with molecular oxygen and monitoring the ensuing changes at various time intervals. In addition, the use of deuterium-labeled substrates allowed for enhanced resolution in distinguishing subtle mechanistic features. Mutagenesis and computational studies were also performed to support and validate the proposed mechanistic pathways.
Results
The integrity and effectiveness of the sample delivery system were confirmed through initial experiments, which successfully initiated the reaction by exposing microcrystals to oxygen. Structural analyses revealed the sequential formation of key intermediates within the catalytic cycle. Notably, the authors observed the emergence of an Fe(III)-superoxo intermediate, followed by the displacement of the ACV-derived sulfur atom, which led to the formation of a thioaldehyde intermediate that does not coordinate with iron. Electron density and isomorphous difference maps provided detailed visualizations of these events. The data demonstrated that the reaction proceeds through several distinct intermediates, including a crucial monocyclic intermediate (MCI) essential for constructing the β-lactam ring.
As the reaction advanced, the formation of the MCI and the subsequent restoration of the Fe-S bond were documented, supported by comparisons with known substrate analogs. The results highlighted the significant role of a water chain present in the enzyme’s active site, which serves to stabilize the intermediates and facilitate the overall reaction process. The findings also provided insights with broader implications for the family of 2-oxoglutarate-dependent oxygenases.
Critique:
Figure 2:
The 24-hour time point needs to be explained a little bit more in detail, as the maintenance of stable crystal conditions can be difficult for such a long time compared to seconds.
Figure 2c, the colors for the different time points should be more varied to better differentiate.
In Fig 2 b: For the isomorphous difference maps: Are they all on the same level in i, ii, iii?
The sulfur atoms are not in the center of the green(positive) difference in ii, iii any explanation, iv is even more difficult to understand.

Mutagenesis experiments:
Second shell mutagenesis of N252 to A, E and Q shows considerable influence on the reaction kinetic which in the found structures show the importance of several water molecules.
First shell mutations like H270E revealed the involvement of the Fe bound water in peroxide cleavage and thioaldehyde formation.
These experimental results are further analyzed with computational modeling to achieve a complete reaction coordinate explanation in detail. It would be helpful in the discussion if the authors clearly mentioned where specific experimental data are missing just to state that not all points e.g., can be measured.

In general: the presented pdb structures should be clearly assigned to certain beamlines (LCLS, PAL XFEL, SACLA and DLS), not only in the supplement table. In this very data heavy paper, the tr-sfx and XES is clearly pronounced. The data coming from the diamond beam line (DLS)are not specifically mentioned (“by traditional, rotation-based methods under cryogenic conditions,” this for non-crystallographers impossible to decipher) or compared with the SFX data. The comparison between tr-sfx and tr-synchrotron data would be of interest as some structures in the paper have only been determined by standard cryogenic methods at DLS (why not at RT).
The data table in the supplement raises the question for the 24 h experiment row 9 and 14, which data are presented the DLS and or LCLS and what was the difference if there was any for the claim of the B-factor change.
Discussion
In summary, the study elucidates the dynamic nature of IPNS catalysis, revealing the importance of both protein conformational flexibility and the participation of water molecules in the reaction mechanism. The authors acknowledge the inherent limitations in resolving every intermediate due to their transient nature, and they recognize the need for further investigation to fully map the entire catalytic pathway. Future research is recommended to focus on the roles of specific amino acid residues and the dynamics of solvent molecules, aiming to further deepen the understanding of enzyme catalysis at a molecular level.
This is a plethora of data to give detailed insight into a reaction coordinate of an important catalytic process. The presentation should follow an overview detail scheme for the results and discussion part.
Suggestions for improvement. The motivation for tr-SFX and XES should be better explained and with tr SSX compared. In the discussion the model described for the reaction coordinate should be more critically discussed in a way the highlights

and shortcomings are expressed.
Overall: I recommend the paper with minor revisions.

Reviewer #4

(Remarks to the Author)
Most 2OG-dependent oxygenases catalyse two-electron hydroxylation reactions coupled to the oxidative decarboxylation of 2OG to succinate. In contrast, IPNS uniquely catalyses a spatially orchestrated four-electron oxidation that converts the tripeptide ACV into the highly strained penicillin scaffold without the involvement of 2OG. Although IPNS has been extensively studied, understanding how precise atomic positioning and enzyme dynamics enable catalysis involving high-energy intermediates remains a central challenge in structural biology. In particular, the structures of the proposed Fe(II)–hydroperoxide thioaldehyde and Fe(IV)=O monocyclic intermediates implicated in the key ring-forming steps have thus far remained elusive.
In this manuscript, the authors harness the power of tr-SFX in combination with tr-XES to probe transient intermediates during IPNS catalysis. Using anaerobic IPNS·Fe(II)·ACV microcrystals as the starting point, the reaction was initiated by controlled exposure to O_2 and followed using XFEL-based structural and spectroscopic approaches. The resulting time-course structures reveal that cysteinyl oxidation of ACV generates a thioaldehyde intermediate that, unexpectedly, does not coordinate the iron center. Subsequent experiments employing isotopically labelled ACV demonstrate the formation of a monocyclic β-lactam intermediate accompanied by restoration of the Fe–S bond. When combined with mutagenesis, spectroscopic, and computational analyses, the XFEL results support a mechanism in which oxidation of the ACV valine residue in the monocyclic intermediate is mediated by an Fe(IV) species. Moreover, a hydrogen-bonding network involving three water molecules appears to play a crucial role in stabilising the intermediates, particularly by facilitating Fe(IV) coordination of the monocyclic intermediate prior to isopenicillin N formation.
Overall, this work is beautifully executed and thoughtfully designed. It compellingly highlights the power of time-resolved XFEL approaches for characterising transient intermediates in oxygenase catalysis. This work offers valuable insights into the significant roles of water molecules in enzymatic reaction mechanisms that are challenging to dissect. Nonetheless, a few points merit further consideration, as outlined below.
1. In the computational section, the authors state that proton transfer from IM-II via water molecule W4 to yield the IPNS:Fe(IV)=O:MCI complex (IM-IV) was energetically unfavourable, instead identifying an intermediate IM-III in which reorientation of the ACV valine methyl groups and W4 enables formation of the monocyclic intermediate (IM-IV) by overcoming an activation barrier of 26.7 kcal mol^{-1}. As these mechanistic conclusions rely heavily on quantum mechanical calculations, the authors are requested to provide the atomic coordinates of the QM region used in the calculations to ensure reproducibility and independent evaluation. In addition, the authors should clarify why the reorientation of the ACV valine methyl groups and W4 was not captured in the tr-SFX analysis.
2. The authors employ substrate or cofactor analogues, such as vanadium in place of iron, in the crystallographic studies to elucidate the structural basis of catalysis. To further support the relevance of these analogues to the native reaction, it would be helpful to provide corresponding biochemical data demonstrating that the analogue faithfully reflects the behaviour of the native metal cofactor. In addition, in the HPLC profile shown in Extended Data Fig. 7, the identities of the peaks at retention times of 3.4 min and 4.2 min should be clarified.
3. Although the difference spectra obtained from Fe $K\alpha_{1,2}$ XES analysis suggest changes in the iron electronic structure, these features appear relatively subtle. It would therefore be helpful if the authors could provide complementary experimental evidence, such as Mössbauer spectroscopy, to further substantiate the proposed electronic and/or spin-state assignments.

Reviewer #5

(Remarks to the Author)
In this work a detailed study is presented on isopenicillin N synthase, which is an unusual nonheme iron dioxygenase where dioxygen triggers four successive hydrogen atom abstractions (formally) resulting in two ring-closure reactions in the substrate. There are still details missing on the mechanism and multiple suggestions have been proposed for this. A large team from various countries report here crystallography, spectroscopy and computational studies. Particularly the trapping of these short-lived intermediates is impressive and gives insight into this important reaction and elucidates details of the mechanism. I am not an expert in crystallography so cannot argue about the methods, procedures etc, so will focus on the computation and general topic mostly. Overall, the paper fits the remit of Nature Catalysis well, but some changes and maybe additional work will benefit the overall discussion.

1. If water is essential for catalysis, there should be a solvent isotope effect when D2O is used. Has the experiment with D2O been attempted?
2. Did the authors use deuterated substrate to establish rates and kinetic isotope effects?
3. Computation: If water molecules are important for the mechanism, why were these not included in the QM region?
4. I am surprised the authors did not use dispersion in the QM/MM calculations.
5. A QM/MM basis set of def2-SVP may be to modest to get good geometries.
6. There are no MD and QM/MM data in the SI. Please add. I also would include Cartesian coordinates of the QM region

structures of all intermediates and transition states.
7. I do not see the activation barrier of 27.2 kcal/mol mentioned in the text in Figure 4. Also, a barrier of 27.2 kcal/mol as well as the energy gap between TS-IV and IM-III of 33.4 kcal/mol seems a bit high for barriers for an enzymatic process. These barriers may be too slow for an enzyme that works at room temperature. How do these barriers compare with experiment?
8. The authors may want to compare the optimized geometries with calculations on other thiol dioxygenases (cysteine dioxygenase, ovothiol biosynthesis enzyme etc).
8. Intro line 2, typo in penicillin.

Version 1:

Reviewer comments:

Reviewer #2

(Remarks to the Author)
Addressing concerns of reviewer 1: The authors have added XES Kalpha raw data, which confirms the validity of the smoothed curves. I also found the difference spectra in Extended Fig. 4d supported the significant change in the iron electronics suggested by the tr-SFX and mechanistic studies. They also added IAD (panel g) and HWHM data for the Kalpha1 and 2 regions (panels h-j) along with reference model compound data (panel k). Although formal oxidation state cannot be assigned from the model compounds the trend is clear and changes in the tr-XES Kalpha first moment analysis and the proposed changes in oxidation states align with the model compounds. In the revised manuscript he authors emphasize that the trends in the XES data are consistent with the proposed changes in iron electronic state based on the tr-SFX and mechanistic analysis. I believe that the authors have successfully made the case for the tr-XES Kalpha data to be included. The analysis and interpretation in the revised manuscript adds substantive value and support and does not over interpret the XES.

Extended Figure 4f legend: “formation of stronger change in the electronic structure which is consistant with interpretation of the tr-SFX data as a Fe(IV)=O species”. I feel “stronger change” is not a good description of the suggested electronic change. Perhaps “significant change” or “pronounced change”? By the way, “consistent” is mis-spelt in this sentence.

The arguments the authors present in the rebuttal convince me that the stability of the XES energy calibration is such that errors due to instrument instability are not a concern and that errors are primarily a result of counting statistics.

Reviewer 2: The authors have addressed all my points to my satisfaction. The changes to Fig. 2a and Extended Data 1, plus the new SI Fig.2 have made the relationship between the active site and helix 10 very clear. SI Fig. 2 is also very helpful for visualizing the lattice contacts and the role played in slowing product release.

Carrie Wilmot (University of Minnesota)

Reviewer #3

(Remarks to the Author)

Reviewer #4

(Remarks to the Author)
The authors have provided detailed explanations and responses to the questions and concerns I raised. Meanwhile, the corresponding contents have been supplemented and modified in the revised draft. The current version can be considered for acceptance in the journal.

Reviewer #5

(Remarks to the Author)
All issues have been addressed well, but I spotted a typo in the conclusion section: "2-oxolutarate" -> 2-oxoglutarate.

Open Access This Peer Review File is licensed under a Creative Commons Attribution 4.0 International License, which permits use, sharing, adaptation, distribution and reproduction in any medium or format, as long as you give appropriate credit to the original author(s) and the source, provide a link to the Creative Commons license, and indicate if changes were made.
In cases where reviewers are anonymous, credit should be given to 'Anonymous Referee' and the source.
The images or other third party material in this Peer Review File are included in the article's Creative Commons license, unless indicated otherwise in a credit line to the material. If material is not included in the article's Creative Commons license and your intended use is not permitted by statutory regulation or exceeds the permitted use, you will need to obtain permission directly from the copyright holder.
To view a copy of this license, visit https://creativecommons.org/licenses/by/4.0/

POINT-TO-POINT RESPONSES TO THE REVIEWERS' COMMENTS:

We thank all the reviewers for their positive concerning our scientific results and very helpful comments to improve the manuscript, which we have addressed as described below.

Reviewer #1

Comment:

The authors use XES to characterize the oxidation state of Fe. It is well established that the Kbeta1,3 first moment or integrated absolute difference (IAD) as well as the Fe Kalpha1 full width at half maximum (FWHM) can be linked to the electron spin density on Fe and thus the formal oxidation state. I am surprised to see that the authors analyzed instead the Kalpha1 first moment. While it is known that the Kalpha1 first moment shows a chemical dependence, there is to this reviewer's knowledge no established mechanism that links the Fe Kalpha1 first moment energy to the oxidation state of Fe.
So at this point the authors may observe changes in the Fe electronic structure but it is unclear how to interpret the changes. It is not possible to assign oxidation states using the Fe Kalpha1 first moment. Consequently, this reviewer rejects the XES analysis and interpretation. In case the authors reanalyze their data and use the Fe Kalpha1 FWHM, they should keep on mind that any assignment of oxidation state must be based on a set of suitable reference compounds.

Response:

We thank the reviewer for this important and thoughtful comment. We fully agree that the Fe Kα_1 first moment is not a rigorously established metric for assigning absolute oxidation states, and we have revised the manuscript to avoid such interpretation. In the revised version, we explicitly use the XES data to describe relative changes in the Fe electronic structure along the reaction coordinate, rather than assigning discrete oxidation states.

Importantly, the spectroscopic analysis is interpreted in combination with the time-resolved crystallographic data, which independently define the sequence of intermediates and demonstrate that mixtures of states are present at each time point (Fig. 2a–c). We emphasise that the XES and tr-SFX datasets were analysed independently, and that the respective interpretations were developed separately to avoid bias; notably, the trends observed by XES are consistent with the structurally defined progression of intermediates. In this context, the XES data provide complementary information on the evolution of the Fe electronic structure.

The observed spectral changes are dominated by a progressive shift of the emission envelope rather than simple peak broadening, as is also evident from the difference spectra (Fig. 2c, Extended Data Fig. 4d). To address the reviewer's concern, we have expanded the analysis to include FWHM and IAD data alongside the first moment (Extended Data Fig. 4g–j). While the difference spectra show that the nature of change is different at later time points versus earlier time points (Extended Data Fig 4d,e), similar with what is seen in the first moment analysis, the FWHM exhibits only minor variation at later time points and does not clearly resolve intermediate stages. This is likely due to the increasingly asymmetric peak shape at later times, combined with a gradual shift of the peak maximum. By contrast, the first moment provides a more sensitive and robust description of the overall spectral shift, in agreement with the trends observed directly in the difference spectra.

To further support the first moment analysis, we have analysed external reference compounds (Extended Data Fig. 4k). We emphasise, however, that our interpretation does not rely on direct comparison to reference compounds for assigning formal oxidation states, but instead focuses on internally consistent trends within the same system. For context, we include Fe model compounds with known formal valence (Extended Data Fig. 4k), based on independently analysed and previously reported datasets (S. Lafuerza et al. Inorganic Chemistry 2020). These data show that the Fe Kα$_1$ first moment exhibits a general, though not strictly linear, correlation with formal valence across different ligand environments, with some deviations, notably for cyanide ligands. While this relationship is not sufficiently robust for quantitative oxidation state assignment, the overall trend is consistent with the relative spectral shifts observed in the IPNS system.

In the revised manuscript, we conclude that the XES data reliably track changes in the Fe electronic structure during catalysis, in agreement with the crystallographic and mechanistic analysis, ensuring to avoid over-interpretation in terms of absolute oxidation state assignment.

Comment:
The authors show error bars for the Fe Kalpha1 first moment below 0.01 eV. The error bar is deduced from the counting statistics. It would be important to discuss systematic errors as well, such as stability of the XES energy calibration. Ideally, the authors should show repeated measurements over time of the same compound to provide an estimate of the stability of the XES energy calibration.

Response:

We thank the reviewer for raising this important point. In the revised manuscript, we clarify that the reported error bars reflect statistical uncertainties derived from counting statistics (Fig.2 legend, Extended Data Fig. 4 legend and Supplementary Material). With respect to systematic uncertainties, all XES datasets were collected within a single experimental campaign using an unchanged spectrometer configuration, thereby minimising potential drift in energy calibration. In addition, the order of data collection was randomised, and consistent spectral trends were observed across independently measured time points, reducing the likelihood that the observed changes arise from systematic effects.

Importantly, the magnitude and nature of the spectral changes, in particular the progressive shift of the emission envelope observed in the difference spectra (Extended Data Fig. 4d,e), are substantially larger than expected variations from instrumental instability and are reproducible across datasets. We therefore interpret the observed trends as intrinsic to the system rather than arising from calibration drift. We have added text to the manuscript to explicitly acknowledge the presence of systematic uncertainties and to clarify that our analysis is based on relative comparisons within a consistent dataset, rather than absolute energy calibration.

Comment:
Finally, it would be important to show all spectra without smoothing in the extended data.
Response:

We agree with the reviewer and have now included the unsmoothed spectra in the Extended Data. These data allow direct inspection of the raw experimental signal without any processing. The spectra shown in the main figures are lightly processed for clarity of presentation; however, all quantitative

analyses (first moment, IAD, and FWHM) were performed consistently across datasets.

Reviewer #2:

Comment:
The IPNS enzyme whose biosynthetic reaction oxidizes the peptide ACV to the bicyclic penicillin backbone provides the substrate from which many clinical antibiotics are derived. Understanding the catalytic formation of the two rings of the penicillin backbone has been elusive. This superb manuscript provides convincing evidence that this involves a ferryl iron with a key role for an active site water. Surprisingly the thioaldehyde intermediate observed does not coordinate the iron. The manuscript contains 25 crystal structures (13 of which are determined by time-resolved serial crystallography supported by trXES). Mutagenesis, mass spectrometry, organic synthesis, NMR and molecular dynamics validate hypotheses that arise from the crystal structures. The data are excellent and every conceivable control has been carried out. This is a very complex manuscript, but the authors do a first-rate job of walking the reader through the data and what these support in terms of mechanism. In particular, the figures provided were comprehensive, and they convincingly supported the manuscript's conclusions. This is an important manuscript on a medically important enzyme. All my specific comments below are minor being typographical or simple fixes.

Response: *We thank the reviewer for the very careful reading of our manuscript and for the generous and positive assessment of our work. We greatly appreciate the positive evaluation and the recognition of details of experimental validation. We have addressed the comments of the reviewe in the revised version as described below.*

Comment: Ln 74: "studied reaction" should be "studied the reaction"

Response: *Corrected.*

Comment: Lns 107 and 110: Fig 1 is not split into panels (a) and (b), so presumably these labels should be "Fig.1 path a" and Fig. 1 path b"

Response: *Corrected. The text now refers to "Fig. 1 path a" and "Fig. 1 path b" as appropriate.*

Comment: Lns 203 and 715: "anaerobicic" should be "anaerobic"

Response: *Corrected in both instances*.

Comment: Ln 224: "ib" should be "at"?

Response: *Corrected to "at".*

Comment: Ln 245: There is no extended fig. 2 "2div" panel. I think the authors mean "2aiv"? It is Figure 2a iv

Response: *Thanks, the correct reference is Extended Data Fig. 2aiv. This has been corrected.*

Comment: Ln 285: There is no extended data fig 2ciii. However, Fig. 2biii in the body of the paper shows increased Fobs difference density for the ring sulfur after 10s compared to the 8s structure. This supports partial formation of the IPN in PDB 913D. Do they mean this figure?

Response: *Thanks, the intended reference is Fig. 2biii. This has been corrected*.

Comment: Ln 291: What is shunt product (1)? The hydrated aldehyde of extended fig. 5o?

Response: *Yes, shunt product (**1**) corresponds to the hydrated aldehyde shown in Extended Data Fig. 5o. This has now been clarified in the text.*

Comment: Ln 293-5: Extended Fig. 3(o,p) should be associated with this sentence.

Response: *Thank you for pointing this out. We have now added the correct reference, which is Extended Data Fig. 3o-q.*

Comment: Ln 296: "Fig2c" should be "Fig. 2c"

Response: *Corrected.*

Comment: Ln 318: None of the ribbon figures in fig. 2 and extended data fig. 1 show where the active site is located in reference to alpha10, so one cannot envision the relationship between the two. Are there direct lattice packing interactions involving helix 10?

Response: *We thank the reviewer for raising this important point. α10 is indeed involved in crystal lattice interactions. Previous crystallographic work has shown that lattice packing prevents release of the IPN product in crystallo (P. Rabe et al. Science Advances 2021). We have included a figure in the SI to visualise this lattice packing (Supplementary Fig 2). This feature is central to our experimental approach, as it enables us to follow a single catalytic turnover event within the crystal. Because the role of lattice contacts in restricting product release has been established previously and is not directly related to the mechanistic focus of the present study, we have not expanded further on these contacts. To improve clarity, we have revised Fig 2 and Extended Data Fig. 1 to more clearly indicate the position of the active site relative to helix α10, and we have clarified the corresponding text in the figure legend.*

Comment: Ln 325: Sentence ends with a comma.

Response: *Corrected.*

Comment: Ln 353: "susbtitued" should be "substituted".

Response: *Corrected.*

Comment: Ln 390: "timpoints" should be "timepoints"

Response: *Corrected.*

Comment: Ln 411: Point to panel a of extended data fig. 6 rather than the whole of the figure. "Extended Data Fig. 6a".

Response: *Corrected. The text now refers specifically to Extended Data Fig. 6a.*

Comment: Ln 423: I believe "Fig. 4j" should be "Fig. 3j"

Response: *Corrected.*

Comment: Ln 425: I believe "Fig3j" should be "Fig. 3j"

Response: *Corrected.*

Comment: Ln 427: I believe "Fig. 4h" should be Fig. 3h"

Response: *Corrected.*

Comment: Ln 435: "Fig. 2" should reference the specific panel a, i.e. "Fig. 2a"

Response: *Corrected.*

Comment: Ln 441: "(Fig. 3g)" should be "(Fig. 3g))"

Response: *Corrected.*

Comment: Ln 450: I believe "Extended Data Fig. 2.8fi" should be "Extended Data Figs. 2c-g, 8fi"

Response: *Corrected to "Extended Data Figs. 2c-g, 8fi".*

Comment: Ln453: I believe "Extended Data Fig. 2,S8a-c" should be "Extended Data Figs. 2c-g, 8a-c"

Response: *Corrected to "Extended Data Figs. 2c-g, 8a-c".*

Comment: Ln 488: "interactions." Should be "interactions)."

Response: *Corrected.*

Comment: Ln 600: "os" should be "of"

Response: *Corrected.*

Comment: Ln 660: Missing space between "O2" and "with"

Response: *Corrected.*

Comment: Ln 670: The "2" of "10s O2" should be subscript thanks

Response: *Corrected.*

Comment: Ln 767: "occupanc" should be "occupancy"

Response: *Corrected.*

Comment: Ln 831: "The +11 charge state was shown" should be "The +11 charge state is shown"

Response: *Corrected.*

Comment: Fig. 2bi: The label box around "Sulfur release from Fe" needs to be extended to cover all the text.

Response: *The label box has been adjusted to fully include the text.*

Comment: Extended Fig. 7dii. The top trace is labelled as showing "HPLC purified 2 and 3" but the trace only contains 3 and 5.

Response: *Thank you for identifying this oversight. This error originated from an earlier version of the manuscript with different compound numbering. The label has now been corrected to reflect the appropriate compounds.*

SUPPORTING INFORMATION

Comment: Lns 151, 155, 160: "Carry" should be "Cary"

Response: *Corrected.*

Comment: Ln 157: "anerobic" should be "anaerobic"

Response: *Corrected.*

Ln 347-8: "Assay samples were aspirated under vacuum for 0.6 s and loaded onto a" should be removed as the complete sentence starts the next paragraph that immediately follows.

Response: *Corrected.*

Reviewer #3

Comment: Isopenicillin N synthase (IPNS) plays a pivotal role in the biosynthesis of penicillin and cephalosporin antibiotics, making it a subject of significant interest in biochemical research. The enzyme's unique catalytic mechanism, which involves the formation and transformation of high-energy intermediates, has been challenging to characterize due to the fleeting nature of these transient states. Previous studies have contributed valuable information regarding the structure and function of IPNS; however, important gaps remained, particularly in the detailed understanding of the intermediates that arise during catalysis. To address these gaps, the authors emphasize the necessity of advanced experimental methods, such as time-resolved crystallography, which can capture the rapid and subtle changes that occur within the enzyme during its reaction cycle.

This is a valuable approach to tackle the problem to understand the enzymatic process of IPNS. It would be appreciated if the authors describe what practical implications that may have to the development of new antibiotics in this field and /or the improvement of current synthesis.

Response: *We thank the reviewer for raising this important point. While the present study focuses on mechanistic elucidation of IPNS catalysis, the insights obtained here have implications for rational engineering of β-lactam biosynthetic enzymes. In particular, our results reveal how subtle perturbations of second-shell residues and active-site solvent organization influence ferryl formation, Fe–S coordination, and product outcome. The observation that small structural changes can redirect the reaction trajectory, as demonstrated by the N252 variants, provides a structural framework for modulating catalytic efficiency and suppressing shunt pathways. We have added a sentence in the Discussion to identify the broader implications.*

Experimental Approach

Comment: To investigate the catalytic mechanism of IPNS, the authors utilized a combination of time-resolved serial femtosecond crystallography and X-ray emission spectroscopy (XES). These methods enabled the observation of both structural transformations and changes in the redox state of iron within the enzyme as the reaction progressed. The experimental setup involved reacting anaerobic IPNS·Fe(II)·ACV microcrystals with molecular oxygen and monitoring the ensuing changes at various time intervals. In addition, the use of deuterium-labelled substrates allowed for enhanced resolution in distinguishing subtle mechanistic features. Mutagenesis and computational studies were also performed to support and validate the proposed mechanistic pathways.

Results

Comment: The integrity and effectiveness of the sample delivery system were confirmed through initial experiments, which successfully initiated the reaction by exposing microcrystals to oxygen. Structural analyses revealed the sequential formation of key intermediates within the catalytic cycle. Notably, the authors observed the emergence of an Fe(III)-superoxo intermediate, followed by the displacement of the ACV-derived sulfur atom, which led to the formation of a thioaldehyde intermediate that does not coordinate with iron. Electron density and isomorphous difference maps provided detailed visualizations of these events. The data demonstrated that the reaction proceeds through several distinct intermediates, including a crucial monocyclic intermediate (MCI) essential for constructing the β-lactam ring.

As the reaction advanced, the formation of the MCI and the subsequent restoration of the Fe-S bond were documented, supported by comparisons with known substrate analogs. The results highlighted the significant role of a water chain present in the enzyme's active site, which serves to stabilize the intermediates and facilitate the overall reaction process. The findings also provided insights with broader implications for the family of 2-oxoglutarate-dependent oxygenases.

Figure 2: The 24-hour time point needs to be explained a little bit more in detail, as the maintenance of stable crystal conditions can be difficult for such a long time compared to seconds.

Response: *We thank the reviewer for this important point. The stability of IPNS crystals under prolonged O_2 exposure has previously been demonstrated ([P. Rabe et al., Sci. Adv. 2021]), where it was shown that lattice packing prevents product release and permits accumulation of the IPNS:Fe:IPN complex in crystallo (new Supplementary Fig. 2). In the present study, crystal integrity after 24 h O_2 exposure was confirmed by maintained diffraction to high resolution and refinement statistics comparable to earlier timepoints. While the IPN product remains bound in the active site, we observe a reduction in Fe occupancy over prolonged exposure. We attribute this to partial oxidation of Fe(II) under conditions of excess O_2, which lowers metal affinity and can lead to gradual dissociation, consistent with previous observations. Importantly, the overall protein structure and lattice order remain intact, and the dataset quality confirms that crystal stability is preserved over this timescale. We have clarified these points in the revised manuscript.*

Comment: Figure 2c, the colours for the different time points should be more varied to better differentiate.

Response: *The colour schemes in Figure 2ci and 2cii has been revised to improve contrast and differentiation between the individual timepoints.*

Comment: In Fig 2 b: For the isomorphous difference maps: Are they all on the same level in i, ii, iii?

Response: *We have clarified the contour levels in the figure legend (3.0* σ contour level) *and confirm that the maps shown are contoured consistently.*

Comment: The sulfur atoms are not in the center of the green(positive) difference in ii, iii any explanation, iv is even more difficult to understand.

Response: *We thank the reviewer for this careful observation. The sulfur atom does not relocate to a completely new discrete position but instead shifts along a defined trajectory while partially overlapping with its original site. The datasets represent mixed populations of coexisting intermediate states, resulting in structural averaging. Consequently, the positive isomorphous difference density reflects the net shift between these partially overlapping positions rather than a fully displaced sulfur atom, and therefore is not centred exactly on the refined sulfur coordinates. We have clarified this point in the revised Fig. 2b legend to better guide interpretation, particularly for panel iv.*

Mutagenesis experiments

Comment: Second shell mutagenesis of N252 to A, E and Q shows considerable influence on the reaction kinetic which in the found structures show the importance of several water molecules.

First shell mutations like H270E revealed the involvement of the Fe bound water in peroxide cleavage and thioaldehyde formation.

These experimental results are further analyzed with computational modeling to achieve a complete reaction coordinate explanation in detail. It would be helpful in the discussion if the authors clearly

mentioned where specific experimental data are missing just to state that not all points e.g., can be measured.

Response: *We thank the reviewer for this important point. We agree that clear distinction between experimentally observed intermediates and mechanistic steps inferred from computational modelling or indirect evidence is essential. While the time-resolved XFEL data directly capture the Fe-superoxo-ACV, Fe-hydroperoxo-thioaldehyde, Fe(IV)=O-monocyclic intermediate, and product states, certain elementary steps including peroxide cleavage and ferryl formation mediated by Fe-bound W1, cannot be directly visualized due to their transient nature and mixed occupancies. These steps are instead supported by indirect experimental evidence, including the H270E variant, which disrupts Fe-bound W1 coordination and stalls catalysis at the thioaldehyde stage, together with computational modelling. We have revised the Discussion to more clearly distinguish directly observed intermediates from mechanistic inferences and to state these experimental limitations explicitly.*

Comment: In general: the presented pdb structures should be clearly assigned to certain beamlines (LCLS, PAL XFEL, SACLA and DLS), not only in the supplement table.

Response: *We have revised the main text and figure legends to clearly indicate the beamline source for the principal datasets, in addition to the Supplementary Information, to improve transparency and readability.*

Comment: The data coming from the diamond beam line (DLS) are not specifically mentioned ("by traditional, rotation-based methods under cryogenic conditions," this for non-crystallographers impossible to decipher) or compared with the SFX data.

Response: *We have clarified in the main text that these datasets were collected at Diamond Light Source using conventional rotation-based synchrotron crystallography under cryogenic conditions. We also added brief explanatory wording to make this clearer to non-crystallographers*.

Comment: The comparison between tr-sfx and tr-synchrotron data would be of interest as some structures in the paper have only been determined by standard cryogenic methods at DLS (why not at RT).

Response: *We have added text discussing the complementary nature of tr SFX and conventional synchrotron crystallography, including practical considerations that motivated the use of cryogenic synchrotron methods for certain variants. Where relevant, we comment on similarities between datasets.*

Comment: The data table in the supplement raises the question for the 24 h experiment row 9 and 14, which data are presented the DLS and or LCLS and what was the difference if there was any for the claim of the B-factor change.

Response: *We thank the reviewer for raising this point. The 24 h O_2 exposure experiment was first performed using microcrystal slurries that were allowed to undergo full turnover. These crystals were initially analysed at Diamond Light Source (I24) using serial synchrotron crystallography (SSX) to validate complete IPN formation. After confirming product formation, the same crystal preparation was cryo-preserved in liquid N_2 and subsequently analysed at LCLS using the tape-drive setup to collect complementary SFX data and simultaneous X-ray emission spectroscopy (XES), which is not available at DLS. This staged approach allowed validation of full turnover prior to XFEL measurements and ensured efficient use of limited deuterated substrate and allocated LCLS beam time.*

Both the DLS and LCLS datasets therefore originate from the same crystal batch but were collected under different experimental conditions. Structural comparisons show no significant differences in overall protein architecture or product binding between the datasets. Importantly, the B-factor analysis presented in the manuscript was performed exclusively on SFX datasets refined under identical protocols. The 24 h dataset discussed in this context refers to the SFX structure (LCLS) and is therefore directly comparable to the other SFX timepoints. The DLS dataset was not included in the B-factor analysis. While small differences in overall global B-factors can arise from variations in exposure conditions between diffraction sources, these do not affect the normalized local B-factor trends within the SFX datasets. We have clarified these points in the revised Extended Data Fig. 3 legend. Both datasets are included to appropriately acknowledge the contributions and experimental support provided at Diamond Light Source (I24).

Discussion

Comment: In summary, the study elucidates the dynamic nature of IPNS catalysis, revealing the importance of both protein conformational flexibility and the participation of water molecules in the reaction mechanism. The authors acknowledge the inherent limitations in resolving every intermediate due to their transient nature, and they recognize the need for further investigation to fully map the entire catalytic pathway. Future research is recommended to focus on the roles of specific amino acid residues and the dynamics of solvent molecules, aiming to further deepen the understanding of enzyme catalysis at a molecular level.

This is a plethora of data to give detailed insight into a reaction coordinate of an important catalytic process. The presentation should follow an overview detail scheme for the results and discussion part.

Response: *We thank the reviewer for this helpful suggestion. To improve the overview–detail structure and guide the reader more clearly through the catalytic sequence, we have revised the opening of the Results section to include a concise roadmap outlining the sequence of intermediates and subsequent analyses. We have also refined the opening of the Discussion to more explicitly frame the mechanistic overview before presenting detailed interpretation. These changes improve readability and logical flow, particularly for non-specialist readers.*

Comment:

Suggestions for improvement. The motivation for tr-SFX and XES should be better explained and with tr SSX compared. In the discussion the model described for the reaction coordinate should be more critically discussed in a way the highlights and shortcomings are expressed. Overall: I recommend the paper with minor revisions.

Response:

We thank the reviewer for this constructive suggestion. We have revised the Introduction to more clearly justify the use of tr-SFX in combination with XES, explicitly highlighting that radiation-sensitive high-valent Fe intermediates can only be reliably monitored under XFEL conditions due to the absence of radiation damage, and that XES measurements are uniquely enabled in this experimental setup. We also clarify the complementary role of the single SSX dataset included in the manuscript.

In the Discussion, we have refined the presentation of the proposed reaction coordinate to more explicitly distinguish directly observed intermediates from mechanistic steps inferred from indirect experimental evidence and computational modelling. In particular, we now state clearly that the peroxide cleavage and ferryl formation steps cannot be directly visualized due to transient lifetimes

and mixed occupancies, and we discuss the strengths and limitations of the mechanistic model accordingly.

Reviewer #4:

Comment: Most 2OG-dependent oxygenases catalyse two-electron hydroxylation reactions coupled to the oxidative decarboxylation of 2OG to succinate. In contrast, IPNS uniquely catalyses a spatially orchestrated four-electron oxidation that converts the tripeptide ACV into the highly strained penicillin scaffold without the involvement of 2OG. Although IPNS has been extensively studied, understanding how precise atomic positioning and enzyme dynamics enable catalysis involving high-energy intermediates remains a central challenge in structural biology. In particular, the structures of the proposed Fe(II)–hydroperoxide thioaldehyde and Fe(IV)=O monocyclic intermediates implicated in the key ring-forming steps have thus far remained elusive.

In this manuscript, the authors harness the power of tr-SFX in combination with tr-XES to probe transient intermediates during IPNS catalysis. Using anaerobic IPNS·Fe(II)·ACV microcrystals as the starting point, the reaction was initiated by controlled exposure to O_2 and followed using XFEL-based structural and spectroscopic approaches. The resulting time-course structures reveal that cysteinyl oxidation of ACV generates a thioaldehyde intermediate that, unexpectedly, does not coordinate the iron center. Subsequent experiments employing isotopically labelled ACV demonstrate the formation of a monocyclic β-lactam intermediate accompanied by restoration of the Fe–S bond. When combined with mutagenesis, spectroscopic, and computational analyses, the XFEL results support a mechanism in which oxidation of the ACV valine residue in the monocyclic intermediate is mediated by an Fe(IV) species. Moreover, a hydrogen-bonding network involving three water molecules appears to play a crucial role in stabilising the intermediates, particularly by facilitating Fe(IV) coordination of the monocyclic intermediate prior to isopenicillin N formation.

Overall, this work is beautifully executed and thoughtfully designed. It compellingly highlights the power of time-resolved XFEL approaches for characterising transient intermediates in oxygenase catalysis. This work offers valuable insights into the significant roles of water molecules in enzymatic reaction mechanisms that are challenging to dissect. Nonetheless, a few points merit further consideration, as outlined below.

Comment: 1. In the computational section, the authors state that proton transfer from IM-II via water molecule W4 to yield the IPNS:Fe(IV)=O:MCI complex (IM-IV) was energetically unfavourable, instead identifying an intermediate IM-III in which reorientation of the ACV valine methyl groups and W4 enables formation of the monocyclic intermediate (IM-IV) by overcoming an activation barrier of 26.7 kcal mol^{-1}. As these mechanistic conclusions rely heavily on quantum mechanical calculations, the authors are requested to provide the atomic coordinates of the QM region used in the calculations to ensure reproducibility and independent evaluation. In addition, the authors should clarify why the reorientation of the ACV valine methyl groups and W4 was not captured in the tr-SFX analysis.

Response: *We thank the reviewer for this comment. The Cartesian coordinates of all QM region structures (intermediates and transition states) are now provided in the revised Supporting Information.*

Our time-resolved data imply reorientation of the ACV valinyl side chain during catalysis. In the substrate-bound state, the Val Cβ–H bond points away from the Fe centre, whereas at the MCI stage it is oriented towards the Fe in a geometry consistent with Val Cβ C–H cleavage. At intermediate

(thioaldehyde) time points, this conformation cannot be unambiguously defined due to side chain flexibility and weak electron density. The proposed reorientation is illustrated schematically in Fig. 4 and we have added an appropriate comment to the figure legend.

Comment: 2. The authors employ substrate or cofactor analogues, such as vanadium in place of iron, in the crystallographic studies to elucidate the structural basis of catalysis. To further support the relevance of these analogues to the native reaction, it would be helpful to provide corresponding biochemical data demonstrating that the analogue faithfully reflects the behaviour of the native metal cofactor.

Response: *We thank the reviewer for this important point. The V(IV)=O complex was not employed as a direct functional substitute for Fe during catalysis, but rather as a structurally stable high-valent metal-oxo mimic to assess metal-oxo geometry and evaluate potential radiation-induced bond elongation in the XFEL datasets. As noted in the manuscript, formation of a catalytically competent Fe(IV)=O:MCI complex is transient and cannot be isolated or stabilised for conventional crystallographic analysis due to rapid turnover and decomposition. The vanadyl complex therefore serves for geometric comparison rather than a mechanistically active analogue. Importantly, we confirmed binding of V(IV)=O to IPNS in the presence of ACV by non-denaturing mass spectrometry and EPR spectroscopy, supporting formation of a well-defined metal–protein–substrate complex (data not shown). However, we do not imply that V(IV)=O reproduces the catalytic behaviour of Fe(IV)=O. We have explicitly clarified this distinction in the revised manuscript.*

Comment: In addition, in the HPLC profile shown in Extended Data Fig. 7, the identities of the peaks at retention times of 3.4 min and 4.2 min should be clarified.

Response: *We thank the reviewer for requesting clarification of the minor LC-MS peaks. We have revised the Extended Data Fig. 7 legend to describe the species observed at ~4.3 min (m/z 543.7171, 2+ charge state) and ~3.4 min (m/z 454.1316). The higher-mass species at ~4.3 min is consistent with formation of an oligomeric product under oxidative conditions, likely arising from partial coupling of multiple ACV and/or thioaldehyde units. We attempted purification of this species; however, the isolated material consisted of mixtures of dimeric and potentially trimeric ACV-derived species. As these oxidative coupling products likely form via disulfide linkages, extensive signal overlap and closely related masses prevented unambiguous structural assignment. Accordingly, we have described this species conservatively in the revised legend. The peak at ~3.4 min (m/z 454.1316) is approximately 32 Da higher than iodoacetamide-protected ACV (422.1596 Da), consistent with additional oxidative modification. This species was not characterised, and we have stated this explicitly in the revised text.*

Comment: 3. Although the difference spectra obtained from Fe $K\alpha_{1,2}$ XES analysis suggest changes in the iron electronic structure, these features appear relatively subtle. It would therefore be helpful if the authors could provide complementary experimental evidence, such as Mössbauer spectroscopy, to further substantiate the proposed electronic and/or spin-state assignments.

Response: *We thank the reviewer for this thoughtful suggestion. We agree that Mössbauer spectroscopy could provide valuable insight into oxidation and spin states of iron intermediates. Indeed, prior solution-phase studies on IPNS employing Mössbauer spectroscopy have independently identified Fe(III)-superoxo and Fe(IV)=O intermediates consistent with the mechanistic assignments proposed here (*J. Am. Chem. Soc. 2016*).These findings are fully in line with our structural and tr-XES observations.*

However, the present study focuses on time-resolved XFEL measurements on reacting microcrystals using the acoustic droplet ejection tape-drive method. Reproducing these transient crystal-based O_2

exposure conditions in a manner compatible with Mössbauer freeze-trapping is currently experimentally not feasible, particularly given the requirement for synchronised O_2 exposure, short time delays, and preservation of crystal integrity. The high-valent species characterised here are extremely short-lived and observed in mixed occupancy states within crystals, making conventional freeze-trap Mössbauer experiments under identical conditions experimentally challenging.

Importantly, the Fe $K\alpha_{1,2}$ XES first-moment shifts have been supported by additional analysis of difference spectra, integrated absolute differences (IAD), full width half max (FWHM) and half width half max (HWHM) analysis as well as with analysis of reference compounds data in the revised manuscript and are internally consistent across timepoints and correlate directly with the structural transitions observed by tr-SFX. While the spectral changes are subtle, they are reproducible and are interpreted in conjunction with crystallographic and computational data. We have clarified in the revised manuscript that the electronic-state assignments are supported by this combined evidence and are consistent with previously reported Mössbauer characterisation of IPNS intermediates.

Reviewer #5:

In this work a detailed study is presented on isopenicillin N synthase, which is an unusual nonheme iron dioxygenase where dioxygen triggers four successive hydrogen atom abstractions (formally) resulting in two ring-closure reactions in the substrate. There are still details missing on the mechanism and multiple suggestions have been proposed for this. A large team from various countries report here crystallography, spectroscopy and computational studies. Particularly the trapping of these short-lived intermediates is impressive and gives insight into this important reaction and elucidates details of the mechanism. I am not an expert in crystallography so cannot argue about the methods, procedures etc, so will focus on the computation and general topic mostly. Overall, the paper fits the remit of Nature Catalysis well, but some changes and maybe additional work will benefit the overall discussion.

Comment: 1. If water is essential for catalysis, there should be a solvent isotope effect when D2O is used. Has the experiment with D2O been attempted?

Response: *We thank the reviewer for this important point. We have explored solvent isotope effects using stopped-flow UV/Vis experiments monitoring formation and decay of the ferryl intermediate under conditions closely related to those reported by Bollinger and co-workers (J. Am. Chem. Soc. 2016). Under comparable single turnover conditions, we observe differences between reactions performed in H_2O and D_2O, consistent with involvement of solvent derived protons in steps associated with ferryl formation and/or decay.*

In addition, comparison of IPNS:Fe:AC(d)V microcrystals grown in H_2O versus D_2O and reacted with O_2 using the tape drive system reveals differences in active site electron density features. Although these differences do not allow quantitative kinetic analysis in crystallo, they are consistent with an influence of solvent composition on the reaction coordinate. We note, however, that appropriate quantitative interpretation of solvent isotope effects in this mechanistically complex system is experimentally challenging. Differences in O_2 solubility, viscosity and diffusion properties between H_2O and D_2O complicate direct comparison under stopped-flow and tape drive conditions, particularly in single turnover experiments in solution and on crystals. Accordingly, multiple independent repeats using tr-SFX analyses are required for reliable kinetic analysis, and we are currently investigating this further.

At present, we believe that a full mechanistic dissection of the solvent isotope effect would extend well beyond the focus of the current study. We have therefore limited ourselves to reporting qualitative observations and will address this aspect in future work.

Comment: 2. Did the authors use deuterated substrate to establish rates and kinetic isotope effects?

Response: *The kinetic isotope effects associated with the two C–H bond cleavage steps in IPNS catalysis have previously been characterised in detail by Bollinger, Krebs, and co-workers (J. Am. Chem. Soc. 2016), combining stopped-flow UV–Vis and Mössbauer spectroscopy under rigorously controlled single-turnover conditions with enzyme in solution. That work established substantial primary kinetic isotope effects for the initial Cys Cβ–H abstraction and for the subsequent Val Cβ–H cleavage step, thereby defining both the sequence and the relative rate contributions of the two hydrogen abstraction events.*

In the present study, we exploited the previously established large kinetic isotope effect for the Val Cβ–H cleavage step by employing ACdV to promote accumulation of the monocyclic intermediate in crystallo. We did not repeat the full solution kinetic analysis with newly synthesised deuterated substrates, as the synthesis is non-trivial and costly, and the key kinetic parameters have already been determined with high precision using complementary spectroscopic approaches (stopped-flow UV–Vis and Mössbauer spectroscopy).

Our focus here was on structural trapping and characterisation of intermediates, rather than re-determination of kinetic parameters that have already been carefully established. We have clarified this point in the revised manuscript and now explicitly reference the prior detailed kinetic and Mössbauer study.

Comment: 3. Computation: If water molecules are important for the mechanism, why were these not included in the QM region?

Response: *We thank the reviewer for this comment. In the standard QM region, only the Fe-coordinated water molecule (W1) was included to capture the primary coordination environment of the active site. In response to the reviewer's suggestion, we have performed additional QM/MM calculations for the first step of the reaction, strating from the Fe(III)–superoxo complex leading to the formation of the IPNS:Fe(II):HO_2:TAL intermediate (IM-I), using an extended QM region that includes W1 along with the catalytically relevant second-sphere water molecules (W2 and W3) and the SCS residue Asp252. Importantly, the computed activation barrier (12.7 kcal/mol) and reaction energy (4.8 kcal/mol) obtained with the extended QM region are consistent with those from the standard QM region (10.6 and 2.4 kcal/mol, respectively). The new studies have been updated in the revised manuscript.*

Comment: 4. I am surprised the authors did not use dispersion in the QM/MM calculations.

Response: *We have performed additional single-point calculations, including Grimme's D3 dispersion correction on the stationary structures along the reaction pathway. The resulting activation barriers and reaction energies are largely consistent with those obtained without dispersion. Notably, inclusion of dispersion leads to a modest decrease (~3 kcal/mol) in the activation barrier for formation of the IM-V intermediate. Accordingly, the QM/MM reaction energy profile has been updated using D3-corrected relative energies.*

Comment: 5. A QM/MM basis set of def2-SVP may be to modest to get good geometries.

Response: *Additional QM/MM calculations were performed by re-optimizing the stationary structures involved in the first step of the reaction mechanism using the larger def2-TZVP basis set. The resulting activation energy remains comparable (10.7 kcal/mol) with that obtained using the standard level of theory (10.6 kcal/mol).*

Comment: 6. There are no MD and QM/MM data in the SI. Please add. I also would include Cartesian coordinates of the QM region structures of all intermediates and transition states.

Response: *We thank the reviewer for this important suggestion. In response, we have now included the relevant MD and QM/MM data in the Supporting Information. Specifically, we have added analyses of RMSD, RMSF, radius of gyration (RoG), and solvent-accessible surface area (SASA) to characterize the stability and dynamics of the system. We have also included time-dependent fluctuations of key distances relevant to the reaction mechanism, particularly the distance between the distal oxygen (Od) and the cysteinyl C-3 carbon involved in the first hydrogen abstraction step leading to peroxide formation.*

We have also provided Cartesian coordinates for all QM region structures corresponding to the optimized intermediates and transition states along the reaction pathway.

Comment: 7. I do not see the activation barrier of 27.2 kcal/mol mentioned in the text in Figure 4. Also, a barrier of 27.2 kcal/mol as well as the energy gap between TS-IV and IM-III of 33.4 kcal/mol seems a bit high for barriers for an enzymatic process. These barriers may be too slow for an enzyme that works at room temperature. How do these barriers compare with experiment?

Response: *We thank the reviewer for this comment. We have corrected the error in reporting the activation energy (27.2 kcal/mol) in the manuscript.*

We agree with the reviewer that an energy gap of 33.4 kcal/mol (30.7 kcal/mol after the inclusion of D3 correction) between TS-IV and IM-III is relatively high for an enzymatic reaction. However, previous computational studies have shown that conformational variability in the starting structure can significantly influence the activation energy by several kcal/mol (J. Chem. Theory Comput. 2017, 13, 11, 5745–5752, ACS Catal. 2024, 14, 24, 18550–18569). We therefore performed additional QM/MM calculations using an alternative snapshot obtained from MD simulations of the Fe(III)–superoxo complex. The resultant computed activation and reaction energies consistently show substantial variation, particularly for the formation of the IM-II, IM-IV, and IM-V intermediates, highlighting the influence of conformational variability on the energetics.

We also performed single-point calculations using the PBE0 functional, which further demonstrate that the activation and reaction energies can vary depending on the choice of functional used. Furthermore, experimental studies show the hydrogen-atom-transfer step in IPNS catalysis exhibits a large kinetic isotope effect (KIE) (J. Am. Chem. Soc. 2016, 138, 28, 8862–8874), suggesting the involvement of quantum tunneling (Biochemistry 2026, 65, 3, 237–248, Annu. Rev. Biochem. 2013. 82:471–96). The computed activation energies should therefore be interpreted with caution, as tunneling can effectively compensate for a higher barrier under physiological conditions. Experimental evidence also suggests correlated motions that change during the catalytic cycle (Sci. Adv. 2021; 7: eabh0250), which might not be well captured by static QM/M calculations and could also influence the observed activation energy, as also reported in ChemCatChem 2020, 12, 4704 – 4720, Chem. Eur. J. 2023, 29, e202301305, Biophysical Journal 104(5) L05–L07, Biophysical Journal, 92, L20-L22).

Overall, while the energy gap of 30.7 kcal/mol appears high, we emphasize that results based on a single snapshot should be treated cautiously. In particular, the relative energy of IM-IV varies by ~13

kcal/mol, while the barrier from TS-IV to IM-V varies by only ~3 kcal/mol. The energetics can vary substantially depending on the initial conformational state, correlated motions of the enzyme during the reaction mechanism, and the computational methodology employed. The experimentally observed KIE further supports that the effective barrier for the reaction may be lower than the relative energies obtained from static QM/MM calculations.

Comment: 8. The authors may want to compare the optimized geometries with calculations on other thiol dioxygenases (cysteine dioxygenase, ovothiol biosynthesis enzyme etc).

Response: *We thank the reviewer for this suggestion. While IPNS follows a distinct catalytic pathway compared to other thiol dioxygenases such as cysteine dioxygenase (CDO, J. Phys. Chem. A 2012, 116, 1, 582–591, J. Am. Chem. Soc. 2011, 133, 11, 3869–3882) and OvoA (an ovothiol biosynthesis enzyme, ACS Catal. 2019, 9, 1, 253–258), the reaction mechanism similarly initiates from the formation of an Fe(III)–superoxo intermediate, as established in prior computational and experimental studies.*

Comparison of the optimized geometries of the Fe(III)-superoxo complex shows that mechanistically important structural parameters, including the Fe-S and Fe-O_2 distances, as well as the Fe-O distance in the EF–III intermediate, are consistent with those observed in previous computational studies on related systems such as CDO and EgtB (ergothioneine biosynthesis enzyme) (J. Phys. Chem. A 2012, 116, 1, 582–591, J. Am. Chem. Soc. 2011, 133, 11, 3869–3882, ACS Catal. 2018, 8, 7, 5875–5889, J. Am. Chem. Soc. 2017, 139, 27, 9259–9270). We have added this to the revised manuscript.

***Comment:** 8. Intro line 2, typo in penicillin.*

Response: *Corrected.*

POINT-TO-POINT RESPONSES TO THE REVIEWERS' COMMENTS:
We are grateful to the reviewers and editors for their careful evaluation of our manuscript and for their constructive comments throughout the review process. We appreciate the positive assessment of the revised manuscript and have addressed the remaining reviewer points as outlined below.

Reviewer #2:
Remarks to the Author:
Addressing concerns of reviewer 1:

Comment:
The authors have added XES Kalpha raw data, which confirms the validity of the smoothed curves. I also found the difference spectra in Extended Fig. 4d supported the significant change in the iron electronics suggested by the tr-SFX and mechanistic studies. They also added IAD (panel g) and HWHM data for the Kalpha1 and 2 regions (panels h-j) along with reference model compound data (panel k). Although formal oxidation state cannot be assigned from the model compounds the trend is clear and changes in the tr-XES Kalpha first moment analysis and the proposed changes in oxidation states align with the model compounds. In the revised manuscript the authors emphasize that the trends in the XES data are consistent with the proposed changes in iron electronic state based on the tr-SFX and mechanistic analysis. I believe that the authors have successfully made the case for the tr-XES Kalpha data to be included. The analysis and interpretation in the revised manuscript adds substantive value and support and does not over interpret the XES.
Response:
We thank the reviewer for this positive assessment. We are pleased that the additional analyses and the revised interpretation satisfactorily address the previous concerns and strengthen the mechanistic conclusions.

Comment:
Extended Figure 4f legend: “formation of stronger change in the electronic structure which is consistant with interpretation of the tr-SFX data as a Fe(IV)=O species”. I feel “stronger change” is not a good description of the suggested electronic change. Perhaps “significant change” or “pronounced change”? By the way, “consistent” is mis-spelt in this sentence.
Response:
We thank the reviewer for identifying these issues. The legend has been revised to replace "stronger change" with "significant change", and the spelling of "consistent" has been corrected.

Comment:
The arguments the authors present in the rebuttal convince me that the stability of the XES energy calibration is such that errors due to instrument instability are not a concern and that errors are primarily a result of counting statistics.
Response:
We thank the reviewer for this assessment and are pleased that the additional explanation and analyses satisfactorily resolved these concerns.

Reviewer 2:
Comment:
The authors have addressed all my points to my satisfaction. The changes to Fig. 2a and Extended Data 1, plus the new SI Fig.2 have made the relationship between the active site and helix 10 very clear. SI Fig. 2 is also very helpful for visualizing the lattice contacts and the role played in slowing product release.
Response: We thank the reviewer for these encouraging comments and are pleased that the additional structural analyses and figures have improved the clarity of the manuscript.

Reviewer #3:
Comment: Overall, the manuscript has improved from the original version. Most of the suggestions raised have been implemented. Just below listed figures need better contrast in labeling to convey the message.
Figures: In Figure 2a: the O2 exposure time needs to be in black (on red background) otherwise it is not readable.
In 2c the difference between depiction of (i) and (ii) is not clear. The description of both must be clarified. Additionally, the x- (eV) and y- axis (a.u.) should be for easy comparison have the same metric size.
Extended Figure 1: the O2 exposure time needs to be in black (on red background) otherwise it is not readable.
Response: We thank the reviewer for these suggestions and have made the requested changes to the figure for easier visualisation and have added a clarification regarding Fig 2c I and ii.

Comment: Extended Data Fig. 3: I hope the weekly defined penicillin CO is just weakly.
Response: We have corrected this typo in Extended figure 3.

Comment: In summary, the manuscript is very technical and would for a broader audience need more 30,000 feet perspective. However, the research topic of a detailed reaction coordinate investigation of a truly relevant medical process is important and due to the use of varies innovative techniques represents a valuable approach.
Response: We thank the reviewer for these thoughtful comments. We have made several changes to improve the accessibility of the manuscript for a broader readership, including clarifying the text and simplifying the presentation by splitting complex figures into smaller, more focused figures that are easier to interpret.

Reviewer #4:
Comment: Remarks to the Author:
The authors have provided detailed explanations and responses to the questions and concerns I raised. Meanwhile, the corresponding contents have been supplemented and modified in the revised draft. The current version can be considered for acceptance in the journal.
Response:
We sincerely thank the reviewer for their constructive comments throughout the review process and for recommending the manuscript for publication.

Reviewer #5:
Comment: Remarks to the Author:
All issues have been addressed well, but I spotted a typo in the conclusion section: "2-oxolutarate" -> 2-oxoglutarate.
Response: We sincerely thank the reviewer for their constructive comments throughout the review process. We thank the reviewer for identifying this typographical error. The text has been corrected to read "2 oxoglutarate".
